# High-throughput sequencing analysis identified microRNAs associated with egg production in ducks ovaries

**DOI:** 10.7717/peerj.8440

**Published:** 2020-02-04

**Authors:** Mohan Qiu, Zengrong Zhang, Xia Xiong, Huarui Du, Qingyun Li, Chunlin Yu, Wu Gan, Hehe Liu, Han Peng, Bo Xia, Jialei Chen, Chenming Hu, Xiaoyan Song, Li Yang, Xiaosong Jiang, Chaowu Yang

**Affiliations:** 1 Sichuan Animal Science Academy, Chengdu, China; 2 Animal Breeding and Genetics Key Laboratory of Sichuan Province, Chengdu, China; 3 Shanghai Ying Biotechnology Company, Shanghai, China; 4 Sichuan Agricultural University, Sichuan, China

**Keywords:** Duck, Ovary, RNA sequencing, miRNAs, Egg laying

## Abstract

**Background:**

MicroRNAs (miRNAs) exist widely and are involved in multiple biological processes in ducks, whereas the regulatory mechanism of miRNAs in egg laying of ducks has remained unclear. This study aims to reveal key miRNAs involved in the regulation of egg production in duck ovaries.

**Methods:**

High-throughput sequencing was performed on four egg-type duck ovaries and four egg-meat-type duck ovaries at the start of the egg-laying stage. Quantitative reverse transcription PCR (qRT-PCR) validation was performed on differentially expressed miRNAs (DE miRNAs). Gene network of DEmiRNA-mRNA-pathway was constructed by Cytoscape.

**Results:**

A total of 251 know miRNAs and 1,972 novel miRNAs were obtained from whole clean reads. Among the known miRNAs, we identified 21 DEmiRNAs, including eight down-regulated and 13 up-regulated miRNAs in egg-type ducks compared with egg-meat-type ducks. Among the novel miRNAs, we identified 70 DEmiRNAs, including 58 down-regulated and 12 up-regulated in egg-type ducks compared with egg-meat-type ducks. The expression patterns of four miRNAs were verified by qRT-PCR. The DEmiRNAs were involved in the function of response to folic acid and the pathway of valine, leucine and isoleucine degradation. Specific target genes of DEmiRNAs enrichment was found in some egg-laying regulation pathways, such as dopaminergic synapse, ovarian steroidogenesis and oocyte meiosis. The DEmiRNA-mRNA-pathway network including three DEmiRNAs, nine mRNAs and 11 pathways. apl-miR-194-5p and apl-miR-215-5p may be potential key miRNAs in regulating egg laying.

**Conclusions:**

This study provided miRNAs profiles in ducks about egg laying and establish a theoretical basis for subsequent selection or modification of duck phenotypes at the molecular level.

## Introduction

Global demand for duck eggs and meat is growing every year, and China is the main producer (Zeng et al., 2016). However, the variety of poultry on the market and the changing tastes of consumers have made the competition fierce in the poultry farming market. Therefore, scholars have been studying how to improve the yield and quality of duck eggs and meat, and adapt to market competition while improving efficiency. Moreover, ovarian and breast muscle function in poultry exerts a direct impact on egg production and meat quality, respectively (Dong et al., 2010; Liu et al., 2012). Thus, poultry breeding scientists usually focus on the ovary or breast muscle to study egg production and meat quality.

MicroRNAs (miRNAs) are short non-coding RNA molecules with approximately 22 nucleotides. miRNAs involved in the regulation of many biological processes through binding to complementary mRNAs sequences to promote mRNA degradation or translational inhibition (Afonso-Grunz & Muller, 2015). High-throughput sequencing of miRNA on embryonic breast muscle of Pekin duck found that the two novel miRNAs (novel-mir-8 and novel-mir-14) and their targets (MAP2K1 and PPARa) may be a potential inhibitor or promoter for embryonic breast muscle development in Duck (Gu et al., 2014). Wu et al. (2017) identified 17 significantly differentially expressed miRNAs, with 8 up-regulated and 9 down-regulated miRNAs in ovaries of high rates of egg production compared to low rates of egg production. The previous study found a significantly up-regulated miRNA of gga-miR-200a-3p in chickens, and gga-miR-200a-3p is ubiquitous in reproduction regulation-related pathways, which may play a special central role in the reproductive management of chicken (Wu et al., 2017). Therefore, miRNAs indeed regulate egg production of ovarian tissue in egg-type ducks and breast muscle formation of meat-type ducks. However, the mechanism of miRNAs participate in duck egg production regulation remains unclear.

The Jin Ding ducks (JD) and Jian Chang ducks (GF) are both of https://www.ncbi.nlm.nih.gov/genome/2793 mallard. JD ducks are egg-type ducks, which lay eggs but grow slowly and have a small body (Qin, Linquan & Fantong, 1996). GF ducks belong to egg-meat-type ducks, which grow fast and have a large body, but also lay eggs (Zhan et al., 2009). In comparison, the egg-laying performance of GF ducks (including the regularity of the first egg, egg production and egg quality) is much lower than that of JD ducks. Thus, the JD ducks and GF ducks were selected as high-throughput sequencing experimental samples. In this paper, we attempted to reveal the differential expression of miRNAs in the ovaries of JD ducks and laying a foundation for future studies on the molecular mechanism of miRNA in egg production. We extracted total RNA from the ovarian tissues of JD ducks and GF ducks with four repetitions in the early laying period and constructed eight libraries for sequencing. This study provides a theoretical foundation at the molecular level for future studies that selection or modification of duck phenotypes.

## Material and Methods

### Ethics statement

All sample’s collection and experiment on the ducks were completely adhered to guidelines and approved by the Animal Care and Use Committee of the Sichuan Animal Science Academy. No associated permit number was required since commercial animal sampling was approved. All efforts were made to minimize ducks suffering.

### Samples

There were four Jin Ding ducks (JD) that represent egg-type ducks, and four Jian Chang (GF) ducks that represent egg-meat-type ducks, all of them were from Sichuan Academy of Animal Science. All ducks were raised together in the environment with free access to feed, water and commercial diets. The eight samples of ovarian tissue from duck were collected when start laying egg, and immediately stored in liquid nitrogen.

### Total RNA extraction, cDNA library construction and sequencing

The total RNA from ovarian tissue was isolated using TRIzol (Invitrogen; Thermo Fisher Scientific, Inc.) with four repetitions dissolved in diethyl pyrocarbonate water. NanoDrop spectrophotometer (Thermo Fisher Scientific, Inc.) was used to detect the concentration and retain RNA tubes with concentrations above 20 µg. The small RNA library was constructed using Small RNAS ample Preparation Kit (Illumina, San Diego, USA), and the RNA fraction of 135–150 nt was excised by polyacrylamide gel electrophoresis (PAGE) and ligated with proprietary adaptors. The cDNA was amplified by reverse transcription-PCR (RT-PCR) and then quality tested on Agilent 2100 Bioanalyzer. The high-quality cDNA library was sequenced on the Illumina Hiseq™ 2500 platform.

### Sequence identified and expression analysis

Clean reads were obtained after eliminating contaminant reads as described by the process from raws reads (Ji et al., 2012), and then highly similar sequences of rRNA, tRNA, and sncRNA were eliminated. Aligned the clean reads with the genome, miRBase and Rfam of https://www.ncbi.nlm.nih.gov/genome/2793 (mallard) based on internationally recognized BWA algorithm to identified miRNA sequences. The miRNA sequence was mapped to human (Venter et al., 2001), Gallus gallus (Li et al., 2015) and Zebra finch (Ekblom et al., 2010) using miRDeep2 (Friedlander et al., 2012) for prediction of novel miRNAs and the unannotated sequences were defined as predicted novel miRNAs. Moreover, the miRNA sequences were counted to obtain the expression. Results of the expression were used for comparing DEmiRNAs between JD ducks with GF ducks, two base mismatches were allowed since the miRNA diversities in different species. DEmiRNAs were calculated based on DEseq2 method and the miRNAs were considered differentially expressed when Log2FC >1 or <−1 and FDR <0.05.

### miRNA target prediction and functional annotation

The miRanda (Hammond et al., 2016) and RNAhybrid (Gantz et al., 2015) were used to analyze the putative targets and predicted target genes. The detailed parameter setting as described by Liu et al., (2014). The targets of DEmiRNAs were aligned in the Gene Ontology (GO) database to obtain the GO annotation and classification. The GO term with a threshold of FDR <0.05 to identify the significant enrichment. The targets of DEmiRNAs were also aligned to the Kyoto encyclopedia of genes and genomes (KEGG) database for pathways enrichment analysis. The *P* value was cut-off 0.05 and the lower of it the more significant the metabolism pathway.

### Quantitative reverse transcription PCR (qRT-PCR) verification

We selected 4 miRNAs to verify the reliability of sequencing data. The chosen DEmiRNAs were broadly conserved and related to egg production. The primers for qRT-PCR were synthesized by Sangon Biotech (Shanghai, China) and listed in Table 1. TRIzol (TaKaRa, Dalian, China) was used to isolate total RNA. The concentration and purity were detected by microspectrophotometer (Tiangen Biotech Co., Ltd.). qRT-PCR was carried out with FastStart Universal SYBR Green Master Mix (TaKaRa, Dalian, China) and QuantStudio 6 Flex Real-Time PCR System (Thermo Fisher Scientific, Inc.). All reactions were performed in triplicate and the 2^−ΔΔ*Cq*^ method was used to determine the relative gene expression.

**Table 1:** The primers used for qRT-PCR.

Name	Application	Sequence
apl-mir-194-5pRT	RT-PCR	5′-GTCGTATCCAGTGCGTGTCGTGGAGTCG GCAATTGCACTGGATACGACGTCCACA-3′
apl-mir-215-5pRT	RT-PCR	5′-GTCGTATCCAGTGCGTGTCGTGGAGTC GGCAATTGCACTGGATACGACAGTCTGT-3′
apl-mir-460b-5pRT	RT-PCR	5′-GTCGTATCCAGTGCGTGTCGTGGAGTC GGCAATTGCACTGGATACGACCACACAG-3′
apl-mir-206RT	RT-PCR	5′-GTCGTATCCAGTGCGTGTCGTGGAGTC GGCAATTGCACTGGATACGACACACACT-3′
U6-F	qRT-PCR	5′-AGAAAATTAGCATGGCCCCTG-3′
U6-R	qRT-PCR	5′-AACGCTTCACGAATTTGCGT-3′
apl-mir-194-5pF	qRT-PCR	5′-GCGGGTGTAACAGCAACTCC-3′
apl-mir-215-5pF	qRT-PCR	5′-GGCGGGATGACCTATGAATTG-3′
apl-mir-460b-5pF	qRT-PCR	5′-GGCGGGTCCTCATTGTACAT-3′
apl-mir-206F	qRT-PCR	5′-GGCGGGTGGAATGTAAGGA-3′
all-R	qRT-PCR	5′-AGTGCGTGTCGTGGAGTCG-3′

**Notes.**

All-R indicate that the four candidate miRNAs share the same REVERSE primer when did qRT-PCR.

### Construction of the gene network

According to the analysis of DEmiRNAs and predicted target gene, the network of DEmiRNA-mRNA between all the DEmiRNAs and their predicted target genes were constructed. Moreover, according to the KEGG pathway that associated with egg production and meat production the network of DEmiRNA-mRNA was also constructed. Based on the results of qRT-PCR, the network of DEmiRNA-mRNA-pathway was constructed. All the network was visualized by version 3.6.1 of Cytoscape (https://www.softpedia.com/get/Science-CAD/Cytoscape.shtml).

### Statistical analysis

Data were expressed as the mean ± standard deviation (SD). Statistical analysis performed by SPSS 16 with a *t*-test. The value of *P* < 0.05 was defined as statistical significance.

## Results

### Overview of miRNA sequencing

The quality control results of the ovarian sequencing data from the ducks of each group were summarized in Table 2. Data range from 7.23–13.63 million clean reads were obtained from eight ovarian tissues after filtering the low-quality sequences, and the filter percentages in all groups were higher than 0.88%. The GC value of percentage above 44%. The percentage of reads mapped to Genome and Rfam was above 90%, and the percentage of reads mapped to miRBase was between 7% and 13%. The length distribution of reads that were compared to miRBase database showed that the priority aggregation lengths of sequences were 21, 22 and 23 nt, which were consistent with the characteristics of miRNA (Fig. S1).

**Table 2:** Raw data quality control and statistics.

Samples	Clead reads	Filter (%)	GC (%)	Mapped rate (%)		
				miRBase	Genome	Rfam
GF1	10,733,087	0.9176	45	7.8	97.7	90.2
GF2	11,438,047	0.8921	44	11.4	93.4	98.8
GF3	11,046,361	0.8808	44	10.4	96.2	98.9
GF4	10,485,850	0.8812	45	10.8	92.5	99
JD1	7,942,134	0.8862	45	11.1	96.8	99.1
JD2	7,232,918	0.8901	45	12.5	98	99.3
JD3	13,625,866	0.8834	45	8.9	97.1	99.1
JD4	11,403,634	0.9110	46	7.7	98.1	99.1

### Expression of known and novel miRNAs

In this study, 251 known miRNAs were identified in eight ovary samples. The miRNA expression profile revealed that expression varies greatly between different miRNAs, and the expression levels of several miRNAs accounted for more than half of the total expression levels of all miRNAs (Table S1). Among them, apl-mir-99-5pdisplayed 180,615 reads with the highest expression level in GF2 group of egg-meat-type ducks, followed by apl-let-7f-5p and apl-mir-145-5p in JD3 group of egg-type ducks. Moreover, only 82 (32.67%) miRNAs expressions exceeded 100 reads in eight ovary samples. Besides, a total of 1972 novel miRNAs were predicted. The expression level of kb744307.1_22722_mature with above 32,000 reads was much higher than others (Table S1). Most miRNAs were even less than 5,000 reads, and only 72 (3.65%) novel miRNAs expressions level exceeded 100 reads in eight ovary samples. Thus, the sequencing frequencies of novel miRNAs were much lower compared to those of known miRNAs.

### Identification of DEmiRNAs

**Figure 1:**
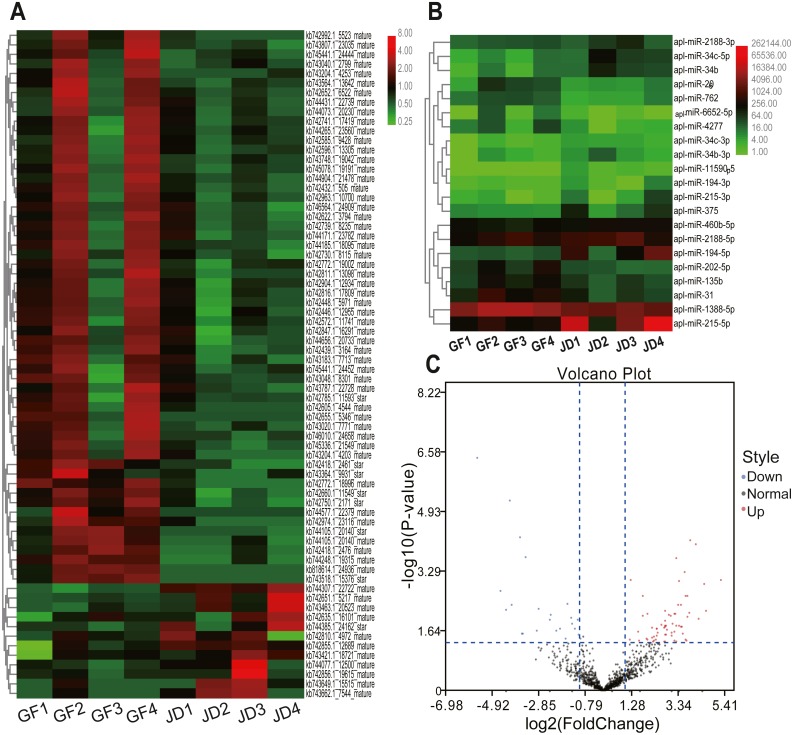
Cluster heatmaps of novel (A) and known (B) DEmiRNAs between egg-type JD ducks and egg-meat-type of GF ducks. Each column represents a sample, the row shaded in red represent up-regulated, and shaded in green represent down-regulated. (C) Volcano plot of all DEmiRNAs between egg-type JD ducks and egg-meat-type of GF ducks.

In the present study, we identified 21 DEmiRNAs in know miRNAs between egg-meat-type ducks and egg-type ducks, including 8 down-regulated and 13 up-regulated in egg- type ducks compared with egg-meat-type ducks, as shown in the Volcano plot (Figs. 1B and 1C). Moreover, 70 DEmiRNAs in novel miRNAs including 58 down-regulated and 12 up-regulated in egg-type ducks compared with egg-meat-type ducks (Figs. 1A and 1C). There was a variability between the 4 replicas within each group, which may be due to differences among individuals, but the DEmiRNAs were selected based on the adjusted *P* value.

### Validation of miRNAs

**Figure 2:**
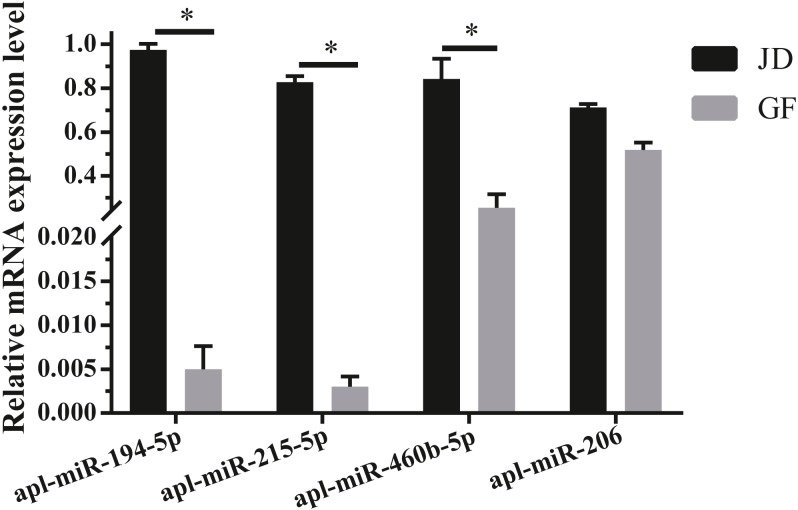
Validation of 4 selected DEmiRNAs. Gene expression was normalized to U6 transcript levels. Data was analyzed by *t*-test. * indicates the significant difference of *P* < 0.05.

To validate the reliability of sequencing data, 4 DEmiRNAs with high expression and large fold differences, including apl-miR-194-5p, apl-miR-215-5p, apl-miR-460b-5p, and apl-miR-206 were selected to examine by qRT-PCR. Among them, the first three miRNAs were up-regulated in JD, and only apl-miR-206 was down-regulated in JD in our sequencing data (Fig. 1B). In the results of qRT-PCR (Fig. 2), compared with egg-meat-type of GF ducks, the expression levels of 4 miRNAs were up-regulated in egg-type of JD ducks. Especially, the difference of apl-miR-194-5p, apl-miR-215-5p and apl-miR-460b-5p was significant and the difference of apl-miR-206 was not significant between egg-type of JD ducks and egg-meat-type of GF ducks. Thus, except apl-miR-206, the expression pattern of other miRNAs was consistent with Illumina sequencing data.

### Target gene prediction of DEmiRNAs

**Figure 3:**
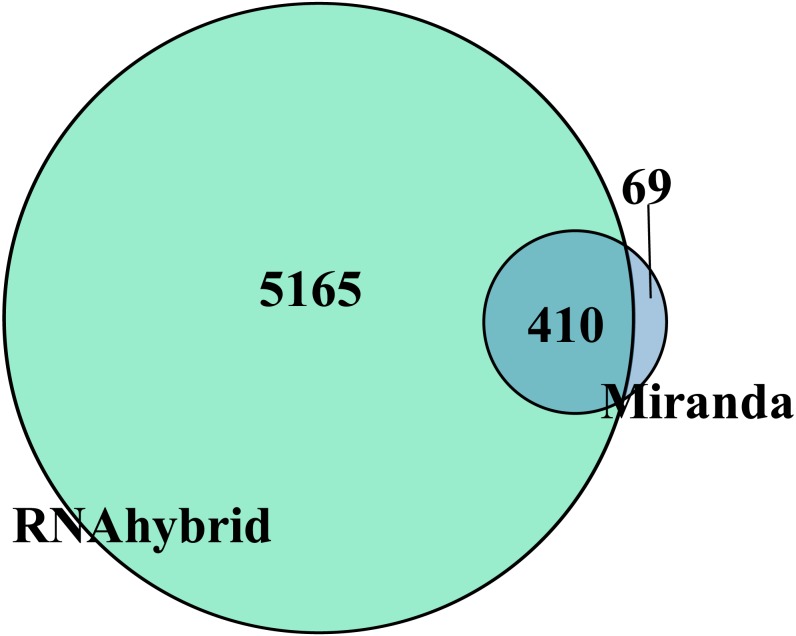
Venn diagram of DEmiRNAs target gene predicted by Miranda and RNAhybrid algorithm. Taking the intersection of the two algorithms as the final target gene, herein obtained 410 target genes.

Target gene prediction is an important way to understand RNA function. In total, the DEmiRNAs were predicted to target 410 genes by RNAhybrid and Miranda (Fig. 3). Only apl-miR-202-5p has no target gene. We constructed the DEmiRNA-mRNA co-expression network between the 20 DEmiRNAs with 410 target mRNAs, shown in Fig. S2. Obviously, a miRNA predicted more than one target genes, such as apl-miR-194-5p and apl-miR-215-5p predicted 15 and 3 targets, respectively. Similarly, a target was might be targeted by multiple miRNAs at multiple targeting sites, such as telomere maintenance 2 (TELO2) can be targeted by three miRNAs (apl-miR-34b, apl-miR-34c-5p and apl-miR-762), and many target genes were shared between apl-miR-34b and apl-miR-34c-5p (Fig. S2).

### GO functional and KEGG pathway analysis of DEmiRNAs

**Figure 4:**
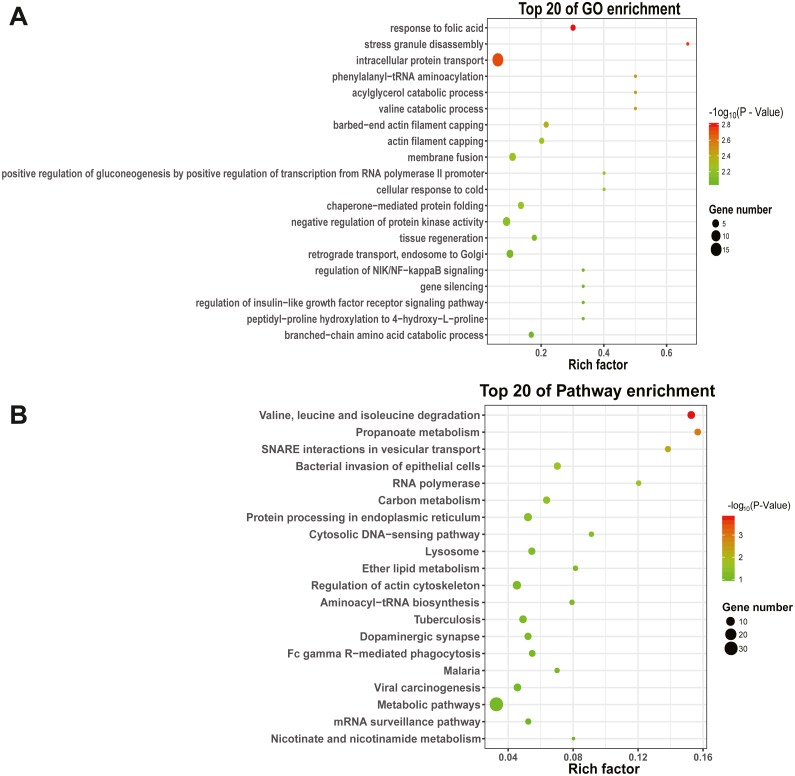
GO and KEGG pathways enrichment based on differentially expressed know miRNA target genes. (A) The top 20 GO enrichment; (B) The top 20 KEGG enrichment. The left represents the GO term or KEGG pathway, the right represents enrichment, and the size of solid circle indicates the number of genes.

To explore the function and metabolic pathway of the DEmiRNAs, all of the predicted target genes were mapped to GO and KEGG databases (Fig. 4). The results suggested that target genes of DEmiRNAs were priority clustered into intracellular protein transport followed by negative regulation of protein kinase activity, membrane fusion and retrograde transport, endosome to golgi. However, the most significantly GO term was response to folic acid, followed by stress granule disassembly and intracellular protein transport successively. Results of the KEGG pathway analysis showed that the DEmiRNAs significantly enriched in valine, leucine and isoleucine degradation, followed by propanoate metabolism and N-ethylmaleimide-sensitive factor attachment protein receptor (SNARE) interactions in vesicular transport. In addition, specific gene enrichment was found in some laying egg and meat production regulation pathways, such as dopaminergic synapse, oocyte meiosis, ovarian steroidogenesis, oxytocin signaling pathway, progesterone-mediated oocyte maturation, insulin signaling pathway and valine, leucine and isoleucine degradation.

### miRNA-mRNA-pathway network

**Figure 5:**
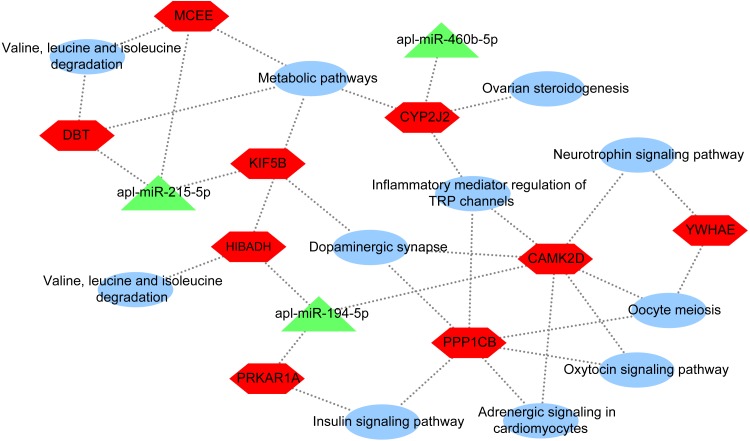
DEmiRNA-mRNA-pathway network. The network consists of three DEmiRNAs, nine mRNA, and 11 pathways. Triangle indicate DEmiRNA, hexagon indicate mRNA, and ellipse indicate pathway.

To further explore the underlying regulatory mechanisms of miRNAs involved in egg production in duck, a DEmiRNA-mRNA-pathway network was constructed (Fig. 5). The DEmiRNA-mRNA-pathway network including 3 significantly DEmiRNAs verified by qRT-PCR, and 9 mRNAs involved in 11 pathways. Given that the expression patterns of apl-miR-206 in sequencing data and qRT-PCR results were inconsistent (Fig. 2), it was not considered when constructing the network. The apl-miR-194-5p was predictively targeted to three target genes, including protein kinase cAMP-dependent regulatory type I alpha (PRKAR1A), 3-hydroxyisobutyrate dehydrogenase (HIBADH) and calcium/calmodulin-dependent protein kinase type II delta chain-like (CAMK2D). PRKAR1A and HIBADH involved insulin signaling pathway and valine, leucine and isoleucine degradation pathway, respectively. Moreover, CAMK2D simultaneously involved in multiple pathways associated with egg production, such as oxytocin signaling pathway, dopaminergic synapse and oocyte meiosis. The apl-miR-215-5p was predicted target gene of kinesin family member 5B (KIF5B), methylmalonyl CoA epimerase (MCEE) and dihydrolipoamide branched chain transacylase E2 (DBT). All three of them were enriched in the same pathway, such as metabolic pathways, whereas MCEE and DBT also involved in valine, leucine and isoleucine degradation pathway, and KIF5B involved in dopaminergic synapse. The cytochrome P450 2J2-like (CYP2J2) was predicted target gene ofapl-miR-460b-5p that involved in metabolic pathways, inflammatory mediator regulation of TRP channels, dopaminergic synapse, and ovarian steroidogenesis.

## Discussions

High-throughput sequencing technology can be used to mine candidates genes associated with a particular background and predict the function of unknown genes (Chen et al., 2014a). In this study, the DEmiRNAs were significantly enriched in pathway of valine, leucine and isoleucine degradation, and DEmiRNAs were significantly-enriched in oocyte meiosis, progesterone-mediated oocyte maturation, insulin signaling pathway, and dopaminergic synapse. According to reports, the above pathways were involved in cell proliferation, development, nutrition and nervous system development and function, respectively (Bayaa et al., 2000; Shi et al., 2018; Uchigashima et al., 2016). These pieces of evidence indicated that the DEmiRNAs regulates egg production through the above pathways in duck.

In this paper, apl-miR-215-5p targeted KIF5B, MCEE, and DBT. KIF5B plays an important role in the early stage of meiosis in female mice (Kidane et al., 2013). Thus, KIF5B may be involved in egg production regulation by affecting meiosis. In addition, DBT and MCEE were involved in valine, leucine and isoleucine degradation (Fig. 5). The study about the interaction of isoleucine, leucine, valine and tryptophan in laying hens by using the three-factor test, which found that leucine and valine would affect the uptake of isoleucine in eggs and the quality of eggs, while tryptophan had no any effect (Peganova & Eder, 2003; Tuttle & Balloun, 1976). In addition, valine may be the first restricted total branched-chain amino acid in egg protein (Riazi et al., 2003). In short, we found that valine, leucine and isoleucine were essential amino acids for laying hens. Thus, DBT and MCEE may be involved in regulating egg reproduction in ducks. In conclusion, apl-miR-215-5p may regulate laying egg, and these regulatory effects were not the result of a single targeted gene, whereas the coordination of KIF5B, MCEE, and DBT.

Moreover, the expression level of apl-miR-194-5p in ovaries from egg-type ducks and egg-meat-type ducks showed a significant difference and apl-miR-194-5p simultaneously targeted CAMK2D, PRKAR1A and HIBADH. As shown in Fig. 5, PRKAR1A and HIBADH were annotated to pathways related to meat metabolism, including insulin signaling pathway and valine, leucine and isoleucine degradation, CAMK2D involved in the reproductive regulation pathway, oxytocin signaling pathway, dopaminergic synapse, and oocyte meiosis. Oxytocin is a neuropeptide involved in animal reproductive to modulate complex behaviors, and oxytocin signaling pathway was not only closely related to reproductive also indirectly affects other metabolism of animals (Quintana et al., 2019). Therefore, apl-miR-194-5p may regulate a complex set of physiological activities by controlling the release of oxytocin. Hence, the complex physiological activity of whether the duck is the long meat or the egg production may be regulated via the apl-miR-194-5p by targeted CAMK2D, PRKAR1A, and HIBADH.

Remarkably, except to apl-miR-194-5p and apl-miR-215-5p which discussed above, and also except to apl-miR-11590-5p and apl-miR-6652-5p which did not find any reports, the remaining 17 DEmiRNAs (Fig. 1) were all appeared in the differential expression profile list relation to the reproduction of other species. The miR-34b and miR-34c both had a significant increase in the high rate of egg production chickens ovary compared with the low rate of egg production chickens ovary (Wu et al., 2017). The miR-202 was only expressed in testis and vitellogenic ovary compared with other tissues in teleosts, and by comparing in five stage of oogenesis, the miR-202 was high expressed in late vitellogenesis (Juanchich et al., 2013). The miR-31 suppressed enrichment of female miRNAs bantam and results in a morphological alternation of ovaries in female schistosomes (Zhu et al., 2016). The miR-1388 was abundant and ovary-enriched in zebrafish by dynamics miRNA transcriptome sequencing (Presslauer et al., 2017). The miR-460-5p exhibited significantly low levels in the egg-laying group compared with broody (Chen et al., 2014b). The presence of primary miR-375 made zygotic genome activation occurs earlier in honeybees than in *Drosophila* during the cleavage stage in haploid and diploid embryonic development (Pires et al., 2016). The miR-4277 was differentially expressed in the Down syndrome placenta (Lim et al., 2015). The miR-762 can negatively regulate menin in ovarian cancer (Hou et al., 2017). The miR-135, miR-2188 and miR-206 were conserved expressed in chicken follicle (Zhang, Li & Du, 2017). In a word, we speculate that these DEmiRNAs may be related to the reproductive system of ducks and were most likely directly involved in the regulation of egg production. Regrettably, we have not been able to further verify the role and mechanism of the above miRNAs in egg production.

## Conclusions

In summary, we obtained the miRNA profiles of ovarian tissues in egg-type and egg-meat-type ducks using sequencing. The target genes of DEmiRNAs were enriched in response to folic acid, and involved in some reproduction regulation pathways. All the DEmiRNAs may be involved in duck egg-laying regulation. The DEmiRNA-mRNA-pathway network revealed that the interaction network of apl-miR-194-5p and apl-miR-215-5p is the key factors in determining whether ducks develop into egg- or meat- producing breeds. This study provided-several key regulators in duck related to egg production and lays a theoretical basis for subsequent selection or modification of duck phenotypes at the molecular level.

##  Supplemental Information

10.7717/peerj.8440/supp-1 Table S1 Length distribution of sequence reads after quality-trimming and adaptor removal The counts of all miRNAs including known and novel in eight ovary samples.

10.7717/peerj.8440/supp-2 Figure S1 Length distribution of sequence reads after quality-trimming and adaptor removal Note: GF4 represent the ovary libraries from fourth group of Jian Chang ducks.

10.7717/peerj.8440/supp-3 Figure S2 The DEmiRNA-mRNA network. The network consists of 20 known DEmiRNAs and 410 target genes Orange indicate DEmiRNA, blue indicate target genes. Blue oval represents the target gene, red diamond represents the up-regulated DEmiRNAs in JD, and green represents the down-regulated DEmiRNAs in JD.

## Additional Information and Declarations

### DNA Deposition

The following information was supplied regarding the deposition of DNA sequences: The RNA sequences are available at NCBI BioSamples: SAMN13021552– SAMN13021559.

### Data Availability

The following information was supplied regarding data availability: The data is available at NCBI: PRJNA577194.
