# Supplementary material for: High-throughput sequencing analysis identified microRNAs associated with egg production in ducks ovaries
Source: PeerJ. 2020 Feb 4;8:e8440. doi: 10.7717/peerj.8440 (PMC7006514; doi:10.7717/peerj.8440)
Supplement: Table S1 — The counts of all miRNAs including known and novel in eight ovary samples. [file peerj-08-8440-s001.docx]

**Supplemental Table S1: The counts of all known miRNAs in eight ovary samples.**

| **miRNA_id** | **blast_id** | **seq** | **GF1** | **GF2** | **GF3** | **GF4** | **JD1** | **JD2** | **JD3** | **JD4** |
| --- | --- | --- | --- | --- | --- | --- | --- | --- | --- | --- |
| apl-let-7a-1-3p | apl-let-7a-1-3p@@tgu-let-7a-1-3p | ctatacaatctactgtctttcc | 159 | 85 | 86 | 161 | 36 | 51 | 152 | 58 |
| apl-let-7a-2-3p | apl-let-7a-2-3p@@tgu-let-7a-2-3p | ctgtacaacctcctagctttcc | 7 | 7 | 9 | 9 | 10 | 5 | 10 | 6 |
| apl-let-7a-4-3p | apl-let-7a-4-3p@@tgu-let-7a-4-3p | ctatacagtctattgccttcct | 138 | 141 | 158 | 191 | 68 | 112 | 203 | 131 |
| apl-let-7a-5p | apl-let-7a-5p@@tgu-let-7a-5p | tgaggtagtaggttgtatagtt | 43890 | 59500 | 43998 | 44695 | 30027 | 39880 | 60828 | 33193 |
| apl-let-7b-5p | apl-let-7b-5p@@tgu-let-7b-5p | tgaggtggtaggttgtgt | 18 | 26 | 24 | 25 | 13 | 30 | 18 | 20 |
| apl-let-7c-3p | apl-let-7c-3p@@tgu-let-7c-3p | ctgtacaaccttctagctttcc | 1 | 7 | 1 | 5 | 1 | 0 | 4 | 2 |
| apl-let-7c-5p | apl-let-7c-5p@@tgu-let-7c-5p | tgaggtagtaggttgtatggtt | 22484 | 48134 | 44316 | 33042 | 23125 | 19777 | 36663 | 21197 |
| apl-let-7d-3p | apl-let-7d-3p@@tgu-let-7d-3p | ctatacaacctgctgcctttct | 20 | 32 | 34 | 29 | 19 | 21 | 44 | 25 |
| apl-let-7d-5p | apl-let-7d-5p@@tgu-let-7d-5p | agaggtagtaggttgcatagtt | 111 | 156 | 160 | 121 | 113 | 124 | 202 | 114 |
| apl-let-7e-5p | apl-let-7e-5p@@tgu-let-7e-5p | tgaggtagtagattgaatagtt | 13468 | 17203 | 12862 | 12733 | 8987 | 12203 | 19090 | 9541 |
| apl-let-7f-3p | apl-let-7f-3p@@tgu-let-7f-3p | ctatacaatctattgccttccc | 42 | 50 | 49 | 55 | 19 | 26 | 58 | 29 |
| apl-let-7f-5p | apl-let-7f-5p@@tgu-let-7f-5p | tgaggtagtagattgtatagtt | 84888 | 126052 | 121418 | 92712 | 64155 | 83001 | 140363 | 79268 |
| apl-let-7g-3p | apl-let-7g-3p@@tgu-let-7g-3p | ctgtacaggccactgccttgcc | 3 | 12 | 18 | 14 | 4 | 7 | 11 | 7 |
| apl-let-7g-5p | apl-let-7g-5p@@tgu-let-7g-5p | tgaggtagtagtttgtacagtt | 43707 | 74089 | 64085 | 52511 | 44309 | 51496 | 72984 | 41587 |
| apl-let-7i-3p | apl-let-7i-3p@@tgu-let-7i-3p | ctgcgcaagctactgccttgct | 7 | 12 | 10 | 6 | 3 | 5 | 9 | 5 |
| apl-let-7i-5p | apl-let-7i-5p@@tgu-let-7i-5p | tgaggtagtagtttgtgctgtt | 67432 | 129899 | 134946 | 62570 | 46119 | 53668 | 122796 | 52984 |
| apl-mir-100-3p | apl-mir-100-3p@@hsa-mir-100-3p | acaagcttgtatctataggtatg | 1 | 2 | 1 | 1 | 1 | 0 | 1 | 1 |
| apl-mir-100-5p | apl-mir-100-5p@@gga-mir-100-5p | aacccgtagatccgaacttgtgg | 25753 | 70475 | 39368 | 72746 | 48784 | 27015 | 45836 | 24859 |
| apl-mir-10585-5p | apl-mir-10585-5p@@gga-mir-10585-5p | tgatgaaatcaccctatggcctgt | 7 | 5 | 7 | 3 | 0 | 1 | 7 | 1 |
| apl-mir-106-3p | apl-mir-106-3p@@gga-mir-106-3p | actgcagtataagcacttctgg | 5 | 10 | 6 | 10 | 12 | 6 | 11 | 1 |
| apl-mir-106-5p | apl-mir-106-5p@@tgu-mir-106-5p | aaaagtgcttacagtgcaggta | 207 | 757 | 490 | 475 | 236 | 150 | 461 | 186 |
| apl-mir-107 | apl-mir-107@@tgu-mir-107 | agcagcattgtacagggctat | 195 | 320 | 370 | 371 | 197 | 238 | 250 | 293 |
| apl-mir-10a-5p | apl-mir-10a-5p@@tgu-mir-10a-5p | taccctgtagaaccgaatttgt | 419 | 419 | 293 | 368 | 244 | 375 | 429 | 280 |
| apl-mir-10b-3p | apl-mir-10b-3p@@gga-mir-10b-3p | agattcgattctaggggaata | 12 | 3 | 2 | 7 | 0 | 6 | 5 | 4 |
| apl-mir-10c-3p | apl-mir-10c-3p@@gga-mir-10c-3p | aaattcgtctctaggggaata | 26 | 32 | 25 | 36 | 31 | 28 | 35 | 25 |
| apl-mir-10c-5p | apl-mir-10c-5p@@gga-mir-10c-5p | taccctgtaggctcgaatttgt | 627 | 895 | 895 | 1309 | 679 | 963 | 662 | 851 |
| apl-mir-11588-3 | apl-mir-11588-3 | tcctggattcgctgctgctgttt | 4 | 2 | 0 | 1 | 1 | 1 | 10 | 0 |
| apl-mir-11589-3 | apl-mir-11589-3 | ttgcagttttgcatacatgctt | 0 | 0 | 0 | 1 | 0 | 1 | 2 | 0 |
| apl-mir-11589-5 | apl-mir-11589-5 | gaatgtctgcagacagaggc | 0 | 1 | 3 | 2 | 0 | 0 | 3 | 1 |
| apl-mir-11590-3 | apl-mir-11590-3 | aggactggagataaaatgttcc | 0 | 4 | 3 | 1 | 1 | 1 | 1 | 0 |
| apl-mir-11590-5 | apl-mir-11590-5 | atgtattacatggacgtgc | 0 | 0 | 0 | 0 | 6 | 1 | 2 | 1 |
| apl-mir-11591-3 | apl-mir-11591-3 | tggacgaggagctcttct | 2 | 14 | 10 | 7 | 8 | 3 | 7 | 2 |
| apl-mir-11591-5 | apl-mir-11591-5 | gctctgtaagtgtacctg | 1 | 3 | 1 | 0 | 1 | 0 | 1 | 0 |
| apl-mir-12214-3p | apl-mir-12214-3p@@gga-mir-12214-3p | aaggggaagcactgacggcca | 0 | 0 | 3 | 1 | 0 | 0 | 0 | 0 |
| apl-mir-12223-3p | apl-mir-12223-3p@@gga-mir-12223-3p | cctcccagcctctcttct | 4 | 0 | 2 | 5 | 3 | 1 | 0 | 2 |
| apl-mir-12239-5p | apl-mir-12239-5p@@gga-mir-12239-5p | ttatgactgctggaaggagc | 0 | 4 | 5 | 3 | 2 | 1 | 2 | 0 |
| apl-mir-122-3p | apl-mir-122-3p@@gga-mir-122-3p | acgccattatcacactaaata | 0 | 0 | 0 | 0 | 0 | 0 | 1 | 0 |
| apl-mir-12253-3p | apl-mir-12253-3p@@gga-mir-12253-3p | tcctctgcccctcgccttgcc | 0 | 2 | 0 | 0 | 1 | 0 | 0 | 0 |
| apl-mir-12253-5p | apl-mir-12253-5p@@gga-mir-12253-5p | ttgcgggagctgagaagcc | 2 | 7 | 5 | 4 | 1 | 1 | 1 | 3 |
| apl-mir-122-5p | apl-mir-122-5p@@tgu-mir-122-5p | tggagtgtgacaatggtgttt | 509 | 296 | 2633 | 361 | 1153 | 218 | 331 | 264 |
| apl-mir-12268-5p | apl-mir-12268-5p@@gga-mir-12268-5p | cggggcgggcgggcggca | 49 | 60 | 118 | 66 | 32 | 87 | 36 | 107 |
| apl-mir-12284-3p | apl-mir-12284-3p@@gga-mir-12284-3p | ccgtgctcgtgtccctctgtc | 0 | 2 | 0 | 0 | 1 | 3 | 2 | 0 |
| apl-mir-125-1-3p | apl-mir-125-1-3p@@tgu-mir-125-1-3p | acaagtcaggctcttgggacc | 1453 | 3794 | 2630 | 3415 | 2202 | 2000 | 2333 | 1512 |
| apl-mir-125-2-3p | apl-mir-125-2-3p@@tgu-mir-125-2-3p | acgggttaggctcttgggagc | 320 | 395 | 330 | 284 | 199 | 190 | 399 | 288 |
| apl-mir-125-5p | apl-mir-125-5p@@tgu-mir-125-5p | tccctgagaccctaacttgtga | 8570 | 11962 | 9955 | 16056 | 10196 | 12955 | 9062 | 6442 |
| apl-mir-129-3p | apl-mir-129-3p@@tgu-mir-129-3p | aagcccttaccccaaaaagca | 3 | 9 | 12 | 3 | 12 | 11 | 5 | 8 |
| apl-mir-129-5p | apl-mir-129-5p@@tgu-mir-129-5p | ctttttgcggtctgggcttgc | 647 | 1514 | 1174 | 626 | 592 | 341 | 1072 | 477 |
| apl-mir-1329-5p | apl-mir-1329-5p@@tgu-mir-1329-5p | tacagtgatcaggttacgatgg | 7 | 16 | 44 | 26 | 15 | 15 | 13 | 5 |
| apl-mir-135a-2-3p | apl-mir-135a-2-3p@@gga-mir-135a-2-3p | tgtagggatggaagccatgaa | 7 | 87 | 21 | 39 | 17 | 4 | 25 | 3 |
| apl-mir-135a-3-3p | apl-mir-135a-3-3p@@gga-mir-135a-3-3p | tgtagggcgaaaagccatggg | 0 | 1 | 0 | 0 | 0 | 0 | 0 | 0 |
| apl-mir-135b | apl-mir-135b@@tgu-mir-135b | tatggctttttattcctatgtg | 58 | 396 | 145 | 337 | 91 | 30 | 90 | 45 |
| apl-mir-138-1-3p | apl-mir-138-1-3p@@tgu-mir-138-1-3p | ggctacttcacaacaccagggtg | 12 | 10 | 16 | 7 | 2 | 21 | 11 | 8 |
| apl-mir-138-5p | apl-mir-138-5p@@tgu-mir-138-5p | agctggtgttgtgaatcaggccgt | 10 | 50 | 18 | 29 | 5 | 22 | 24 | 10 |
| apl-mir-1388-5p | apl-mir-1388-5p@@tgu-mir-1388-5p | aggactgtctaacctgagaatggt | 6321 | 17208 | 18630 | 9650 | 3706 | 2772 | 4954 | 2712 |
| apl-mir-1416-3p | apl-mir-1416-3p@@gga-mir-1416-3p | caattgtgtgagttgagtaca | 1 | 3 | 3 | 2 | 2 | 6 | 5 | 4 |
| apl-mir-1416-5p | apl-mir-1416-5p@@gga-mir-1416-5p | tccttaactcatgctgctgc | 14 | 13 | 7 | 55 | 25 | 33 | 17 | 65 |
| apl-mir-142-5p | apl-mir-142-5p@@tgu-mir-142-5p | cataaagtagaaagcaca | 21 | 29 | 18 | 43 | 21 | 38 | 30 | 8 |
| apl-mir-1451-3p | apl-mir-1451-3p@@tgu-mir-1451-3p | agtaacttgctcctgtgagagg | 4 | 14 | 6 | 23 | 4 | 12 | 10 | 3 |
| apl-mir-1451-5p | apl-mir-1451-5p@@gga-mir-1451-5p | tcgcacaggagcaagttaccgc | 66 | 127 | 172 | 127 | 82 | 88 | 101 | 82 |
| apl-mir-145-3p | apl-mir-145-3p@@hsa-mir-145-3p | ggattcctggaaatactgttct | 1113 | 688 | 576 | 1034 | 500 | 1112 | 1407 | 1013 |
| apl-mir-145-5p | apl-mir-145-5p@@gga-mir-145-5p | gtccagttttcccaggaatccct | 112045 | 52334 | 118751 | 58783 | 68204 | 130481 | 136297 | 100311 |
| apl-mir-1465 | apl-mir-1465@@gga-mir-1465 | aggcttgccccagcacctctc | 2 | 7 | 3 | 4 | 1 | 1 | 2 | 2 |
| apl-mir-1466 | apl-mir-1466@@gga-mir-1466 | gaggagctcaggcaggtgctg | 1 | 3 | 4 | 0 | 0 | 0 | 1 | 2 |
| apl-mir-1467-3p | apl-mir-1467-3p@@gga-mir-1467-3p | ttcacaccagagtaactgggagc | 4 | 6 | 4 | 14 | 7 | 11 | 11 | 4 |
| apl-mir-1467-5p | apl-mir-1467-5p@@tgu-mir-1467-5p | tctcagctacgtcggtgtaaatc | 4 | 6 | 6 | 6 | 3 | 5 | 11 | 8 |
| apl-mir-146a-5p | apl-mir-146a-5p@@tgu-mir-146a-5p | agagtactgaattccatggaca | 108 | 184 | 160 | 264 | 132 | 168 | 181 | 142 |
| apl-mir-146b-3p | apl-mir-146b-3p@@tgu-mir-146b-3p | gccctatggattcagttctgcag | 2 | 1 | 5 | 1 | 3 | 0 | 1 | 3 |
| apl-mir-146b-5p | apl-mir-146b-5p@@tgu-mir-146b-5p | tgagaactgaattccataggcgt | 1890 | 3086 | 5884 | 3494 | 1870 | 1273 | 5158 | 1798 |
| apl-mir-146c | apl-mir-146c@@tgu-mir-146c | agaactgaattccatgggttg | 23 | 45 | 46 | 39 | 11 | 15 | 39 | 26 |
| apl-mir-1552-3p | apl-mir-1552-3p@@gga-mir-1552-3p | acgctagctgctctgcactaact | 13 | 37 | 62 | 49 | 25 | 34 | 35 | 19 |
| apl-mir-1552-5p | apl-mir-1552-5p@@gga-mir-1552-5p | ttagtgcgcagtaagctagggtgt | 73 | 147 | 192 | 127 | 81 | 73 | 169 | 92 |
| apl-mir-155-5p | apl-mir-155-5p@@hsa-mir-155-5p | ttaatgctaattgtgataggggtt | 162 | 280 | 370 | 272 | 162 | 93 | 400 | 56 |
| apl-mir-1559 | apl-mir-1559@@tgu-mir-1559 | ttcgatgcttgtatgctactcc | 3045 | 7911 | 11248 | 5051 | 2678 | 2243 | 6869 | 3094 |
| apl-mir-1559-3p | apl-mir-1559-3p@@gga-mir-1559-3p | ctacatgtatgcatttgatcag | 0 | 1 | 0 | 0 | 1 | 0 | 1 | 0 |
| apl-mir-1563 | apl-mir-1563@@gga-mir-1563 | acagtgcttcctccttgtaga | 12 | 4 | 4 | 4 | 19 | 5 | 8 | 6 |
| apl-mir-1575 | apl-mir-1575@@gga-mir-1575 | acgtgctgccagctgagc | 1 | 9 | 7 | 5 | 5 | 3 | 3 | 1 |
| apl-mir-1597-3p | apl-mir-1597-3p@@gga-mir-1597-3p | gaagagctctgcaagcatgcaa | 0 | 6 | 7 | 2 | 3 | 2 | 5 | 4 |
| apl-mir-1597-5p | apl-mir-1597-5p@@gga-mir-1597-5p | acatgtttacagggctcttcat | 7 | 7 | 9 | 11 | 5 | 6 | 9 | 6 |
| apl-mir-15a-3p | apl-mir-15a-3p@@tgu-mir-15a-3p | aggccatattgtgctgcctca | 1 | 0 | 0 | 1 | 0 | 0 | 0 | 0 |
| apl-mir-15a-5p | apl-mir-15a-5p@@tgu-mir-15a-5p | tagcagcacataatggtttgt | 270 | 774 | 1342 | 499 | 509 | 278 | 584 | 295 |
| apl-mir-15b-5p | apl-mir-15b-5p@@tgu-mir-15b-5p | tagcagcacatcatggtttgc | 292 | 992 | 1722 | 581 | 643 | 222 | 692 | 338 |
| apl-mir-15c-3p | apl-mir-15c-3p@@tgu-mir-15c-3p | agaccattctgggctgcctca | 0 | 0 | 1 | 0 | 1 | 1 | 4 | 1 |
| apl-mir-15c-5p | apl-mir-15c-5p@@tgu-mir-15c-5p | aacagtgcagaaccatgatgtgt | 2 | 3 | 8 | 0 | 0 | 0 | 7 | 0 |
| apl-mir-1601 | apl-mir-1601@@gga-mir-1601 | tgcgcccctctgcacctgcga | 0 | 0 | 1 | 0 | 1 | 0 | 0 | 0 |
| apl-mir-16-2-3p | apl-mir-16-2-3p@@gga-mir-16-2-3p | ccaatattattgtgctgcttaa | 2 | 3 | 2 | 5 | 3 | 0 | 5 | 4 |
| apl-mir-1635 | apl-mir-1635@@gga-mir-1635 | tgtccctgtgctgtgctcctgg | 1 | 6 | 10 | 10 | 9 | 3 | 3 | 1 |
| apl-mir-1641 | apl-mir-1641@@tgu-mir-1641 | tgaggattaatgactgtctgggg | 2 | 5 | 6 | 2 | 2 | 1 | 9 | 3 |
| apl-mir-1662 | apl-mir-1662@@tgu-mir-1662 | ttgacatcatcatacttgggat | 95 | 70 | 141 | 163 | 68 | 73 | 63 | 120 |
| apl-mir-1672 | apl-mir-1672@@gga-mir-1672 | tgttcatcccttcctgagc | 0 | 1 | 0 | 1 | 0 | 1 | 0 | 2 |
| apl-mir-1677-5p | apl-mir-1677-5p@@tgu-mir-1677-5p | tcctgcaccactgaagtca | 0 | 3 | 1 | 1 | 3 | 1 | 0 | 2 |
| apl-mir-16a-5p | apl-mir-16a-5p@@tgu-mir-16a-5p | tagcagcacgtaaatattggtg | 1731 | 3214 | 3758 | 2571 | 1388 | 1682 | 2710 | 1475 |
| apl-mir-16c | apl-mir-16c@@tgu-mir-16c | tagcagtacgtaaatactgt | 25 | 45 | 43 | 15 | 13 | 17 | 24 | 19 |
| apl-mir-16c-3p | apl-mir-16c-3p@@gga-mir-16c-3p | tccagtattgcattgctgcttta | 2 | 2 | 0 | 0 | 0 | 0 | 0 | 3 |
| apl-mir-1754-5p | apl-mir-1754-5p@@gga-mir-1754-5p | ctgctggagctgtgcctgg | 0 | 2 | 5 | 4 | 3 | 3 | 9 | 5 |
| apl-mir-1756 | apl-mir-1756@@tgu-mir-1756 | tccagctggtgtgaaccc | 2 | 15 | 6 | 15 | 9 | 2 | 3 | 1 |
| apl-mir-1762 | apl-mir-1762@@gga-mir-1762 | aggagggaaccagagcca | 1 | 3 | 0 | 0 | 0 | 0 | 0 | 0 |
| apl-mir-1770 | apl-mir-1770@@gga-mir-1770 | ctgaggagggagggagga | 2 | 6 | 4 | 1 | 2 | 1 | 0 | 1 |
| apl-mir-1784b-5p | apl-mir-1784b-5p@@gga-mir-1784b-5p | tgacttaaataggagcagaatt | 32 | 10 | 6 | 29 | 9 | 11 | 22 | 9 |
| apl-mir-17a-3p | apl-mir-17a-3p@@tgu-mir-17a-3p | actgcagtgaaggcacttgtag | 1 | 12 | 10 | 17 | 12 | 11 | 10 | 5 |
| apl-mir-17a-5p | apl-mir-17a-5p@@tgu-mir-17a-5p | caaagtgcttacagtgcaggta | 662 | 1545 | 1378 | 1473 | 830 | 545 | 1041 | 727 |
| apl-mir-181a-1-3p | apl-mir-181a-1-3p@@tgu-mir-181a-1-3p | ccatcgaccgttgattgtacc | 1 | 2 | 3 | 2 | 1 | 1 | 2 | 3 |
| apl-mir-181a-2-3p | apl-mir-181a-2-3p@@tgu-mir-181a-2-3p | ccatcgaccgttgactgtacc | 4 | 3 | 9 | 1 | 2 | 4 | 1 | 4 |
| apl-mir-181a-5p | apl-mir-181a-5p@@tgu-mir-181a-5p | aacattcaacgctgtcggtgagt | 7444 | 13026 | 9807 | 13097 | 12039 | 15174 | 9901 | 12452 |
| apl-mir-181b-1-3p | apl-mir-181b-1-3p@@gga-mir-181b-1-3p | tcactgaacaatgaatgcaac | 0 | 3 | 1 | 1 | 1 | 1 | 3 | 0 |
| apl-mir-181b-2-3p | apl-mir-181b-2-3p@@gga-mir-181b-2-3p | tcactgatcaatgaatgcaaa | 2 | 0 | 0 | 0 | 0 | 0 | 0 | 0 |
| apl-mir-181b-5p | apl-mir-181b-5p@@tgu-mir-181b-5p | aacattcattgctgtcggtgggt | 2962 | 6127 | 3710 | 5081 | 4363 | 3653 | 3214 | 3988 |
| apl-mir-183 | apl-mir-183@@tgu-mir-183 | tatggcactggtagaattcact | 32 | 27 | 73 | 20 | 36 | 28 | 40 | 23 |
| apl-mir-184 | apl-mir-184@@tgu-mir-184 | tggacggagaactgataagggt | 176 | 835 | 591 | 235 | 138 | 75 | 788 | 155 |
| apl-mir-187-5p | apl-mir-187-5p@@tgu-mir-187-5p | tgtgtgttgtagcaatggt | 2 | 5 | 0 | 6 | 1 | 5 | 1 | 1 |
| apl-mir-18b | apl-mir-18b@@tgu-mir-18b | taaggtgcatctagtgcagt | 10 | 27 | 41 | 15 | 9 | 12 | 28 | 11 |
| apl-mir-18b-3p | apl-mir-18b-3p@@gga-mir-18b-3p | tgccctaaatgctccttctggc | 1 | 0 | 1 | 0 | 1 | 0 | 0 | 1 |
| apl-mir-190b-3p | apl-mir-190b-3p@@gga-mir-190b-3p | aactaaatatcaaacatattctta | 6 | 5 | 9 | 6 | 2 | 5 | 3 | 8 |
| apl-mir-190b-5p | apl-mir-190b-5p@@gga-mir-190b-5p | tgatatgtttgatattaggttgt | 11 | 19 | 23 | 28 | 4 | 17 | 14 | 4 |
| apl-mir-193a-3p | apl-mir-193a-3p@@gga-mir-193a-3p | tgggactttgtaggccagttga | 10 | 14 | 19 | 20 | 15 | 8 | 33 | 13 |
| apl-mir-193b-3p | apl-mir-193b-3p@@tgu-mir-193b-3p | actggcctacaaagtcccagt | 0 | 4 | 3 | 2 | 0 | 1 | 10 | 0 |
| apl-mir-193b-5p | apl-mir-193b-5p@@tgu-mir-193b-5p | atctcgcccgcaaagacccagc | 14 | 22 | 23 | 27 | 29 | 35 | 17 | 16 |
| apl-mir-194-3p | apl-mir-194-3p@@tgu-mir-194-3p | ccagtggagatgctgttacttt | 1 | 2 | 1 | 1 | 7 | 1 | 0 | 14 |
| apl-mir-194-5p | apl-mir-194-5p@@tgu-mir-194-5p | tgtaacagcaactccatgtggac | 37 | 44 | 30 | 46 | 902 | 22 | 282 | 3296 |
| apl-mir-196-1-3p | apl-mir-196-1-3p@@gga-mir-196-1-3p | caagaacatcaaactacctgat | 1 | 10 | 1 | 5 | 0 | 0 | 1 | 2 |
| apl-mir-196-2-3p | apl-mir-196-2-3p@@gga-mir-196-2-3p | ctacagcacgaaactgccttaa | 0 | 1 | 1 | 1 | 0 | 1 | 0 | 0 |
| apl-mir-196a-5p | apl-mir-196a-5p@@hsa-mir-196a-5p | taggtagtttcatgttgttggg | 38 | 66 | 33 | 122 | 5 | 157 | 85 | 7 |
| apl-mir-199-3p | apl-mir-199-3p@@tgu-mir-199-3p | acagtagtctgcacattggtta | 15950 | 22566 | 30964 | 21799 | 22277 | 27136 | 24036 | 21279 |
| apl-mir-199-5p | apl-mir-199-5p@@gga-mir-199-5p | cccagtgttcagactacctgttc | 20932 | 55221 | 56239 | 35349 | 41614 | 23476 | 50297 | 23442 |
| apl-mir-202-5p | apl-mir-202-5p@@gga-mir-202-5p | ttcctatgcatatatttcc | 63 | 247 | 73 | 516 | 49 | 40 | 22 | 24 |
| apl-mir-204 | apl-mir-204@@tgu-mir-204 | ttccctttgtcatcctatgcct | 49 | 86 | 227 | 124 | 52 | 51 | 67 | 47 |
| apl-mir-204-3p | apl-mir-204-3p@@hsa-mir-204-3p | gctgggaaggcaaagggacgt | 1 | 4 | 5 | 4 | 2 | 0 | 4 | 1 |
| apl-mir-205 | apl-mir-205@@tgu-mir-205 | cccttcattccaccggaatctg | 5 | 107 | 59 | 47 | 6 | 8 | 7 | 17 |
| apl-mir-205a | apl-mir-205a@@gga-mir-205a | tccttcattccaccggagtctgt | 2 | 20 | 39 | 12 | 6 | 19 | 3 | 6 |
| apl-mir-206 | apl-mir-206@@gga-mir-206 | tggaatgtaaggaagtgtgt | 2465 | 1417 | 1741 | 2055 | 1549 | 2440 | 2881 | 3167 |
| apl-mir-20a-3p | apl-mir-20a-3p@@tgu-mir-20a-3p | actgcattataagcacttaaagt | 0 | 3 | 0 | 0 | 0 | 0 | 0 | 1 |
| apl-mir-20a-5p | apl-mir-20a-5p@@tgu-mir-20a-5p | taaagtgcttatagtgcaggtag | 1403 | 1877 | 1641 | 2336 | 1038 | 1808 | 1827 | 1364 |
| apl-mir-20b-3p | apl-mir-20b-3p@@tgu-mir-20b-3p | ttgcccacattacattttctatgt | 0 | 1 | 2 | 1 | 0 | 1 | 0 | 1 |
| apl-mir-20b-5p | apl-mir-20b-5p@@tgu-mir-20b-5p | caaagtgctcatagtgcaggtag | 298 | 623 | 419 | 481 | 203 | 257 | 583 | 237 |
| apl-mir-2110 | apl-mir-2110@@hsa-mir-2110 | ttccaggccgtttccccaccct | 0 | 0 | 0 | 1 | 0 | 1 | 1 | 3 |
| apl-mir-211-3p | apl-mir-211-3p@@hsa-mir-211-3p | gcagggacagcaaagggatgc | 0 | 0 | 0 | 9 | 10 | 0 | 1 | 1 |
| apl-mir-2130 | apl-mir-2130@@gga-mir-2130 | ccctgccttgcagagctc | 0 | 0 | 0 | 0 | 1 | 0 | 0 | 1 |
| apl-mir-21-3p | apl-mir-21-3p@@gga-mir-21-3p | caacaacagtcggtaggctgtc | 15 | 21 | 37 | 21 | 17 | 20 | 19 | 28 |
| apl-mir-214-5p | apl-mir-214-5p@@tgu-mir-214-5p | cacagcaagtgtagacaggc | 6 | 5 | 6 | 11 | 7 | 4 | 1 | 6 |
| apl-mir-215-3p | apl-mir-215-3p@@gga-mir-215-3p | cctgtcatttctgtaggccaata | 3 | 5 | 0 | 1 | 18 | 0 | 5 | 65 |
| apl-mir-215-5p | apl-mir-215-5p@@tgu-mir-215-5p | atgacctatgaattgacagact | 263 | 812 | 281 | 443 | 36117 | 140 | 5443 | 83891 |
| apl-mir-21-5p | apl-mir-21-5p@@tgu-mir-21-5p | tagcttttcagactgatgc | 1081 | 1202 | 2539 | 2998 | 1424 | 1481 | 1360 | 2134 |
| apl-mir-216a-3p | apl-mir-216a-3p@@hsa-mir-216a-3p | acagtggtatctgggattatg | 0 | 0 | 1 | 0 | 0 | 0 | 0 | 0 |
| apl-mir-216a-5p | apl-mir-216a-5p@@tgu-mir-216a-5p | taatctcagctggcaactgtga | 0 | 0 | 0 | 5 | 1 | 3 | 3 | 6 |
| apl-mir-216b | apl-mir-216b@@gga-mir-216b | aaatctctgcaggcaaatgtg | 3 | 5 | 10 | 24 | 10 | 6 | 6 | 10 |
| apl-mir-216b-3p | apl-mir-216b-3p@@tgu-mir-216b-3p | caattacctgtagagattct | 0 | 0 | 0 | 2 | 0 | 0 | 2 | 0 |
| apl-mir-218-3p | apl-mir-218-3p@@tgu-mir-218-3p | tccacctgaacatggttctggcgag | 0 | 1 | 1 | 1 | 0 | 0 | 0 | 1 |
| apl-mir-218-5p | apl-mir-218-5p@@tgu-mir-218-5p | ttgtgcttgatctaaccatgt | 39530 | 39426 | 30424 | 34692 | 23151 | 19551 | 37456 | 21740 |
| apl-mir-2188-3p | apl-mir-2188-3p@@gga-mir-2188-3p | gatatatgtggtcagacctatc | 24 | 37 | 40 | 49 | 29 | 77 | 134 | 30 |
| apl-mir-2188-5p | apl-mir-2188-5p@@tgu-mir-2188-5p | aaggtccaacctcacatgtcct | 326 | 639 | 1280 | 533 | 1026 | 1260 | 2209 | 512 |
| apl-mir-219b | apl-mir-219b@@tgu-mir-219b | cacaagaattgcgtttggaca | 54 | 171 | 198 | 130 | 48 | 100 | 112 | 88 |
| apl-mir-22-3p | apl-mir-22-3p@@gga-mir-22-3p | agctgccagttgaagaactgt | 8 | 11 | 9 | 14 | 12 | 6 | 10 | 13 |
| apl-mir-22-5p | apl-mir-22-5p@@gga-mir-22-5p | agttcttcagtggcaagcttt | 302 | 476 | 585 | 480 | 322 | 186 | 544 | 289 |
| apl-mir-23-5p | apl-mir-23-5p@@tgu-mir-23-5p | tctctggttcctggcatcctcc | 2 | 0 | 5 | 2 | 2 | 1 | 0 | 2 |
| apl-mir-26-3p | apl-mir-26-3p@@tgu-mir-26-3p | cctattcttggttacttgcact | 21 | 24 | 22 | 20 | 10 | 11 | 15 | 7 |
| apl-mir-26-5p | apl-mir-26-5p@@tgu-mir-26-5p | ttcaagtaatccaggataggct | 75057 | 104631 | 109125 | 125424 | 114394 | 127051 | 97804 | 101387 |
| apl-mir-2963-3p | apl-mir-2963-3p@@tgu-mir-2963-3p | ctcatttttgggatgtagagagtaa | 2 | 7 | 13 | 15 | 6 | 5 | 4 | 3 |
| apl-mir-2965 | apl-mir-2965@@tgu-mir-2965 | gcagactgcttcagagagc | 0 | 3 | 3 | 0 | 0 | 2 | 4 | 0 |
| apl-mir-2970-5p | apl-mir-2970-5p@@tgu-mir-2970-5p | ttactgctgactggagagc | 1 | 6 | 2 | 5 | 2 | 3 | 1 | 2 |
| apl-mir-2976 | apl-mir-2976@@tgu-mir-2976 | cggagcggagcggggaggg | 2 | 6 | 15 | 6 | 8 | 4 | 3 | 14 |
| apl-mir-2982 | apl-mir-2982@@tgu-mir-2982 | cggggctggagggagggc | 1 | 3 | 9 | 7 | 4 | 4 | 2 | 4 |
| apl-mir-2987-5p | apl-mir-2987-5p@@tgu-mir-2987-5p | ctcggctctggctgcggc | 1 | 2 | 0 | 2 | 4 | 3 | 3 | 1 |
| apl-mir-2995 | apl-mir-2995@@tgu-mir-2995 | tcgcactgttcgtaacctctt | 47 | 22 | 34 | 16 | 5 | 4 | 300 | 8 |
| apl-mir-29a-1-5p | apl-mir-29a-1-5p@@tgu-mir-29a-1-5p | actgatttcttttggtgtgt | 5 | 1 | 10 | 6 | 4 | 3 | 1 | 4 |
| apl-mir-30a-3p | apl-mir-30a-3p@@tgu-mir-30a-3p | ctttcagtcggatgtttacagc | 4256 | 7840 | 4444 | 7202 | 4497 | 2313 | 6050 | 3819 |
| apl-mir-30a-5p | apl-mir-30a-5p@@tgu-mir-30a-5p | tgtaaacatccttgactggaagct | 10146 | 7032 | 4217 | 10743 | 4447 | 12007 | 9025 | 8158 |
| apl-mir-30b-3p | apl-mir-30b-3p@@gga-mir-30b-3p | ctggggggtggatgtttacttc | 1 | 3 | 0 | 0 | 1 | 2 | 2 | 0 |
| apl-mir-30b-5p | apl-mir-30b-5p@@tgu-mir-30b-5p | tgtaaacatcctcgactggaagct | 50126 | 59661 | 45896 | 59396 | 24631 | 32105 | 43345 | 32866 |
| apl-mir-30c-1-3p | apl-mir-30c-1-3p@@gga-mir-30c-1-3p | ttggggagaggattgtagtgga | 2 | 2 | 0 | 0 | 1 | 1 | 0 | 2 |
| apl-mir-30c-3p | apl-mir-30c-3p@@tgu-mir-30c-3p | tgggagaaggctgtttactct | 192 | 364 | 299 | 286 | 142 | 99 | 257 | 136 |
| apl-mir-30c-5p | apl-mir-30c-5p@@tgu-mir-30c-5p | tgtaaacatcctacactctcagc | 9172 | 16048 | 12671 | 14254 | 11938 | 11019 | 15675 | 8933 |
| apl-mir-30d-3p | apl-mir-30d-3p@@tgu-mir-30d-3p | tttcagtcagatgtttgctgc | 28 | 93 | 55 | 71 | 29 | 28 | 48 | 25 |
| apl-mir-30d-5p | apl-mir-30d-5p@@tgu-mir-30d-5p | tgtaaacatccccgactggaagc | 16808 | 28345 | 19594 | 24792 | 16157 | 16583 | 23772 | 10494 |
| apl-mir-30e | apl-mir-30e@@tgu-mir-30e | tgtaaacatcctacactcagct | 450 | 676 | 836 | 730 | 558 | 396 | 667 | 296 |
| apl-mir-31 | apl-mir-31@@tgu-mir-31 | aggcaagatgttggcatagctg | 128 | 1033 | 312 | 568 | 186 | 30 | 94 | 57 |
| apl-mir-3125 | apl-mir-3125@@hsa-mir-3125 | tgggaagctgtggaggga | 0 | 4 | 4 | 3 | 0 | 0 | 0 | 1 |
| apl-mir-31-3p | apl-mir-31-3p@@gga-mir-31-3p | gctatgccaacatattgtcatc | 0 | 2 | 0 | 2 | 0 | 0 | 0 | 0 |
| apl-mir-3196 | apl-mir-3196@@hsa-mir-3196 | cggggcggcagggaggg | 1 | 6 | 8 | 11 | 3 | 1 | 1 | 7 |
| apl-mir-32 | apl-mir-32@@tgu-mir-32 | tattgcacattactaagttgca | 210 | 226 | 192 | 397 | 93 | 387 | 262 | 163 |
| apl-mir-320d | apl-mir-320d@@hsa-mir-320d | caaaaaaagctgggttgagaggccgca | 74 | 88 | 60 | 79 | 41 | 35 | 107 | 75 |
| apl-mir-32-3p | apl-mir-32-3p@@gga-mir-32-3p | caatttagtgtgtgcgatact | 28 | 58 | 50 | 38 | 17 | 13 | 36 | 13 |
| apl-mir-34a-3p | apl-mir-34a-3p@@gga-mir-34a-3p | caatcagcaagtatactgcccta | 0 | 0 | 2 | 1 | 1 | 0 | 1 | 0 |
| apl-mir-34a-5p | apl-mir-34a-5p@@gga-mir-34a-5p | tggcagtgtcttagctggttgtt | 48 | 38 | 38 | 70 | 32 | 28 | 99 | 35 |
| apl-mir-34b | apl-mir-34b@@tgu-mir-34b | aggcagtgtagttagctgattgc | 2 | 18 | 2 | 21 | 21 | 163 | 62 | 51 |
| apl-mir-34b-3p | apl-mir-34b-3p@@gga-mir-34b-3p | aatcactaaattcactgccatc | 0 | 8 | 4 | 2 | 8 | 34 | 7 | 4 |
| apl-mir-34c-3p | apl-mir-34c-3p@@gga-mir-34c-3p | aatcactaaccacacagccagg | 0 | 3 | 1 | 2 | 6 | 5 | 5 | 3 |
| apl-mir-34c-5p | apl-mir-34c-5p@@tgu-mir-34c-5p | aggcagtgtagttagctgattgt | 4 | 12 | 4 | 22 | 20 | 257 | 73 | 63 |
| apl-mir-3529 | apl-mir-3529@@gga-mir-3529 | gggcagactgtgacttgttgt | 1 | 1 | 10 | 2 | 2 | 0 | 4 | 0 |
| apl-mir-3536 | apl-mir-3536@@gga-mir-3536 | ctgcattctagtagaccctttctg | 0 | 1 | 1 | 0 | 2 | 2 | 3 | 2 |
| apl-mir-3537 | apl-mir-3537@@gga-mir-3537 | atgagtgctgtagcagagt | 1 | 4 | 3 | 2 | 4 | 1 | 2 | 1 |
| apl-mir-3616-3p | apl-mir-3616-3p@@hsa-mir-3616-3p | ctgcatcatgaaaagtggggttg | 0 | 1 | 0 | 1 | 0 | 0 | 0 | 0 |
| apl-mir-375 | apl-mir-375@@tgu-mir-375 | tttgttcgttcggctcgcgtt | 12 | 9 | 9 | 8 | 161 | 6 | 23 | 96 |
| apl-mir-378c | apl-mir-378c@@hsa-mir-378c | gctggacttggagtcagaa | 4 | 9 | 3 | 6 | 2 | 5 | 3 | 3 |
| apl-mir-3914 | apl-mir-3914@@hsa-mir-3914 | ctagaaaatgagaagaagaa | 1 | 3 | 1 | 3 | 1 | 4 | 2 | 2 |
| apl-mir-425-3p | apl-mir-425-3p@@gga-mir-425-3p | catcggggatgtcgtgtctgtcc | 460 | 865 | 993 | 655 | 474 | 308 | 674 | 363 |
| apl-mir-425-5p | apl-mir-425-5p@@gga-mir-425-5p | aatgacacgatcactcccgctgag | 1407 | 3195 | 5103 | 2619 | 1627 | 1095 | 3565 | 1280 |
| apl-mir-4277 | apl-mir-4277@@hsa-mir-4277 | tgagtactgtgctcagctttgggcc | 15 | 41 | 3 | 65 | 8 | 0 | 3 | 4 |
| apl-mir-4454 | apl-mir-4454@@hsa-mir-4454 | tccgagtcacggcacca | 77 | 36 | 88 | 130 | 53 | 6 | 76 | 99 |
| apl-mir-449a | apl-mir-449a@@gga-mir-449a | tggcagtgtatgttagctggt | 2 | 1 | 1 | 3 | 3 | 8 | 8 | 0 |
| apl-mir-449c-5p | apl-mir-449c-5p@@gga-mir-449c-5p | tggcagtgcctgttagctggctgtt | 2 | 6 | 0 | 1 | 3 | 5 | 10 | 0 |
| apl-mir-449d-3p | apl-mir-449d-3p@@gga-mir-449d-3p | caactaactacactgccaga | 0 | 6 | 0 | 0 | 0 | 0 | 0 | 0 |
| apl-mir-449d-5p | apl-mir-449d-5p@@gga-mir-449d-5p | aggcagtgtgttgttagttagct | 2 | 12 | 7 | 6 | 1 | 2 | 13 | 1 |
| apl-mir-455-3p | apl-mir-455-3p@@tgu-mir-455-3p | catgcagtccatgggcatataca | 7 | 43 | 141 | 39 | 31 | 13 | 34 | 17 |
| apl-mir-455-5p | apl-mir-455-5p@@tgu-mir-455-5p | tatgtgcccttggactacatcgt | 857 | 2351 | 4491 | 1659 | 836 | 508 | 1264 | 704 |
| apl-mir-458b-5p | apl-mir-458b-5p@@gga-mir-458b-5p | tagctctttgaatggtact | 0 | 0 | 0 | 0 | 1 | 0 | 1 | 0 |
| apl-mir-460a-3p | apl-mir-460a-3p@@tgu-mir-460a-3p | acagcgcatacaatgtggatt | 6 | 1 | 0 | 4 | 1 | 9 | 1 | 2 |
| apl-mir-460a-5p | apl-mir-460a-5p@@tgu-mir-460a-5p | cctgcattgtacacactgtgt | 36 | 27 | 20 | 44 | 32 | 51 | 25 | 41 |
| apl-mir-460b-3p | apl-mir-460b-3p@@tgu-mir-460b-3p | cagcgcatgcaatgtggaca | 1 | 0 | 0 | 2 | 2 | 0 | 1 | 2 |
| apl-mir-460b-5p | apl-mir-460b-5p@@gga-mir-460b-5p | tcctcattgtacatgctgtgtg | 250 | 204 | 141 | 304 | 230 | 414 | 392 | 400 |
| apl-mir-4649-5p | apl-mir-4649-5p@@hsa-mir-4649-5p | cggggcgaggggtggggg | 4 | 5 | 5 | 2 | 6 | 4 | 0 | 18 |
| apl-mir-4672 | apl-mir-4672@@hsa-mir-4672 | tctggcgctctgtccagctgcc | 0 | 4 | 15 | 7 | 3 | 2 | 9 | 1 |
| apl-mir-4759 | apl-mir-4759@@hsa-mir-4759 | gctgtctcttccaacatctaga | 1 | 5 | 7 | 3 | 7 | 3 | 7 | 2 |
| apl-mir-4763-3p | apl-mir-4763-3p@@hsa-mir-4763-3p | aagggaggcagcaggggctggtg | 2 | 5 | 6 | 1 | 2 | 1 | 1 | 1 |
| apl-mir-499-5p | apl-mir-499-5p@@tgu-mir-499-5p | ttaagacttgtagtgatgttt | 164 | 738 | 124 | 596 | 35 | 223 | 94 | 165 |
| apl-mir-504-3p | apl-mir-504-3p@@hsa-mir-504-3p | ggatgtgcagggcagggactggggga | 1 | 3 | 1 | 0 | 2 | 2 | 3 | 3 |
| apl-mir-505-3p | apl-mir-505-3p@@hsa-mir-505-3p | tcaggaacacttgctggttt | 0 | 2 | 2 | 0 | 0 | 0 | 0 | 0 |
| apl-mir-551-3p | apl-mir-551-3p@@tgu-mir-551-3p | gcgacccatgcttggtttcca | 28 | 39 | 29 | 25 | 23 | 11 | 31 | 33 |
| apl-mir-5590-3p | apl-mir-5590-3p@@hsa-mir-5590-3p | catgaactttattgccagtctgg | 3 | 2 | 1 | 1 | 0 | 0 | 1 | 1 |
| apl-mir-586 | apl-mir-586@@hsa-mir-586 | ctaaaaatacaattctg | 1 | 0 | 0 | 0 | 0 | 0 | 1 | 0 |
| apl-mir-606 | apl-mir-606@@hsa-mir-606 | tgatcttgattttcagta | 10 | 129 | 43 | 11 | 25 | 5 | 9 | 4 |
| apl-mir-6085 | apl-mir-6085@@hsa-mir-6085 | tggggctgggggaggtg | 1 | 1 | 5 | 1 | 0 | 2 | 2 | 2 |
| apl-mir-6124 | apl-mir-6124@@hsa-mir-6124 | gaggaaggaagggggagct | 0 | 1 | 0 | 3 | 5 | 0 | 1 | 0 |
| apl-mir-631 | apl-mir-631@@hsa-mir-631 | atgcccagacctcagctgc | 0 | 0 | 1 | 0 | 1 | 0 | 0 | 1 |
| apl-mir-6511b-5p | apl-mir-6511b-5p@@hsa-mir-6511b-5p | tggactgcaggcagaagctga | 0 | 4 | 1 | 0 | 2 | 2 | 4 | 2 |
| apl-mir-6575-3p | apl-mir-6575-3p@@gga-mir-6575-3p | cccaagcaggctgatgtcaggc | 0 | 2 | 0 | 0 | 2 | 5 | 0 | 0 |
| apl-mir-6652-5p | apl-mir-6652-5p@@gga-mir-6652-5p | gatgcggtgggacagctcg | 0 | 42 | 1 | 18 | 2 | 0 | 1 | 0 |
| apl-mir-6669-3p | apl-mir-6669-3p@@gga-mir-6669-3p | gcgcagcctggccgagtccag | 3 | 7 | 2 | 3 | 1 | 0 | 2 | 6 |
| apl-mir-6672-3p | apl-mir-6672-3p@@gga-mir-6672-3p | tgtgttccagtttgtgcccattgc | 5 | 12 | 10 | 13 | 5 | 1 | 6 | 6 |
| apl-mir-6726-5p | apl-mir-6726-5p@@hsa-mir-6726-5p | aagagctggggtctgagcc | 0 | 18 | 9 | 27 | 15 | 3 | 3 | 5 |
| apl-mir-6728-3p | apl-mir-6728-3p@@hsa-mir-6728-3p | tggggacgggagagcagagctgg | 1 | 4 | 1 | 2 | 2 | 0 | 1 | 3 |
| apl-mir-6753-5p | apl-mir-6753-5p@@hsa-mir-6753-5p | agagcagggctgaaact | 1 | 1 | 1 | 1 | 1 | 1 | 0 | 0 |
| apl-mir-6773-3p | apl-mir-6773-3p@@hsa-mir-6773-3p | tggctatcacttctctgccata | 0 | 1 | 1 | 1 | 1 | 0 | 2 | 2 |
| apl-mir-6787-3p | apl-mir-6787-3p@@hsa-mir-6787-3p | cagctgctgccctcttcctgc | 0 | 1 | 7 | 0 | 0 | 1 | 0 | 2 |
| apl-mir-6796-3p | apl-mir-6796-3p@@hsa-mir-6796-3p | ctccatcctctcccctccccttc | 3 | 7 | 12 | 5 | 6 | 0 | 4 | 7 |
| apl-mir-6837-3p | apl-mir-6837-3p@@hsa-mir-6837-3p | aaactgtgactctgctgca | 1 | 2 | 0 | 0 | 1 | 2 | 3 | 0 |
| apl-mir-7106-5p | apl-mir-7106-5p@@hsa-mir-7106-5p | attgggaggaggggatggg | 1 | 6 | 2 | 6 | 2 | 0 | 0 | 2 |
| apl-mir-7109-3p | apl-mir-7109-3p@@hsa-mir-7109-3p | gccctctcctgccctgc | 0 | 2 | 2 | 0 | 0 | 0 | 0 | 0 |
| apl-mir-7-1-3p | apl-mir-7-1-3p@@tgu-mir-7-1-3p | caacaaatcacagtctgccata | 46 | 86 | 107 | 50 | 54 | 44 | 93 | 41 |
| apl-mir-7156-3p | apl-mir-7156-3p@@hsa-mir-7156-3p | ccagagcatctgcagccacttg | 2 | 11 | 8 | 14 | 7 | 1 | 1 | 2 |
| apl-mir-7162-3p | apl-mir-7162-3p@@hsa-mir-7162-3p | cctgaggtggaacaactttagaggtg | 0 | 4 | 0 | 1 | 0 | 0 | 1 | 0 |
| apl-mir-7-4-3p | apl-mir-7-4-3p@@tgu-mir-7-4-3p | caacaaatcatagcctgccata | 3 | 1 | 1 | 2 | 0 | 0 | 0 | 0 |
| apl-mir-7442-3p | apl-mir-7442-3p@@gga-mir-7442-3p | tcgtcctcttcctcctcctcc | 2 | 15 | 125 | 11 | 12 | 4 | 11 | 15 |
| apl-mir-757 | apl-mir-757@@gga-mir-757 | tctggtgagctgcagatgagc | 0 | 3 | 4 | 3 | 2 | 1 | 2 | 2 |
| apl-mir-7-5p | apl-mir-7-5p@@tgu-mir-7-5p | tggaagactagtgattttgttgtt | 12334 | 27083 | 19970 | 18797 | 6616 | 7068 | 28625 | 7449 |
| apl-mir-762 | apl-mir-762@@gga-mir-762 | tggggagggagggaggtg | 18 | 47 | 50 | 32 | 4 | 5 | 5 | 16 |
| apl-mir-9-3p | apl-mir-9-3p@@gga-mir-9-3p | ataaagctagagaaccgaatgta | 0 | 4 | 1 | 2 | 0 | 1 | 0 | 0 |
| apl-mir-9-4-3p | apl-mir-9-4-3p@@gga-mir-9-4-3p | ataaagctagataaccgaaagta | 12 | 14 | 2 | 9 | 11 | 16 | 15 | 8 |
| apl-mir-9-5p | apl-mir-9-5p@@tgu-mir-9-5p | tctttggttatctagctgtatga | 1182 | 1257 | 501 | 1376 | 856 | 1099 | 1569 | 882 |
| apl-mir-96-3p | apl-mir-96-3p@@gga-mir-96-3p | caattatgtgtagtgccaatat | 0 | 2 | 0 | 0 | 0 | 0 | 0 | 0 |
| apl-mir-96-5p | apl-mir-96-5p@@hsa-mir-96-5p | tttggcactagcacatttttgct | 9 | 45 | 26 | 23 | 23 | 20 | 16 | 8 |
| apl-mir-99-3p | apl-mir-99-3p@@tgu-mir-99-3p | aagctcgcttctatgggtctg | 0 | 1 | 0 | 0 | 1 | 1 | 0 | 0 |
| apl-mir-99-5p | apl-mir-99-5p@@tgu-mir-99-5p | aacccgtagatccgatcttgt | 63799 | 180615 | 76448 | 174388 | 121463 | 85236 | 100579 | 65739 |

**Supplemental Table S1: The counts of all novel miRNAs in eight ovary samples.**

| **miRNA_id** | **blast_id** | **seq** | **GF1** | **GF2** | **GF3** | **GF4** | **JD1** | **JD2** | **JD3** | **JD4** |
| --- | --- | --- | --- | --- | --- | --- | --- | --- | --- | --- |
| kb742483.1_1731_mature | kb742483.1_1731_mature | ttgtggtagtgggggact | 11 | 30 | 14 | 13 | 17 | 11 | 8 | 6 |
| kb742915.1_23424_star | kb742915.1_23424_star | cactgcccagagcagc | 0 | 5 | 0 | 0 | 3 | 0 | 0 | 0 |
| kb743739.1_14645_star | kb743739.1_14645_star | tactttaaattcatctaggaaa | 0 | 0 | 1 | 0 | 0 | 0 | 0 | 0 |
| kb743912.1_17130_star | kb743912.1_17130_star | tcgtggttggagcttccagc | 0 | 3 | 2 | 9 | 2 | 0 | 1 | 4 |
| kb742571.1_4072_mature | kb742571.1_4072_mature | tggagcggctgcagaagc | 12 | 65 | 2 | 68 | 65 | 3 | 7 | 17 |
| kb744345.1_17158_star | kb744345.1_17158_star | agattaagcatcttcagtatata | 0 | 1 | 0 | 1 | 1 | 0 | 2 | 0 |
| kb742588.1_6980_star | kb742588.1_6980_star | gggactcctccggccctttttc | 1 | 1 | 2 | 0 | 2 | 0 | 1 | 0 |
| kb743876.1_25298_mature | kb743876.1_25298_mature | agagattatttttgaacagta | 0 | 0 | 1 | 0 | 0 | 0 | 5 | 0 |
| kb743434.1_14198_star | kb743434.1_14198_star | gaaaaagagttcattgctacag | 1 | 5 | 5 | 0 | 1 | 0 | 2 | 1 |
| kb744466.1_17606_star | kb744466.1_17606_star | ggaaggtggggataggtgatgaat | 0 | 1 | 1 | 1 | 0 | 0 | 0 | 0 |
| kb742833.1_53_star | kb742833.1_53_star | gcaggccaagcccatagc | 0 | 1 | 0 | 0 | 1 | 0 | 0 | 0 |
| kb743058.1_6251_mature | kb743058.1_6251_mature | tgcctgtcccttctgaaa | 1 | 3 | 2 | 5 | 1 | 2 | 0 | 1 |
| kb742750.1_2074_mature | kb742750.1_2074_mature | gagtgcctggtgcagaact | 0 | 5 | 3 | 10 | 6 | 0 | 0 | 3 |
| kb743217.1_9779_mature | kb743217.1_9779_mature | gtaagtgatgtaactgctctgata | 4 | 11 | 3 | 1 | 3 | 0 | 5 | 1 |
| kb744042.1_11203_mature | kb744042.1_11203_mature | taggcttcagcgtagact | 0 | 1 | 0 | 0 | 0 | 0 | 0 | 3 |
| kb744553.1_17005_star | kb744553.1_17005_star | attcagccgtgtttgg | 0 | 8 | 0 | 12 | 2 | 0 | 0 | 0 |
| kb742840.1_6695_mature | kb742840.1_6695_mature | aagtcggtgcacttctcatcc | 0 | 2 | 2 | 3 | 0 | 0 | 0 | 0 |
| kb742439.1_3172_star | kb742439.1_3172_star | cttttatttcagcacatttgcata | 0 | 4 | 0 | 1 | 1 | 0 | 3 | 1 |
| kb742851.1_13581_star | kb742851.1_13581_star | tgctggggcagggtgtgaggtcc | 0 | 0 | 2 | 1 | 0 | 0 | 1 | 0 |
| kb743105.1_19472_star | kb743105.1_19472_star | tccagccagtcccattccagctc | 2 | 2 | 5 | 2 | 0 | 2 | 0 | 1 |
| kb743311.1_18858_star | kb743311.1_18858_star | tagcacgccagcatacatgg | 0 | 2 | 0 | 0 | 1 | 0 | 0 | 1 |
| kb743608.1_9442_mature | kb743608.1_9442_mature | ttgttctttctccacagtt | 6 | 5 | 5 | 10 | 1 | 4 | 6 | 1 |
| kb742448.1_5978_mature | kb742448.1_5978_mature | acctgtggtgcatgtggaaga | 3 | 8 | 3 | 2 | 2 | 3 | 3 | 1 |
| kb743085.1_10571_mature | kb743085.1_10571_mature | gtttgtgatggtgaattc | 34 | 209 | 66 | 36 | 19 | 13 | 36 | 23 |
| kb748900.1_25258_mature | kb748900.1_25258_mature | tggtgaccagttgaccttcc | 0 | 9 | 1 | 0 | 1 | 0 | 0 | 0 |
| kb743260.1_3345_mature | kb743260.1_3345_mature | cagatttctgaaagtacgt | 0 | 0 | 3 | 0 | 0 | 0 | 0 | 0 |
| kb743446.1_11674_star | kb743446.1_11674_star | cacaaacaagagaagaaaagga | 1 | 0 | 3 | 0 | 0 | 1 | 1 | 0 |
| kb742777.1_8175_mature | kb742777.1_8175_mature | gcacaggaatggaactgggaac | 2 | 0 | 1 | 1 | 1 | 0 | 7 | 1 |
| kb743105.1_19469_mature | kb743105.1_19469_mature | accagcaggctgtggttctgtt | 1 | 0 | 7 | 0 | 1 | 0 | 1 | 3 |
| kb742471.1_390_mature | kb742471.1_390_mature | agaagtaatcttacgtcgc | 0 | 0 | 3 | 0 | 0 | 0 | 0 | 0 |
| kb743359.1_15222_mature | kb743359.1_15222_mature | cacggctgtgagataacacctct | 0 | 8 | 0 | 3 | 3 | 2 | 3 | 0 |
| kb742741.1_17419_mature | kb742741.1_17419_mature | tagtgactgacctccagctggact | 29 | 280 | 2 | 472 | 40 | 4 | 17 | 11 |
| kb742781.1_1894_star | kb742781.1_1894_star | ctggctggtcacgctccctc | 0 | 0 | 1 | 0 | 0 | 0 | 1 | 0 |
| kb742808.1_191_mature | kb742808.1_191_mature | ttgtataactttgaataaa | 1 | 1 | 1 | 1 | 0 | 0 | 5 | 0 |
| kb742559.1_5641_mature | kb742559.1_5641_mature | gaggacatggccatgctgacg | 1 | 0 | 3 | 3 | 2 | 4 | 4 | 0 |
| kb743186.1_17591_mature | kb743186.1_17591_mature | gaagacgtcgggctcacc | 0 | 0 | 4 | 3 | 1 | 1 | 0 | 1 |
| kb813882.1_25219_star | kb813882.1_25219_star | ccttcctcctcagctgctga | 1 | 2 | 1 | 1 | 2 | 0 | 2 | 1 |
| kb742833.1_45_mature | kb742833.1_45_mature | tagacttggaatctcctaat | 0 | 0 | 0 | 1 | 3 | 0 | 0 | 0 |
| kb743149.1_16899_mature | kb743149.1_16899_mature | tattcgggactcttgtgtatc | 1 | 1 | 5 | 1 | 1 | 2 | 1 | 0 |
| kb743171.1_10219_mature | kb743171.1_10219_mature | aagctgaaacttaaagga | 16 | 16 | 23 | 12 | 7 | 6 | 18 | 21 |
| kb742811.1_13116_star | kb742811.1_13116_star | ttcctgagcctggtggtcc | 0 | 0 | 2 | 1 | 0 | 0 | 0 | 2 |
| kb743509.1_7197_star | kb743509.1_7197_star | tgttctttgctaaaacttcaaga | 2 | 2 | 0 | 1 | 0 | 0 | 0 | 0 |
| kb744656.1_20733_mature | kb744656.1_20733_mature | tgctgtctgggcctgatcccct | 36 | 50 | 6 | 84 | 20 | 1 | 4 | 5 |
| kb743412.1_5060_mature | kb743412.1_5060_mature | tggggagcaggagagagacga | 0 | 0 | 4 | 2 | 0 | 1 | 1 | 2 |
| kb744899.1_8646_star | kb744899.1_8646_star | cttcagaaagagcaaagctcagt | 0 | 0 | 2 | 2 | 3 | 0 | 5 | 0 |
| kb743509.1_7188_star | kb743509.1_7188_star | tctaccaaatacaaggttctgtttt | 1 | 2 | 0 | 0 | 0 | 3 | 0 | 0 |
| kb742904.1_12934_star | kb742904.1_12934_star | ttctttcttgcccttct | 0 | 1 | 0 | 4 | 2 | 0 | 1 | 0 |
| kb743297.1_21460_mature | kb743297.1_21460_mature | tggttctgtttattttgac | 1 | 5 | 0 | 1 | 2 | 0 | 5 | 0 |
| kb743748.1_19042_mature | kb743748.1_19042_mature | tggaacttaaaggattgtttgtta | 15 | 178 | 4 | 254 | 10 | 5 | 18 | 8 |
| kb742722.1_13453_star | kb742722.1_13453_star | tctgccaggggaaaggagct | 2 | 1 | 1 | 0 | 0 | 0 | 0 | 0 |
| kb743210.1_15290_star | kb743210.1_15290_star | gtctctaggtagctatatgaaagacagct | 3 | 9 | 0 | 2 | 5 | 0 | 2 | 0 |
| kb742554.1_1833_mature | kb742554.1_1833_mature | tctggaagaggaagatctctgc | 3 | 2 | 8 | 2 | 3 | 1 | 5 | 2 |
| kb742975.1_9901_mature | kb742975.1_9901_mature | caaagctcacgtgcagccagtgtt | 0 | 1 | 3 | 0 | 1 | 0 | 0 | 2 |
| kb743359.1_15235_star | kb743359.1_15235_star | tggtggtcttgtaggcggaatgc | 0 | 1 | 0 | 1 | 1 | 0 | 1 | 0 |
| kb742540.1_13389_star | kb742540.1_13389_star | gttgcccactttctcctgcaa | 0 | 0 | 2 | 1 | 0 | 0 | 0 | 2 |
| kb744088.1_4017_mature | kb744088.1_4017_mature | ttgcgatgatgagctgctgagcac | 1 | 5 | 4 | 8 | 3 | 2 | 9 | 1 |
| kb743518.1_15376_mature | kb743518.1_15376_mature | cattctaccctcgccttcttgct | 5 | 14 | 8 | 5 | 1 | 2 | 1 | 3 |
| kb742585.1_9428_star | kb742585.1_9428_star | cctttgtgcagcctcacctcga | 0 | 4 | 0 | 2 | 5 | 4 | 2 | 2 |
| kb743062.1_19332_star | kb743062.1_19332_star | ggatgttggtgttgctctgtct | 1 | 3 | 8 | 3 | 0 | 0 | 5 | 0 |
| kb742777.1_8164_mature | kb742777.1_8164_mature | cagtctagatgttatttctct | 0 | 3 | 2 | 0 | 0 | 1 | 2 | 0 |
| kb742575.1_12387_star | kb742575.1_12387_star | acggcttcattcccaagaactaca | 1 | 4 | 1 | 1 | 1 | 1 | 2 | 0 |
| kb743649.1_15525_mature | kb743649.1_15525_mature | gagctgtgacttaggatg | 0 | 4 | 0 | 0 | 0 | 0 | 1 | 0 |
| kb742833.1_53_mature | kb742833.1_53_mature | taggacttgttctccag | 32 | 162 | 0 | 249 | 79 | 7 | 12 | 11 |
| kb744389.1_22416_star | kb744389.1_22416_star | catcacattagcaagtctc | 0 | 0 | 1 | 0 | 1 | 0 | 0 | 0 |
| kb745441.1_24452_mature | kb745441.1_24452_mature | gaggatctgctccacgagg | 219 | 1259 | 5 | 584 | 127 | 14 | 28 | 56 |
| kb743145.1_821_mature | kb743145.1_821_mature | aattaaaagaagttctggtg | 0 | 6 | 0 | 1 | 1 | 3 | 3 | 0 |
| kb743171.1_10227_mature | kb743171.1_10227_mature | tggagaagaacgtgaac | 2 | 1 | 6 | 5 | 0 | 0 | 4 | 1 |
| kb743217.1_9768_mature | kb743217.1_9768_mature | ctggtgagttatgttcttcatt | 7 | 23 | 34 | 2 | 2 | 0 | 14 | 2 |
| kb742730.1_8104_star | kb742730.1_8104_star | ggtccatctactgcagtgat | 0 | 3 | 0 | 0 | 0 | 1 | 0 | 0 |
| kb819001.1_24903_mature | kb819001.1_24903_mature | tgagtcttccggcagaaat | 0 | 2 | 1 | 13 | 0 | 1 | 3 | 0 |
| kb742559.1_5630_mature | kb742559.1_5630_mature | gccctgagagaacctcct | 0 | 1 | 0 | 0 | 0 | 0 | 2 | 1 |
| kb743471.1_15461_star | kb743471.1_15461_star | cagctgtgtcttccccgcag | 1 | 1 | 0 | 0 | 0 | 0 | 1 | 0 |
| kb742984.1_1905_mature | kb742984.1_1905_mature | ttaacaacagtgactctcagga | 53 | 56 | 51 | 38 | 47 | 47 | 61 | 89 |
| kb746010.1_24658_mature | kb746010.1_24658_mature | ttctgtgcatctgatgt | 5 | 12 | 0 | 25 | 1 | 1 | 0 | 1 |
| kb743642.1_19561_star | kb743642.1_19561_star | gcttggtaagtacttg | 0 | 0 | 2 | 0 | 0 | 0 | 0 | 0 |
| kb743120.1_18164_mature | kb743120.1_18164_mature | cagtcactactgcactgtcacc | 0 | 2 | 3 | 1 | 2 | 0 | 3 | 0 |
| kb744435.1_16038_star | kb744435.1_16038_star | ttagaaatggcaggctgagta | 0 | 0 | 1 | 2 | 0 | 0 | 0 | 0 |
| kb742736.1_12000_mature | kb742736.1_12000_mature | aaactgagcgtgctgaaaatga | 1 | 1 | 2 | 3 | 1 | 1 | 1 | 0 |
| kb744360.1_23720_star | kb744360.1_23720_star | tgctggggagggacagggacagg | 0 | 3 | 2 | 1 | 0 | 1 | 1 | 1 |
| kb742489.1_7302_star | kb742489.1_7302_star | ctttttgtcatgttatgcagtgaact | 0 | 4 | 1 | 0 | 0 | 0 | 1 | 0 |
| kb743197.1_1133_mature | kb743197.1_1133_mature | atggaacaacaggaatag | 0 | 0 | 4 | 0 | 0 | 0 | 3 | 0 |
| kb743076.1_7940_mature | kb743076.1_7940_mature | acacaaagcttgaacta | 0 | 0 | 0 | 1 | 0 | 0 | 2 | 0 |
| kb742762.1_19494_mature | kb742762.1_19494_mature | tggaagaacttttggagaa | 1 | 10 | 4 | 0 | 1 | 1 | 17 | 0 |
| kb742553.1_13404_mature | kb742553.1_13404_mature | tgtgctaaggtctcttgaag | 1 | 1 | 1 | 0 | 1 | 1 | 2 | 3 |
| kb742772.1_19001_star | kb742772.1_19001_star | agtttctaactaaatgaag | 0 | 0 | 2 | 0 | 0 | 0 | 1 | 0 |
| kb742523.1_1927_mature | kb742523.1_1927_mature | agtacgtgctggacttgctgct | 1 | 9 | 6 | 5 | 5 | 5 | 5 | 2 |
| kb743220.1_18067_mature | kb743220.1_18067_mature | tttctgtgtggaatttga | 1 | 19 | 6 | 5 | 2 | 0 | 9 | 1 |
| kb742907.1_4760_star | kb742907.1_4760_star | gatcctcaagtccttttcat | 0 | 1 | 6 | 1 | 2 | 0 | 4 | 1 |
| kb742395.1_8096_mature | kb742395.1_8096_mature | tggctgtgtggatcttaacccc | 0 | 1 | 0 | 6 | 3 | 1 | 3 | 0 |
| kb743509.1_7197_mature | kb743509.1_7197_mature | ttttagcatgtggggaag | 0 | 5 | 0 | 0 | 0 | 0 | 0 | 0 |
| kb742418.1_2461_star | kb742418.1_2461_star | aatgaggaaggacaacgtacat | 4 | 10 | 6 | 2 | 1 | 1 | 0 | 0 |
| kb742438.1_21875_star | kb742438.1_21875_star | ctataagagtcagactatttaggta | 3 | 18 | 0 | 33 | 24 | 0 | 3 | 2 |
| kb788489.1_25797_star | kb788489.1_25797_star | ccacatttcaactaatttaatagcctg | 0 | 0 | 0 | 0 | 0 | 0 | 0 | 2 |
| kb742878.1_11098_star | kb742878.1_11098_star | acctaagttacacggtaagccg | 0 | 0 | 0 | 0 | 0 | 0 | 1 | 0 |
| kb744221.1_21790_star | kb744221.1_21790_star | taagtgttgcccaaagttctcc | 0 | 2 | 1 | 0 | 0 | 1 | 0 | 0 |
| kb743899.1_20956_mature | kb743899.1_20956_mature | aggatttggatttgtttg | 0 | 6 | 1 | 2 | 1 | 1 | 3 | 0 |
| kb745238.1_19989_star | kb745238.1_19989_star | catattgtatgaagcaatgaca | 4 | 0 | 0 | 2 | 0 | 1 | 0 | 0 |
| kb742899.1_7094_star | kb742899.1_7094_star | ggcacagtttttgtca | 0 | 1 | 1 | 0 | 0 | 1 | 1 | 0 |
| kb742524.1_4612_mature | kb742524.1_4612_mature | aacttcttgaatgctgaca | 4 | 137 | 0 | 116 | 24 | 4 | 5 | 4 |
| kb744524.1_22317_star | kb744524.1_22317_star | atttttgattgcatccctg | 0 | 1 | 0 | 0 | 0 | 0 | 0 | 0 |
| kb742933.1_16265_mature | kb742933.1_16265_mature | tggctgtaacttggcatgtt | 0 | 0 | 1 | 2 | 0 | 0 | 6 | 1 |
| kb742442.1_13514_mature | kb742442.1_13514_mature | ttggagtgtagttgttccc | 6 | 18 | 5 | 16 | 6 | 10 | 11 | 7 |
| kb742778.1_14666_star | kb742778.1_14666_star | tagcagttttgtttttt | 1 | 2 | 0 | 0 | 0 | 1 | 0 | 0 |
| kb742907.1_4743_mature | kb742907.1_4743_mature | cagtgctgaccagaattcaggta | 0 | 3 | 0 | 1 | 0 | 0 | 5 | 1 |
| kb742435.1_5941_mature | kb742435.1_5941_mature | tctgttgtatataccagtgaac | 8 | 13 | 9 | 6 | 7 | 5 | 11 | 7 |
| kb742900.1_11376_star | kb742900.1_11376_star | tcagagctttcagtgcaagaacc | 0 | 2 | 1 | 2 | 0 | 0 | 1 | 4 |
| kb742714.1_4176_star | kb742714.1_4176_star | gattcccaaattgccagttgaaata | 0 | 0 | 0 | 2 | 0 | 0 | 0 | 2 |
| kb742736.1_11989_star | kb742736.1_11989_star | agggatcgtttccacctcacggg | 0 | 3 | 0 | 1 | 0 | 0 | 0 | 0 |
| kb744160.1_15427_star | kb744160.1_15427_star | caggtcatggctccttcct | 0 | 0 | 0 | 0 | 1 | 1 | 0 | 1 |
| kb742389.1_11430_star | kb742389.1_11430_star | aggagcttgttttctttctttgc | 0 | 3 | 2 | 0 | 0 | 0 | 1 | 1 |
| kb743650.1_19110_mature | kb743650.1_19110_mature | atctgttagtgattcttc | 0 | 4 | 3 | 3 | 0 | 1 | 4 | 0 |
| kb745974.1_23663_star | kb745974.1_23663_star | ctcacccgtccctcccc | 0 | 0 | 0 | 0 | 0 | 0 | 1 | 0 |
| kb809831.1_25349_mature | kb809831.1_25349_mature | tgccatttctcttgtcccagcagg | 1 | 6 | 4 | 7 | 1 | 1 | 4 | 1 |
| kb742832.1_11524_star | kb742832.1_11524_star | gcgtggggtaggggggct | 0 | 1 | 0 | 0 | 0 | 1 | 1 | 0 |
| kb742618.1_11465_star | kb742618.1_11465_star | tggaaagaaagggc | 1 | 0 | 0 | 0 | 0 | 0 | 0 | 0 |
| kb743525.1_10182_mature | kb743525.1_10182_mature | aaaagccatttctgagacacagt | 8 | 11 | 6 | 7 | 7 | 1 | 14 | 5 |
| kb742427.1_18768_star | kb742427.1_18768_star | agccttcggcctcctcct | 2 | 5 | 2 | 1 | 1 | 5 | 7 | 2 |
| kb742650.1_8472_mature | kb742650.1_8472_mature | atggataaaggcagcaagtagctct | 0 | 4 | 5 | 0 | 1 | 0 | 2 | 0 |
| kb744079.1_21184_star | kb744079.1_21184_star | caggtgcaggaggcgttttat | 0 | 1 | 0 | 1 | 0 | 0 | 0 | 0 |
| kb746632.1_16654_mature | kb746632.1_16654_mature | gtgactagtcgcaggagct | 0 | 0 | 0 | 0 | 3 | 0 | 0 | 0 |
| kb743364.1_9935_star | kb743364.1_9935_star | ttctttttcctgccttgat | 0 | 0 | 2 | 0 | 0 | 0 | 0 | 1 |
| kb743581.1_20313_mature | kb743581.1_20313_mature | acgtggaccagctcgagtacg | 0 | 1 | 2 | 1 | 0 | 0 | 2 | 2 |
| kb743257.1_4665_mature | kb743257.1_4665_mature | tcttttttgactgtgtgtg | 2 | 2 | 2 | 1 | 1 | 0 | 8 | 3 |
| kb743474.1_21446_star | kb743474.1_21446_star | tcactgttctcttactg | 1 | 8 | 0 | 1 | 0 | 0 | 0 | 0 |
| kb743547.1_18204_star | kb743547.1_18204_star | ctcaccgactcggcggcggc | 2 | 1 | 1 | 0 | 0 | 0 | 2 | 2 |
| kb742588.1_6980_mature | kb742588.1_6980_mature | aaaagggctggagctgctcctct | 2 | 10 | 17 | 6 | 7 | 2 | 4 | 3 |
| kb742635.1_16101_mature | kb742635.1_16101_mature | tcagaaggctgcgtgttc | 3 | 15 | 26 | 14 | 14 | 5 | 39 | 88 |
| kb743374.1_21335_star | kb743374.1_21335_star | tgggagtcatagaatcataga | 0 | 0 | 4 | 2 | 0 | 1 | 1 | 0 |
| kb743226.1_4916_mature | kb743226.1_4916_mature | agggccgaggtctggggtgatt | 2 | 3 | 4 | 2 | 0 | 0 | 3 | 2 |
| kb744955.1_13835_mature | kb744955.1_13835_mature | accagagtaaccagagta | 1 | 11 | 9 | 6 | 1 | 3 | 2 | 5 |
| kb742469.1_19787_star | kb742469.1_19787_star | caactcaggatattcggtgattct | 1 | 23 | 2 | 17 | 4 | 2 | 2 | 0 |
| kb742432.1_463_star | kb742432.1_463_star | caggtcaacagtagaattgaat | 2 | 3 | 0 | 1 | 2 | 0 | 0 | 0 |
| kb742563.1_12897_star | kb742563.1_12897_star | tcgaatctggggtctgt | 0 | 2 | 2 | 0 | 0 | 0 | 1 | 0 |
| kb742678.1_15758_mature | kb742678.1_15758_mature | cgttactgtatatttcttagtgc | 0 | 3 | 0 | 0 | 0 | 0 | 5 | 0 |
| kb743912.1_17128_star | kb743912.1_17128_star | attttctagatgctat | 0 | 1 | 0 | 0 | 0 | 0 | 0 | 0 |
| kb746737.1_23829_mature | kb746737.1_23829_mature | aacatagaaacaatgtgggacc | 0 | 2 | 4 | 1 | 0 | 0 | 1 | 0 |
| kb743391.1_16599_star | kb743391.1_16599_star | ccaaattcctgagttttcctaca | 0 | 1 | 1 | 8 | 7 | 0 | 3 | 0 |
| kb743110.1_6038_star | kb743110.1_6038_star | gagtctgcacagcccattagtg | 0 | 7 | 1 | 4 | 2 | 2 | 1 | 1 |
| kb742395.1_8063_mature | kb742395.1_8063_mature | atttgtaaaggctgttgat | 0 | 2 | 3 | 0 | 1 | 1 | 0 | 0 |
| kb744519.1_22650_star | kb744519.1_22650_star | aatgtacagcatggctgtga | 0 | 0 | 0 | 0 | 1 | 0 | 0 | 1 |
| kb745306.1_22118_star | kb745306.1_22118_star | aagattacagcatcttgta | 45 | 36 | 28 | 28 | 5 | 69 | 22 | 40 |
| kb743876.1_25298_star | kb743876.1_25298_star | ctggatcactaatttgatactttaa | 5 | 2 | 1 | 4 | 2 | 7 | 20 | 1 |
| kb743616.1_22008_mature | kb743616.1_22008_mature | ttcaaaagccacctgga | 0 | 0 | 0 | 4 | 0 | 0 | 1 | 0 |
| kb742466.1_4845_star | kb742466.1_4845_star | ttacaataaacatttttaaataaga | 0 | 0 | 1 | 0 | 0 | 0 | 1 | 0 |
| kb745020.1_18404_star | kb745020.1_18404_star | ctgttgtgggtcatctgtg | 0 | 0 | 0 | 7 | 1 | 0 | 0 | 0 |
| kb744077.1_12523_mature | kb744077.1_12523_mature | aaatgcttgaactactgtacaggt | 0 | 4 | 7 | 0 | 2 | 2 | 4 | 4 |
| kb742963.1_10701_mature | kb742963.1_10701_mature | tgtgtgctgagaatctcaaaca | 0 | 12 | 7 | 3 | 1 | 2 | 10 | 1 |
| kb746255.1_23677_mature | kb746255.1_23677_mature | aaacctggtagaatgtttgag | 1 | 3 | 3 | 1 | 3 | 2 | 2 | 1 |
| kb742659.1_3592_star | kb742659.1_3592_star | cgctggggttttgtcggtg | 0 | 0 | 0 | 1 | 0 | 0 | 0 | 0 |
| kb742966.1_4459_star | kb742966.1_4459_star | atgccaattttagtgctgtagc | 2 | 3 | 2 | 4 | 2 | 4 | 8 | 1 |
| kb743171.1_10216_star | kb743171.1_10216_star | tacaaggtggtggcgctgcgctg | 0 | 3 | 4 | 0 | 0 | 1 | 0 | 1 |
| kb742499.1_8257_star | kb742499.1_8257_star | cttcgtgggagcttttgtctgtcagt | 1 | 4 | 0 | 5 | 7 | 0 | 2 | 1 |
| kb742595.1_8422_mature | kb742595.1_8422_mature | gtgggctgcagcttccctgc | 1 | 4 | 2 | 0 | 0 | 0 | 0 | 3 |
| kb742473.1_6391_star | kb742473.1_6391_star | ccaaaaattgttcttaatgccttc | 1 | 0 | 1 | 0 | 0 | 0 | 1 | 1 |
| kb744332.1_17321_star | kb744332.1_17321_star | ccaagtattcacttgccagt | 0 | 0 | 1 | 0 | 0 | 0 | 0 | 0 |
| kb742382.1_14244_mature | kb742382.1_14244_mature | gatttgtgctcttcctctgcag | 2 | 7 | 7 | 4 | 6 | 0 | 5 | 1 |
| kb743412.1_5060_star | kb743412.1_5060_star | gctttctccggcttgtccagc | 0 | 2 | 0 | 1 | 0 | 0 | 4 | 0 |
| kb742582.1_7351_star | kb742582.1_7351_star | gagagcaagccttagataagt | 0 | 7 | 0 | 4 | 0 | 0 | 0 | 0 |
| kb742791.1_9551_star | kb742791.1_9551_star | ttttgtgtttctacctagttaact | 0 | 2 | 2 | 2 | 0 | 2 | 2 | 0 |
| kb743120.1_18165_star | kb743120.1_18165_star | cagctgctagctgcactccata | 0 | 1 | 2 | 0 | 1 | 0 | 5 | 0 |
| kb743541.1_21773_star | kb743541.1_21773_star | tgggagggtacgggagcaggcgga | 0 | 0 | 1 | 1 | 0 | 0 | 0 | 0 |
| kb742880.1_6501_star | kb742880.1_6501_star | aacttgctatgttgtcagc | 0 | 1 | 0 | 0 | 0 | 3 | 0 | 1 |
| kb742451.1_6350_star | kb742451.1_6350_star | agtgctgacaacttgt | 0 | 0 | 1 | 0 | 0 | 0 | 0 | 0 |
| kb743858.1_24463_mature | kb743858.1_24463_mature | tacgagctgaccagaaatgttta | 1 | 1 | 1 | 17 | 4 | 0 | 1 | 1 |
| kb742482.1_16316_mature | kb742482.1_16316_mature | cctttctgtgttctcttt | 1 | 3 | 2 | 1 | 1 | 2 | 2 | 0 |
| kb742479.1_277_mature | kb742479.1_277_mature | aactgtgctgactgctgaggt | 2 | 9 | 7 | 4 | 4 | 4 | 4 | 0 |
| kb743210.1_15290_mature | kb743210.1_15290_mature | atgtataatggctggtgtgaactt | 0 | 5 | 6 | 1 | 2 | 2 | 2 | 3 |
| kb743004.1_8916_star | kb743004.1_8916_star | gtaagtttggttgcagca | 0 | 2 | 0 | 0 | 1 | 1 | 0 | 0 |
| kb742712.1_8599_star | kb742712.1_8599_star | aatgaaaacagttggagatgga | 0 | 2 | 0 | 0 | 1 | 0 | 2 | 1 |
| kb744160.1_15417_mature | kb744160.1_15417_mature | agttgtgtgcatctgaagat | 4 | 8 | 1 | 6 | 5 | 1 | 5 | 7 |
| kb743922.1_17818_mature | kb743922.1_17818_mature | ttttttctttaactgtagaaa | 2 | 2 | 3 | 4 | 1 | 0 | 4 | 3 |
| kb742580.1_2701_star | kb742580.1_2701_star | ttctgttttgtgaccctaaatc | 0 | 1 | 0 | 2 | 2 | 0 | 3 | 0 |
| kb742390.1_22076_star | kb742390.1_22076_star | tttcaaatcaccctgcaatt | 0 | 1 | 1 | 0 | 0 | 0 | 0 | 0 |
| kb743161.1_22926_mature | kb743161.1_22926_mature | ctctgctggagggttttgacatcc | 1 | 2 | 4 | 1 | 1 | 0 | 0 | 0 |
| kb742959.1_16107_mature | kb742959.1_16107_mature | tcggccccgtgtcaccgcgc | 0 | 1 | 0 | 0 | 0 | 1 | 1 | 0 |
| kb742595.1_8411_mature | kb742595.1_8411_mature | agctggatactctctgtg | 0 | 0 | 3 | 1 | 1 | 2 | 0 | 2 |
| kb742701.1_6478_mature | kb742701.1_6478_mature | aaaatgggctgatagaaaa | 4 | 0 | 1 | 1 | 0 | 0 | 0 | 1 |
| kb743609.1_4596_mature | kb743609.1_4596_mature | ttgtctgtgatctataacactgt | 3 | 2 | 6 | 3 | 0 | 7 | 2 | 0 |
| kb742652.1_6556_mature | kb742652.1_6556_mature | aatcgatccttgcagttgcagca | 0 | 0 | 6 | 1 | 0 | 0 | 2 | 1 |
| kb744232.1_13272_mature | kb744232.1_13272_mature | atgttcttcagtagcctga | 4 | 11 | 0 | 11 | 1 | 2 | 0 | 0 |
| kb742441.1_714_mature | kb742441.1_714_mature | gtgcgaggagtgtagctgca | 5 | 6 | 3 | 0 | 0 | 1 | 0 | 1 |
| kb742993.1_11354_star | kb742993.1_11354_star | gtaaccagtgtaaaagt | 0 | 0 | 0 | 0 | 1 | 1 | 0 | 0 |
| kb743509.1_7190_star | kb743509.1_7190_star | attttaagacactacagccta | 0 | 1 | 0 | 2 | 0 | 0 | 2 | 0 |
| kb742467.1_8804_star | kb742467.1_8804_star | acagaaaggtgcgctgttc | 0 | 0 | 0 | 2 | 0 | 0 | 0 | 0 |
| kb743364.1_9931_mature | kb743364.1_9931_mature | tgtcctggggtctgtgcgctgc | 1 | 5 | 6 | 0 | 2 | 0 | 2 | 2 |
| kb794474.1_25695_star | kb794474.1_25695_star | ccaagtattaggtattacaaggt | 0 | 0 | 0 | 1 | 0 | 1 | 0 | 0 |
| kb744204.1_11867_mature | kb744204.1_11867_mature | ttgattcctggttatactgtaact | 1 | 1 | 5 | 0 | 0 | 0 | 1 | 0 |
| kb742605.1_4571_star | kb742605.1_4571_star | aaaactagggacttgcttggt | 0 | 1 | 4 | 2 | 1 | 1 | 0 | 0 |
| kb807964.1_25406_star | kb807964.1_25406_star | cgagcagcgagagcgccccaaa | 0 | 0 | 1 | 0 | 0 | 0 | 0 | 1 |
| kb743037.1_11824_star | kb743037.1_11824_star | tgctgaggatgcaactag | 0 | 1 | 0 | 0 | 0 | 0 | 1 | 1 |
| kb745179.1_20931_star | kb745179.1_20931_star | gacctgctgccagagc | 0 | 3 | 1 | 1 | 0 | 0 | 0 | 1 |
| kb742659.1_3592_mature | kb742659.1_3592_mature | caagcggatccatggcgtg | 0 | 1 | 0 | 0 | 0 | 0 | 3 | 0 |
| kb742855.1_12689_star | kb742855.1_12689_star | agcaccacgggtggctctccagac | 0 | 4 | 3 | 2 | 0 | 1 | 2 | 1 |
| kb743302.1_19391_mature | kb743302.1_19391_mature | ggagatcatgtgtactgc | 0 | 3 | 5 | 0 | 0 | 2 | 2 | 4 |
| kb744156.1_10324_star | kb744156.1_10324_star | acgctgtcaagtcaatggagttct | 0 | 0 | 2 | 2 | 1 | 0 | 2 | 1 |
| kb743110.1_6039_mature | kb743110.1_6039_mature | atgtttgagagttgtatgt | 0 | 1 | 0 | 3 | 0 | 0 | 1 | 1 |
| kb742887.1_1483_star | kb742887.1_1483_star | actgcaagtaagagaa | 0 | 0 | 0 | 2 | 0 | 0 | 0 | 0 |
| kb744212.1_8548_star | kb744212.1_8548_star | ggtagcatcatgctccaaatgtct | 1 | 0 | 2 | 0 | 0 | 0 | 0 | 0 |
| kb742658.1_9170_mature | kb742658.1_9170_mature | caagcagagctgtgggtg | 0 | 4 | 0 | 39 | 3 | 0 | 4 | 5 |
| kb742808.1_145_mature | kb742808.1_145_mature | atgtttgtgttttctttg | 0 | 2 | 4 | 2 | 0 | 0 | 5 | 2 |
| kb742418.1_2475_star | kb742418.1_2475_star | cgtgtctggatattcccatgg | 0 | 0 | 2 | 2 | 0 | 0 | 0 | 0 |
| kb744243.1_15689_star | kb744243.1_15689_star | ttgtttagttccagcctggg | 0 | 0 | 1 | 0 | 0 | 0 | 0 | 2 |
| kb743553.1_21115_mature | kb743553.1_21115_mature | gagtgcagtagttctggat | 0 | 5 | 0 | 0 | 0 | 0 | 1 | 0 |
| kb742875.1_10959_mature | kb742875.1_10959_mature | taacttctcttttctgttctccaga | 2 | 7 | 4 | 1 | 4 | 2 | 7 | 1 |
| kb745040.1_20267_mature | kb745040.1_20267_mature | acagaaactggaacagcaatttttc | 13 | 65 | 2 | 35 | 11 | 3 | 5 | 1 |
| kb743323.1_3663_mature | kb743323.1_3663_mature | atccacacctctgcagggctgt | 2 | 5 | 4 | 5 | 0 | 1 | 5 | 2 |
| kb743057.1_15968_mature | kb743057.1_15968_mature | atgcactgctgagcccc | 0 | 0 | 0 | 0 | 0 | 1 | 0 | 2 |
| kb743277.1_20350_mature | kb743277.1_20350_mature | ggggcaggtggagcagc | 0 | 3 | 3 | 1 | 0 | 0 | 1 | 0 |
| kb742785.1_11595_star | kb742785.1_11595_star | gaggagagagtggtggttgtgtatcttgac | 1 | 3 | 4 | 3 | 2 | 0 | 0 | 0 |
| kb742845.1_5775_star | kb742845.1_5775_star | gtcaagtaagttccagatttg | 1 | 1 | 2 | 2 | 0 | 1 | 1 | 2 |
| kb742750.1_2175_mature | kb742750.1_2175_mature | aagtctggtgccagcagca | 3 | 12 | 5 | 0 | 4 | 0 | 3 | 1 |
| kb743728.1_10648_star | kb743728.1_10648_star | catggagcttgaaagaatcattcg | 0 | 1 | 0 | 0 | 0 | 0 | 0 | 1 |
| kb742737.1_21693_star | kb742737.1_21693_star | cctcctcccttggccaag | 1 | 0 | 0 | 0 | 0 | 0 | 0 | 0 |
| kb744553.1_17005_mature | kb744553.1_17005_mature | gagcattgttggctgatgtg | 1 | 0 | 0 | 1 | 3 | 1 | 0 | 5 |
| kb743473.1_22935_star | kb743473.1_22935_star | tgaccaaagccatc | 0 | 0 | 0 | 0 | 0 | 1 | 0 | 0 |
| kb742977.1_9640_mature | kb742977.1_9640_mature | tggtttctgtatctgcag | 0 | 4 | 3 | 3 | 0 | 1 | 1 | 1 |
| kb742802.1_7867_mature | kb742802.1_7867_mature | taaatcttttggctgtttcaga | 0 | 3 | 5 | 0 | 0 | 2 | 8 | 0 |
| kb745078.1_19191_star | kb745078.1_19191_star | gcctgttggagtttaag | 0 | 7 | 1 | 2 | 1 | 0 | 1 | 0 |
| kb743186.1_17591_star | kb743186.1_17591_star | tgagtcccctgcagcag | 0 | 1 | 0 | 0 | 0 | 0 | 1 | 1 |
| kb743459.1_15677_mature | kb743459.1_15677_mature | aaaagattggctgtttgcacct | 5 | 32 | 10 | 2 | 6 | 12 | 9 | 2 |
| kb742786.1_17315_mature | kb742786.1_17315_mature | cacttcttgaatgctgaca | 5 | 134 | 3 | 126 | 23 | 1 | 4 | 1 |
| kb743317.1_3307_mature | kb743317.1_3307_mature | ctagagacttcagagaact | 0 | 3 | 3 | 2 | 1 | 1 | 2 | 3 |
| kb744900.1_18356_mature | kb744900.1_18356_mature | attcagatgagaagaacgtatt | 3 | 3 | 13 | 2 | 0 | 0 | 3 | 0 |
| kb743194.1_20366_mature | kb743194.1_20366_mature | tcagagaactgtaaatgtggaaa | 1 | 1 | 2 | 0 | 3 | 0 | 9 | 0 |
| kb742677.1_7513_star | kb742677.1_7513_star | aaaccggcacgtccaagccctttc | 1 | 1 | 4 | 0 | 0 | 0 | 1 | 1 |
| kb742739.1_8235_mature | kb742739.1_8235_mature | ctaatcacaagggggttgttt | 71 | 498 | 4 | 463 | 66 | 5 | 15 | 5 |
| kb742957.1_4292_star | kb742957.1_4292_star | ggtaccagtgcccatttaaaagt | 0 | 1 | 1 | 2 | 0 | 0 | 0 | 0 |
| kb744520.1_22196_mature | kb744520.1_22196_mature | ttgctggaagttctcacc | 1 | 4 | 0 | 0 | 0 | 0 | 2 | 0 |
| kb747234.1_23193_mature | kb747234.1_23193_mature | gagcacaggctgagtggt | 2 | 19 | 18 | 3 | 2 | 3 | 8 | 4 |
| kb802624.1_25541_mature | kb802624.1_25541_mature | tgtgctcttgtagaaaaggaa | 1 | 4 | 3 | 2 | 7 | 2 | 1 | 0 |
| kb743556.1_17227_star | kb743556.1_17227_star | aaagatgggatggaaacctactgt | 1 | 4 | 0 | 1 | 1 | 1 | 6 | 1 |
| kb743412.1_5055_mature | kb743412.1_5055_mature | tgtggggttgtggaaacca | 3 | 14 | 2 | 12 | 2 | 1 | 5 | 5 |
| kb743307.1_13925_mature | kb743307.1_13925_mature | ctggtggggcagtactcc | 0 | 0 | 1 | 1 | 1 | 0 | 3 | 0 |
| kb742554.1_1770_mature | kb742554.1_1770_mature | gactgctgatagatgacg | 0 | 1 | 0 | 12 | 40 | 0 | 1 | 1 |
| kb746554.1_24396_star | kb746554.1_24396_star | tccagcctggagtttaagtctcttgt | 3 | 2 | 5 | 1 | 1 | 1 | 2 | 0 |
| kb744909.1_18946_mature | kb744909.1_18946_mature | acgagagctttgaaggc | 22 | 215 | 82 | 47 | 35 | 14 | 14 | 54 |
| kb742809.1_13891_star | kb742809.1_13891_star | gtggcagatgggtcgtg | 1 | 0 | 0 | 0 | 0 | 0 | 0 | 0 |
| kb742833.1_18_mature | kb742833.1_18_mature | aaagctgtttccatctgtgcaagt | 0 | 4 | 7 | 5 | 1 | 1 | 3 | 0 |
| kb745020.1_18404_mature | kb745020.1_18404_mature | taggacttgctcttcagta | 31 | 164 | 4 | 283 | 74 | 5 | 10 | 6 |
| kb748669.1_25372_star | kb748669.1_25372_star | tgtcatcaccctggcaatga | 0 | 0 | 0 | 1 | 2 | 0 | 0 | 0 |
| kb744306.1_3739_mature | kb744306.1_3739_mature | aagtggagtgtcttatgtaaata | 0 | 1 | 4 | 0 | 0 | 0 | 2 | 1 |
| kb742677.1_7513_mature | kb742677.1_7513_mature | tatggcattggactccggtttgg | 2 | 7 | 7 | 2 | 2 | 5 | 5 | 1 |
| kb744577.1_22379_star | kb744577.1_22379_star | tttgaagaaggaaaggagt | 2 | 3 | 2 | 1 | 0 | 0 | 1 | 1 |
| kb742937.1_7548_mature | kb742937.1_7548_mature | ctatggagctatgtgtagagctgct | 0 | 3 | 1 | 2 | 2 | 2 | 4 | 0 |
| kb743183.1_7711_star | kb743183.1_7711_star | agacaagtatgaaacatgggcaatg | 1 | 0 | 2 | 0 | 1 | 0 | 0 | 0 |
| kb743145.1_780_star | kb743145.1_780_star | ctttgcaaacagcctaatagg | 0 | 3 | 1 | 1 | 0 | 0 | 0 | 1 |
| kb742397.1_12275_star | kb742397.1_12275_star | gtgttagtgtgcacacgtca | 0 | 0 | 0 | 1 | 0 | 0 | 2 | 2 |
| kb743307.1_13940_mature | kb743307.1_13940_mature | agaagaaagctctagacctg | 0 | 0 | 0 | 0 | 2 | 1 | 2 | 4 |
| kb743520.1_3906_mature | kb743520.1_3906_mature | gtgtttaactctgaagagca | 0 | 1 | 2 | 5 | 1 | 0 | 4 | 0 |
| kb743067.1_21825_mature | kb743067.1_21825_mature | taaggatgttgcaaggatc | 5 | 7 | 1 | 9 | 1 | 1 | 1 | 0 |
| kb742616.1_12588_star | kb742616.1_12588_star | gcccggaacttctccacaaatgg | 0 | 0 | 3 | 1 | 1 | 0 | 1 | 0 |
| kb743046.1_19357_star | kb743046.1_19357_star | gtttttcaaaagcaatgatgg | 2 | 5 | 0 | 8 | 0 | 0 | 1 | 2 |
| kb743139.1_2203_mature | kb743139.1_2203_mature | aaaaatgaggatattctgatt | 0 | 6 | 5 | 1 | 0 | 0 | 2 | 1 |
| kb742967.1_10096_mature | kb742967.1_10096_mature | aatggagatagagaacatcgtt | 1 | 1 | 4 | 7 | 1 | 5 | 8 | 1 |
| kb742703.1_20193_star | kb742703.1_20193_star | tctttatatggtcacagccacg | 0 | 0 | 2 | 0 | 0 | 1 | 0 | 0 |
| kb742757.1_11061_mature | kb742757.1_11061_mature | tgcaggctcctgccagggctgt | 1 | 2 | 0 | 4 | 1 | 1 | 3 | 3 |
| kb744150.1_22612_star | kb744150.1_22612_star | tacatttccctgtcaaccagcactgc | 0 | 2 | 1 | 1 | 0 | 1 | 2 | 0 |
| kb744306.1_3739_star | kb744306.1_3739_star | tttcatagaataaaacatagct | 0 | 0 | 0 | 0 | 0 | 0 | 2 | 0 |
| kb743435.1_2984_star | kb743435.1_2984_star | aaaggcagtcaggagcctaatt | 0 | 0 | 1 | 0 | 0 | 0 | 1 | 1 |
| kb744145.1_18900_mature | kb744145.1_18900_mature | ctgcagtcaggagctcgggg | 1 | 0 | 0 | 0 | 3 | 2 | 0 | 3 |
| kb743584.1_13901_star | kb743584.1_13901_star | actttatacatttttttcctta | 1 | 0 | 2 | 1 | 0 | 2 | 1 | 0 |
| kb743357.1_14745_mature | kb743357.1_14745_mature | cggaaggatgtaccaggcatcga | 0 | 2 | 1 | 5 | 0 | 1 | 0 | 1 |
| kb742867.1_9028_star | kb742867.1_9028_star | gaggggggacctcgctgccactgt | 0 | 1 | 0 | 0 | 1 | 0 | 0 | 0 |
| kb743210.1_15329_mature | kb743210.1_15329_mature | aggatgttttagaattaaa | 1 | 2 | 0 | 1 | 0 | 0 | 1 | 2 |
| kb743090.1_9126_star | kb743090.1_9126_star | caacctgcagccagtc | 0 | 0 | 0 | 0 | 0 | 0 | 0 | 1 |
| kb744234.1_21737_star | kb744234.1_21737_star | gggaacggcctggagccctgcc | 1 | 2 | 1 | 1 | 0 | 1 | 3 | 1 |
| kb743260.1_3350_mature | kb743260.1_3350_mature | aaatgtcagctgaaacttgtg | 3 | 5 | 7 | 4 | 0 | 2 | 5 | 1 |
| kb744353.1_15005_star | kb744353.1_15005_star | gtgccacagggatctgggtgc | 2 | 0 | 1 | 3 | 0 | 0 | 0 | 1 |
| kb742791.1_9551_mature | kb742791.1_9551_mature | ctgacagcaggaagcagactcca | 2 | 0 | 0 | 0 | 2 | 0 | 4 | 3 |
| kb743108.1_15447_mature | kb743108.1_15447_mature | atgactgttttgttctgat | 8 | 3 | 1 | 5 | 1 | 2 | 3 | 0 |
| kb742582.1_7351_mature | kb742582.1_7351_mature | tgatttaagttatctctg | 1 | 0 | 3 | 0 | 0 | 0 | 0 | 0 |
| kb743487.1_17944_mature | kb743487.1_17944_mature | gtgtttacctggaggattttgta | 0 | 0 | 3 | 0 | 0 | 1 | 1 | 1 |
| kb742677.1_7524_mature | kb742677.1_7524_mature | tgttgtgtagagcccagtggaggt | 0 | 1 | 4 | 0 | 0 | 0 | 3 | 3 |
| kb744822.1_20008_star | kb744822.1_20008_star | tgatcaggagactcaagact | 1 | 3 | 5 | 2 | 0 | 0 | 5 | 0 |
| kb744558.1_15598_mature | kb744558.1_15598_mature | ttgtgtatcatgagctgg | 0 | 0 | 0 | 0 | 0 | 0 | 3 | 1 |
| kb742544.1_17748_star | kb742544.1_17748_star | tgggctgtgtccatccagcagt | 6 | 22 | 12 | 10 | 5 | 0 | 5 | 3 |
| kb743402.1_5886_mature | kb743402.1_5886_mature | actctggactttgaatcc | 218 | 1086 | 1440 | 442 | 298 | 126 | 316 | 1342 |
| kb742864.1_10343_star | kb742864.1_10343_star | gctacctgggcacacttctgg | 1 | 5 | 0 | 3 | 1 | 0 | 1 | 4 |
| kb742678.1_15747_star | kb742678.1_15747_star | aggacaaacgcactgtaaacac | 0 | 0 | 1 | 1 | 1 | 1 | 0 | 0 |
| kb743217.1_9768_star | kb743217.1_9768_star | tgaagagcctaattcacttaca | 2 | 1 | 3 | 1 | 0 | 2 | 0 | 0 |
| kb744638.1_20605_star | kb744638.1_20605_star | ctcaacacaggtttggtttctttg | 0 | 3 | 0 | 0 | 0 | 0 | 0 | 1 |
| kb742923.1_14524_mature | kb742923.1_14524_mature | gtttataaacctgtatgtactgtac | 2 | 36 | 1 | 40 | 10 | 2 | 4 | 1 |
| kb743214.1_8712_star | kb743214.1_8712_star | gtgaatatctagagcttgttt | 0 | 0 | 1 | 0 | 0 | 0 | 1 | 0 |
| kb743829.1_10880_star | kb743829.1_10880_star | caaggtgaacatcagactct | 0 | 1 | 1 | 3 | 0 | 1 | 2 | 0 |
| kb742840.1_6715_star | kb742840.1_6715_star | cgtcgctggggctgagnn | 1 | 0 | 4 | 1 | 0 | 0 | 0 | 1 |
| kb743493.1_18274_star | kb743493.1_18274_star | cactgctcctaatcacggggtcagcag | 0 | 0 | 2 | 3 | 0 | 0 | 1 | 1 |
| kb742734.1_7067_mature | kb742734.1_7067_mature | cccgagctgtgctatgag | 0 | 1 | 0 | 0 | 3 | 0 | 0 | 0 |
| kb746564.1_24909_mature | kb746564.1_24909_mature | aacggccgaaagcacgacagacacc | 32 | 155 | 5 | 143 | 34 | 3 | 7 | 1 |
| kb742629.1_525_star | kb742629.1_525_star | aaatgctgttgttgtgcagaga | 0 | 3 | 0 | 0 | 1 | 1 | 2 | 1 |
| kb742511.1_13656_star | kb742511.1_13656_star | tggtgcgtggacaaatacggcatgaa | 0 | 2 | 0 | 0 | 0 | 0 | 0 | 1 |
| kb742629.1_575_mature | kb742629.1_575_mature | aggacgtattggttggtttc | 0 | 1 | 0 | 4 | 0 | 0 | 0 | 1 |
| kb742404.1_2830_star | kb742404.1_2830_star | gtgctggtgcagccacccaggg | 1 | 0 | 2 | 1 | 2 | 0 | 0 | 2 |
| kb742701.1_6480_star | kb742701.1_6480_star | aggtgctgtatctctagagcag | 1 | 1 | 0 | 1 | 0 | 1 | 2 | 0 |
| kb742762.1_19494_star | kb742762.1_19494_star | tgggagaagctgcttcctgt | 0 | 0 | 2 | 1 | 2 | 0 | 0 | 0 |
| kb742928.1_10828_mature | kb742928.1_10828_mature | tttagcatgacgccagatggt | 83 | 470 | 0 | 533 | 85 | 5 | 25 | 9 |
| kb745003.1_21573_star | kb745003.1_21573_star | ctgtaatgctgttgattttg | 0 | 1 | 0 | 1 | 1 | 1 | 1 | 0 |
| kb742809.1_13889_star | kb742809.1_13889_star | tggctgacggccatcttcaggata | 1 | 1 | 1 | 0 | 1 | 0 | 0 | 1 |

| kb743175.1_22042_mature | kb743175.1_22042_mature | aggtgctgtgggagagct | 1 | 4 | 3 | 2 | 3 | 1 | 11 | 0 |
| --- | --- | --- | --- | --- | --- | --- | --- | --- | --- | --- |
| kb743111.1_4083_mature | kb743111.1_4083_mature | atggaaatggtgctctcgc | 4 | 19 | 4 | 506 | 2 | 3 | 10 | 2 |
| kb742605.1_4500_star | kb742605.1_4500_star | tgataatgcaactttgct | 0 | 0 | 0 | 0 | 0 | 1 | 2 | 0 |
| kb744198.1_12212_mature | kb744198.1_12212_mature | ttgttctccagatggtctcttgtt | 3 | 25 | 1 | 25 | 4 | 4 | 3 | 2 |
| kb742992.1_5466_star | kb742992.1_5466_star | taggacgagatgaatcatgtaac | 5 | 3 | 6 | 6 | 1 | 2 | 6 | 1 |
| kb746368.1_21805_star | kb746368.1_21805_star | ttttgttgttgtagga | 0 | 0 | 0 | 1 | 0 | 1 | 1 | 1 |
| kb742757.1_11049_star | kb742757.1_11049_star | tacacccttgtcattgcttttgt | 1 | 0 | 0 | 1 | 0 | 0 | 0 | 0 |
| kb746345.1_23788_star | kb746345.1_23788_star | aaaaaagaaacagctttgaccgaagcga | 0 | 3 | 6 | 0 | 1 | 1 | 5 | 1 |
| kb742697.1_16950_star | kb742697.1_16950_star | caggatatgggcagttttcag | 0 | 1 | 1 | 0 | 0 | 0 | 1 | 0 |
| kb742454.1_21092_star | kb742454.1_21092_star | atacagctttggttgaaa | 0 | 0 | 0 | 0 | 1 | 0 | 0 | 0 |
| kb744088.1_3992_mature | kb744088.1_3992_mature | tgattagcagaagtttga | 1 | 1 | 1 | 0 | 0 | 0 | 3 | 0 |
| kb745078.1_19191_mature | kb745078.1_19191_mature | tggactgtgctcttggtca | 280 | 6237 | 56 | 5304 | 118 | 36 | 151 | 91 |
| kb744332.1_17329_mature | kb744332.1_17329_mature | tttctgcaggactctgtg | 23 | 60 | 46 | 43 | 28 | 16 | 25 | 12 |
| kb742785.1_11593_star | kb742785.1_11593_star | tggggttgttttcttgcagtt | 31 | 84 | 1 | 174 | 19 | 6 | 7 | 6 |
| kb744709.1_24284_mature | kb744709.1_24284_mature | gaaatgcaacaaggactttgc | 7 | 0 | 20 | 0 | 2 | 0 | 0 | 1 |
| kb742520.1_11482_star | kb742520.1_11482_star | tgcagtttctagttccgaagag | 0 | 2 | 0 | 1 | 1 | 0 | 0 | 0 |
| kb743158.1_2920_star | kb743158.1_2920_star | tctttttctgccttgtactat | 0 | 1 | 0 | 2 | 0 | 0 | 1 | 0 |
| kb742531.1_10727_mature | kb742531.1_10727_mature | ctcgggctgaagtcagatc | 0 | 0 | 0 | 3 | 2 | 0 | 1 | 0 |
| kb745336.1_21549_mature | kb745336.1_21549_mature | ttcttcccttactaaaca | 12 | 51 | 1 | 72 | 4 | 1 | 0 | 1 |
| kb744395.1_20615_star | kb744395.1_20615_star | tttgtgacctctcaggtgcccgac | 0 | 2 | 2 | 0 | 1 | 2 | 2 | 0 |
| kb742441.1_714_star | kb742441.1_714_star | tgcaccacccctcgtctgt | 0 | 2 | 2 | 4 | 0 | 0 | 0 | 0 |
| kb743153.1_6790_mature | kb743153.1_6790_mature | aattacagttgggactgta | 0 | 0 | 3 | 1 | 2 | 0 | 2 | 1 |
| kb743600.1_21293_mature | kb743600.1_21293_mature | tgtgacatctctgcaggactgt | 1 | 2 | 3 | 1 | 4 | 2 | 3 | 0 |
| kb742899.1_7120_star | kb742899.1_7120_star | gcgggcaaactacctggctcccttt | 0 | 0 | 3 | 0 | 0 | 1 | 0 | 1 |
| kb744345.1_17158_mature | kb744345.1_17158_mature | tggctgttgatgctttttctgc | 3 | 3 | 2 | 6 | 4 | 0 | 0 | 0 |
| kb742969.1_1034_mature | kb742969.1_1034_mature | agtgatgggactagaaacttgg | 1 | 1 | 1 | 2 | 0 | 0 | 4 | 5 |
| kb742809.1_13885_mature | kb742809.1_13885_mature | ccgatgtggatgtgaatct | 1 | 10 | 9 | 1 | 2 | 2 | 4 | 2 |
| kb742446.1_12980_mature | kb742446.1_12980_mature | agtgcttttggagaaggtgaaca | 1 | 4 | 5 | 1 | 1 | 1 | 2 | 5 |
| kb742471.1_426_star | kb742471.1_426_star | tgggagcctcattgggaac | 0 | 0 | 1 | 0 | 0 | 1 | 0 | 0 |
| kb742386.1_9876_star | kb742386.1_9876_star | gacaaagctgagacctggcaaca | 10 | 33 | 88 | 18 | 34 | 19 | 5 | 23 |
| kb742830.1_16494_star | kb742830.1_16494_star | tttttctctcctacagtc | 0 | 9 | 0 | 2 | 1 | 1 | 0 | 0 |
| kb751569.1_24640_mature | kb751569.1_24640_mature | ttgtaggacttgttctcc | 8 | 85 | 5 | 80 | 29 | 8 | 7 | 4 |
| kb743005.1_3003_mature | kb743005.1_3003_mature | tctattgtgggatgcact | 0 | 0 | 0 | 1 | 0 | 0 | 2 | 0 |
| kb743518.1_15375_star | kb743518.1_15375_star | tagaaggcaacagtgaaaagag | 0 | 5 | 0 | 0 | 1 | 0 | 0 | 0 |
| kb742712.1_8596_mature | kb742712.1_8596_mature | ctgcaggtgaactcgggc | 0 | 0 | 2 | 1 | 2 | 2 | 1 | 1 |
| kb745441.1_24444_mature | kb745441.1_24444_mature | tcttgtccgattttagctatttgtt | 10 | 35 | 5 | 101 | 3 | 6 | 9 | 3 |
| kb743789.1_20582_mature | kb743789.1_20582_mature | atactggatattctaatgaat | 0 | 1 | 4 | 1 | 0 | 0 | 0 | 0 |
| kb744656.1_20733_star | kb744656.1_20733_star | ggagatgaggttggtcaggcaggact | 0 | 3 | 2 | 0 | 1 | 1 | 1 | 0 |
| kb743226.1_4867_mature | kb743226.1_4867_mature | ttcacgtctgctggaatgg | 0 | 3 | 0 | 2 | 0 | 0 | 1 | 1 |
| kb743297.1_21460_star | kb743297.1_21460_star | gtaaatatcccaaaccatg | 0 | 0 | 0 | 0 | 0 | 0 | 1 | 1 |
| kb742884.1_16504_mature | kb742884.1_16504_mature | gaggactaagctaaaaacc | 0 | 1 | 2 | 3 | 0 | 1 | 2 | 0 |
| kb742989.1_22227_star | kb742989.1_22227_star | tttacatttatccaaa | 2 | 0 | 0 | 0 | 0 | 0 | 0 | 0 |
| kb742922.1_21241_star | kb742922.1_21241_star | tcggatcacttccttgt | 0 | 0 | 2 | 0 | 0 | 0 | 1 | 1 |
| kb743525.1_10176_star | kb743525.1_10176_star | tgattttttgcggaaagtagg | 0 | 0 | 0 | 0 | 0 | 0 | 1 | 0 |
| kb742811.1_13117_mature | kb742811.1_13117_mature | ctccctgtgggctgagtg | 0 | 2 | 5 | 3 | 3 | 0 | 0 | 1 |
| kb743580.1_14137_star | kb743580.1_14137_star | gccttcacctctcttca | 0 | 4 | 0 | 0 | 0 | 0 | 0 | 1 |
| kb742523.1_1936_star | kb742523.1_1936_star | aacagcctgagtgaagggct | 0 | 2 | 1 | 0 | 0 | 0 | 0 | 0 |
| kb743374.1_21337_star | kb743374.1_21337_star | agcaacagcgaggtga | 0 | 1 | 0 | 0 | 0 | 0 | 1 | 0 |
| kb743167.1_13294_mature | kb743167.1_13294_mature | agagatgatgactacctgatc | 2 | 2 | 2 | 1 | 2 | 0 | 4 | 1 |
| kb743110.1_6005_mature | kb743110.1_6005_mature | atttggatttgaatgacc | 1 | 1 | 1 | 1 | 1 | 0 | 1 | 2 |
| kb742693.1_8755_mature | kb742693.1_8755_mature | gctctggggctctgtgaccc | 2 | 8 | 2 | 2 | 1 | 2 | 11 | 1 |
| kb742503.1_11004_mature | kb742503.1_11004_mature | aaatgagaactttgaggag | 3 | 8 | 5 | 4 | 1 | 5 | 6 | 3 |
| kb742585.1_9428_mature | kb742585.1_9428_mature | aaggacatgaagctgttggagagt | 20 | 275 | 6 | 377 | 40 | 8 | 27 | 16 |
| kb742595.1_8384_star | kb742595.1_8384_star | tgactaaagctacctaatggcaaccca | 0 | 0 | 0 | 0 | 0 | 0 | 1 | 0 |
| kb743625.1_7618_mature | kb743625.1_7618_mature | ctgtgttcctccttccatcattg | 1 | 5 | 0 | 0 | 2 | 0 | 1 | 0 |
| kb742588.1_6984_mature | kb742588.1_6984_mature | agcctaggcctggactcagagca | 0 | 3 | 3 | 2 | 0 | 1 | 3 | 0 |
| kb743547.1_18206_mature | kb743547.1_18206_mature | agagagcggtgatttttt | 0 | 3 | 1 | 4 | 0 | 0 | 0 | 1 |
| kb742497.1_4716_star | kb742497.1_4716_star | tcaagtctgtttccacaa | 0 | 2 | 2 | 0 | 0 | 2 | 0 | 0 |
| kb743896.1_6948_mature | kb743896.1_6948_mature | caggaggtgtgactgtaggaa | 1 | 2 | 21 | 2 | 3 | 6 | 0 | 4 |
| kb818614.1_24936_mature | kb818614.1_24936_mature | attatcttcctggagactctcaga | 1 | 5 | 6 | 5 | 0 | 0 | 0 | 0 |
| kb742703.1_20193_mature | kb742703.1_20193_mature | tggtgaagtcatctgaattgc | 3 | 0 | 1 | 0 | 1 | 0 | 1 | 1 |
| kb742392.1_20512_mature | kb742392.1_20512_mature | tggtggtgctacttccctgtgaagt | 2 | 17 | 3 | 16 | 3 | 1 | 1 | 2 |
| kb744077.1_12500_star | kb744077.1_12500_star | ctcatttttggttcacagctg | 0 | 1 | 0 | 1 | 0 | 1 | 4 | 0 |
| kb742803.1_17885_star | kb742803.1_17885_star | cagtggaacaagtggaattggcactgga | 0 | 0 | 0 | 2 | 1 | 2 | 3 | 0 |
| kb742959.1_16107_star | kb742959.1_16107_star | gcggtgccgcggggctttgt | 0 | 2 | 1 | 1 | 1 | 0 | 1 | 2 |
| kb742639.1_10610_mature | kb742639.1_10610_mature | tccggatccggcttctgagatga | 207 | 611 | 430 | 251 | 477 | 155 | 270 | 159 |
| kb819533.1_24845_mature | kb819533.1_24845_mature | atggtttactgggacctgcatc | 2 | 4 | 4 | 2 | 1 | 3 | 0 | 0 |
| kb742617.1_6191_mature | kb742617.1_6191_mature | attagaagatctgtgaa | 0 | 8 | 0 | 6 | 3 | 1 | 9 | 3 |
| kb742464.1_2384_star | kb742464.1_2384_star | tggaggaaaccaaactctatc | 1 | 4 | 1 | 2 | 1 | 0 | 0 | 0 |
| kb743720.1_23419_mature | kb743720.1_23419_mature | cacgagtacaagcactga | 0 | 2 | 0 | 1 | 1 | 0 | 0 | 0 |
| kb743518.1_15368_mature | kb743518.1_15368_mature | ttgatctgtcagagtaccgagac | 0 | 9 | 1 | 4 | 2 | 4 | 14 | 1 |
| kb743686.1_3389_star | kb743686.1_3389_star | tggagggacagaaatcagggactgg | 0 | 2 | 3 | 2 | 0 | 0 | 3 | 2 |
| kb744462.1_23614_mature | kb744462.1_23614_mature | ccagtcctggtgcatgaggagc | 16 | 47 | 53 | 50 | 33 | 30 | 25 | 20 |
| kb742629.1_532_star | kb742629.1_532_star | tacgtggatagccgtttag | 0 | 0 | 0 | 1 | 0 | 0 | 0 | 0 |
| kb743702.1_18437_star | kb743702.1_18437_star | ttgtaatctgtaatttggaaat | 1 | 4 | 1 | 2 | 0 | 2 | 3 | 2 |
| kb746316.1_19650_mature | kb746316.1_19650_mature | tgaggctgtagcagcacgctca | 1 | 2 | 2 | 0 | 0 | 0 | 2 | 1 |
| kb742579.1_8731_star | kb742579.1_8731_star | tgaccttggtccaccaga | 0 | 4 | 0 | 4 | 0 | 0 | 0 | 1 |
| kb743662.1_7528_mature | kb743662.1_7528_mature | gtaatagcatgtgctctgatc | 0 | 9 | 5 | 9 | 7 | 1 | 0 | 2 |
| kb742824.1_11561_mature | kb742824.1_11561_mature | tgatactgtgtgtgaaacctaaa | 0 | 7 | 4 | 3 | 1 | 1 | 9 | 0 |
| kb744237.1_17842_mature | kb744237.1_17842_mature | agtttaggctgtgtctttgct | 1 | 2 | 4 | 3 | 4 | 2 | 5 | 4 |
| kb742565.1_11788_star | kb742565.1_11788_star | gagcagttgtactcagcgtg | 0 | 0 | 1 | 0 | 0 | 0 | 0 | 0 |
| kb743188.1_21313_star | kb743188.1_21313_star | ggtcacagccctgagtctgagga | 0 | 4 | 0 | 2 | 0 | 0 | 3 | 0 |
| kb742807.1_16446_star | kb742807.1_16446_star | ggctttggttgggtctgctccagg | 0 | 8 | 3 | 1 | 2 | 0 | 0 | 1 |
| kb742643.1_14385_star | kb742643.1_14385_star | aggagcaattcacaga | 0 | 0 | 0 | 0 | 0 | 0 | 2 | 0 |
| kb742772.1_19002_mature | kb742772.1_19002_mature | tcttgaggagaatgctgt | 16 | 82 | 1 | 134 | 5 | 0 | 5 | 6 |
| kb742554.1_1770_star | kb742554.1_1770_star | acatctgccagctctctc | 2 | 4 | 1 | 4 | 1 | 1 | 0 | 0 |
| kb742605.1_4544_star | kb742605.1_4544_star | tcactgtagtgtgctggg | 0 | 0 | 0 | 2 | 0 | 0 | 1 | 1 |
| kb743183.1_7713_mature | kb743183.1_7713_mature | tgctgactgggcctgatcgct | 305 | 1012 | 5 | 329 | 125 | 3 | 28 | 4 |
| kb742439.1_3164_mature | kb742439.1_3164_mature | tagtgatttggacaatgcc | 63 | 288 | 2 | 497 | 19 | 0 | 1 | 3 |
| kb743072.1_9517_star | kb743072.1_9517_star | tggaatgatgaactgagaagtacct | 1 | 3 | 4 | 4 | 3 | 4 | 5 | 1 |
| kb743713.1_6756_mature | kb743713.1_6756_mature | agaactggaagctgaat | 1 | 4 | 1 | 0 | 2 | 0 | 3 | 1 |
| kb742651.1_5123_mature | kb742651.1_5123_mature | cgtatttgatgttgtaggtc | 0 | 1 | 4 | 0 | 0 | 0 | 2 | 0 |
| kb742616.1_12585_star | kb742616.1_12585_star | gacagaagaggagtcgaaatgtatg | 0 | 3 | 1 | 0 | 0 | 0 | 0 | 0 |
| kb742622.1_3805_mature | kb742622.1_3805_mature | ttgcactgtatggtatctgcaccc | 27 | 8 | 26 | 24 | 19 | 6 | 125 | 17 |
| kb742984.1_1913_mature | kb742984.1_1913_mature | tttcagggcaaagtgaaaggaa | 1 | 0 | 2 | 2 | 0 | 0 | 0 | 0 |
| kb743686.1_3399_star | kb743686.1_3399_star | ctttcataccttaccatgtatggt | 0 | 2 | 2 | 0 | 0 | 0 | 0 | 0 |
| kb742866.1_14616_star | kb742866.1_14616_star | atcagcagaagtgaaatttctgttgc | 0 | 1 | 2 | 4 | 0 | 2 | 1 | 1 |
| kb742798.1_12572_star | kb742798.1_12572_star | aatatggaaccaggcatt | 0 | 0 | 0 | 1 | 0 | 0 | 0 | 0 |
| kb744088.1_3983_mature | kb744088.1_3983_mature | atttcagtgatctgttggagaact | 2 | 8 | 6 | 6 | 1 | 2 | 3 | 0 |
| kb743833.1_10824_mature | kb743833.1_10824_mature | gtaatcgtctgggacggg | 1 | 0 | 0 | 0 | 0 | 4 | 0 | 0 |
| kb742974.1_23116_star | kb742974.1_23116_star | agggtcctgtcatcaaaaagtatataaa | 0 | 0 | 4 | 0 | 0 | 0 | 2 | 0 |
| kb742671.1_7159_star | kb742671.1_7159_star | caagtataggaaggtgtgca | 0 | 1 | 0 | 0 | 0 | 0 | 0 | 0 |
| kb743545.1_16427_mature | kb743545.1_16427_mature | tcttgtgtgaaacactgtccacc | 550 | 1358 | 28 | 5730 | 960 | 49 | 322 | 206 |
| kb810642.1_25334_star | kb810642.1_25334_star | catatttggtgaggatgatgagc | 2 | 14 | 1 | 25 | 10 | 0 | 1 | 1 |
| kb743963.1_19072_star | kb743963.1_19072_star | aggtttcttcctgggtttcaggag | 0 | 0 | 0 | 0 | 0 | 0 | 2 | 0 |
| kb743829.1_10876_mature | kb743829.1_10876_mature | cacccggaggtccctttctgc | 0 | 0 | 3 | 0 | 0 | 0 | 1 | 1 |
| kb742876.1_14062_star | kb742876.1_14062_star | ggggaaccaggtctgctaca | 0 | 0 | 1 | 0 | 0 | 0 | 0 | 1 |
| kb743037.1_11824_mature | kb743037.1_11824_mature | gcgtgctgtctcctgaga | 0 | 2 | 1 | 3 | 1 | 0 | 2 | 3 |
| kb743319.1_12031_mature | kb743319.1_12031_mature | gaactgtcatctttagacctgtgtg | 0 | 4 | 2 | 1 | 1 | 0 | 5 | 0 |
| kb742808.1_104_mature | kb742808.1_104_mature | tggtagttctttggaggctggc | 0 | 2 | 1 | 0 | 0 | 0 | 3 | 0 |
| kb742618.1_11465_mature | kb742618.1_11465_mature | tcttctatgattctgtg | 2 | 5 | 0 | 4 | 0 | 2 | 0 | 0 |
| kb742499.1_8266_star | kb742499.1_8266_star | accctgctgctagatgaggagagg | 0 | 4 | 3 | 0 | 1 | 0 | 0 | 1 |
| kb744033.1_9323_mature | kb744033.1_9323_mature | ctctgtgaaagctctgacagcag | 5 | 4 | 8 | 1 | 6 | 3 | 6 | 1 |
| kb758252.1_26056_star | kb758252.1_26056_star | ctgcccctcagggctccccg | 1 | 0 | 1 | 1 | 0 | 0 | 4 | 0 |
| kb742490.1_1386_star | kb742490.1_1386_star | atacactgcttttaggaattcactgccgg | 1 | 1 | 2 | 1 | 0 | 0 | 0 | 0 |
| kb742477.1_14364_mature | kb742477.1_14364_mature | actgggctggtgtgtgtatgtcc | 2 | 13 | 3 | 1 | 1 | 1 | 1 | 0 |
| kb742547.1_16706_star | kb742547.1_16706_star | actttattggtttgtttta | 0 | 0 | 0 | 2 | 0 | 0 | 0 | 0 |
| kb742887.1_1557_mature | kb742887.1_1557_mature | acccatgctggctgggcc | 6 | 4 | 0 | 9 | 4 | 0 | 4 | 1 |
| kb742438.1_21848_star | kb742438.1_21848_star | gcgatgctccatctgg | 0 | 0 | 0 | 0 | 0 | 0 | 0 | 1 |
| kb742660.1_11544_mature | kb742660.1_11544_mature | tgtctgttctcgtttgcccaga | 0 | 3 | 1 | 0 | 0 | 1 | 1 | 1 |
| kb743260.1_3328_star | kb743260.1_3328_star | cttcagactgcttcgcatg | 1 | 0 | 0 | 0 | 1 | 0 | 1 | 0 |
| kb744667.1_17789_mature | kb744667.1_17789_mature | tggatactctgactgagca | 0 | 4 | 1 | 0 | 1 | 0 | 4 | 0 |
| kb743145.1_828_mature | kb743145.1_828_mature | ctggagtttgctgaaga | 0 | 1 | 3 | 1 | 0 | 0 | 2 | 0 |
| kb743740.1_8890_mature | kb743740.1_8890_mature | agggtggggagggatggg | 0 | 1 | 6 | 1 | 4 | 0 | 0 | 0 |
| kb742513.1_2628_mature | kb742513.1_2628_mature | cacaaaatcagctgagatg | 0 | 0 | 0 | 0 | 0 | 0 | 5 | 0 |
| kb743435.1_2991_star | kb743435.1_2991_star | tgttcggcaaactcatcgtatct | 0 | 1 | 0 | 8 | 0 | 0 | 0 | 0 |
| kb742904.1_12941_mature | kb742904.1_12941_mature | atgactttgataaatgaaa | 0 | 5 | 2 | 3 | 0 | 1 | 3 | 0 |
| kb742728.1_18177_star | kb742728.1_18177_star | actgtcctggtttcagttagcaca | 1 | 6 | 2 | 13 | 4 | 1 | 1 | 0 |
| kb745238.1_20000_star | kb745238.1_20000_star | gtggtgagactcgggaaagtgacgta | 4 | 1 | 43 | 3 | 1 | 2 | 5 | 2 |
| kb743680.1_13567_mature | kb743680.1_13567_mature | gttattgatgtgtatct | 1 | 2 | 0 | 2 | 0 | 0 | 0 | 0 |
| kb742664.1_9858_star | kb742664.1_9858_star | tgtttcaacgcttcttac | 1 | 0 | 0 | 2 | 1 | 0 | 0 | 0 |
| kb743413.1_16014_mature | kb743413.1_16014_mature | cataggctgctgtgacctgat | 0 | 0 | 8 | 2 | 1 | 0 | 4 | 1 |
| kb743235.1_6577_star | kb743235.1_6577_star | gatgtggcctttgaaat | 0 | 0 | 1 | 0 | 2 | 1 | 2 | 0 |
| kb742655.1_5346_mature | kb742655.1_5346_mature | ctgcttcgatgctgggaca | 3 | 3 | 0 | 8 | 0 | 0 | 0 | 0 |
| kb742957.1_4306_mature | kb742957.1_4306_mature | caggtccttctctgcct | 1 | 22 | 1 | 15 | 7 | 1 | 1 | 0 |
| kb743748.1_19045_star | kb743748.1_19045_star | gcaggctgcaggtggcttccaaat | 0 | 3 | 5 | 2 | 3 | 0 | 2 | 1 |
| kb742907.1_4739_mature | kb742907.1_4739_mature | aaaaggatgtggagttgcc | 10 | 6 | 8 | 8 | 5 | 4 | 4 | 5 |
| kb743028.1_7250_star | kb743028.1_7250_star | tttctcagctgttgatg | 0 | 0 | 0 | 0 | 0 | 1 | 0 | 0 |
| kb742588.1_7036_mature | kb742588.1_7036_mature | aacgtgactttatcaagagcgatgc | 0 | 6 | 0 | 0 | 0 | 1 | 4 | 1 |
| kb742452.1_10576_star | kb742452.1_10576_star | gggctcggaggtcttcatcggg | 0 | 0 | 0 | 0 | 1 | 0 | 0 | 1 |
| kb743364.1_9931_star | kb743364.1_9931_star | tgtgcacagctcaccagcgagcgcc | 6 | 38 | 3 | 1 | 2 | 0 | 0 | 2 |
| kb742505.1_8515_mature | kb742505.1_8515_mature | tgaccagcaaggaagcaa | 60 | 49 | 50 | 62 | 41 | 39 | 85 | 49 |
| kb743412.1_5041_mature | kb743412.1_5041_mature | agaatactctgttagctttgt | 0 | 0 | 5 | 0 | 0 | 0 | 1 | 0 |
| kb743307.1_13926_star | kb743307.1_13926_star | ggaggctgcccctgcagga | 0 | 1 | 2 | 2 | 0 | 1 | 3 | 1 |
| kb742873.1_1665_mature | kb742873.1_1665_mature | ctgtgattatgatttggact | 3 | 1 | 2 | 1 | 2 | 0 | 0 | 1 |
| kb743929.1_17191_star | kb743929.1_17191_star | acgaagtgttacagacataat | 0 | 3 | 0 | 1 | 0 | 1 | 2 | 0 |
| kb742757.1_11049_mature | kb742757.1_11049_mature | tgctgcaactcttgacaact | 0 | 1 | 3 | 1 | 1 | 0 | 0 | 0 |
| kb745974.1_23661_star | kb745974.1_23661_star | gtgcacctggcctaggaggt | 0 | 1 | 0 | 0 | 0 | 0 | 0 | 0 |
| kb742830.1_16497_mature | kb742830.1_16497_mature | tggacaggctgatagaaacc | 2 | 2 | 3 | 1 | 0 | 0 | 6 | 0 |
| kb744822.1_20008_mature | kb744822.1_20008_mature | tctgtgtctctactgtgga | 0 | 4 | 0 | 1 | 0 | 1 | 2 | 0 |
| kb742781.1_1894_mature | kb742781.1_1894_mature | gatgccggggtgtgactggctgtgg | 0 | 2 | 2 | 0 | 1 | 0 | 3 | 0 |
| kb742830.1_16497_star | kb742830.1_16497_star | aaagtataaagtcctgaacccagg | 2 | 2 | 4 | 1 | 2 | 2 | 2 | 1 |
| kb744462.1_23603_mature | kb744462.1_23603_mature | catcaggccctcgtgcaccagg | 1 | 4 | 1 | 2 | 5 | 3 | 4 | 3 |
| kb743589.1_20880_mature | kb743589.1_20880_mature | agttgtggagaaaaactt | 1 | 0 | 0 | 0 | 4 | 1 | 2 | 0 |
| kb743111.1_4086_star | kb743111.1_4086_star | tgtgatgtgggaggtgacaatac | 0 | 5 | 5 | 6 | 4 | 1 | 0 | 0 |
| kb744486.1_15068_mature | kb744486.1_15068_mature | tgcaggagtgaggctcgccttgc | 0 | 2 | 4 | 4 | 1 | 0 | 1 | 1 |
| kb743020.1_7810_mature | kb743020.1_7810_mature | tggactatcaaaagaggat | 1 | 14 | 0 | 2 | 1 | 0 | 0 | 0 |
| kb742773.1_1227_mature | kb742773.1_1227_mature | tgttgaacttcatgcatt | 22 | 350 | 5 | 530 | 137 | 6 | 23 | 19 |
| kb742442.1_13514_star | kb742442.1_13514_star | gctcactgttacactcttttt | 0 | 6 | 1 | 0 | 1 | 0 | 1 | 2 |
| kb743090.1_9113_star | kb743090.1_9113_star | ccatgaaatcatacatattatttc | 0 | 0 | 1 | 1 | 0 | 0 | 2 | 0 |
| kb743446.1_11706_mature | kb743446.1_11706_mature | cagccctctcgctcttccccagg | 1 | 2 | 6 | 1 | 2 | 0 | 0 | 0 |
| kb742969.1_1049_star | kb742969.1_1049_star | cgcggcgcgcgcgcacccccc | 2 | 8 | 4 | 5 | 8 | 1 | 11 | 15 |
| kb743943.1_22946_mature | kb743943.1_22946_mature | caaggcttctgacactgtctcccat | 6 | 63 | 3 | 66 | 24 | 3 | 7 | 4 |
| kb743399.1_14091_mature | kb743399.1_14091_mature | ttgttaagatctctaaaa | 1 | 2 | 0 | 5 | 0 | 0 | 5 | 1 |
| kb745208.1_24259_star | kb745208.1_24259_star | gcggcgtgctgggtatcgcgat | 0 | 3 | 3 | 0 | 4 | 0 | 0 | 11 |
| kb743356.1_12151_mature | kb743356.1_12151_mature | aggattttgttgggttgact | 4 | 4 | 0 | 5 | 0 | 1 | 2 | 0 |
| kb743021.1_5558_mature | kb743021.1_5558_mature | ggcttgcctgtaagactt | 3 | 2 | 1 | 1 | 0 | 0 | 0 | 1 |
| kb742981.1_12294_mature | kb742981.1_12294_mature | cattacagtagaacctcact | 3 | 1 | 0 | 0 | 0 | 0 | 0 | 0 |
| kb742703.1_20203_mature | kb742703.1_20203_mature | tggaattgagtcttttgaact | 0 | 1 | 3 | 0 | 0 | 1 | 4 | 0 |
| kb744020.1_10239_mature | kb744020.1_10239_mature | acgaactactgtattgat | 0 | 4 | 1 | 0 | 0 | 0 | 0 | 0 |
| kb763423.1_26018_star | kb763423.1_26018_star | tccagctcctggcagagcc | 1 | 0 | 2 | 0 | 1 | 0 | 0 | 2 |
| kb743922.1_17818_star | kb743922.1_17818_star | attgcagtcacgaagggttaaagt | 0 | 1 | 0 | 0 | 0 | 0 | 1 | 0 |
| kb742659.1_3611_star | kb742659.1_3611_star | gctgcttacttttcttttttccc | 1 | 0 | 4 | 0 | 0 | 0 | 1 | 0 |
| kb744306.1_3705_star | kb744306.1_3705_star | cctttcagaacttcagctgaa | 1 | 7 | 0 | 0 | 0 | 1 | 2 | 1 |
| kb742588.1_6957_star | kb742588.1_6957_star | ctgatgttatggtaaat | 0 | 0 | 0 | 0 | 0 | 0 | 1 | 0 |
| kb743323.1_3663_star | kb743323.1_3663_star | agccatgggagagaggaagtgggctc | 2 | 3 | 3 | 3 | 3 | 0 | 0 | 1 |
| kb742989.1_22227_mature | kb742989.1_22227_mature | tcggtagagcatgagac | 40 | 143 | 129 | 54 | 47 | 56 | 65 | 55 |
| kb744241.1_23721_mature | kb744241.1_23721_mature | cggagaagcagtggctcaagg | 1 | 9 | 4 | 3 | 2 | 0 | 8 | 0 |
| kb742572.1_11741_star | kb742572.1_11741_star | tgttacagactgaaa | 0 | 0 | 0 | 0 | 0 | 0 | 1 | 0 |
| kb742963.1_10694_mature | kb742963.1_10694_mature | caggcgggtgctgagcacc | 0 | 3 | 3 | 2 | 3 | 2 | 4 | 4 |
| kb742489.1_7303_mature | kb742489.1_7303_mature | taagcctcgtgtctctgcagt | 13 | 15 | 34 | 20 | 13 | 8 | 28 | 5 |
| kb743100.1_5653_mature | kb743100.1_5653_mature | ttcctctgcacttccatatagcc | 1 | 23 | 1 | 37 | 3 | 4 | 3 | 1 |
| kb742531.1_10744_mature | kb742531.1_10744_mature | aagactggtagaaagaaa | 3 | 1 | 1 | 0 | 1 | 0 | 2 | 0 |
| kb742444.1_8018_star | kb742444.1_8018_star | caatgttgtcgatcccatgac | 0 | 1 | 1 | 0 | 0 | 0 | 0 | 0 |
| kb744033.1_9320_star | kb744033.1_9320_star | gcatctatccggagactggaga | 0 | 0 | 0 | 0 | 0 | 1 | 0 | 0 |
| kb743171.1_10227_star | kb743171.1_10227_star | acatgctttctgatgcaga | 0 | 0 | 2 | 1 | 0 | 1 | 0 | 0 |
| kb769248.1_25969_mature | kb769248.1_25969_mature | ctggctggatttgctgtcc | 0 | 5 | 2 | 0 | 0 | 5 | 11 | 0 |
| kb743217.1_9778_star | kb743217.1_9778_star | aagtgtatgctttcttac | 0 | 2 | 0 | 0 | 0 | 1 | 0 | 2 |
| kb742542.1_13723_star | kb742542.1_13723_star | gtgcagtttgtgcttgatactga | 0 | 1 | 0 | 0 | 0 | 0 | 5 | 0 |
| kb743441.1_16401_star | kb743441.1_16401_star | aggagcaaaagagcagtg | 0 | 0 | 1 | 0 | 1 | 0 | 0 | 0 |
| kb744484.1_23486_mature | kb744484.1_23486_mature | tgctgtctgggcctgatctcc | 27 | 26 | 5 | 58 | 15 | 1 | 4 | 0 |
| kb743458.1_14268_mature | kb743458.1_14268_mature | taaagaagtgtttctgagcgg | 3 | 8 | 9 | 3 | 4 | 3 | 7 | 4 |
| kb743686.1_3421_mature | kb743686.1_3421_mature | acgatttgatttctaaac | 0 | 0 | 0 | 3 | 1 | 0 | 0 | 0 |
| kb742963.1_10694_star | kb742963.1_10694_star | tgctggcacccacccggt | 0 | 0 | 1 | 0 | 0 | 0 | 0 | 2 |
| kb742741.1_17419_star | kb742741.1_17419_star | acatgattggacccagtattgac | 0 | 1 | 1 | 0 | 0 | 1 | 1 | 0 |
| kb742799.1_11292_star | kb742799.1_11292_star | gaatggggcttgtaagaagtg | 0 | 0 | 1 | 1 | 0 | 0 | 3 | 0 |
| kb743109.1_14533_star | kb743109.1_14533_star | tattaaaggctagaacattt | 1 | 1 | 1 | 1 | 0 | 0 | 1 | 1 |
| kb743923.1_22878_mature | kb743923.1_22878_mature | gaagaattcttctcttgatgcaacc | 0 | 0 | 4 | 0 | 0 | 0 | 1 | 1 |
| kb743520.1_3890_mature | kb743520.1_3890_mature | agtgtctgcaggatctcagctgc | 8 | 12 | 8 | 7 | 12 | 8 | 16 | 9 |
| kb743558.1_17512_star | kb743558.1_17512_star | aagggaatgggggctggagaag | 0 | 1 | 3 | 5 | 1 | 1 | 2 | 0 |
| kb744462.1_23599_star | kb744462.1_23599_star | ccatgtccccccacggcagctcc | 0 | 1 | 4 | 0 | 0 | 2 | 0 | 2 |
| kb742730.1_8104_mature | kb742730.1_8104_mature | ttctgtagtgttggactct | 1 | 6 | 5 | 5 | 0 | 7 | 2 | 0 |
| kb744105.1_20140_mature | kb744105.1_20140_mature | ctgggattttggggcagaacctg | 9 | 143 | 190 | 40 | 2 | 2 | 13 | 1 |
| kb742928.1_10835_star | kb742928.1_10835_star | aggaatggaaaatggagaccgtt | 1 | 15 | 4 | 5 | 3 | 1 | 3 | 2 |
| kb743745.1_15558_mature | kb743745.1_15558_mature | aggataaggacagggaca | 0 | 0 | 1 | 0 | 1 | 0 | 0 | 3 |
| kb742659.1_3602_mature | kb742659.1_3602_mature | caacggatgaggaagatgatg | 1 | 6 | 8 | 3 | 0 | 1 | 4 | 2 |
| kb742503.1_11004_star | kb742503.1_11004_star | ccttaataggcctggaaaaa | 0 | 0 | 0 | 2 | 0 | 0 | 1 | 0 |
| kb742531.1_10714_star | kb742531.1_10714_star | tgcagagggttttctgtttgg | 0 | 1 | 0 | 1 | 0 | 0 | 1 | 1 |
| kb742808.1_104_star | kb742808.1_104_star | tagtccacagaggtgagtaccaac | 0 | 2 | 1 | 1 | 0 | 0 | 0 | 0 |
| kb742651.1_5201_mature | kb742651.1_5201_mature | aatgcactggggctgtgtctgc | 20 | 16 | 38 | 16 | 17 | 2 | 8 | 4 |
| kb743204.1_4203_mature | kb743204.1_4203_mature | gaaggacatcgaggccctggagt | 7 | 26 | 1 | 56 | 5 | 0 | 0 | 0 |
| kb742622.1_3830_mature | kb742622.1_3830_mature | ctgtttgtgatgtagcatc | 1 | 8 | 2 | 5 | 2 | 2 | 14 | 2 |
| kb743176.1_3945_star | kb743176.1_3945_star | aaacacaggtaacagcagtgtgt | 1 | 5 | 0 | 2 | 1 | 0 | 0 | 1 |
| kb743192.1_11618_star | kb743192.1_11618_star | acaaagcaacagcatagtt | 0 | 0 | 1 | 0 | 0 | 0 | 2 | 0 |
| kb742887.1_1558_star | kb742887.1_1558_star | gttggtcagacaggacat | 0 | 1 | 0 | 0 | 0 | 0 | 1 | 0 |
| kb743220.1_18067_star | kb743220.1_18067_star | aaatcttcaatatttgtgacc | 0 | 0 | 0 | 0 | 0 | 0 | 1 | 0 |
| kb742439.1_3150_mature | kb742439.1_3150_mature | aaagagccacctattaaacttccaa | 0 | 0 | 4 | 0 | 0 | 0 | 2 | 0 |
| kb743625.1_7626_mature | kb743625.1_7626_mature | tctaagatgagactgcagtct | 0 | 1 | 3 | 3 | 0 | 0 | 7 | 0 |
| kb742531.1_10747_star | kb742531.1_10747_star | gctgcagatcctgaacgtgtcc | 0 | 4 | 2 | 0 | 1 | 0 | 5 | 0 |
| kb744722.1_10794_mature | kb744722.1_10794_mature | aactgaaggttggagact | 0 | 0 | 5 | 2 | 4 | 1 | 1 | 1 |
| kb742712.1_8598_mature | kb742712.1_8598_mature | tggaagatgtgtgcaaa | 0 | 5 | 3 | 3 | 0 | 1 | 5 | 0 |
| kb745110.1_19018_star | kb745110.1_19018_star | aggtgtcgcacagaactctgt | 0 | 4 | 1 | 3 | 2 | 0 | 1 | 1 |
| kb742411.1_7602_mature | kb742411.1_7602_mature | atcattttctttcataagc | 3 | 1 | 1 | 0 | 0 | 0 | 1 | 0 |
| kb742459.1_3538_star | kb742459.1_3538_star | aagtgctaagatctgaacatgac | 1 | 2 | 1 | 0 | 0 | 0 | 2 | 0 |

| kb743461.1_19803_mature | kb743461.1_19803_mature | cgaggaggaagaggaggct | 1 | 6 | 20 | 5 | 7 | 0 | 5 | 7 |
| --- | --- | --- | --- | --- | --- | --- | --- | --- | --- | --- |
| kb743437.1_21710_mature | kb743437.1_21710_mature | cactagaagctgaacaagctgt | 3 | 3 | 3 | 1 | 0 | 0 | 1 | 0 |
| kb742810.1_4993_mature | kb742810.1_4993_mature | gctgtttggcttgtgtgacatc | 0 | 4 | 1 | 0 | 1 | 1 | 1 | 2 |
| kb743073.1_9073_star | kb743073.1_9073_star | acatccagagcagctttg | 0 | 1 | 2 | 0 | 0 | 3 | 0 | 1 |
| kb743520.1_3906_star | kb743520.1_3906_star | ttctgcagagagcaagcacag | 1 | 1 | 1 | 0 | 0 | 1 | 5 | 2 |
| kb743748.1_19042_star | kb743748.1_19042_star | atggattgtttattactttttctga | 0 | 2 | 0 | 2 | 1 | 0 | 2 | 0 |
| kb742664.1_9855_star | kb742664.1_9855_star | caatctggcacctttcacgagcgca | 1 | 1 | 0 | 0 | 1 | 0 | 0 | 0 |
| kb742556.1_16191_star | kb742556.1_16191_star | tagatagagacaaagaaggcatt | 0 | 3 | 0 | 0 | 0 | 0 | 1 | 0 |
| kb753911.1_25543_star | kb753911.1_25543_star | tgtttccattcttgttt | 0 | 0 | 1 | 0 | 0 | 0 | 1 | 0 |
| kb743356.1_12126_star | kb743356.1_12126_star | gctcgtggatgccgtgggcga | 1 | 0 | 1 | 0 | 1 | 0 | 1 | 0 |
| kb743136.1_7894_star | kb743136.1_7894_star | tagaggatacaaggcaatgattatg | 2 | 0 | 2 | 0 | 0 | 0 | 1 | 0 |
| kb742777.1_8210_mature | kb742777.1_8210_mature | actgcagtagacgaaagc | 1 | 2 | 0 | 1 | 1 | 3 | 0 | 0 |
| kb744049.1_21211_mature | kb744049.1_21211_mature | tttccagaactgcttgtgagc | 0 | 2 | 2 | 2 | 1 | 0 | 2 | 0 |
| kb743518.1_15368_star | kb743518.1_15368_star | ctctactcctgtcagtgttatt | 0 | 1 | 1 | 1 | 2 | 0 | 4 | 0 |
| kb742544.1_17751_star | kb742544.1_17751_star | cttccacctgttgactctg | 0 | 0 | 0 | 0 | 0 | 0 | 1 | 0 |
| kb743200.1_11492_star | kb743200.1_11492_star | ccaatcagttctgaagtg | 1 | 0 | 0 | 1 | 0 | 0 | 1 | 0 |
| kb742757.1_11048_mature | kb742757.1_11048_mature | ccgacagctgatcagtgctc | 0 | 0 | 1 | 0 | 2 | 0 | 5 | 0 |
| kb802624.1_25541_star | kb802624.1_25541_star | tctttctgattggagccgtt | 1 | 1 | 0 | 2 | 2 | 1 | 2 | 1 |
| kb742807.1_16449_mature | kb742807.1_16449_mature | tgtgtgtgtgtgtgctctgc | 4 | 9 | 15 | 7 | 6 | 0 | 9 | 2 |
| kb744462.1_23614_star | kb744462.1_23614_star | tcctggtgcacaaggacctggtg | 2 | 11 | 8 | 13 | 2 | 3 | 4 | 3 |
| kb742811.1_13098_star | kb742811.1_13098_star | gcttcctagaactctgca | 0 | 4 | 0 | 0 | 0 | 0 | 1 | 0 |
| kb742397.1_12275_mature | kb742397.1_12275_mature | ttgtgtagagctctgataatt | 0 | 2 | 1 | 1 | 1 | 5 | 4 | 0 |
| kb744706.1_23010_star | kb744706.1_23010_star | aaagagctgtttgttctgaaca | 2 | 5 | 4 | 4 | 1 | 2 | 5 | 2 |
| kb743343.1_14295_star | kb743343.1_14295_star | gtctgtccccacatgcccaaa | 5 | 9 | 0 | 13 | 8 | 0 | 2 | 2 |
| kb742622.1_3805_star | kb742622.1_3805_star | gtgttcaaaaaacagtgctacagca | 0 | 2 | 3 | 2 | 0 | 2 | 5 | 0 |
| kb742439.1_3172_mature | kb742439.1_3172_mature | aaaagatagtgctgaagtaagaaca | 0 | 10 | 3 | 4 | 2 | 5 | 5 | 0 |
| kb743171.1_10219_star | kb743171.1_10219_star | gtttgagttctgcattt | 1 | 2 | 2 | 0 | 0 | 0 | 0 | 0 |
| kb742554.1_1746_mature | kb742554.1_1746_mature | agtgcaaaggcagctcatgctct | 35 | 104 | 55 | 139 | 76 | 67 | 51 | 62 |
| kb744105.1_20113_star | kb744105.1_20113_star | attgttcagcagctcgaagttg | 0 | 2 | 2 | 1 | 0 | 0 | 0 | 1 |
| kb742579.1_8731_mature | kb742579.1_8731_mature | aggtgagcctccgttact | 0 | 1 | 7 | 1 | 0 | 1 | 0 | 1 |
| kb742522.1_6455_mature | kb742522.1_6455_mature | agttctgtgtgtaatttctgtgt | 2 | 3 | 8 | 0 | 0 | 4 | 11 | 5 |
| kb744306.1_3751_star | kb744306.1_3751_star | ttttttttttgaaatatttctttccc | 1 | 0 | 1 | 1 | 0 | 1 | 2 | 0 |
| kb743531.1_19939_star | kb743531.1_19939_star | ttgtcatggctacactgctgtcggt | 1 | 1 | 1 | 1 | 3 | 1 | 2 | 1 |
| kb743179.1_21673_mature | kb743179.1_21673_mature | agtgatgatggtatccca | 0 | 5 | 0 | 0 | 0 | 0 | 0 | 0 |
| kb743569.1_19234_mature | kb743569.1_19234_mature | ttctctgaatcactgcctttgt | 0 | 1 | 1 | 1 | 0 | 1 | 4 | 0 |
| kb743644.1_3285_mature | kb743644.1_3285_mature | cccttctgttctgagaatcagc | 0 | 0 | 1 | 3 | 2 | 2 | 3 | 0 |
| kb742867.1_9028_mature | kb742867.1_9028_mature | agggctgtcggggtcctcctcatg | 2 | 4 | 2 | 3 | 3 | 0 | 4 | 0 |
| kb742929.1_329_mature | kb742929.1_329_mature | ccgattgttttgcgaagc | 0 | 2 | 3 | 0 | 1 | 0 | 1 | 0 |
| kb742855.1_12689_mature | kb742855.1_12689_mature | ctggaagtgcagctctggcactgc | 0 | 4 | 2 | 3 | 6 | 5 | 6 | 4 |
| kb743300.1_12062_star | kb743300.1_12062_star | ctactggtgttgtgcttttg | 20 | 129 | 0 | 122 | 58 | 1 | 2 | 2 |
| kb742770.1_17490_mature | kb742770.1_17490_mature | tctgagtgccttctgtcctagg | 0 | 9 | 1 | 0 | 1 | 2 | 2 | 2 |
| kb742421.1_16835_mature | kb742421.1_16835_mature | cggagatgccccaagccctgca | 2 | 4 | 9 | 7 | 2 | 1 | 1 | 1 |
| kb743020.1_7771_mature | kb743020.1_7771_mature | ccggcacaactttgggttc | 15 | 33 | 0 | 161 | 1 | 0 | 3 | 0 |
| kb742543.1_11170_star | kb742543.1_11170_star | atgggcattcctacaccacaga | 1 | 2 | 0 | 0 | 0 | 1 | 2 | 0 |
| kb742537.1_6831_mature | kb742537.1_6831_mature | aatctctgagccacagtgccttg | 2 | 8 | 1 | 6 | 2 | 2 | 2 | 0 |
| kb743662.1_7538_star | kb743662.1_7538_star | atactgaaagcaggccgag | 1 | 0 | 0 | 0 | 0 | 0 | 1 | 0 |
| kb742701.1_6472_mature | kb742701.1_6472_mature | gccgcatctaggatgaattt | 2 | 0 | 0 | 0 | 0 | 3 | 0 | 2 |
| kb742992.1_5465_mature | kb742992.1_5465_mature | agcgtggttcttctgtcctact | 6 | 11 | 10 | 1 | 3 | 0 | 3 | 3 |
| kb743183.1_7711_mature | kb743183.1_7711_mature | ttgtctactagaaattctgt | 0 | 4 | 1 | 1 | 0 | 0 | 4 | 3 |
| kb744170.1_17366_star | kb744170.1_17366_star | ggatctgcaggactaataaagc | 2 | 1 | 3 | 2 | 1 | 1 | 3 | 2 |
| kb742406.1_7412_star | kb742406.1_7412_star | agcaccatcattcaggaa | 0 | 0 | 0 | 0 | 0 | 1 | 1 | 0 |
| kb742451.1_6350_mature | kb742451.1_6350_mature | tggtctagtttgtactga | 0 | 0 | 0 | 2 | 0 | 0 | 3 | 0 |
| kb742554.1_1806_star | kb742554.1_1806_star | tccttttctcatcagagagca | 25 | 19 | 17 | 26 | 10 | 8 | 36 | 26 |
| kb743311.1_18858_mature | kb743311.1_18858_mature | agcgtgtgctggaggctcca | 30 | 51 | 51 | 41 | 26 | 25 | 36 | 33 |
| kb744553.1_17004_mature | kb744553.1_17004_mature | ttctggggcacgtggagaagct | 1 | 2 | 4 | 2 | 1 | 0 | 6 | 1 |
| kb743633.1_15479_star | kb743633.1_15479_star | tccaggatcacctcctc | 6 | 9 | 4 | 3 | 2 | 2 | 1 | 6 |
| kb742542.1_13723_mature | kb742542.1_13723_mature | tgtgtctagatcaaagtgtctct | 2 | 3 | 1 | 4 | 0 | 0 | 4 | 0 |
| kb742833.1_57_star | kb742833.1_57_star | attcttcaaaaataaaagctt | 2 | 0 | 0 | 0 | 0 | 0 | 0 | 0 |
| kb742955.1_12835_star | kb742955.1_12835_star | caggttactgatcttctc | 1 | 0 | 2 | 1 | 0 | 0 | 2 | 0 |
| kb742531.1_10722_star | kb742531.1_10722_star | tgttcttaatgtggtgtttatt | 0 | 0 | 1 | 4 | 0 | 0 | 2 | 0 |
| kb743139.1_2227_mature | kb743139.1_2227_mature | acagcgggtgaaggactaagcaga | 2 | 6 | 5 | 4 | 1 | 0 | 1 | 3 |
| kb743948.1_17925_star | kb743948.1_17925_star | agggaaatacaagttcagga | 0 | 1 | 0 | 0 | 2 | 0 | 0 | 1 |
| kb743556.1_17243_mature | kb743556.1_17243_mature | aagaactttgaagagag | 65 | 99 | 70 | 61 | 36 | 20 | 60 | 70 |
| kb743518.1_15376_star | kb743518.1_15376_star | caaagggaggggtaggtttag | 1 | 5 | 4 | 6 | 0 | 0 | 0 | 0 |
| kb743474.1_21446_mature | kb743474.1_21446_mature | tagagagaactactgaca | 0 | 3 | 2 | 2 | 2 | 2 | 1 | 2 |
| kb743204.1_4247_star | kb743204.1_4247_star | tacatgctgatgctagctgagttattt | 0 | 0 | 0 | 2 | 0 | 0 | 1 | 1 |
| kb743004.1_8918_star | kb743004.1_8918_star | aaaaaacatggctttg | 1 | 0 | 0 | 0 | 1 | 0 | 0 | 0 |
| kb742816.1_17809_mature | kb742816.1_17809_mature | tgacgtgaggctgacaggcctgt | 74 | 317 | 3 | 731 | 56 | 1 | 24 | 7 |
| kb743110.1_6049_mature | kb743110.1_6049_mature | cctgtggctgtctttgc | 4 | 14 | 6 | 4 | 6 | 4 | 4 | 2 |
| kb742918.1_13084_star | kb742918.1_13084_star | ggccatgatctcgttgcaatcac | 0 | 0 | 0 | 0 | 0 | 0 | 1 | 0 |
| kb742622.1_3794_mature | kb742622.1_3794_mature | tagtggctggcctccagctggact | 29 | 264 | 6 | 468 | 49 | 5 | 12 | 3 |
| kb743829.1_10852_mature | kb743829.1_10852_mature | aacttccgactgtctcaggacga | 12 | 35 | 346 | 34 | 12 | 4 | 13 | 6 |
| kb744484.1_23486_star | kb744484.1_23486_star | agatgaggttggtaggcaggact | 0 | 1 | 1 | 0 | 1 | 1 | 1 | 0 |
| kb744292.1_12777_star | kb744292.1_12777_star | aggtgtgtaatattataaaa | 0 | 0 | 0 | 0 | 0 | 0 | 1 | 0 |
| kb742896.1_17385_star | kb742896.1_17385_star | attttccaagtgaaataaggaca | 1 | 1 | 1 | 1 | 0 | 0 | 1 | 0 |
| kb745404.1_16854_star | kb745404.1_16854_star | cttactttgcagagaatt | 2 | 0 | 0 | 0 | 0 | 0 | 2 | 0 |
| kb742627.1_5359_star | kb742627.1_5359_star | ctcagactgaaccctgtacttca | 1 | 4 | 7 | 0 | 0 | 1 | 1 | 0 |
| kb742810.1_4972_star | kb742810.1_4972_star | cacatggattcagctctg | 0 | 0 | 1 | 0 | 0 | 0 | 2 | 1 |
| kb743685.1_10032_mature | kb743685.1_10032_mature | ctgaaaatcgacgtgttcttaac | 1 | 3 | 0 | 1 | 1 | 5 | 10 | 0 |
| kb742406.1_7412_mature | kb742406.1_7412_mature | tttgatttgtggtactca | 4 | 0 | 0 | 0 | 1 | 0 | 5 | 0 |
| kb742531.1_10747_mature | kb742531.1_10747_mature | aacgtgactgggatcagcaactt | 2 | 4 | 4 | 1 | 1 | 1 | 2 | 1 |
| kb742418.1_2444_mature | kb742418.1_2444_mature | tggttgattttgtagaa | 3 | 11 | 0 | 4 | 0 | 0 | 2 | 0 |
| kb743204.1_4247_mature | kb743204.1_4247_mature | ataactgtgctctgatctctgtaat | 7 | 27 | 13 | 13 | 5 | 6 | 9 | 3 |
| kb743829.1_10876_star | kb743829.1_10876_star | agacgagagatcgtccggaaaac | 0 | 2 | 0 | 0 | 0 | 0 | 0 | 0 |
| kb743204.1_4191_star | kb743204.1_4191_star | ctgagtcagtaatgctatt | 1 | 1 | 0 | 1 | 0 | 0 | 0 | 0 |
| kb747464.1_24596_mature | kb747464.1_24596_mature | atgttctgcagtggttttctactcc | 4 | 3 | 8 | 1 | 3 | 0 | 13 | 1 |
| kb743297.1_21458_mature | kb743297.1_21458_mature | caacggctgtctgattgtcaga | 5 | 9 | 10 | 12 | 2 | 0 | 13 | 0 |
| kb743609.1_4596_star | kb743609.1_4596_star | catgttatgctctcagttgtga | 0 | 1 | 0 | 1 | 0 | 0 | 0 | 0 |
| kb742678.1_15758_star | kb742678.1_15758_star | actttgaaaatacagaaagct | 0 | 0 | 0 | 1 | 1 | 0 | 1 | 0 |
| kb742658.1_9170_star | kb742658.1_9170_star | ctgatattttcggttaaaggt | 0 | 1 | 0 | 0 | 0 | 0 | 3 | 0 |
| kb742622.1_3830_star | kb742622.1_3830_star | tgaaatagatacacaccagaa | 0 | 1 | 5 | 1 | 0 | 0 | 1 | 0 |
| kb743109.1_14541_mature | kb743109.1_14541_mature | aatatgaggactggctgca | 0 | 10 | 2 | 6 | 0 | 0 | 1 | 4 |
| kb742659.1_3602_star | kb742659.1_3602_star | tctacttctggaccagacgatgtc | 0 | 2 | 3 | 1 | 0 | 1 | 4 | 0 |
| kb742685.1_1592_star | kb742685.1_1592_star | aggtcagcattggctcctgtcttc | 1 | 5 | 7 | 2 | 1 | 3 | 3 | 4 |
| kb743897.1_19283_mature | kb743897.1_19283_mature | tcagctgcagcatgagcag | 0 | 3 | 0 | 1 | 0 | 0 | 0 | 2 |
| kb742554.1_1833_star | kb742554.1_1833_star | agttgatctctgctttcagacc | 0 | 1 | 2 | 2 | 2 | 2 | 4 | 1 |
| kb744307.1_22722_mature | kb744307.1_22722_mature | tgagatggagcactgcagcc | 35668 | 32152 | 36750 | 36267 | 48930 | 50291 | 47469 | 58381 |
| kb815851.1_25129_star | kb815851.1_25129_star | tttaactcttaagttcttta | 1 | 0 | 0 | 1 | 0 | 0 | 1 | 0 |
| kb743650.1_19108_mature | kb743650.1_19108_mature | tctattgactgaacaggattt | 0 | 1 | 1 | 0 | 0 | 0 | 4 | 0 |
| kb742921.1_16135_star | kb742921.1_16135_star | ctggggctcccagctaaacccattac | 0 | 0 | 1 | 1 | 1 | 0 | 1 | 1 |
| kb742690.1_5391_star | kb742690.1_5391_star | ctgttggggttgaggcag | 0 | 0 | 0 | 2 | 0 | 0 | 1 | 0 |
| kb742725.1_13870_star | kb742725.1_13870_star | cacagtttcctgcagcaggtaatga | 0 | 1 | 2 | 0 | 0 | 2 | 1 | 0 |
| kb743493.1_18284_mature | kb743493.1_18284_mature | gagatttcctgagctttgtttg | 5 | 4 | 18 | 1 | 4 | 1 | 19 | 3 |
| kb743625.1_7618_star | kb743625.1_7618_star | gtgtaggatactgaaaacatggca | 1 | 3 | 1 | 3 | 0 | 0 | 3 | 0 |
| kb742651.1_5217_mature | kb742651.1_5217_mature | tgagatgaagccctgga | 2303 | 2368 | 2528 | 2404 | 2511 | 3365 | 2654 | 4526 |
| kb743912.1_17130_mature | kb743912.1_17130_mature | tggggggggagggaaggag | 5 | 1 | 4 | 5 | 7 | 2 | 6 | 2 |
| kb742459.1_3529_mature | kb742459.1_3529_mature | gagagccgggaaggacgtg | 2 | 4 | 2 | 1 | 6 | 0 | 3 | 1 |
| kb743685.1_10032_star | kb743685.1_10032_star | ttgggctctcgtgtttttgtggt | 0 | 3 | 3 | 2 | 2 | 0 | 0 | 0 |
| kb819533.1_24845_star | kb819533.1_24845_star | ctggggctcccagttaaacccattac | 0 | 2 | 0 | 0 | 0 | 1 | 2 | 0 |
| kb742427.1_18768_mature | kb742427.1_18768_mature | gaggcctcgggcagagctcg | 3 | 2 | 0 | 2 | 1 | 0 | 0 | 4 |
| kb744105.1_20146_star | kb744105.1_20146_star | tggagcttgagtcatgataacatcctggga | 0 | 0 | 0 | 2 | 1 | 0 | 1 | 0 |
| kb742808.1_144_star | kb742808.1_144_star | tccaacttgcattttatatgct | 0 | 0 | 0 | 1 | 0 | 0 | 0 | 0 |
| kb819001.1_24903_star | kb819001.1_24903_star | gtgtgcgggtaaggagcagcagg | 1 | 0 | 2 | 0 | 0 | 0 | 1 | 0 |
| kb743284.1_19602_star | kb743284.1_19602_star | tcattttccaggcacgagcacgg | 1 | 0 | 0 | 0 | 0 | 0 | 2 | 0 |
| kb744087.1_18315_star | kb744087.1_18315_star | ttagctttgtcctgttcttt | 1 | 2 | 1 | 2 | 0 | 3 | 0 | 0 |
| kb743893.1_20154_mature | kb743893.1_20154_mature | caccctcctttcactttgccagt | 4 | 0 | 9 | 10 | 1 | 1 | 30 | 3 |
| kb742553.1_13406_star | kb742553.1_13406_star | gagactaaagggactacgtgacc | 4 | 11 | 4 | 4 | 1 | 1 | 10 | 5 |
| kb743317.1_3307_star | kb743317.1_3307_star | tttaatgctttctctaggg | 0 | 1 | 1 | 0 | 0 | 0 | 3 | 0 |
| kb754269.1_25092_star | kb754269.1_25092_star | gccagtcctgtgggcaggcagag | 1 | 3 | 0 | 3 | 2 | 1 | 2 | 0 |
| kb742711.1_5019_star | kb742711.1_5019_star | agtggagttaacctgatttaagc | 0 | 1 | 0 | 0 | 0 | 2 | 1 | 0 |
| kb742907.1_4742_mature | kb742907.1_4742_mature | tcagtctgtaacttcgt | 13 | 45 | 1 | 31 | 29 | 0 | 7 | 3 |
| kb742899.1_7138_mature | kb742899.1_7138_mature | ggcacctggatgtggattt | 3 | 7 | 5 | 11 | 5 | 2 | 7 | 6 |
| kb744088.1_3992_star | kb744088.1_3992_star | tttcttctggtatcaaa | 0 | 0 | 2 | 0 | 0 | 0 | 1 | 0 |
| kb742811.1_13098_mature | kb742811.1_13098_mature | ccagactgaacaagctc | 3 | 13 | 1 | 29 | 2 | 0 | 1 | 1 |
| kb742616.1_12588_mature | kb742616.1_12588_mature | aatttgtgggagttctgggatg | 3 | 7 | 8 | 1 | 2 | 1 | 3 | 4 |
| kb742471.1_425_mature | kb742471.1_425_mature | actcagactggctcaaaga | 1 | 1 | 2 | 3 | 8 | 0 | 2 | 0 |
| kb743923.1_22878_star | kb743923.1_22878_star | ttcagtcgaatcaaagccttctatgtc | 0 | 0 | 0 | 1 | 1 | 0 | 1 | 1 |
| kb742830.1_16496_star | kb742830.1_16496_star | agttctttgtggatcagcccca | 0 | 1 | 0 | 1 | 0 | 1 | 0 | 0 |
| kb742448.1_5971_mature | kb742448.1_5971_mature | cgacgtgagactgacaggcctgt | 89 | 311 | 9 | 827 | 64 | 2 | 22 | 11 |
| kb744942.1_17914_star | kb744942.1_17914_star | gatactagtgatccattg | 0 | 0 | 0 | 1 | 0 | 0 | 0 | 0 |
| kb743197.1_1145_mature | kb743197.1_1145_mature | tgccaactgagaaaagatgac | 1 | 0 | 2 | 1 | 1 | 0 | 1 | 4 |
| kb794474.1_25695_mature | kb794474.1_25695_mature | ccactgcttcacttgaca | 2 | 0 | 1 | 1 | 0 | 0 | 1 | 0 |
| kb742629.1_520_mature | kb742629.1_520_mature | tggtgcaaaaccctgagcagc | 0 | 4 | 0 | 1 | 0 | 1 | 1 | 0 |
| kb744699.1_17456_mature | kb744699.1_17456_mature | tgtggtgggctgggcgggt | 16 | 9 | 22 | 25 | 8 | 25 | 8 | 31 |
| kb743158.1_2878_mature | kb743158.1_2878_mature | ggtgcctgaggactggtga | 2 | 0 | 0 | 1 | 0 | 0 | 0 | 5 |
| kb742960.1_17261_mature | kb742960.1_17261_mature | ttgttctctgaatacccag | 1 | 14 | 0 | 12 | 0 | 0 | 2 | 0 |
| kb743090.1_9126_mature | kb743090.1_9126_mature | ctggaagggaacttgca | 0 | 5 | 2 | 2 | 3 | 0 | 2 | 1 |
| kb743113.1_6633_star | kb743113.1_6633_star | tttcaagtgccatcttttt | 0 | 3 | 1 | 1 | 0 | 0 | 1 | 0 |
| kb744113.1_10669_mature | kb744113.1_10669_mature | cgagttctgtgttcagttttgggct | 72 | 298 | 2 | 386 | 84 | 3 | 40 | 14 |
| kb742928.1_10842_star | kb742928.1_10842_star | tgatgatgggcttcgggacccaggg | 0 | 1 | 5 | 2 | 1 | 1 | 3 | 0 |
| kb743067.1_21825_star | kb743067.1_21825_star | gccttacagatgtccacgta | 0 | 1 | 3 | 0 | 1 | 0 | 0 | 1 |
| kb744221.1_21790_mature | kb744221.1_21790_mature | cggacagggtgccttatc | 0 | 0 | 1 | 1 | 3 | 0 | 0 | 0 |
| kb743748.1_19049_mature | kb743748.1_19049_mature | ctccttgtggtccccctgcagg | 4 | 5 | 1 | 2 | 0 | 0 | 1 | 2 |
| kb742499.1_8270_star | kb742499.1_8270_star | tcatcaatactgtctttt | 0 | 0 | 0 | 1 | 0 | 0 | 1 | 0 |
| kb743545.1_16435_mature | kb743545.1_16435_mature | tttggaaagaagcgtctga | 0 | 3 | 1 | 1 | 5 | 0 | 0 | 0 |
| kb742442.1_13521_mature | kb742442.1_13521_mature | cgacttgtgtttctgtca | 0 | 5 | 1 | 1 | 0 | 1 | 0 | 1 |
| kb743412.1_5055_star | kb743412.1_5055_star | gtgtggctctgcccagg | 0 | 5 | 2 | 0 | 0 | 2 | 2 | 1 |
| kb742929.1_329_star | kb742929.1_329_star | agagcaaaataaataaaggga | 3 | 0 | 1 | 0 | 0 | 0 | 1 | 0 |
| kb745553.1_25354_star | kb745553.1_25354_star | cgcgcagcacctccagcacctcagg | 3 | 3 | 7 | 7 | 3 | 4 | 3 | 11 |
| kb756629.1_24623_mature | kb756629.1_24623_mature | aggatctttgtctgcaac | 0 | 12 | 1 | 13 | 6 | 1 | 3 | 1 |
| kb744577.1_22379_mature | kb744577.1_22379_mature | tctctgttatctttgaaag | 0 | 13 | 1 | 4 | 0 | 0 | 0 | 0 |
| kb742833.1_57_mature | kb742833.1_57_mature | gcggttactggagaagtg | 1 | 0 | 1 | 2 | 4 | 1 | 1 | 0 |
| kb742984.1_1904_mature | kb742984.1_1904_mature | tttttgactgtattttacaca | 3 | 8 | 0 | 0 | 0 | 0 | 7 | 0 |
| kb743564.1_13642_star | kb743564.1_13642_star | cgaatatattactacttcactgag | 1 | 0 | 2 | 0 | 0 | 1 | 1 | 0 |
| kb742969.1_1047_mature | kb742969.1_1047_mature | agacagatgtttgtgtgtagactt | 1 | 4 | 0 | 0 | 1 | 2 | 6 | 1 |
| kb744413.1_22167_mature | kb744413.1_22167_mature | tccaactcaggactcgattctatt | 0 | 2 | 0 | 7 | 0 | 0 | 1 | 0 |
| kb742588.1_7038_mature | kb742588.1_7038_mature | aatccggttctgagaaatca | 0 | 4 | 5 | 1 | 0 | 0 | 0 | 2 |
| kb742515.1_4128_star | kb742515.1_4128_star | ccaccaggtccataaagcccat | 0 | 0 | 1 | 2 | 0 | 0 | 0 | 1 |
| kb742675.1_10535_star | kb742675.1_10535_star | tttactcctcatat | 0 | 0 | 1 | 0 | 0 | 0 | 0 | 0 |
| kb743217.1_9785_star | kb743217.1_9785_star | ctactcttcttgcctat | 0 | 0 | 0 | 1 | 0 | 0 | 0 | 0 |
| kb742541.1_17016_star | kb742541.1_17016_star | atccttatatcgtgttctca | 0 | 1 | 1 | 0 | 0 | 0 | 1 | 0 |
| kb742618.1_11461_star | kb742618.1_11461_star | tcactcagtcacaga | 0 | 1 | 0 | 1 | 1 | 3 | 1 | 0 |
| kb744477.1_11827_star | kb744477.1_11827_star | tcagttttgcaagataatttta | 0 | 3 | 1 | 0 | 0 | 0 | 1 | 0 |
| kb742992.1_5465_star | kb742992.1_5465_star | taaggaaaggaacaagcatt | 1 | 4 | 0 | 1 | 0 | 1 | 1 | 0 |
| kb743463.1_20523_mature | kb743463.1_20523_mature | tgaggtgaagcactggag | 2599 | 2491 | 2974 | 2742 | 2825 | 3619 | 2987 | 4774 |
| kb744454.1_19867_star | kb744454.1_19867_star | aaatttagccactgcc | 0 | 0 | 1 | 0 | 0 | 0 | 0 | 0 |
| kb742527.1_3881_star | kb742527.1_3881_star | tggcacaatctgagcac | 0 | 0 | 0 | 0 | 0 | 1 | 0 | 0 |
| kb752331.1_25236_star | kb752331.1_25236_star | caataaccagctgcatcagga | 1 | 0 | 0 | 0 | 0 | 0 | 1 | 0 |
| kb742629.1_585_mature | kb742629.1_585_mature | ttgcaagtccttcatgtggagt | 0 | 0 | 5 | 0 | 1 | 2 | 7 | 1 |
| kb743255.1_18607_star | kb743255.1_18607_star | gctgcttttactcaaga | 0 | 1 | 0 | 0 | 0 | 0 | 2 | 0 |
| kb742404.1_2852_mature | kb742404.1_2852_mature | tttggcactgtgatttat | 3 | 0 | 0 | 0 | 0 | 2 | 0 | 0 |
| kb743833.1_10826_mature | kb743833.1_10826_mature | agggcactccgaggacgt | 0 | 0 | 1 | 0 | 3 | 1 | 1 | 0 |
| kb744228.1_23280_star | kb744228.1_23280_star | tgccaatataaccccagtgacagatc | 0 | 1 | 0 | 2 | 0 | 0 | 0 | 2 |
| kb742444.1_8018_mature | kb742444.1_8018_mature | cttgggacaggctgtggg | 0 | 2 | 0 | 0 | 4 | 0 | 1 | 1 |
| kb742784.1_10912_star | kb742784.1_10912_star | tcctctgctgtaggatc | 1 | 0 | 0 | 1 | 1 | 0 | 0 | 0 |
| kb743977.1_12630_star | kb743977.1_12630_star | agtagtgaactctgagaaacgcctct | 2 | 2 | 6 | 2 | 0 | 2 | 3 | 4 |
| kb743547.1_18206_star | kb743547.1_18206_star | agccaggccgttgtgcgttt | 0 | 0 | 1 | 0 | 2 | 0 | 0 | 1 |
| kb742523.1_1953_mature | kb742523.1_1953_mature | cgcgtggaagcaggactg | 1 | 4 | 1 | 4 | 5 | 0 | 6 | 4 |
| kb744725.1_16613_star | kb744725.1_16613_star | ccgtgttagtttcttg | 1 | 0 | 1 | 0 | 0 | 1 | 1 | 1 |
| kb742410.1_18149_star | kb742410.1_18149_star | tgcaggtcagatctgctgttaa | 3 | 14 | 2 | 11 | 4 | 0 | 2 | 0 |
| kb744955.1_13807_star | kb744955.1_13807_star | gtttgttttaaattgtggatatttgg | 0 | 1 | 0 | 3 | 1 | 0 | 1 | 0 |

| kb744209.1_16235_mature | kb744209.1_16235_mature | actggaagtgtgcgaatt | 0 | 0 | 0 | 0 | 0 | 0 | 3 | 0 |
| --- | --- | --- | --- | --- | --- | --- | --- | --- | --- | --- |
| kb743046.1_19357_mature | kb743046.1_19357_mature | gttttgctggtcgttgagtgtctgt | 0 | 0 | 1 | 0 | 1 | 0 | 3 | 0 |
| kb745003.1_21573_mature | kb745003.1_21573_mature | tgatgaacctgggcagct | 0 | 1 | 1 | 2 | 0 | 1 | 0 | 1 |
| kb742816.1_17809_star | kb742816.1_17809_star | aggatcttctttctcacccagc | 0 | 1 | 1 | 1 | 0 | 1 | 1 | 0 |
| kb742452.1_10576_mature | kb742452.1_10576_mature | catgtgttgctctgtgcccgc | 1 | 14 | 2 | 28 | 3 | 0 | 9 | 1 |
| kb742633.1_16972_mature | kb742633.1_16972_mature | ctttgtggtgatgggctcag | 0 | 2 | 0 | 0 | 0 | 0 | 0 | 0 |
| kb743374.1_21339_mature | kb743374.1_21339_mature | ttggagttcagtctgacctt | 1 | 2 | 5 | 6 | 1 | 2 | 5 | 2 |
| kb743188.1_21313_mature | kb743188.1_21313_mature | aaaggacttaatggctgtgacagt | 3 | 3 | 6 | 1 | 0 | 1 | 3 | 4 |
| kb744160.1_15427_mature | kb744160.1_15427_mature | ttcggagtcattgtgctgat | 0 | 3 | 1 | 1 | 0 | 0 | 0 | 0 |
| kb743226.1_4913_mature | kb743226.1_4913_mature | tgaacttttgctgatgtgctggct | 0 | 19 | 9 | 15 | 1 | 2 | 2 | 1 |
| kb743696.1_14044_star | kb743696.1_14044_star | gagtttgtggaacctttcc | 1 | 3 | 0 | 2 | 1 | 1 | 3 | 2 |
| kb745553.1_25354_mature | kb745553.1_25354_mature | tgaggaaggacttgccggtgcggt | 4 | 4 | 11 | 4 | 5 | 2 | 5 | 2 |
| kb742745.1_15181_mature | kb742745.1_15181_mature | tgtggttgtaaacgtgatc | 0 | 2 | 0 | 2 | 0 | 0 | 1 | 3 |
| kb742887.1_1506_mature | kb742887.1_1506_mature | gctgtctgctctgggattttcct | 1 | 7 | 3 | 0 | 2 | 1 | 5 | 0 |
| kb743073.1_9063_star | kb743073.1_9063_star | tacaccagattcgatgac | 0 | 1 | 0 | 0 | 0 | 0 | 0 | 0 |
| kb743713.1_6766_mature | kb743713.1_6766_mature | agttgtttttggaggctggaa | 1 | 3 | 1 | 0 | 0 | 6 | 0 | 0 |
| kb743944.1_1453_star | kb743944.1_1453_star | aaggagtccatcacattcagc | 1 | 2 | 2 | 3 | 1 | 1 | 1 | 2 |
| kb743929.1_17191_mature | kb743929.1_17191_mature | tttgctgtaacaatcttttaga | 1 | 2 | 1 | 0 | 0 | 0 | 2 | 0 |
| kb744413.1_22167_star | kb744413.1_22167_star | tggttgtgtgctggtgtcttggaaa | 1 | 10 | 4 | 9 | 2 | 0 | 4 | 0 |
| kb742646.1_6778_mature | kb742646.1_6778_mature | agcctggatctgttttc | 5 | 0 | 3 | 0 | 0 | 0 | 2 | 3 |
| kb742479.1_272_star | kb742479.1_272_star | agtatcaatgagctgggaattaga | 0 | 0 | 0 | 1 | 0 | 0 | 1 | 0 |
| kb743473.1_22935_mature | kb743473.1_22935_mature | tggacttcctgatcgagc | 0 | 4 | 1 | 1 | 0 | 0 | 8 | 0 |
| kb743291.1_4321_mature | kb743291.1_4321_mature | aacaaaggtagattttagggaggct | 1 | 0 | 0 | 1 | 0 | 3 | 3 | 0 |
| kb743446.1_11681_star | kb743446.1_11681_star | caaggtgtcaatatttctgttaataa | 0 | 2 | 0 | 1 | 0 | 0 | 2 | 0 |
| kb742618.1_11453_mature | kb742618.1_11453_mature | agaattggacagagcaaatc | 0 | 8 | 6 | 0 | 0 | 2 | 2 | 0 |
| kb744022.1_20234_mature | kb744022.1_20234_mature | ctactgtgtgaggaattact | 1 | 0 | 0 | 0 | 0 | 0 | 3 | 2 |
| kb743007.1_12740_mature | kb743007.1_12740_mature | ctgctgtggtgaaactgc | 7 | 10 | 4 | 3 | 6 | 2 | 4 | 1 |
| kb742832.1_11519_mature | kb742832.1_11519_mature | ttggcagaattactggtc | 2 | 1 | 0 | 2 | 1 | 0 | 0 | 0 |
| kb742448.1_5965_mature | kb742448.1_5965_mature | tagggatggatgaaaaaa | 4 | 41 | 0 | 12 | 15 | 2 | 4 | 2 |
| kb744237.1_17842_star | kb744237.1_17842_star | gaagggctgcagtccttcagcaac | 1 | 6 | 15 | 0 | 0 | 0 | 5 | 5 |
| kb742929.1_310_star | kb742929.1_310_star | ttcttctggaagagaaaag | 0 | 1 | 0 | 2 | 2 | 0 | 0 | 0 |
| kb743197.1_1145_star | kb743197.1_1145_star | cattttatttgtacagtttaacta | 1 | 0 | 1 | 0 | 0 | 0 | 3 | 0 |
| kb742479.1_272_mature | kb742479.1_272_mature | tatttcagcacattgacacttt | 0 | 3 | 3 | 2 | 0 | 1 | 2 | 0 |
| kb743284.1_19602_mature | kb743284.1_19602_mature | gactcaaggcctggagacggagc | 0 | 0 | 1 | 7 | 11 | 0 | 5 | 1 |
| kb743963.1_19074_mature | kb743963.1_19074_mature | agattacgttggatgaggcaag | 0 | 0 | 2 | 0 | 2 | 0 | 0 | 4 |
| kb742690.1_5389_star | kb742690.1_5389_star | taactaaagctgtgggaaataaa | 0 | 1 | 3 | 2 | 1 | 0 | 1 | 0 |
| kb742808.1_178_mature | kb742808.1_178_mature | cttgggaggagagcctacagc | 0 | 1 | 0 | 1 | 0 | 2 | 1 | 5 |
| kb746316.1_19656_star | kb746316.1_19656_star | gcgctcccccgccgggac | 0 | 2 | 0 | 1 | 3 | 0 | 0 | 1 |
| kb742933.1_16263_mature | kb742933.1_16263_mature | aaaagaatgctggacctggctct | 3 | 10 | 3 | 1 | 5 | 3 | 4 | 2 |
| kb743282.1_15712_star | kb743282.1_15712_star | tgcactagaagtcatttttgcag | 2 | 2 | 1 | 2 | 0 | 0 | 0 | 3 |
| kb743200.1_11492_mature | kb743200.1_11492_mature | cttcaggctgttcttgt | 0 | 7 | 4 | 3 | 0 | 9 | 5 | 1 |
| kb742579.1_8725_mature | kb742579.1_8725_mature | cttctgtactgtacctcagg | 1 | 0 | 2 | 4 | 0 | 1 | 0 | 0 |
| kb742580.1_2701_mature | kb742580.1_2701_mature | tttggtgtcatcagtaaa | 2 | 2 | 0 | 6 | 8 | 1 | 2 | 2 |
| kb742639.1_10610_star | kb742639.1_10610_star | ttctgagaagtattactcttcaat | 0 | 0 | 1 | 0 | 0 | 0 | 0 | 0 |
| kb752849.1_24272_mature | kb752849.1_24272_mature | agactgcagttcttctgc | 1 | 5 | 8 | 2 | 1 | 1 | 7 | 1 |
| kb744243.1_15689_mature | kb744243.1_15689_mature | aaaactggagctgagcagcc | 4 | 7 | 3 | 12 | 0 | 0 | 7 | 1 |
| kb743692.1_18569_mature | kb743692.1_18569_mature | ctcagtctgtaggcactg | 0 | 0 | 2 | 0 | 0 | 1 | 0 | 3 |
| kb816966.1_25067_mature | kb816966.1_25067_mature | ttttgaactctttagaag | 0 | 3 | 0 | 0 | 0 | 1 | 1 | 0 |
| kb743323.1_3658_star | kb743323.1_3658_star | ttagattatggggaatctttaca | 0 | 0 | 0 | 0 | 1 | 0 | 1 | 0 |
| kb742596.1_13305_mature | kb742596.1_13305_mature | agcctgtagctctgcttggggtt | 38 | 634 | 7 | 658 | 59 | 8 | 19 | 26 |
| kb742563.1_12897_mature | kb742563.1_12897_mature | acgtttgagattctgatc | 0 | 4 | 1 | 2 | 0 | 0 | 1 | 1 |
| kb742928.1_10831_mature | kb742928.1_10831_mature | aaaggatacttagaaaagg | 3 | 59 | 0 | 26 | 1 | 4 | 5 | 0 |
| kb742682.1_9978_star | kb742682.1_9978_star | gtctttgaggtctttgtgtat | 0 | 3 | 2 | 3 | 1 | 2 | 1 | 0 |
| kb742808.1_144_mature | kb742808.1_144_mature | catttagattgcagctgcc | 0 | 0 | 0 | 0 | 0 | 0 | 3 | 0 |
| kb742571.1_4067_mature | kb742571.1_4067_mature | agtttgatggctgtgtgc | 5 | 7 | 3 | 0 | 0 | 1 | 5 | 3 |
| kb742873.1_1674_star | kb742873.1_1674_star | tgtattatcagatctatggggt | 0 | 2 | 2 | 2 | 0 | 0 | 0 | 0 |
| kb742541.1_17016_mature | kb742541.1_17016_mature | aggacagtgacaaggacct | 1 | 1 | 0 | 1 | 4 | 0 | 2 | 0 |
| kb743300.1_12062_mature | kb743300.1_12062_mature | ttcgggcaataaaatggag | 18 | 556 | 1 | 397 | 278 | 5 | 39 | 27 |
| kb742899.1_7111_star | kb742899.1_7111_star | acagtcttgtcttagtcatca | 1 | 3 | 4 | 0 | 1 | 0 | 1 | 0 |
| kb743149.1_16899_star | kb743149.1_16899_star | tgcccgagagtgtttgaagag | 0 | 2 | 1 | 3 | 1 | 0 | 0 | 0 |
| kb742438.1_21875_mature | kb742438.1_21875_mature | tcttgtgtgaaactctatccgcc | 0 | 5 | 1 | 10 | 2 | 0 | 3 | 0 |
| kb743688.1_18774_star | kb743688.1_18774_star | tctgtgtcctgatctgaa | 1 | 3 | 2 | 3 | 2 | 1 | 3 | 2 |
| kb742823.1_13488_mature | kb742823.1_13488_mature | tggcattcagctttccctct | 1 | 0 | 4 | 0 | 2 | 0 | 1 | 1 |
| kb742981.1_12282_star | kb742981.1_12282_star | aattttcctgagatctgt | 4 | 5 | 12 | 1 | 6 | 4 | 8 | 4 |
| kb743642.1_19562_star | kb743642.1_19562_star | cttcaggtattgatgcg | 0 | 0 | 2 | 0 | 0 | 0 | 0 | 0 |
| kb744033.1_9307_mature | kb744033.1_9307_mature | ttgctttcagatacgtagtgct | 0 | 0 | 0 | 2 | 0 | 2 | 4 | 2 |
| kb743110.1_6064_star | kb743110.1_6064_star | gacttggtcctgagagagca | 2 | 0 | 4 | 0 | 2 | 0 | 7 | 0 |
| kb742383.1_14837_star | kb742383.1_14837_star | agcctgaatgacagccggccagtg | 0 | 0 | 1 | 1 | 1 | 1 | 1 | 0 |
| kb743748.1_19045_mature | kb743748.1_19045_mature | ttggacctctctgttcttgcag | 6 | 30 | 15 | 4 | 3 | 2 | 5 | 2 |
| kb744113.1_10662_mature | kb744113.1_10662_mature | ttgtgcatagattcttaat | 0 | 4 | 5 | 1 | 3 | 0 | 8 | 2 |
| kb742711.1_5023_star | kb742711.1_5023_star | tgagagctgactacagctga | 0 | 4 | 0 | 1 | 1 | 2 | 0 | 1 |
| kb742745.1_15183_star | kb742745.1_15183_star | aggttggtttctctcctttattca | 0 | 1 | 0 | 2 | 0 | 1 | 0 | 0 |
| kb743176.1_3940_mature | kb743176.1_3940_mature | tgttctcaggggcatgtaacttg | 0 | 1 | 6 | 0 | 0 | 0 | 1 | 0 |
| kb743153.1_6808_mature | kb743153.1_6808_mature | aggaagatcgtgtgatgtc | 0 | 1 | 0 | 0 | 0 | 1 | 4 | 1 |
| kb743291.1_4381_star | kb743291.1_4381_star | atggttattactttcct | 0 | 0 | 1 | 0 | 0 | 0 | 0 | 1 |
| kb742418.1_2497_mature | kb742418.1_2497_mature | taggttctgggattctttcc | 0 | 1 | 6 | 1 | 0 | 2 | 0 | 1 |
| kb743686.1_3434_mature | kb743686.1_3434_mature | tgcagagctctgccctg | 0 | 1 | 2 | 0 | 0 | 0 | 2 | 0 |
| kb742811.1_13103_mature | kb742811.1_13103_mature | tgagtactgtggtcagttttgggcc | 31 | 129 | 2 | 195 | 44 | 1 | 24 | 3 |
| kb743228.1_14930_mature | kb743228.1_14930_mature | aacagaggcaaagaaggca | 0 | 6 | 0 | 2 | 1 | 0 | 3 | 0 |
| kb743062.1_19332_mature | kb743062.1_19332_mature | actgcaactctgtacttccag | 0 | 3 | 1 | 2 | 0 | 0 | 4 | 2 |
| kb744228.1_23280_mature | kb744228.1_23280_mature | aacgtgctggggcttggccta | 4 | 3 | 4 | 6 | 2 | 3 | 3 | 1 |
| kb742393.1_17350_star | kb742393.1_17350_star | gtatctggtgcctgaggtctgg | 0 | 0 | 1 | 0 | 2 | 0 | 0 | 1 |
| kb743004.1_8918_mature | kb743004.1_8918_mature | gaagtatggagagttttga | 0 | 1 | 3 | 1 | 0 | 0 | 2 | 1 |
| kb743366.1_17996_star | kb743366.1_17996_star | ggggaccatctttacgtctta | 0 | 0 | 0 | 0 | 0 | 0 | 1 | 0 |
| kb745238.1_20000_mature | kb745238.1_20000_mature | catccgggatcgagttgctacgg | 0 | 1 | 3 | 1 | 0 | 0 | 0 | 1 |
| kb742382.1_14228_mature | kb742382.1_14228_mature | actggaggtggagcaggct | 5 | 4 | 24 | 1 | 3 | 0 | 2 | 5 |
| kb744105.1_20131_star | kb744105.1_20131_star | cttctccaccaacccttcccagg | 0 | 2 | 0 | 1 | 1 | 0 | 1 | 0 |
| kb742497.1_4727_mature | kb742497.1_4727_mature | gtatttctagagcttattgctgg | 3 | 0 | 0 | 0 | 0 | 1 | 0 | 0 |
| kb743203.1_21095_star | kb743203.1_21095_star | agcatcacatcttcctccagt | 1 | 3 | 2 | 0 | 1 | 1 | 3 | 2 |
| kb743091.1_21604_star | kb743091.1_21604_star | gaggggcgccaggcccgtct | 0 | 2 | 3 | 1 | 2 | 2 | 3 | 4 |
| kb742957.1_4306_star | kb742957.1_4306_star | gtaagagattcctagtt | 0 | 0 | 0 | 0 | 2 | 0 | 0 | 0 |
| kb743197.1_1142_star | kb743197.1_1142_star | tgtaaaatactgagcagga | 2 | 2 | 1 | 0 | 0 | 1 | 3 | 0 |
| kb746064.1_19104_mature | kb746064.1_19104_mature | ggggctggagagatggct | 2 | 2 | 2 | 12 | 3 | 0 | 2 | 3 |
| kb742466.1_4845_mature | kb742466.1_4845_mature | ttgaagaaagtgtgaagtgaaa | 2 | 7 | 2 | 3 | 2 | 1 | 1 | 1 |
| kb769248.1_25969_star | kb769248.1_25969_star | agagaggtgcagaaggaa | 0 | 2 | 1 | 0 | 0 | 2 | 0 | 0 |
| kb742459.1_3538_mature | kb742459.1_3538_mature | aggatagatgttagaactact | 3 | 0 | 0 | 3 | 0 | 1 | 0 | 0 |
| kb746632.1_16657_mature | kb746632.1_16657_mature | ggcatgttggaacaatgt | 1 | 5 | 3 | 4 | 0 | 1 | 2 | 2 |
| kb815851.1_25129_mature | kb815851.1_25129_mature | taggattgttagtagtagct | 0 | 0 | 0 | 2 | 0 | 2 | 2 | 0 |
| kb742957.1_4292_mature | kb742957.1_4292_mature | ttgtaatgatctgtgtgaaatctac | 3 | 12 | 5 | 10 | 2 | 6 | 20 | 2 |
| kb743302.1_19391_star | kb743302.1_19391_star | cgtacctccatcctgc | 0 | 0 | 2 | 0 | 0 | 0 | 0 | 0 |
| kb743204.1_4253_mature | kb743204.1_4253_mature | aacagaggcaaagaaggcatt | 2 | 25 | 0 | 24 | 0 | 0 | 0 | 1 |
| kb742711.1_5019_mature | kb742711.1_5019_mature | tgtaaatcatgtaactctcttgc | 0 | 1 | 2 | 3 | 1 | 1 | 4 | 3 |
| kb742483.1_1719_star | kb742483.1_1719_star | tagacagtggcagtgatgtgtcgtagt | 0 | 0 | 0 | 3 | 3 | 2 | 1 | 0 |
| kb743210.1_15329_star | kb743210.1_15329_star | attttcaggatgttttag | 0 | 2 | 0 | 0 | 0 | 0 | 0 | 0 |
| kb743343.1_14285_star | kb743343.1_14285_star | cacccccaacacatgccagcac | 0 | 0 | 1 | 0 | 0 | 1 | 0 | 0 |
| kb789915.1_25765_mature | kb789915.1_25765_mature | cggtgctggtgatctacc | 0 | 2 | 2 | 2 | 2 | 3 | 0 | 1 |
| kb743275.1_2674_mature | kb743275.1_2674_mature | tatctcactgttgaaatgcaga | 1 | 5 | 8 | 2 | 3 | 1 | 0 | 1 |
| kb743458.1_14255_mature | kb743458.1_14255_mature | atgtctgtgtggatttcc | 0 | 3 | 3 | 2 | 0 | 1 | 4 | 0 |
| kb743897.1_19285_star | kb743897.1_19285_star | gcgcaggagctgcatgtaggtg | 0 | 0 | 0 | 0 | 1 | 0 | 0 | 0 |
| kb742781.1_1892_star | kb742781.1_1892_star | cttggatgcctcctggca | 0 | 1 | 1 | 0 | 0 | 0 | 0 | 0 |
| kb742873.1_1686_mature | kb742873.1_1686_mature | taacgtctgcagctaacccttc | 0 | 1 | 0 | 0 | 1 | 0 | 4 | 0 |
| kb742668.1_5611_star | kb742668.1_5611_star | agtgccacctgtaatacatttatcacaa | 0 | 0 | 1 | 0 | 2 | 0 | 0 | 0 |
| kb744435.1_16038_mature | kb744435.1_16038_mature | ctgggctgtgccagttgcac | 0 | 1 | 1 | 0 | 0 | 0 | 2 | 2 |
| kb743446.1_11689_mature | kb743446.1_11689_mature | ggatttactgtagtttgt | 0 | 4 | 0 | 0 | 0 | 2 | 0 | 0 |
| kb742934.1_13228_star | kb742934.1_13228_star | tttattggaataagttgaaacta | 0 | 0 | 2 | 0 | 0 | 1 | 1 | 0 |
| kb742833.1_47_star | kb742833.1_47_star | agaaaaatcagaccatc | 2 | 2 | 0 | 0 | 3 | 0 | 4 | 0 |
| kb789254.1_25778_mature | kb789254.1_25778_mature | ctgtttcggcagagcta | 35 | 64 | 169 | 19 | 64 | 18 | 52 | 24 |
| kb744353.1_15001_star | kb744353.1_15001_star | cagaccttattttggcaaaact | 1 | 2 | 0 | 0 | 1 | 0 | 0 | 0 |
| kb742448.1_5971_star | kb742448.1_5971_star | attgtcacccaggtgtgc | 0 | 3 | 0 | 4 | 1 | 0 | 3 | 0 |
| kb742499.1_8255_star | kb742499.1_8255_star | gggtgacgttctgccaagaa | 0 | 4 | 0 | 2 | 0 | 0 | 4 | 0 |
| kb742582.1_7371_star | kb742582.1_7371_star | ctgctcctttctgctccacggt | 0 | 0 | 0 | 3 | 1 | 0 | 2 | 0 |
| kb745110.1_19011_mature | kb745110.1_19011_mature | tctaactgtaatggtatctgga | 0 | 0 | 0 | 0 | 0 | 0 | 3 | 0 |
| kb742873.1_1641_star | kb742873.1_1641_star | ccctggcagatggttttaat | 0 | 0 | 1 | 0 | 0 | 0 | 0 | 0 |
| kb742490.1_1402_mature | kb742490.1_1402_mature | agtgaatgtttgaactggatgag | 0 | 9 | 6 | 1 | 3 | 4 | 6 | 1 |
| kb742652.1_6562_star | kb742652.1_6562_star | ctgctttctataaacgctt | 1 | 5 | 1 | 2 | 3 | 2 | 2 | 1 |
| kb743374.1_21335_mature | kb743374.1_21335_mature | tgtgattctaggatgctcagc | 1 | 3 | 4 | 1 | 0 | 2 | 1 | 0 |
| kb742758.1_20730_mature | kb742758.1_20730_mature | gacattttaaaaatggacaggt | 3 | 0 | 2 | 0 | 0 | 1 | 1 | 1 |
| kb742677.1_7512_mature | kb742677.1_7512_mature | aggctgcaagatggatgc | 3 | 2 | 7 | 0 | 1 | 1 | 3 | 0 |
| kb804379.1_25492_mature | kb804379.1_25492_mature | aatctaagatgactgggcaccta | 0 | 0 | 0 | 1 | 1 | 0 | 3 | 0 |
| kb743740.1_8890_star | kb743740.1_8890_star | tattcttccctaatgctgt | 1 | 2 | 1 | 1 | 1 | 0 | 0 | 2 |
| kb743108.1_15446_star | kb743108.1_15446_star | gcaaggggcaaacagagcaa | 0 | 0 | 1 | 0 | 0 | 0 | 0 | 0 |
| kb743435.1_2983_star | kb743435.1_2983_star | aaagcactgagtcccaacaaat | 0 | 4 | 1 | 0 | 2 | 2 | 1 | 2 |
| kb743520.1_3890_star | kb743520.1_3890_star | tgctgagttccttcaggcagtct | 6 | 16 | 12 | 4 | 2 | 3 | 5 | 2 |
| kb743037.1_11817_star | kb743037.1_11817_star | taggttaacaacatggatgcatta | 0 | 1 | 2 | 0 | 0 | 0 | 0 | 0 |
| kb743278.1_18082_mature | kb743278.1_18082_mature | tgactgggatctgatctctgccc | 3 | 11 | 3 | 8 | 2 | 0 | 3 | 2 |
| kb743771.1_8033_star | kb743771.1_8033_star | taaaagcaagtgttactgcttaag | 0 | 2 | 0 | 1 | 0 | 1 | 0 | 0 |
| kb742652.1_6522_star | kb742652.1_6522_star | acgagaacaagcactgat | 0 | 0 | 0 | 0 | 0 | 1 | 0 | 0 |
| kb807964.1_25406_mature | kb807964.1_25406_mature | tggcgtgctttctctcctcagc | 0 | 4 | 2 | 6 | 1 | 5 | 2 | 1 |
| kb742605.1_4570_mature | kb742605.1_4570_mature | tggacagtgttgcgaatgc | 0 | 3 | 2 | 2 | 1 | 0 | 1 | 0 |
| kb742444.1_8011_mature | kb742444.1_8011_mature | tgtaatttctgtatgaacttca | 0 | 8 | 2 | 7 | 3 | 2 | 5 | 0 |
| kb742725.1_13853_star | kb742725.1_13853_star | aactacaagtgaaggaggactggtaa | 3 | 5 | 6 | 2 | 2 | 1 | 1 | 2 |
| kb743833.1_10826_star | kb743833.1_10826_star | gtctctcactccatcccacc | 0 | 0 | 2 | 0 | 0 | 1 | 0 | 1 |
| kb742745.1_15184_mature | kb742745.1_15184_mature | atcaaaggaatagagcacctct | 100 | 101 | 95 | 189 | 184 | 176 | 119 | 123 |
| kb743584.1_13901_mature | kb743584.1_13901_mature | atgataaagtctctgtaaagttt | 0 | 1 | 2 | 0 | 0 | 0 | 4 | 4 |
| kb742678.1_15752_mature | kb742678.1_15752_mature | tggggcgggaggttgggg | 1 | 0 | 4 | 2 | 0 | 1 | 1 | 3 |
| kb742777.1_8208_mature | kb742777.1_8208_mature | gctgctcgggctgccggc | 0 | 1 | 0 | 3 | 1 | 0 | 2 | 0 |
| kb743275.1_2652_mature | kb743275.1_2652_mature | aggttccttgcctctgagca | 1 | 5 | 2 | 1 | 0 | 1 | 1 | 0 |
| kb742432.1_461_star | kb742432.1_461_star | gctatcttccacactctccagaa | 1 | 13 | 3 | 11 | 1 | 1 | 3 | 4 |
| kb742598.1_14469_star | kb742598.1_14469_star | tgaagatccaagcaggtac | 0 | 0 | 0 | 0 | 0 | 0 | 1 | 0 |
| kb742575.1_12387_mature | kb742575.1_12387_mature | aggttttgaatgaagaatgcg | 0 | 3 | 3 | 1 | 1 | 0 | 7 | 0 |
| kb744187.1_24329_star | kb744187.1_24329_star | agcctttcctcggttgct | 0 | 1 | 0 | 0 | 0 | 0 | 0 | 0 |
| kb743569.1_19234_star | kb743569.1_19234_star | aaaggtactgatttttaagaaga | 0 | 0 | 0 | 1 | 0 | 0 | 0 | 0 |
| kb742807.1_16446_mature | kb742807.1_16446_mature | tgggcactgtgaccaaggcacg | 1 | 4 | 1 | 0 | 0 | 0 | 1 | 1 |
| kb742671.1_7159_mature | kb742671.1_7159_mature | aattgagtactggacttggg | 2 | 8 | 0 | 5 | 0 | 0 | 5 | 0 |
| kb743787.1_22728_star | kb743787.1_22728_star | gatcggttgggactgggc | 0 | 2 | 0 | 0 | 0 | 1 | 0 | 0 |
| kb744198.1_12200_mature | kb744198.1_12200_mature | tagcatgacgccagatggg | 37 | 184 | 0 | 272 | 55 | 3 | 8 | 4 |
| kb744677.1_24324_mature | kb744677.1_24324_mature | tgacggcctctttctctttgc | 0 | 3 | 1 | 1 | 0 | 1 | 2 | 1 |
| kb743458.1_14267_star | kb743458.1_14267_star | gttcagttgcaacgttctttaga | 0 | 0 | 0 | 0 | 0 | 0 | 2 | 1 |
| kb746572.1_23773_star | kb746572.1_23773_star | agaggaggcagccagcaccttc | 2 | 0 | 2 | 1 | 3 | 1 | 6 | 2 |
| kb742744.1_6102_star | kb742744.1_6102_star | tcaggtgagcagagcttt | 0 | 1 | 2 | 4 | 0 | 0 | 0 | 0 |
| kb742682.1_9978_mature | kb742682.1_9978_mature | acatctactgaccagagctt | 0 | 0 | 0 | 3 | 1 | 1 | 1 | 2 |
| kb756629.1_24623_star | kb756629.1_24623_star | ctcagtacacatcctgg | 0 | 0 | 1 | 9 | 0 | 1 | 1 | 0 |
| kb742714.1_4146_star | kb742714.1_4146_star | atgtgccactgagtcactcgggca | 0 | 0 | 3 | 0 | 0 | 0 | 1 | 0 |
| kb744033.1_9323_star | kb744033.1_9323_star | gctgccagggacacagctgct | 0 | 0 | 3 | 0 | 1 | 0 | 1 | 1 |
| kb742830.1_16496_mature | kb742830.1_16496_mature | ctgctgtctccagctgtcgagctgt | 0 | 5 | 1 | 5 | 0 | 0 | 1 | 1 |
| kb742520.1_11482_mature | kb742520.1_11482_mature | cttgctggtctctgaaca | 2 | 0 | 2 | 2 | 1 | 1 | 0 | 1 |
| kb744188.1_22206_star | kb744188.1_22206_star | ttggtctggacacactgcttgt | 0 | 10 | 1 | 5 | 1 | 4 | 8 | 1 |
| kb744033.1_9316_mature | kb744033.1_9316_mature | gtattgcctgttctagagaact | 1 | 4 | 1 | 3 | 2 | 4 | 0 | 4 |
| kb743348.1_22030_mature | kb743348.1_22030_mature | gggaagctgccctcagagg | 3 | 49 | 0 | 58 | 7 | 1 | 23 | 0 |
| kb742595.1_8379_star | kb742595.1_8379_star | ttaacgcagaaggacaggcat | 0 | 0 | 2 | 0 | 1 | 0 | 0 | 0 |
| kb742446.1_12978_mature | kb742446.1_12978_mature | tggcagaacagggacttcgctg | 0 | 3 | 1 | 2 | 0 | 0 | 4 | 0 |
| kb744073.1_20230_star | kb744073.1_20230_star | aagttggtcgggcactgacacaggc | 0 | 3 | 0 | 5 | 0 | 0 | 1 | 1 |
| kb744332.1_17321_mature | kb744332.1_17321_mature | tggcagaattctcgcct | 240 | 874 | 669 | 510 | 181 | 159 | 588 | 269 |
| kb743356.1_12151_star | kb743356.1_12151_star | tgcaacatcttcagcagagttttaa | 3 | 7 | 4 | 1 | 0 | 3 | 6 | 7 |
| kb743217.1_9785_mature | kb743217.1_9785_mature | aggctgttggaagacgac | 2 | 7 | 2 | 3 | 0 | 2 | 1 | 2 |
| kb742616.1_12591_mature | kb742616.1_12591_mature | agctaggcctggggctcccag | 0 | 0 | 0 | 2 | 0 | 0 | 1 | 0 |
| kb742778.1_14681_mature | kb742778.1_14681_mature | cttctgatgtggttatggcc | 3 | 0 | 1 | 0 | 2 | 0 | 5 | 0 |
| kb742955.1_12835_mature | kb742955.1_12835_mature | gatggttaaggatctgca | 1 | 1 | 4 | 2 | 1 | 0 | 0 | 0 |
| kb742418.1_2476_star | kb742418.1_2476_star | aaggattttttgtacttatttttg | 3 | 5 | 2 | 2 | 1 | 2 | 3 | 0 |
| kb744588.1_17421_star | kb744588.1_17421_star | tagtgtttctatttccatatt | 0 | 2 | 0 | 3 | 3 | 1 | 3 | 0 |
| kb742651.1_5123_star | kb742651.1_5123_star | cccccaacagaataaaac | 0 | 0 | 0 | 3 | 0 | 1 | 0 | 0 |
| kb743556.1_17227_mature | kb743556.1_17227_mature | agtagtgctcttatttaagt | 0 | 1 | 2 | 0 | 3 | 0 | 1 | 0 |
| kb742896.1_17390_star | kb742896.1_17390_star | actcccctttgatttcagagca | 8 | 19 | 4 | 10 | 8 | 3 | 6 | 5 |
| kb743106.1_6128_mature | kb743106.1_6128_mature | ctgatgactgctctggca | 2 | 5 | 0 | 2 | 3 | 2 | 5 | 1 |
| kb742515.1_4128_mature | kb742515.1_4128_mature | gggacagacgtgggaaggga | 0 | 0 | 1 | 0 | 0 | 0 | 4 | 0 |
| kb742714.1_4176_mature | kb742714.1_4176_mature | ttaaatatggctggagagtggt | 0 | 1 | 6 | 0 | 2 | 1 | 1 | 0 |
| kb742391.1_14704_mature | kb742391.1_14704_mature | ttgtgatctgtaaatgtttat | 2 | 5 | 1 | 2 | 0 | 1 | 5 | 1 |
| kb743395.1_12822_star | kb743395.1_12822_star | ccctgctgctaatcctcca | 1 | 0 | 3 | 0 | 0 | 1 | 0 | 2 |
| kb744020.1_10239_star | kb744020.1_10239_star | gaatacagtagtttttga | 2 | 0 | 0 | 1 | 0 | 0 | 0 | 0 |
| kb743211.1_11241_mature | kb743211.1_11241_mature | tgctgctagactgtgagtc | 0 | 4 | 2 | 0 | 2 | 1 | 5 | 0 |
| kb742451.1_6326_mature | kb742451.1_6326_mature | taacaactgtcctctaaccatc | 2 | 5 | 4 | 6 | 3 | 0 | 3 | 2 |
| kb744000.1_19343_mature | kb744000.1_19343_mature | tgtatttccttgcagtt | 1 | 2 | 1 | 2 | 0 | 1 | 0 | 0 |
| kb752565.1_23337_star | kb752565.1_23337_star | tggtgcagcccccatcc | 0 | 0 | 1 | 0 | 0 | 0 | 1 | 1 |
| kb742931.1_949_mature | kb742931.1_949_mature | aaaaaaggactgagaag | 1 | 6 | 1 | 1 | 0 | 0 | 2 | 0 |
| kb742558.1_23296_mature | kb742558.1_23296_mature | tggtctggctgtaagaggcatg | 1 | 2 | 1 | 7 | 0 | 0 | 2 | 0 |
| kb742466.1_4810_mature | kb742466.1_4810_mature | ccccctctgctgcaagtctggaaga | 2 | 5 | 2 | 1 | 2 | 0 | 2 | 1 |
| kb742985.1_12952_star | kb742985.1_12952_star | cccagctgcttgtttatttgaagg | 0 | 1 | 1 | 0 | 1 | 0 | 1 | 0 |
| kb742907.1_4742_star | kb742907.1_4742_star | gcagttagaaacactacaa | 1 | 2 | 2 | 1 | 2 | 0 | 1 | 0 |
| kb742712.1_8641_mature | kb742712.1_8641_mature | ctctgctgtgcatccaccagc | 0 | 1 | 4 | 1 | 1 | 3 | 1 | 1 |
| kb743136.1_7894_mature | kb743136.1_7894_mature | tgatttgtgctctggggctttttgt | 3 | 2 | 7 | 0 | 5 | 0 | 4 | 3 |
| kb743374.1_21339_star | kb743374.1_21339_star | taaaggaaaactacaagc | 0 | 0 | 0 | 2 | 0 | 0 | 1 | 0 |
| kb743085.1_10571_star | kb743085.1_10571_star | atgtatgaacagattt | 3 | 11 | 8 | 3 | 6 | 3 | 7 | 6 |
| kb742616.1_12585_mature | kb742616.1_12585_mature | aacatggtctctgaacagtcag | 5 | 6 | 11 | 2 | 4 | 6 | 8 | 0 |
| kb746255.1_23677_star | kb746255.1_23677_star | cagcattgtgctgggtcctg | 1 | 0 | 3 | 0 | 0 | 1 | 0 | 1 |
| kb743139.1_2227_star | kb743139.1_2227_star | tagcaaagcctacaaccacggtgcag | 5 | 7 | 8 | 0 | 4 | 2 | 7 | 1 |
| kb742992.1_5523_mature | kb742992.1_5523_mature | gtgcctgaagactggagga | 1 | 38 | 2 | 51 | 1 | 0 | 0 | 0 |
| kb743046.1_19354_mature | kb743046.1_19354_mature | ccgtgaccagcagcttgtgtt | 0 | 0 | 3 | 1 | 0 | 1 | 5 | 2 |
| kb742481.1_5270_star | kb742481.1_5270_star | ccagttcctcggtaatgttg | 0 | 1 | 0 | 1 | 0 | 1 | 0 | 0 |
| kb742811.1_13116_mature | kb742811.1_13116_mature | cacacctgcccagggatg | 0 | 0 | 3 | 0 | 0 | 0 | 0 | 1 |
| kb742690.1_5382_mature | kb742690.1_5382_mature | tctttttaagactgtgta | 5 | 19 | 12 | 10 | 0 | 10 | 34 | 1 |
| kb742733.1_1317_mature | kb742733.1_1317_mature | atttgctgactgaaatgc | 0 | 2 | 0 | 1 | 0 | 0 | 7 | 1 |
| kb744831.1_24169_mature | kb744831.1_24169_mature | agcggggtcaggacgtggcacggtc | 7 | 4 | 2 | 8 | 8 | 2 | 5 | 5 |
| kb785238.1_25838_star | kb785238.1_25838_star | accgcttccgaaagagggacaagg | 0 | 0 | 0 | 0 | 1 | 0 | 0 | 0 |
| kb742808.1_191_star | kb742808.1_191_star | tatttaaagattaaaaaga | 0 | 1 | 0 | 0 | 0 | 0 | 1 | 0 |
| kb743146.1_13252_star | kb743146.1_13252_star | cagctgctcaggaaagt | 0 | 3 | 1 | 3 | 1 | 2 | 1 | 1 |
| kb742602.1_14852_star | kb742602.1_14852_star | gttagcttgcagccgcctccct | 1 | 2 | 0 | 1 | 1 | 1 | 1 | 0 |
| kb746071.1_19970_star | kb746071.1_19970_star | cagtgggcaccactgaaaat | 1 | 0 | 0 | 1 | 0 | 0 | 1 | 1 |
| kb746316.1_19656_mature | kb746316.1_19656_mature | cctgctgggactcagcta | 0 | 14 | 3 | 1 | 3 | 0 | 1 | 5 |
| kb744204.1_11867_star | kb744204.1_11867_star | aagaagttgagcaggacctaatcaaac | 1 | 0 | 4 | 1 | 1 | 4 | 1 | 0 |
| kb742652.1_6555_star | kb742652.1_6555_star | ctgcaacaaaggatgtcatgc | 1 | 2 | 0 | 0 | 1 | 1 | 1 | 0 |
| kb744706.1_23007_mature | kb744706.1_23007_mature | ccactatatgtactcgta | 0 | 0 | 0 | 0 | 3 | 0 | 0 | 0 |
| kb742851.1_13581_mature | kb742851.1_13581_mature | accgtgctcgtgtcctggcaga | 4 | 3 | 5 | 2 | 1 | 2 | 1 | 0 |
| kb742794.1_18829_star | kb742794.1_18829_star | ttgtccttggctttgcttagtcttct | 0 | 1 | 0 | 0 | 1 | 0 | 2 | 0 |
| kb743963.1_19074_star | kb743963.1_19074_star | tgctaaagaggagcgtgtttct | 0 | 3 | 1 | 0 | 0 | 0 | 0 | 0 |
| kb742878.1_11100_star | kb742878.1_11100_star | tgtcagaagctaaagtttgag | 6 | 5 | 6 | 7 | 2 | 4 | 6 | 2 |
| kb742784.1_10912_mature | kb742784.1_10912_mature | tcctgagagtcggagggct | 0 | 0 | 0 | 0 | 0 | 2 | 2 | 0 |
| kb742712.1_8613_mature | kb742712.1_8613_mature | agtgcgagaacttaaaca | 28 | 78 | 61 | 129 | 21 | 21 | 30 | 45 |
| kb742393.1_17348_mature | kb742393.1_17348_mature | attgtccattgtgtcagcttgc | 0 | 2 | 1 | 1 | 2 | 1 | 3 | 0 |
| kb743098.1_20648_mature | kb743098.1_20648_mature | ttacagcatgcgtttctgag | 0 | 0 | 0 | 0 | 0 | 0 | 3 | 0 |
| kb742392.1_20512_star | kb742392.1_20512_star | ttcagaagctggcaggtaag | 0 | 1 | 1 | 1 | 0 | 0 | 1 | 0 |
| kb744068.1_20022_star | kb744068.1_20022_star | gtcttctccagttcaccc | 0 | 11 | 2 | 35 | 6 | 2 | 1 | 4 |
| kb742664.1_9855_mature | kb742664.1_9855_mature | ccctgttttagaacacttcagtgat | 0 | 1 | 1 | 1 | 1 | 0 | 5 | 3 |
| kb742668.1_5624_star | kb742668.1_5624_star | tgtttaattttactgtga | 0 | 0 | 0 | 0 | 1 | 0 | 1 | 0 |
| kb743395.1_12822_mature | kb743395.1_12822_mature | tgggatctggctactttggct | 1 | 3 | 0 | 1 | 0 | 2 | 1 | 1 |
| kb744900.1_18370_mature | kb744900.1_18370_mature | acatgcttgcaggactgtaagt | 3 | 5 | 8 | 2 | 2 | 5 | 0 | 3 |
| kb742701.1_6478_star | kb742701.1_6478_star | ttccatcaggctcaaaaatt | 0 | 1 | 0 | 1 | 0 | 0 | 1 | 0 |
| kb742511.1_13667_star | kb742511.1_13667_star | acccaggaggtgggggaccagt | 1 | 1 | 0 | 3 | 0 | 2 | 4 | 2 |
| kb742736.1_12027_star | kb742736.1_12027_star | gcaggtgggaggcagcagag | 0 | 2 | 1 | 1 | 1 | 1 | 2 | 0 |
| kb743581.1_20330_star | kb743581.1_20330_star | tccccccagccgggcg | 0 | 0 | 0 | 0 | 1 | 2 | 0 | 0 |
| kb743297.1_21459_star | kb743297.1_21459_star | atgaatgaataggaacatc | 1 | 2 | 0 | 1 | 0 | 0 | 2 | 0 |
| kb742678.1_15752_star | kb742678.1_15752_star | tgtgctgctcgctgccagg | 0 | 1 | 1 | 0 | 0 | 0 | 1 | 1 |
| kb743529.1_12855_star | kb743529.1_12855_star | tgccttgtgattatctccta | 0 | 0 | 0 | 0 | 0 | 1 | 0 | 0 |
| kb744068.1_20022_mature | kb744068.1_20022_mature | aagggctcggagaaggcac | 0 | 1 | 1 | 7 | 3 | 0 | 3 | 0 |
| kb743228.1_14928_star | kb743228.1_14928_star | aggagccacagagaccacagaagac | 2 | 4 | 3 | 0 | 2 | 3 | 2 | 3 |
| kb743403.1_19668_star | kb743403.1_19668_star | gtgcccccctgccctcctc | 1 | 3 | 7 | 4 | 3 | 2 | 0 | 11 |
| kb742725.1_13866_star | kb742725.1_13866_star | cttgtattattttcagggca | 0 | 0 | 3 | 0 | 0 | 0 | 0 | 0 |
| kb742464.1_2369_star | kb742464.1_2369_star | ggtcccatccatgaaa | 0 | 0 | 1 | 0 | 0 | 0 | 0 | 0 |
| kb743509.1_7190_mature | kb743509.1_7190_mature | aactgtatgtttttcttgc | 0 | 0 | 0 | 2 | 2 | 0 | 1 | 0 |
| kb743810.1_19135_mature | kb743810.1_19135_mature | ctgggccgggacgaggaggg | 4 | 1 | 2 | 5 | 1 | 1 | 1 | 3 |
| kb743090.1_9113_mature | kb743090.1_9113_mature | agttatgagacttatgagtc | 0 | 3 | 1 | 0 | 0 | 0 | 0 | 0 |
| kb743948.1_17921_mature | kb743948.1_17921_mature | cgcctcgtaggctctgcccggc | 0 | 2 | 7 | 2 | 1 | 0 | 1 | 2 |
| kb743783.1_22087_mature | kb743783.1_22087_mature | atcggtgccattggtctgcctcc | 1 | 4 | 3 | 1 | 0 | 0 | 1 | 0 |
| kb743348.1_22030_star | kb743348.1_22030_star | gctgaaacgaggcttttccccc | 2 | 0 | 0 | 0 | 0 | 0 | 0 | 0 |
| kb742388.1_5741_star | kb742388.1_5741_star | gagatctggcagaggagaa | 0 | 0 | 0 | 0 | 1 | 1 | 1 | 0 |
| kb742736.1_12027_mature | kb742736.1_12027_mature | ctgattgatgagaccgtcctgctt | 2 | 1 | 0 | 0 | 1 | 1 | 4 | 0 |
| kb813882.1_25219_mature | kb813882.1_25219_mature | agtgcctgaagacgatgaat | 3 | 1 | 0 | 2 | 0 | 0 | 1 | 0 |
| kb742558.1_23296_star | kb742558.1_23296_star | tgtccgaggcagacacacg | 0 | 0 | 3 | 0 | 0 | 0 | 0 | 0 |
| kb742617.1_6182_star | kb742617.1_6182_star | attttctgttccctctct | 1 | 1 | 0 | 1 | 0 | 1 | 1 | 0 |
| kb742937.1_7573_star | kb742937.1_7573_star | aacgtggccgaaaataagcaaa | 0 | 1 | 0 | 1 | 0 | 0 | 0 | 1 |
| kb743670.1_4933_mature | kb743670.1_4933_mature | gagttgagacgttgctgtggc | 0 | 1 | 3 | 0 | 0 | 1 | 0 | 0 |
| kb742432.1_461_mature | kb742432.1_461_mature | ctgggctgtgttgtgctt | 105 | 280 | 3 | 467 | 96 | 0 | 15 | 5 |
| kb745238.1_19989_mature | kb745238.1_19989_mature | tcctgcttgacgagcctga | 1 | 5 | 1 | 7 | 12 | 0 | 0 | 5 |
| kb742931.1_922_mature | kb742931.1_922_mature | tccctgaattatgtggtagcct | 4 | 18 | 16 | 7 | 3 | 2 | 10 | 4 |
| kb744831.1_24169_star | kb744831.1_24169_star | cccctgctccatcccgggccgtgctgg | 0 | 0 | 3 | 0 | 0 | 2 | 2 | 3 |
| kb744429.1_15131_star | kb744429.1_15131_star | ggaaggcagggataggtaatgaat | 14 | 39 | 1 | 19 | 22 | 3 | 1 | 2 |
| kb743948.1_17921_star | kb743948.1_17921_star | cgggcggagcctgcgagggagc | 0 | 1 | 1 | 2 | 1 | 2 | 0 | 3 |
| kb743153.1_6790_star | kb743153.1_6790_star | tagcctgtctgtaaacta | 0 | 3 | 1 | 1 | 0 | 0 | 1 | 0 |
| kb742993.1_11354_mature | kb742993.1_11354_mature | tttgaagactgaacattt | 3 | 2 | 2 | 0 | 4 | 1 | 4 | 0 |
| kb743167.1_13284_mature | kb743167.1_13284_mature | gtgtctctgtgcttctgaatgaa | 5 | 3 | 5 | 2 | 2 | 2 | 3 | 1 |
| kb743814.1_11941_star | kb743814.1_11941_star | tctgtatcactcagtt | 0 | 0 | 1 | 1 | 0 | 1 | 0 | 0 |
| kb744074.1_11226_star | kb744074.1_11226_star | tcctgcccttccctga | 0 | 1 | 0 | 3 | 0 | 0 | 0 | 0 |
| kb744558.1_15598_star | kb744558.1_15598_star | agcaggaagggtagagagtg | 0 | 1 | 1 | 0 | 0 | 0 | 0 | 0 |
| kb744020.1_10268_mature | kb744020.1_10268_mature | ctgcttctctacaacttcacagg | 4 | 3 | 1 | 5 | 1 | 5 | 7 | 0 |
| kb742736.1_11989_mature | kb742736.1_11989_mature | agcggggtgatgagacactgt | 3 | 12 | 7 | 4 | 8 | 0 | 3 | 3 |
| kb743435.1_2983_mature | kb743435.1_2983_mature | ttatgctcctgaatgcctttgg | 13 | 22 | 13 | 30 | 14 | 12 | 11 | 1 |
| kb743505.1_24117_mature | kb743505.1_24117_mature | ccatgtccctgtccccacagt | 1 | 1 | 2 | 3 | 2 | 1 | 1 | 0 |
| kb746564.1_24909_star | kb746564.1_24909_star | ggtctcagcgtgtccgggtctca | 0 | 3 | 2 | 1 | 2 | 0 | 0 | 0 |
| kb742629.1_532_mature | kb742629.1_532_mature | cacaggcttctccatgtaga | 5 | 1 | 2 | 2 | 2 | 2 | 2 | 1 |
| kb744395.1_20615_mature | kb744395.1_20615_mature | ccagcgctttggtgacagtggatg | 1 | 4 | 0 | 2 | 1 | 0 | 1 | 0 |
| kb742616.1_12591_star | kb742616.1_12591_star | aagagctttaaggcattcctct | 1 | 1 | 1 | 0 | 0 | 0 | 1 | 0 |
| kb743963.1_19072_mature | kb743963.1_19072_mature | aatgaaacttgggaagaaatctta | 2 | 6 | 6 | 3 | 4 | 2 | 9 | 4 |
| kb742418.1_2435_star | kb742418.1_2435_star | gaggaggagagagggaggaggg | 2 | 11 | 12 | 8 | 8 | 7 | 4 | 6 |
| kb745135.1_22103_mature | kb745135.1_22103_mature | atgctaggcaatggactgt | 0 | 0 | 0 | 0 | 0 | 0 | 0 | 3 |
| kb742808.1_199_mature | kb742808.1_199_mature | ttttttggtgactgatg | 27 | 27 | 43 | 53 | 37 | 15 | 7 | 9 |
| kb744033.1_9307_star | kb744033.1_9307_star | ctactacgtatttgaaagcaaat | 0 | 0 | 1 | 0 | 0 | 1 | 1 | 0 |
| kb742967.1_10091_star | kb742967.1_10091_star | tgctcgaacagtattctgc | 1 | 1 | 1 | 0 | 0 | 0 | 0 | 0 |
| kb743832.1_17963_mature | kb743832.1_17963_mature | catttagccaatttgaag | 0 | 0 | 4 | 0 | 0 | 0 | 0 | 0 |
| kb746316.1_19650_star | kb746316.1_19650_star | ggctgctgtcacagacttaga | 1 | 2 | 3 | 2 | 2 | 0 | 3 | 1 |
| kb742479.1_270_star | kb742479.1_270_star | tcaatagacaccaaaagg | 0 | 0 | 0 | 0 | 0 | 0 | 3 | 0 |
| kb744187.1_24329_mature | kb744187.1_24329_mature | cagggaggggaagggaggc | 6 | 2 | 10 | 3 | 3 | 4 | 4 | 5 |
| kb743943.1_22946_star | kb743943.1_22946_star | gaggggagagtggtggatgttgtcta | 0 | 3 | 1 | 1 | 2 | 0 | 0 | 3 |
| kb743402.1_5886_star | kb743402.1_5886_star | gttcaagtctcagcagaac | 2 | 9 | 1 | 3 | 2 | 2 | 3 | 2 |
| kb742757.1_11059_star | kb742757.1_11059_star | cagcccaggtctctggaggaa | 0 | 1 | 5 | 1 | 0 | 0 | 2 | 1 |
| kb744435.1_16058_mature | kb744435.1_16058_mature | tttggatggaagactgctcat | 2 | 5 | 6 | 0 | 3 | 0 | 5 | 3 |
| kb742618.1_11457_star | kb742618.1_11457_star | ggatgctcaagagggatggg | 0 | 0 | 0 | 1 | 1 | 0 | 0 | 0 |
| kb743037.1_11817_mature | kb743037.1_11817_mature | ctgatcttgttgtaatctgct | 1 | 5 | 2 | 0 | 1 | 0 | 1 | 3 |
| kb742527.1_3882_mature | kb742527.1_3882_mature | gatgtggatgtgtctct | 14 | 15 | 70 | 25 | 14 | 17 | 50 | 7 |
| kb743097.1_10493_mature | kb743097.1_10493_mature | aggacaacaaggacaaca | 1 | 1 | 5 | 2 | 3 | 0 | 1 | 0 |
| kb742471.1_425_star | kb742471.1_425_star | tgtgtttcccagtcattctct | 0 | 3 | 2 | 0 | 1 | 0 | 2 | 3 |
| kb743091.1_21604_mature | kb743091.1_21604_mature | tccggcctggagacgaccttatc | 4 | 6 | 4 | 6 | 5 | 8 | 7 | 4 |
| kb742757.1_11033_mature | kb742757.1_11033_mature | ctggactgcccaggcaatgacga | 1 | 2 | 5 | 1 | 2 | 2 | 1 | 1 |
| kb742496.1_9558_star | kb742496.1_9558_star | ctccaagatcaccaagtccaacctct | 9 | 64 | 5 | 55 | 13 | 2 | 0 | 3 |
| kb742432.1_505_mature | kb742432.1_505_mature | ccagagagtttgtagagtc | 4 | 24 | 0 | 25 | 2 | 0 | 1 | 0 |

| kb748326.1_22635_mature | | kb748326.1_22635_mature | | atccggcttctgtagtgacttgc | 0 | | | 1 | | 4 | | 4 | | 3 | | 1 | | 6 | | 4 |
| --- | --- | --- | --- | --- | --- | --- | --- | --- | --- | --- | --- | --- | --- | --- | --- | --- | --- | --- | --- | --- |
| kb745003.1_21568_mature | | kb745003.1_21568_mature | | tttagagatgactgttt | 0 | | | 0 | | 1 | | 0 | | 0 | | 0 | | 1 | | 0 |
| kb742438.1_21842_star | | kb742438.1_21842_star | | tgtgaggtcccgggttca | 3 | | | 3 | | 11 | | 0 | | 1 | | 0 | | 0 | | 8 |
| kb742660.1_11549_star | | kb742660.1_11549_star | | agaagcataaggcatgggaaaa | 33 | | | 101 | | 42 | | 88 | | 24 | | 5 | | 13 | | 8 |
| kb742777.1_8208_star | | kb742777.1_8208_star | | cagcagttaagcagcgg | 0 | | | 0 | | 0 | | 1 | | 0 | | 0 | | 0 | | 0 |
| kb742522.1_6455_star | | kb742522.1_6455_star | | acttgttagccctttcagatggaattgc | 0 | | | 3 | | 0 | | 2 | | 0 | | 0 | | 1 | | 0 |
| kb742772.1_19002_star | | kb742772.1_19002_star | | agtgtcaaaagccttactgaa | 4 | | | 1 | | 3 | | 8 | | 0 | | 2 | | 0 | | 3 |
| kb743181.1_19161_star | | kb743181.1_19161_star | | cgtcgagccccacgtgcaagac | 1 | | | 4 | | 0 | | 3 | | 2 | | 0 | | 2 | | 0 |
| kb743037.1_11806_mature | | kb743037.1_11806_mature | | ttggacctgatgatcctgaag | 2 | | | 30 | | 3 | | 35 | | 6 | | 3 | | 7 | | 2 |
| kb742629.1_585_star | | kb742629.1_585_star | | tgcacagctgagcttgctggaa | 0 | | | 0 | | 3 | | 0 | | 3 | | 0 | | 3 | | 0 |
| kb743891.1_15790_mature | | kb743891.1_15790_mature | | aagagacattctctgtct | 0 | | | 0 | | 0 | | 0 | | 0 | | 0 | | 3 | | 0 |
| kb742854.1_7287_mature | | kb742854.1_7287_mature | | ttttctgcaggactctg | 0 | | | 5 | | 0 | | 17 | | 2 | | 0 | | 0 | | 6 |
| kb744904.1_21478_star | | kb744904.1_21478_star | | cagacagtttatgatcctgaaa | 1 | | | 4 | | 0 | | 2 | | 1 | | 1 | | 1 | | 0 |
| kb743235.1_6581_mature | | kb743235.1_6581_mature | | gtggcaatgctgtagact | 0 | | | 4 | | 0 | | 1 | | 0 | | 2 | | 0 | | 0 |
| kb744904.1_21478_mature | | kb744904.1_21478_mature | | ttaggaacaaagactgtcctggt | 7 | | | 60 | | 2 | | 95 | | 8 | | 3 | | 6 | | 2 |
| kb742617.1_6191_star | | kb742617.1_6191_star | | tatatgtcttttttgtcc | 1 | | | 2 | | 1 | | 0 | | 0 | | 0 | | 1 | | 0 |
| kb742421.1_16835_star | | kb742421.1_16835_star | | cagagcaggggcagatcctgc | 0 | | | 3 | | 6 | | 1 | | 3 | | 1 | | 4 | | 1 |
| kb742444.1_7990_mature | | kb742444.1_7990_mature | | cacctgtgagctgacatgcgaat | 0 | | | 2 | | 7 | | 0 | | 0 | | 1 | | 1 | | 1 |
| kb743686.1_3417_star | | kb743686.1_3417_star | | ggtaagtgtctggttgtggg | 1 | | | 0 | | 0 | | 1 | | 0 | | 0 | | 0 | | 0 |
| kb742690.1_5389_mature | | kb742690.1_5389_mature | | gatttctggcctgttggaagttgca | 1 | | | 6 | | 7 | | 2 | | 2 | | 1 | | 0 | | 0 |
| kb742650.1_8458_mature | | kb742650.1_8458_mature | | actgggatgtaggaatgcc | 2 | | | 1 | | 6 | | 0 | | 0 | | 0 | | 0 | | 0 |
| kb743005.1_3031_mature | | kb743005.1_3031_mature | | aggatttgtaagactgttgca | 4 | | | 3 | | 1 | | 7 | | 0 | | 10 | | 23 | | 2 |
| kb742585.1_9389_star | | kb742585.1_9389_star | | gtggactgcagggacgacc | 0 | | | 2 | | 0 | | 0 | | 1 | | 1 | | 1 | | 0 |
| kb742651.1_5217_star | | kb742651.1_5217_star | | ggggagctgtttcaac | 1 | | | 0 | | 0 | | 0 | | 0 | | 0 | | 1 | | 0 |
| kb744955.1_13796_mature | | kb744955.1_13796_mature | | ttttcagtgtgctctctgtgt | 6 | | | 22 | | 31 | | 15 | | 2 | | 2 | | 17 | | 7 |
| kb742736.1_12008_mature | | kb742736.1_12008_mature | | ttggccatgatgtggtggagcc | 2 | | | 11 | | 3 | | 3 | | 0 | | 0 | | 3 | | 1 |
| kb743944.1_1453_mature | | kb743944.1_1453_mature | | tgaagcagatgatgagctcttcca | 7 | | | 23 | | 11 | | 6 | | 3 | | 2 | | 19 | | 5 |
| kb742808.1_108_star | | kb742808.1_108_star | | gaaggaatggtgtgatgttttgc | 0 | | | 0 | | 2 | | 1 | | 1 | | 1 | | 2 | | 0 |
| kb743180.1_19957_mature | | kb743180.1_19957_mature | | ctcgtcgaggtcttctcc | 0 | | | 3 | | 0 | | 0 | | 1 | | 0 | | 0 | | 1 |
| kb744927.1_18246_mature | | kb744927.1_18246_mature | | ttcccatcgtgcagctgtgtcgg | 1 | | | 0 | | 2 | | 4 | | 1 | | 0 | | 0 | | 0 |
| kb743475.1_18384_mature | | kb743475.1_18384_mature | | cttgtcttagtgcctgtctccagac | 3 | | | 1 | | 0 | | 0 | | 2 | | 1 | | 3 | | 0 |
| kb742808.1_199_star | | kb742808.1_199_star | | tgagtcatgaaatcatt | 0 | | | 0 | | 0 | | 2 | | 0 | | 0 | | 0 | | 0 |
| kb743437.1_21710_star | | kb743437.1_21710_star | | ggccccaattcagtttcttttgag | 0 | | | 2 | | 2 | | 1 | | 0 | | 1 | | 4 | | 0 |
| kb742651.1_5201_star | | kb742651.1_5201_star | | tggacagccacagtgctttct | 2 | | | 6 | | 1 | | 0 | | 0 | | 0 | | 5 | | 3 |
| kb743194.1_20366_star | | kb743194.1_20366_star | | tccaacttcatagttttcctgagg | 0 | | | 1 | | 3 | | 2 | | 3 | | 0 | | 0 | | 0 |
| kb744466.1_17606_mature | | kb744466.1_17606_mature | | tcaagactgctctccgctttgggt | 9 | | | 78 | | 1 | | 108 | | 35 | | 3 | | 19 | | 10 |
| kb742646.1_6778_star | | kb742646.1_6778_star | | taaaagactccaaggtcac | 0 | | | 0 | | 1 | | 0 | | 0 | | 0 | | 0 | | 0 |
| kb742745.1_15184_star | | kb742745.1_15184_star | | aggtgctccagtcccttggtca | 1 | | | 6 | | 8 | | 2 | | 2 | | 2 | | 1 | | 0 |
| kb744722.1_10780_star | | kb744722.1_10780_star | | aactacgtaacctttacagcagta | 0 | | | 0 | | 1 | | 1 | | 0 | | 0 | | 0 | | 0 |
| kb742819.1_21534_star | | kb742819.1_21534_star | | ctcattatcagatctgca | 1 | | | 0 | | 0 | | 0 | | 0 | | 1 | | 0 | | 0 |
| kb742446.1_12980_star | | kb742446.1_12980_star | | ttcagtatttccttttgtagtga | 0 | | | 6 | | 1 | | 3 | | 0 | | 0 | | 1 | | 0 |
| kb742832.1_11524_mature | | kb742832.1_11524_mature | | tcccttctctgcctcacgcag | 0 | | | 2 | | 0 | | 5 | | 3 | | 0 | | 0 | | 0 |
| kb743105.1_19472_mature | | kb743105.1_19472_mature | | aatggatgagatggctggacc | 1 | | | 5 | | 7 | | 3 | | 0 | | 1 | | 2 | | 1 |
| kb743062.1_19334_mature | | kb743062.1_19334_mature | | ttggagactggctctatgcc | 0 | | | 0 | | 0 | | 4 | | 0 | | 1 | | 2 | | 0 |
| kb742665.1_22443_mature | | kb742665.1_22443_mature | | cctggctgaagcaggctct | 0 | | | 0 | | 6 | | 0 | | 1 | | 1 | | 2 | | 0 |
| kb743403.1_19670_mature | | kb743403.1_19670_mature | | catgtctaagtacgcacggg | 0 | | | 0 | | 2 | | 1 | | 0 | | 0 | | 0 | | 1 |
| kb742478.1_20560_mature | | kb742478.1_20560_mature | | ttccaaactcagacatattg | 8 | | | 24 | | 11 | | 16 | | 2 | | 4 | | 15 | | 5 |
| kb746071.1_19970_mature | | kb746071.1_19970_mature | | tttcagtctgtgctcactgcc | 5 | | | 7 | | 2 | | 3 | | 2 | | 1 | | 9 | | 0 |
| kb743366.1_17995_star | | kb743366.1_17995_star | | agcctgtaaactaccgcaactcttcctt | 0 | | | 0 | | 0 | | 1 | | 0 | | 1 | | 0 | | 0 |
| kb742384.1_20257_mature | | kb742384.1_20257_mature | | gtgaagacttgatgaaca | 2 | | | 7 | | 5 | | 15 | | 2 | | 3 | | 20 | | 1 |
| kb743192.1_11605_star | | kb743192.1_11605_star | | gaatgaggccacagaaggagca | 1 | | | 0 | | 2 | | 1 | | 1 | | 0 | | 0 | | 1 |
| kb743564.1_13642_mature | | kb743564.1_13642_mature | | tttggaactagtagatgtgctctct | 23 | | | 383 | | 4 | | 122 | | 7 | | 3 | | 12 | | 7 |
| kb742675.1_10535_mature | | kb742675.1_10535_mature | | gtgaaactggagtactcc | 1 | | | 4 | | 5 | | 0 | | 2 | | 4 | | 4 | | 2 |
| kb742479.1_277_star | | kb742479.1_277_star | | ctcgtttgtcagcttagttgg | 0 | | | 0 | | 1 | | 0 | | 0 | | 1 | | 0 | | 1 |
| kb742750.1_2171_star | | kb742750.1_2171_star | | cgatgaagctggtgaggggtc | 5 | | | 13 | | 5 | | 23 | | 3 | | 0 | | 1 | | 0 |
| kb742456.1_13538_mature | | kb742456.1_13538_mature | | atttccagtcatttctcta | 0 | | | 0 | | 5 | | 4 | | 0 | | 1 | | 3 | | 0 |
| kb742734.1_7054_mature | | kb742734.1_7054_mature | | atgtgtgactgcatcatgtgct | 1 | | | 10 | | 2 | | 3 | | 2 | | 0 | | 2 | | 0 |
| kb742515.1_4126_star | | kb742515.1_4126_star | | tggaagtacaatcacagctcaggg | 2 | | | 6 | | 0 | | 0 | | 0 | | 2 | | 6 | | 1 |
| kb745003.1_21574_star | | kb745003.1_21574_star | | ctgccgtcctcacc | 0 | | | 0 | | 0 | | 1 | | 0 | | 0 | | 0 | | 0 |
| kb742808.1_211_mature | | kb742808.1_211_mature | | tggaatttgtctttggca | 0 | | | 0 | | 0 | | 0 | | 0 | | 0 | | 6 | | 0 |
| kb742876.1_14062_mature | | kb742876.1_14062_mature | | acgcagactccgatcactt | 24 | | | 16 | | 12 | | 39 | | 7 | | 2 | | 171 | | 13 |
| kb742696.1_10444_mature | | kb742696.1_10444_mature | | aaagtaggtgcagtagatgcaga | 0 | | | 0 | | 4 | | 4 | | 1 | | 0 | | 0 | | 0 |
| kb742411.1_7602_star | | kb742411.1_7602_star | | ttgtgccagatcatttggtga | 0 | | | 0 | | 1 | | 3 | | 1 | | 0 | | 0 | | 1 |
| kb742967.1_10091_mature | | kb742967.1_10091_mature | | agctagctgcgagaatt | 5 | | | 5 | | 2 | | 4 | | 3 | | 4 | | 5 | | 5 |
| kb743241.1_16749_mature | | kb743241.1_16749_mature | | tggagttgttcctctggc | 2 | | | 4 | | 1 | | 1 | | 3 | | 0 | | 1 | | 0 |
| kb742807.1_16447_star | | kb742807.1_16447_star | | cacgcgtctgcagtgctcctcagt | 0 | | | 4 | | 2 | | 1 | | 1 | | 0 | | 8 | | 1 |
| kb743651.1_7914_mature | | kb743651.1_7914_mature | | ggtgaactgcagtctctgc | 7 | | | 3 | | 5 | | 2 | | 3 | | 2 | | 3 | | 1 |
| kb743120.1_18164_star | | kb743120.1_18164_star | | taggcagtgtgttgttcggtggctgct | 1 | | | 2 | | 1 | | 2 | | 0 | | 2 | | 3 | | 2 |
| kb742543.1_11170_mature | | kb742543.1_11170_mature | | cttggaagcaattcttctattc | 1 | | | 4 | | 0 | | 0 | | 0 | | 0 | | 2 | | 0 |
| kb743145.1_821_star | | kb743145.1_821_star | | tcaaatactaattcagattat | 1 | | | 0 | | 0 | | 0 | | 0 | | 0 | | 0 | | 1 |
| kb743435.1_2991_mature | | kb743435.1_2991_mature | | atctgttgagtgctaaatgac | 1 | | | 1 | | 0 | | 0 | | 0 | | 0 | | 3 | | 0 |
| kb743547.1_18203_star | | kb743547.1_18203_star | | caccgctgccctgagtgtgcc | 0 | | | 0 | | 1 | | 0 | | 0 | | 0 | | 1 | | 0 |
| kb743787.1_22728_mature | | kb743787.1_22728_mature | | tgtgagccgatggaccg | 6 | | | 40 | | 0 | | 192 | | 13 | | 2 | | 1 | | 0 |
| kb743381.1_20048_mature | | kb743381.1_20048_mature | | tcccgagctttgaatagt | 0 | | | 5 | | 0 | | 0 | | 1 | | 0 | | 0 | | 0 |
| kb742728.1_18177_mature | | kb742728.1_18177_mature | | ttttggcttaggatgagaagagt | 16 | | | 158 | | 4 | | 179 | | 72 | | 4 | | 12 | | 16 |
| kb742489.1_7317_star | | kb742489.1_7317_star | | atggaattcagcttatctgcaaatc | 0 | | | 0 | | 3 | | 0 | | 1 | | 1 | | 0 | | 1 |
| kb743915.1_20297_mature | | kb743915.1_20297_mature | | ctggagcagttgcaggagg | 0 | | | 11 | | 0 | | 13 | | 3 | | 0 | | 4 | | 2 |
| kb744553.1_17004_star | | kb744553.1_17004_star | | attctcactcgtttgggtttt | 0 | | | 3 | | 0 | | 3 | | 0 | | 0 | | 2 | | 0 |
| kb742613.1_16261_mature | | kb742613.1_16261_mature | | aagtgcactgccagctgatagcc | 1 | | | 1 | | 4 | | 4 | | 1 | | 1 | | 1 | | 0 |
| kb742489.1_7302_mature | | kb742489.1_7302_mature | | atgactgtgttctttgaatgagcc | 1 | | | 12 | | 4 | | 10 | | 1 | | 2 | | 14 | | 2 |
| kb744800.1_22494_star | | kb744800.1_22494_star | | ccagagccacaacagcaaggccg | 0 | | | 2 | | 3 | | 3 | | 0 | | 0 | | 1 | | 2 |
| kb742845.1_5842_mature | | kb742845.1_5842_mature | | gacgtgctgttgctcttctagg | 8 | | | 11 | | 8 | | 3 | | 3 | | 3 | | 7 | | 1 |
| kb742716.1_17730_star | | kb742716.1_17730_star | | tcgatgggaacgctcacggaa | 2 | | | 2 | | 1 | | 1 | | 2 | | 0 | | 1 | | 0 |
| kb742499.1_8255_mature | | kb742499.1_8255_mature | | tttgcagatctctctgaa | 0 | | | 3 | | 0 | | 1 | | 0 | | 0 | | 7 | | 0 |
| kb744188.1_22206_mature | | kb744188.1_22206_mature | | aaggacattgccagaaagt | 2 | | | 10 | | 2 | | 9 | | 6 | | 2 | | 0 | | 0 |
| kb743228.1_14904_star | | kb743228.1_14904_star | | aattactcacacaacttc | 1 | | | 0 | | 2 | | 0 | | 1 | | 0 | | 0 | | 0 |
| kb744328.1_20788_star | | kb744328.1_20788_star | | ctggctcatcctgcctttct | 0 | | | 0 | | 1 | | 0 | | 1 | | 0 | | 1 | | 0 |
| kb743146.1_13252_mature | | kb743146.1_13252_mature | | tttctctgggctgtgtt | 1 | | | 48 | | 0 | | 31 | | 9 | | 0 | | 1 | | 2 |
| kb743335.1_9717_star | | kb743335.1_9717_star | | ctccactgtgtgacatcttttgttg | 2 | | | 1 | | 3 | | 2 | | 3 | | 0 | | 3 | | 1 |
| kb742722.1_13453_mature | | kb742722.1_13453_mature | | cccctttctgctgacggagt | 0 | | | 2 | | 0 | | 2 | | 0 | | 0 | | 1 | | 3 |
| kb742685.1_1622_mature | | kb742685.1_1622_mature | | ttcagacaggctcggtg | 33 | | | 184 | | 0 | | 298 | | 63 | | 8 | | 12 | | 10 |
| kb745863.1_24310_mature | | kb745863.1_24310_mature | | accgccgccagggctccgacacc | 1 | | | 4 | | 4 | | 0 | | 0 | | 1 | | 3 | | 2 |
| kb742887.1_1499_mature | | kb742887.1_1499_mature | | tgattgtattctgtgta | 0 | | | 1 | | 0 | | 0 | | 0 | | 1 | | 1 | | 0 |
| kb742931.1_975_star | | kb742931.1_975_star | | ctgagcagctcctctcccattg | 4 | | | 7 | | 8 | | 11 | | 5 | | 3 | | 3 | | 3 |
| kb743589.1_20877_mature | | kb743589.1_20877_mature | | gtggctgtaggagatggc | 1 | | | 0 | | 1 | | 0 | | 2 | | 1 | | 2 | | 1 |
| kb742448.1_5978_star | | kb742448.1_5978_star | | tttcagatgttacggtgcag | 1 | | | 1 | | 0 | | 0 | | 0 | | 0 | | 0 | | 0 |
| kb742617.1_6182_mature | | kb742617.1_6182_mature | | acgttggacagaaattcc | 1 | | | 4 | | 0 | | 0 | | 0 | | 0 | | 0 | | 0 |
| kb743403.1_19681_mature | | kb743403.1_19681_mature | | ctgatgatgaggatgaagat | 1 | | | 2 | | 9 | | 5 | | 3 | | 1 | | 7 | | 1 |
| kb746071.1_19965_mature | | kb746071.1_19965_mature | | tcagctgctcttctcctgccaga | 1 | | | 6 | | 3 | | 2 | | 1 | | 4 | | 2 | | 1 |
| kb743005.1_3003_star | | kb743005.1_3003_star | | tgattcctcatttgatg | 0 | | | 0 | | 1 | | 2 | | 1 | | 1 | | 1 | | 1 |
| kb742873.1_1641_mature | | kb742873.1_1641_mature | | cgagatccgagacggggac | 0 | | | 1 | | 0 | | 3 | | 0 | | 0 | | 0 | | 0 |
| kb744466.1_17599_star | | kb744466.1_17599_star | | gcctgcagccaagcaccc | 0 | | | 1 | | 2 | | 0 | | 3 | | 0 | | 0 | | 1 |
| kb742531.1_10727_star | | kb742531.1_10727_star | | ggtgtatcagcgcctgtgct | 1 | | | 0 | | 0 | | 0 | | 1 | | 0 | | 1 | | 0 |
| kb743046.1_19354_star | | kb743046.1_19354_star | | caggagccgtaatcggaaatggat | 0 | | | 0 | | 0 | | 0 | | 0 | | 0 | | 1 | | 0 |
| kb742851.1_13582_star | | kb742851.1_13582_star | | cgctacccggacagcagggtca | 0 | | | 0 | | 2 | | 1 | | 2 | | 0 | | 0 | | 0 |
| kb742499.1_8266_mature | | kb742499.1_8266_mature | | attcctaggatctacggaggggctt | 0 | | | 0 | | 0 | | 3 | | 0 | | 0 | | 0 | | 0 |
| kb743609.1_4586_mature | | kb743609.1_4586_mature | | gaggattcagagcaatgca | 0 | | | 1 | | 1 | | 0 | | 0 | | 3 | | 4 | | 0 |
| kb742745.1_15171_mature | | kb742745.1_15171_mature | | gtgtgtggatgaaacatcta | 0 | | | 2 | | 1 | | 0 | | 1 | | 1 | | 6 | | 3 |
| kb818614.1_24936_star | | kb818614.1_24936_star | | agtttgtttccaagaaggctgatga | 0 | | | 5 | | 3 | | 1 | | 2 | | 0 | | 2 | | 1 |
| kb742773.1_1207_mature | | kb742773.1_1207_mature | | ctctgcgaggacaactggaca | 0 | | | 1 | | 3 | | 1 | | 0 | | 3 | | 2 | | 1 |
| kb743192.1_11605_mature | | kb743192.1_11605_mature | | ctccttctgtggcctcattcac | 9 | | | 0 | | 2 | | 1 | | 2 | | 0 | | 9 | | 0 |
| kb743649.1_15515_star | | kb743649.1_15515_star | | tttgtatccggaaatccattg | 1 | | | 1 | | 0 | | 0 | | 0 | | 0 | | 0 | | 0 |
| kb744955.1_13807_mature | | kb744955.1_13807_mature | | aaaagtgtgcaatagatatctct | 0 | | | 2 | | 1 | | 0 | | 0 | | 1 | | 4 | | 0 |
| kb742466.1_4810_star | | kb742466.1_4810_star | | ttcctcactagtgcaagctatggggga | 0 | | | 1 | | 0 | | 0 | | 0 | | 3 | | 0 | | 1 |
| kb743569.1_19235_mature | | kb743569.1_19235_mature | | ttttggggctctgtgacc | 0 | | | 0 | | 4 | | 0 | | 0 | | 1 | | 2 | | 1 |
| kb743319.1_12031_star | | kb743319.1_12031_star | | gatagagtgaggaggaagaagaatcag | 4 | | | 75 | | 6 | | 23 | | 15 | | 2 | | 3 | | 10 |
| kb742866.1_14616_mature | | kb742866.1_14616_mature | | aatgggacacacgtgctggagt | 1 | | | 7 | | 1 | | 3 | | 1 | | 2 | | 1 | | 1 |
| kb743600.1_21293_star | | kb743600.1_21293_star | | aatgctgcacaggtgtcacggg | 0 | | | 0 | | 1 | | 1 | | 0 | | 1 | | 0 | | 1 |
| kb742556.1_16191_mature | | kb742556.1_16191_mature | | tgccttctctgtatcttct | 0 | | | 11 | | 0 | | 0 | | 1 | | 1 | | 1 | | 0 |
| kb742966.1_4458_mature | | kb742966.1_4458_mature | | cacatggcagctgcaggta | 0 | | | 0 | | 3 | | 0 | | 0 | | 0 | | 0 | | 0 |
| kb743893.1_20154_star | | kb743893.1_20154_star | | tggtcattcaaggtgggtaaa | 1 | | | 0 | | 0 | | 0 | | 0 | | 0 | | 0 | | 0 |
| kb743807.1_23035_star | | kb743807.1_23035_star | | ccagatcattggtaaaggttttgaagag | 2 | | | 1 | | 2 | | 11 | | 2 | | 0 | | 1 | | 0 |
| kb743158.1_2885_mature | | kb743158.1_2885_mature | | atgtcgtttcttcctttcagt | 0 | | | 2 | | 3 | | 0 | | 0 | | 0 | | 4 | | 0 |
| kb743028.1_7251_mature | | kb743028.1_7251_mature | | gtgctgtggtgaaacttc | 426 | | | 1213 | | 489 | | 639 | | 424 | | 135 | | 222 | | 16 |
| kb744360.1_23719_star | | kb744360.1_23719_star | | tgcaggagggcaggaaatggccac | 1 | | | 0 | | 1 | | 1 | | 0 | | 1 | | 2 | | 0 |
| kb743110.1_6007_mature | | kb743110.1_6007_mature | | atattgtggatcttcaga | 0 | | | 3 | | 1 | | 2 | | 2 | | 0 | | 5 | | 0 |
| kb743025.1_20344_star | | kb743025.1_20344_star | | ttaactccactggaat | 0 | | | 2 | | 0 | | 0 | | 0 | | 0 | | 0 | | 0 |
| kb742712.1_8624_star | | kb742712.1_8624_star | | cgtgacatctccatggctgta | 1 | | | 0 | | 3 | | 0 | | 0 | | 0 | | 0 | | 1 |
| kb743493.1_18284_star | | kb743493.1_18284_star | | agcagagcttgtgaaatcccc | 0 | | | 3 | | 5 | | 0 | | 0 | | 3 | | 2 | | 1 |
| kb744389.1_22415_star | | kb744389.1_22415_star | | caactaccttagcaccttt | 0 | | | 1 | | 2 | | 0 | | 1 | | 1 | | 0 | | 1 |
| kb742586.1_16543_star | | kb742586.1_16543_star | | ctgtcaagttttgaagtggtgag | 0 | | | 1 | | 1 | | 4 | | 0 | | 2 | | 0 | | 0 |
| kb742471.1_387_mature | | kb742471.1_387_mature | | actggttgggagctgttactggt | 2 | | | 1 | | 8 | | 2 | | 4 | | 1 | | 2 | | 2 |
| kb742974.1_23116_mature | | kb742974.1_23116_mature | | tatggaactagtagacgcgctctct | 1 | | | 20 | | 5 | | 6 | | 2 | | 0 | | 0 | | 0 |
| kb743048.1_8301_mature | | kb743048.1_8301_mature | | ttggccttgttgaacctc | 126 | | | 185 | | 0 | | 195 | | 20 | | 6 | | 4 | | 6 |
| kb744772.1_17697_star | | kb744772.1_17697_star | | agcatggagagttgcagcagg | 0 | | | 0 | | 0 | | 0 | | 1 | | 0 | | 2 | | 1 |
| kb743028.1_7251_star | | kb743028.1_7251_star | | agtcagtcacccagagata | 2 | | | 3 | | 0 | | 1 | | 1 | | 1 | | 0 | | 0 |
| kb752032.1_25714_star | | kb752032.1_25714_star | | tggtgtctgacctggag | 0 | | | 0 | | 0 | | 1 | | 0 | | 2 | | 1 | | 0 |
| kb743487.1_17951_star | | kb743487.1_17951_star | | tgccttctctctgcatgc | 0 | | | 0 | | 0 | | 0 | | 0 | | 1 | | 1 | | 0 |
| kb742574.1_16076_star | | kb742574.1_16076_star | | tcagctgcaagccaaaaatctgct | 2 | | | 0 | | 0 | | 2 | | 1 | | 0 | | 0 | | 1 |
| kb742651.1_5179_star | | kb742651.1_5179_star | | tggacgtgtatgtgatgtaggaca | 5 | | | 31 | | 20 | | 9 | | 5 | | 2 | | 9 | | 8 |
| kb743471.1_15457_mature | | kb743471.1_15457_mature | | cccgcgctgcttttactgccg | 0 | | | 0 | | 2 | | 0 | | 3 | | 0 | | 0 | | 0 |
| kb743057.1_15968_star | | kb743057.1_15968_star | | ggtcacgcaggagtattc | 0 | | | 0 | | 0 | | 0 | | 0 | | 0 | | 1 | | 0 |
| kb744942.1_17914_mature | | kb744942.1_17914_mature | | gtggatttgttaatacttt | 0 | | | 0 | | 4 | | 0 | | 0 | | 0 | | 0 | | 0 |
| kb742418.1_2465_mature | | kb742418.1_2465_mature | | tctgctatgacttggagc | 0 | | | 1 | | 0 | | 1 | | 0 | | 0 | | 2 | | 0 |
| kb742875.1_10959_star | | kb742875.1_10959_star | | aggggagcaaaagggaggcaaat | 0 | | | 0 | | 0 | | 0 | | 0 | | 0 | | 1 | | 0 |
| kb744307.1_22722_star | | kb744307.1_22722_star | | tgtgcagggagatcctcactccgc | 0 | | | 1 | | 1 | | 3 | | 1 | | 0 | | 1 | | 0 |
| kb743110.1_6089_mature | | kb743110.1_6089_mature | | ctgtctcctcgctcgtgggg | 23 | | | 33 | | 24 | | 17 | | 16 | | 22 | | 29 | | 24 |
| kb743459.1_15677_star | | kb743459.1_15677_star | | atgcatacagccagctccttg | 0 | | | 1 | | 1 | | 1 | | 1 | | 1 | | 0 | | 0 |
| kb744265.1_23560_star | | kb744265.1_23560_star | | tcttcattggtgcagcctcacctca | 8 | | | 92 | | 23 | | 41 | | 18 | | 13 | | 31 | | 16 |
| kb743356.1_12126_mature | | kb743356.1_12126_mature | | gccgatggtgtggaaggtg | 0 | | | 1 | | 1 | | 2 | | 4 | | 0 | | 3 | | 0 |
| kb743110.1_6005_star | | kb743110.1_6005_star | | tcctaccagagatctatcatca | 4 | | | 1 | | 0 | | 0 | | 0 | | 0 | | 3 | | 0 |
| kb742477.1_14355_star | | kb742477.1_14355_star | | agtaagacatttcacatt | 1 | | | 0 | | 0 | | 2 | | 0 | | 1 | | 0 | | 0 |
| kb742395.1_8096_star | | kb742395.1_8096_star | | agttgggatacatgcagcacaaa | 0 | | | 1 | | 0 | | 1 | | 0 | | 2 | | 0 | | 2 |
| kb742992.1_5523_star | | kb742992.1_5523_star | | ctctgtcatctttgaaaggtcct | 0 | | | 2 | | 0 | | 2 | | 0 | | 0 | | 2 | | 1 |
| kb742393.1_17361_star | | kb742393.1_17361_star | | gcggcaagatcattctggagtgca | 0 | | | 1 | | 0 | | 0 | | 0 | | 0 | | 0 | | 0 |
| kb743399.1_14091_star | | kb743399.1_14091_star | | ttcctaaagaccttcactgct | 1 | | | 0 | | 2 | | 1 | | 0 | | 0 | | 0 | | 0 |
| kb742652.1_6562_mature | | kb742652.1_6562_mature | | gtgcctgaagactggagaa | 0 | | | 4 | | 1 | | 2 | | 1 | | 0 | | 3 | | 0 |
| kb742840.1_6712_mature | | kb742840.1_6712_mature | | aactgtttttaacttcttcatttcc | 1 | | | 3 | | 5 | | 1 | | 3 | | 0 | | 1 | | 0 |
| kb743459.1_15670_star | | kb743459.1_15670_star | | tcttcatcaggtaggagctcttgtct | 0 | | | 2 | | 4 | | 1 | | 0 | | 2 | | 1 | | 1 |
| kb742451.1_6367_star | | kb742451.1_6367_star | | tggttagaggacagttgttaat | 0 | | | 1 | | 0 | | 0 | | 2 | | 0 | | 1 | | 2 |
| kb742418.1_2476_mature | | kb742418.1_2476_mature | | gtaaggagtgtggagattatggt | 1 | | | 4 | | 7 | | 4 | | 0 | | 0 | | 1 | | 0 |
| kb743722.1_14627_mature | | kb743722.1_14627_mature | | tgctgtaatgtgattgatac | 0 | | | 6 | | 5 | | 7 | | 1 | | 1 | | 4 | | 0 |
| kb743421.1_18721_mature | | kb743421.1_18721_mature | | ctaatggataaggcactg | 21 | | | 127 | | 101 | | 110 | | 83 | | 116 | | 312 | | 171 |
| kb803520.1_25507_star | | kb803520.1_25507_star | | tgcactcagcggcgttgg | 0 | | | 0 | | 0 | | 0 | | 1 | | 1 | | 0 | | 0 |
| kb744033.1_9316_star | | kb744033.1_9316_star | | ttccgatgacagacagtataa | 1 | | | 12 | | 1 | | 16 | | 3 | | 1 | | 1 | | 1 |
| kb742467.1_8783_mature | | kb742467.1_8783_mature | | ttgagagtcatcttgcggcacttg | 0 | | | 6 | | 0 | | 30 | | 5 | | 2 | | 0 | | 2 |
| kb742664.1_9858_mature | | kb742664.1_9858_mature | | ctgaagattttgaataaa | 0 | | | 11 | | 1 | | 9 | | 3 | | 2 | | 6 | | 2 |
| kb742896.1_17390_mature | | kb742896.1_17390_mature | | ctttgattgaacgtgagagc | 0 | | | 2 | | 0 | | 0 | | 0 | | 4 | | 0 | | 0 |
| kb743642.1_19561_mature | | kb743642.1_19561_mature | | tgtgaatttactggggat | 0 | | | 0 | | 0 | | 3 | | 0 | | 0 | | 0 | | 0 |
| kb742864.1_10344_star | | kb742864.1_10344_star | | aacaaccacttccctagtcctgc | 0 | | | 2 | | 1 | | 4 | | 2 | | 0 | | 0 | | 1 |
| kb742473.1_6391_mature | | kb742473.1_6391_mature | | tggctgcttagcatttttgagaa | 0 | | | 4 | | 0 | | 0 | | 2 | | 0 | | 0 | | 0 |
| kb742553.1_13404_star | | kb742553.1_13404_star | | ctaagggggctgtatttc | 0 | | | 3 | | 0 | | 0 | | 1 | | 0 | | 0 | | 0 |
| kb744509.1_22052_mature | | kb744509.1_22052_mature | | tctctacggtgctggagagc | 1 | | | 6 | | 4 | | 3 | | 3 | | 1 | | 1 | | 1 |
| kb742934.1_13234_star | | kb742934.1_13234_star | | tggtatactttgatggaagtgat | 0 | | | 3 | | 3 | | 0 | | 0 | | 0 | | 0 | | 0 |
| kb742438.1_21848_mature | | kb742438.1_21848_mature | | aggggcagggcagggctg | 0 | | | 0 | | 4 | | 0 | | 1 | | 0 | | 0 | | 1 |
| kb743463.1_20524_star | | kb743463.1_20524_star | | ttcaagcatccttcctcaaaacc | 0 | | | 1 | | 2 | | 0 | | 2 | | 0 | | 1 | | 0 |
| kb742811.1_13102_mature | | kb742811.1_13102_mature | | cccctgaccgaccccactgaga | 6 | | | 18 | | 12 | | 21 | | 10 | | 5 | | 7 | | 3 |
| kb742616.1_12593_mature | | kb742616.1_12593_mature | | actccacgctctcctctgcagg | 1 | | | 8 | | 1 | | 1 | | 2 | | 1 | | 5 | | 0 |
| kb742701.1_6472_star | | kb742701.1_6472_star | | attcatccctcatgcgtgccc | 0 | | | 0 | | 0 | | 1 | | 0 | | 2 | | 0 | | 0 |
| kb743316.1_12305_star | | kb743316.1_12305_star | | ttatgacaacaaactggagaaa | 1 | | | 0 | | 2 | | 0 | | 0 | | 0 | | 0 | | 0 |
| kb753911.1_25543_mature | | kb753911.1_25543_mature | | actgtctttgtggaaacgct | 2 | | | 2 | | 1 | | 2 | | 1 | | 2 | | 2 | | 1 |
| kb744625.1_23131_star | | kb744625.1_23131_star | | tcttttccactcgtt | 0 | | | 0 | | 1 | | 0 | | 0 | | 0 | | 0 | | 1 |
| kb743291.1_4321_star | | kb743291.1_4321_star | | gcgtttcccttgagggatgctcagcccga | 0 | | | 2 | | 7 | | 2 | | 3 | | 1 | | 0 | | 0 |
| kb743072.1_9517_mature | | kb743072.1_9517_mature | | ctgtttctcagactgcattctgtt | 5 | | | 6 | | 3 | | 6 | | 1 | | 0 | | 1 | | 1 |
| kb743228.1_14928_mature | | kb743228.1_14928_mature | | cttctgtgaggctctgtggctgc | 5 | | | 4 | | 2 | | 1 | | 1 | | 2 | | 6 | | 0 |
| kb742907.1_4743_star | | kb742907.1_4743_star | | cctgccagctgggtgggtgctggcc | 1 | | | 1 | | 1 | | 0 | | 1 | | 0 | | 1 | | 1 |
| kb743561.1_15980_mature | | kb743561.1_15980_mature | | cgggcctttactgcaacc | 0 | | | 0 | | 3 | | 0 | | 0 | | 0 | | 0 | | 0 |
| kb742585.1_9389_mature | | kb742585.1_9389_mature | | caggacggtggccatgg | 69 | | | 245 | | 260 | | 93 | | 60 | | 23 | | 119 | | 92 |
| kb743809.1_17044_star | | kb743809.1_17044_star | | taactatcatcacttgcta | 0 | | | 0 | | 1 | | 0 | | 0 | | 0 | | 0 | | 0 |
| kb743204.1_4253_star | | kb743204.1_4253_star | | cgtctctgctttgtctatgtcct | 0 | | | 0 | | 0 | | 1 | | 1 | | 0 | | 0 | | 2 |
| kb744189.1_21386_mature | | kb744189.1_21386_mature | | cccccttccccctccgca | 7 | | | 13 | | 13 | | 6 | | 6 | | 4 | | 10 | | 10 |
| kb743733.1_16911_star | | kb743733.1_16911_star | | cttcgggggcgtgggttcg | 19 | | | 45 | | 222 | | 35 | | 28 | | 35 | | 55 | | 38 |
| kb742489.1_7333_star | | kb742489.1_7333_star | | tcgagcgcttgcccaggatatg | 0 | | | 1 | | 1 | | 0 | | 0 | | 0 | | 1 | | 2 |
| kb743013.1_13175_star | | kb743013.1_13175_star | | attttctttcaaagcagaaa | 0 | | | 1 | | 0 | | 1 | | 0 | | 0 | | 1 | | 0 |
| kb742565.1_11788_mature | | kb742565.1_11788_mature | | gggactgggtgtgcagcc | 1 | | | 5 | | 2 | | 7 | | 2 | | 0 | | 1 | | 1 |
| kb744042.1_11203_star | | kb744042.1_11203_star | | aagacgttagtctgta | 1 | | | 0 | | 2 | | 0 | | 0 | | 0 | | 0 | | 1 |
| kb744706.1_23010_mature | | kb744706.1_23010_mature | | ttctgactagattgctctgttc | 0 | | | 2 | | 3 | | 1 | | 1 | | 0 | | 0 | | 4 |
| kb745269.1_19546_star | | kb745269.1_19546_star | | ggcttgctcagtatgtatgca | 0 | | | 1 | | 1 | | 0 | | 0 | | 0 | | 1 | | 0 |
| kb742393.1_17350_mature | | kb742393.1_17350_mature | | aactatcgggctagatggag | 0 | | | 0 | | 0 | | 0 | | 0 | | 3 | | 1 | | 1 |
| kb743161.1_22926_star | | kb743161.1_22926_star | | agcacatcgaccggcaggtgagagcc | 0 | | | 2 | | 0 | | 1 | | 5 | | 2 | | 0 | | 0 |
| kb743773.1_18974_mature | | kb743773.1_18974_mature | | ttatactgtggtgcccac | 7 | | | 66 | | 0 | | 77 | | 13 | | 2 | | 8 | | 6 |
| kb743558.1_17512_mature | | kb743558.1_17512_mature | | tctaaagtccctgttccttcct | 3 | | | 1 | | 2 | | 3 | | 5 | | 1 | | 5 | | 2 |
| kb743611.1_22297_star | | kb743611.1_22297_star | | tcgactaggattgtattctaa | 2 | | | 1 | | 2 | | 1 | | 0 | | 0 | | 2 | | 2 |
| kb742871.1_8817_star | | kb742871.1_8817_star | | gaaaatccacagctggatgaa | 0 | | | 0 | | 1 | | 0 | | 0 | | 1 | | 1 | | 0 |
| kb744430.1_14512_star | | kb744430.1_14512_star | | ggagacaaagcattttggccatc | 0 | | | 0 | | 0 | | 1 | | 0 | | 0 | | 1 | | 0 |
| kb743519.1_20634_mature | | kb743519.1_20634_mature | | caccccatgggatgtgtgcttc | 3 | | | 3 | | 3 | | 1 | | 2 | | 1 | | 1 | | 0 |
| kb742878.1_11100_mature | | kb742878.1_11100_mature | | ttagcagaacaagacata | 0 | | | 2 | | 1 | | 1 | | 1 | | 0 | | 8 | | 0 |
| kb743387.1_22887_star | | kb743387.1_22887_star | | tgcccctgcccctcgggtctgcg | 2 | | | 11 | | 0 | | 5 | | 1 | | 1 | | 1 | | 3 |
| kb746388.1_21634_mature | | kb746388.1_21634_mature | | tagagctctccttaaccctagg | 0 | | | 1 | | 3 | | 4 | | 3 | | 3 | | 1 | | 1 |
| kb743487.1_17944_star | | kb743487.1_17944_star | | aaaagtccttccaaacaatag | 2 | | | 1 | | 1 | | 1 | | 1 | | 2 | | 0 | | 0 |
| kb742883.1_21720_mature | | kb742883.1_21720_mature | | atggtcggagacttcagtctc | 0 | | | 1 | | 3 | | 0 | | 0 | | 0 | | 3 | | 0 |
| kb742873.1_1701_star | | kb742873.1_1701_star | | tctccaagcagtacagagtgtg | 1 | | | 2 | | 7 | | 0 | | 2 | | 7 | | 6 | | 2 |
| kb743446.1_11659_mature | | kb743446.1_11659_mature | | tgtgctgagtgactttgtca | 0 | | | 0 | | 4 | | 0 | | 2 | | 0 | | 0 | | 0 |
| kb742629.1_578_star | | kb742629.1_578_star | | tggcaggacagcatgaa | 0 | | | 0 | | 1 | | 0 | | 1 | | 0 | | 0 | | 0 |
| kb744739.1_20150_star | | kb744739.1_20150_star | | tgcagaatacatcagtatggaga | 0 | | | 4 | | 0 | | 1 | | 0 | | 0 | | 0 | | 0 |
| kb744105.1_20131_mature | | kb744105.1_20131_mature | | tggcgtggtgacgacgat | 4 | | | 4 | | 0 | | 7 | | 2 | | 2 | | 8 | | 4 |
| kb743073.1_9073_mature | | kb743073.1_9073_mature | | aacctgtttactgtgtatgtgt | 1 | | | 2 | | 1 | | 3 | | 1 | | 0 | | 0 | | 1 |
| kb742811.1_13121_mature | | kb742811.1_13121_mature | | gtttgtgatgatgaatt | 27 | | | 51 | | 20 | | 25 | | 17 | | 24 | | 19 | | 51 |
| kb743197.1_1142_mature | | kb743197.1_1142_mature | | ctgcgtagtgttttgaagc | 0 | | | 2 | | 2 | | 0 | | 1 | | 0 | | 0 | | 0 |
| kb743608.1_9444_mature | | kb743608.1_9444_mature | | ttctgtcaaacttttattctctct | 0 | | | 2 | | 0 | | 2 | | 0 | | 0 | | 4 | | 1 |
| kb743650.1_19110_star | | kb743650.1_19110_star | | agaaatgaaaccagaagatat | 1 | | | 2 | | 0 | | 1 | | 0 | | 1 | | 1 | | 0 |
| kb742680.1_6654_star | | kb742680.1_6654_star | | ctgcatccacatttttag | 0 | | | 0 | | 0 | | 0 | | 0 | | 1 | | 2 | | 2 |
| kb744204.1_11858_mature | | kb744204.1_11858_mature | | ttagactgcagaaggcacgtgct | 0 | | | 7 | | 3 | | 0 | | 11 | | 0 | | 3 | | 2 |
| kb743275.1_2654_star | | kb743275.1_2654_star | | gtagcccaacgtgccagtatgtca | 0 | | | 0 | | 0 | | 1 | | 0 | | 0 | | 0 | | 1 |
| kb745209.1_21437_star | | kb745209.1_21437_star | | aggcagttgctgagcgtgtgt | 0 | | | 0 | | 0 | | 1 | | 1 | | 0 | | 1 | | 0 |
| kb742404.1_2829_star | | kb742404.1_2829_star | | gggcagggttctacagctcgg | 2 | | | 2 | | 2 | | 7 | | 1 | | 0 | | 3 | | 0 |
| kb742637.1_18796_mature | | kb742637.1_18796_mature | | atgtttgactggctggataatgcc | 0 | | | 0 | | 3 | | 1 | | 0 | | 4 | | 4 | | 0 |
| kb742957.1_4280_star | | kb742957.1_4280_star | | gtagcaatctctggacctt | 1 | | | 3 | | 1 | | 0 | | 0 | | 0 | | 0 | | 1 |
| kb742773.1_1207_star | | kb742773.1_1207_star | | tcccaagctgcccctgcagaaca | 0 | | | 4 | | 4 | | 3 | | 0 | | 1 | | 1 | | 2 |
| kb742473.1_6392_star | | kb742473.1_6392_star | | ctcagtgatgtgtcttgtatgctttt | 3 | | | 2 | | 2 | | 3 | | 1 | | 0 | | 0 | | 0 |
| kb743110.1_6089_star | | kb743110.1_6089_star | | ccgggagcagcgggcagcc | 1 | | | 2 | | 2 | | 0 | | 0 | | 3 | | 1 | | 1 |
| kb744344.1_22131_mature | | kb744344.1_22131_mature | | tccgtttggtactgagctggttt | 0 | | | 2 | | 5 | | 0 | | 0 | | 1 | | 6 | | 1 |
| kb742675.1_10562_mature | | kb742675.1_10562_mature | | atcccggacgagccccca | 277 | | | 215 | | 184 | | 224 | | 118 | | 143 | | 170 | | 105 |
| kb743260.1_3343_mature | | kb743260.1_3343_mature | | tagactgggcgaagaagaa | 4 | | | 84 | | 0 | | 69 | | 26 | | 0 | | 10 | | 1 |
| kb742928.1_10828_star | | kb742928.1_10828_star | | ccttttggccagttagact | 0 | | | 0 | | 3 | | 1 | | 1 | | 0 | | 1 | | 0 |
| kb744105.1_20140_star | | kb744105.1_20140_star | | ggcagctccataaccccagta | 6 | | | 42 | | 48 | | 14 | | 2 | | 2 | | 2 | | 1 |
| kb743175.1_22039_mature | | kb743175.1_22039_mature | | tctgtctgtctgtctccttcc | 2 | | | 12 | | 8 | | 6 | | 6 | | 7 | | 9 | | 7 |
| kb742622.1_3794_star | | kb742622.1_3794_star | | tccacttttcagtccacctcg | 1 | | | 2 | | 4 | | 0 | | 2 | | 0 | | 3 | | 1 |
| kb744431.1_22739_mature | | kb744431.1_22739_mature | | tcaagaagtgtttggacg | 1 | | | 94 | | 0 | | 39 | | 6 | | 0 | | 2 | | 0 |
| kb744667.1_17803_star | | kb744667.1_17803_star | | cagttgcagcctgttctg | 0 | | | 4 | | 3 | | 1 | | 0 | | 0 | | 1 | | 0 |
| kb743526.1_18254_star | | kb743526.1_18254_star | | aggctaaactgacatcag | 0 | | | 0 | | 0 | | 0 | | 0 | | 0 | | 1 | | 0 |
| kb743301.1_2054_star | | kb743301.1_2054_star | | gagatgtgggagtttggcc | 0 | | | 0 | | 0 | | 1 | | 0 | | 0 | | 0 | | 0 |
| kb744621.1_10146_star | | kb744621.1_10146_star | | gcagggttcttccacaggcggcag | 4 | | | 24 | | 5 | | 31 | | 22 | | 3 | | 5 | | 3 |
| kb746368.1_21805_mature | | kb746368.1_21805_mature | | ctgctcgcgaaggaggaaa | 1 | | | 4 | | 0 | | 0 | | 0 | | 0 | | 1 | | 0 |
| kb742410.1_18149_mature | | kb742410.1_18149_mature | | aacaaatgatcgtgacctgccacc | 0 | | | 2 | | 4 | | 1 | | 2 | | 0 | | 2 | | 0 |
| kb742444.1_7985_mature | | kb742444.1_7985_mature | | aggattgacagattgag | 8 | | | 8 | | 12 | | 6 | | 0 | | 3 | | 2 | | 2 |
| kb743321.1_19113_mature | | kb743321.1_19113_mature | | tctgatgtctgttgcgaac | 4 | | | 6 | | 2 | | 3 | | 0 | | 0 | | 3 | | 0 |
| kb743771.1_8033_mature | | kb743771.1_8033_mature | | tgagagtgactgttgcttgcc | 1 | | | 8 | | 3 | | 8 | | 3 | | 2 | | 4 | | 2 |
| kb744049.1_21214_star | | kb744049.1_21214_star | | tgtttgtcctgttgtggt | 0 | | | 1 | | 0 | | 1 | | 0 | | 0 | | 0 | | 0 |
| kb742678.1_15760_star | | kb742678.1_15760_star | | cagagatacaagttgcacagtaca | 0 | | | 1 | | 1 | | 1 | | 0 | | 0 | | 4 | | 0 |
| kb743454.1_18579_star | | kb743454.1_18579_star | | tctgggtctggttttttg | 1 | | | 1 | | 3 | | 1 | | 0 | | 0 | | 0 | | 0 |
| kb743158.1_2885_star | | kb743158.1_2885_star | | tgaagggagagaggaaatgatatcc | 2 | | | 3 | | 2 | | 3 | | 1 | | 0 | | 2 | | 0 |
| kb744430.1_14512_mature | | kb744430.1_14512_mature | | tggctgcagtgcatgtctctga | 11 | | | 8 | | 27 | | 5 | | 26 | | 8 | | 14 | | 6 |
| kb742685.1_1599_mature | | kb742685.1_1599_mature | | cgtgtactgtgtcataaa | 0 | | | 1 | | 2 | | 6 | | 0 | | 0 | | 0 | | 0 |
| kb743282.1_15712_mature | | kb743282.1_15712_mature | | ttgaaagtgactgtgcagg | 1 | | | 7 | | 6 | | 4 | | 6 | | 1 | | 9 | | 1 |
| kb742823.1_13495_mature | | kb742823.1_13495_mature | | catcgacctcatcgtccgctgtg | 8 | | | 26 | | 8 | | 6 | | 7 | | 11 | | 3 | | 25 |
| kb742823.1_13488_star | | kb742823.1_13488_star | | aaatgaagcgtggggtggct | 2 | | | 0 | | 4 | | 0 | | 0 | | 2 | | 0 | | 0 |
| kb742873.1_1648_mature | | kb742873.1_1648_mature | | caagtggtctggctacatcgtc | 0 | | | 0 | | 3 | | 0 | | 5 | | 0 | | 1 | | 0 |
| kb742486.1_15041_star | | kb742486.1_15041_star | | cagtaaaatgaaaatcatac | 0 | | | 0 | | 2 | | 0 | | 0 | | 0 | | 0 | | 0 |
| kb742928.1_10835_mature | | kb742928.1_10835_mature | | aggattgtttgttaccttt | 3 | | | 18 | | 1 | | 57 | | 7 | | 3 | | 3 | | 0 |
| kb743454.1_18572_mature | | kb743454.1_18572_mature | | agaagaggagggagacgg | 0 | | | 1 | | 2 | | 4 | | 0 | | 1 | | 0 | | 1 |
| kb743158.1_2920_mature | | kb743158.1_2920_mature | | agtagctgaatgaaagaca | 0 | | | 3 | | 3 | | 0 | | 1 | | 0 | | 0 | | 0 |
| kb743529.1_12860_star | | kb743529.1_12860_star | | gggaggcacgaggggagta | 0 | | | 2 | | 2 | | 0 | | 0 | | 1 | | 1 | | 1 |
| kb743308.1_7977_mature | | kb743308.1_7977_mature | | catttaaagtggtacgtg | 4 | | | 3 | | 15 | | 5 | | 7 | | 0 | | 2 | | 1 |
| kb743612.1_12155_mature | | kb743612.1_12155_mature | | acaccgttgaagtctgaagctttg | 1 | | | 4 | | 1 | | 1 | | 0 | | 0 | | 1 | | 0 |
| kb752331.1_25236_mature | | kb752331.1_25236_mature | | ctgggctgtgttgtgcaa | 6 | | | 14 | | 0 | | 30 | | 6 | | 1 | | 8 | | 3 |
| kb742599.1_14531_star | | kb742599.1_14531_star | | acacactattgacacaggcta | 1 | | | 0 | | 0 | | 1 | | 0 | | 0 | | 1 | | 0 |
| kb742809.1_13885_star | | kb742809.1_13885_star | | atttctatgactacattgtgc | 0 | | | 0 | | 0 | | 0 | | 0 | | 2 | | 1 | | 0 |
| kb743161.1_22927_mature | | kb743161.1_22927_mature | | ctaatggacaaggcactg | 0 | | | 1 | | 8 | | 4 | | 2 | | 1 | | 17 | | 2 |
| kb743359.1_15235_mature | | kb743359.1_15235_mature | | cttccgcgtcaatgtcatccacg | 0 | | | 1 | | 2 | | 3 | | 1 | | 1 | | 3 | | 1 |
| kb742712.1_8601_mature | | kb742712.1_8601_mature | | ctcgtgtgctaatgaagtgat | 1 | | | 0 | | 2 | | 3 | | 0 | | 0 | | 1 | | 0 |
| kb744160.1_15417_star | | kb744160.1_15417_star | | ctacagtgaccacactacttc | 0 | | | 0 | | 0 | | 4 | | 2 | | 0 | | 1 | | 0 |
| kb743581.1_20330_mature | | kb743581.1_20330_mature | | ttgggattggggacggggc | 5 | | | 1 | | 1 | | 0 | | 0 | | 0 | | 0 | | 0 |
| kb744667.1_17803_mature | | kb744667.1_17803_mature | | gacgtgagcgcggctgtg | 0 | | | 0 | | 2 | | 0 | | 2 | | 0 | | 0 | | 0 |
| kb742808.1_110_star | | kb742808.1_110_star | | ctcttcccttagtgctctgc | 0 | | | 2 | | 2 | | 3 | | 2 | | 0 | | 0 | | 0 |
| kb743048.1_8305_mature | | kb743048.1_8305_mature | | gtgttgcttttctgatttta | 2 | | | 2 | | 3 | | 0 | | 5 | | 2 | | 3 | | 1 |
| kb743534.1_15861_star | | kb743534.1_15861_star | | ttttccagtcactgtag | 0 | | | 0 | | 0 | | 0 | | 0 | | 0 | | 2 | | 0 |
| kb745336.1_21549_star | | kb745336.1_21549_star | | tctgtatatgtggtggttttcat | 0 | | | 2 | | 1 | | 0 | | 1 | | 0 | | 1 | | 0 |
| kb746554.1_24396_mature | | kb746554.1_24396_mature | | gagagactggccttgctgagggc | 1 | | | 5 | | 5 | | 3 | | 1 | | 0 | | 2 | | 2 |
| kb742880.1_6503_mature | | kb742880.1_6503_mature | | atgtctgtagttggtttgc | 0 | | | 0 | | 3 | | 1 | | 0 | | 1 | | 3 | | 2 |
| kb747464.1_24596_star | | kb747464.1_24596_star | | aaatgtagttaaactattcctaatgttt | 0 | | | 1 | | 2 | | 1 | | 1 | | 2 | | 0 | | 0 |
| kb743204.1_4203_star | | kb743204.1_4203_star | | ttcagggcctggagcacaagtctt | 9 | | | 32 | | 4 | | 23 | | 3 | | 4 | | 10 | | 7 |
| kb743176.1_3940_star | | kb743176.1_3940_star | | aacagacattctgtgattaaattgtagg | 5 | | | 19 | | 10 | | 10 | | 6 | | 1 | | 15 | | 7 |
| kb742884.1_16504_star | | kb742884.1_16504_star | | tatgtacattttagtctttgt | 1 | | | 3 | | 1 | | 0 | | 0 | | 0 | | 1 | | 0 |
| kb742522.1_6432_star | | kb742522.1_6432_star | | ccgctgtcatgtttctgtcgccg | 1 | | | 3 | | 1 | | 1 | | 0 | | 1 | | 0 | | 0 |
| kb743226.1_4873_mature | | kb743226.1_4873_mature | | tgagcgatgacagtttg | 1 | | | 0 | | 0 | | 0 | | 0 | | 0 | | 0 | | 0 |
| kb742808.1_141_mature | | kb742808.1_141_mature | | ttgatctgttctgaaaaataac | 2 | | | 10 | | 7 | | 2 | | 1 | | 3 | | 10 | | 2 |
| kb743109.1_14533_mature | | kb743109.1_14533_mature | | gtgtgggctaaagagatttt | 1 | | | 4 | | 3 | | 1 | | 1 | | 0 | | 3 | | 0 |
| kb743058.1_6261_star | | kb743058.1_6261_star | | tatacacacacgcacacatgca | 1 | | | 2 | | 4 | | 1 | | 1 | | 2 | | 4 | | 3 |
| kb742439.1_3150_star | | kb742439.1_3150_star | | ggtttaaagtttgtatgttatgtcttctga | 2 | | | 27 | | 4 | | 21 | | 8 | | 4 | | 3 | | 3 |
| kb744621.1_10146_mature | | kb744621.1_10146_mature | | tccgtcggtgcagggccacctgcta | 30 | | | 188 | | 3 | | 157 | | 48 | | 8 | | 22 | | 18 |
| kb742833.1_31_star | | kb742833.1_31_star | | ttcttttttaaatttcaat | 0 | | | 1 | | 0 | | 0 | | 0 | | 1 | | 0 | | 0 |
| kb743399.1_14114_mature | | kb743399.1_14114_mature | | caagaactccaacagaat | 5 | | | 2 | | 0 | | 3 | | 0 | | 1 | | 4 | | 0 |
| kb743197.1_1131_mature | | kb743197.1_1131_mature | | gtttattgatgcagaattt | 1 | | | 4 | | 3 | | 0 | | 0 | | 0 | | 0 | | 0 |
| kb742808.1_110_mature | | kb742808.1_110_mature | | ggtggactgtaggaaggct | 3 | | | 2 | | 0 | | 1 | | 2 | | 2 | | 3 | | 0 |
| kb742596.1_13317_star | | kb742596.1_13317_star | | acagagatgatgagggga | 1 | | | 0 | | 1 | | 0 | | 0 | | 0 | | 0 | | 0 |
| kb742439.1_3137_star | | kb742439.1_3137_star | | ttcagcatttcccttctt | 0 | | | 1 | | 1 | | 0 | | 1 | | 0 | | 2 | | 0 |
| kb742446.1_12955_mature | | kb742446.1_12955_mature | | tcagaggggtgtaccagg | 24 | | | 64 | | 2 | | 95 | | 12 | | 0 | | 12 | | 2 |
| kb742449.1_19449_mature | | kb742449.1_19449_mature | | aggtgtttgactttgagct | 0 | | | 2 | | 1 | | 3 | | 1 | | 2 | | 4 | | 0 |
| kb743601.1_14592_star | | kb743601.1_14592_star | | atgttcagttaaaaacacttt | 3 | | | 2 | | 2 | | 1 | | 4 | | 3 | | 0 | | 0 |
| kb742553.1_13406_mature | | kb742553.1_13406_mature | | tctgtagccacctttggtccatt | 23 | | | 53 | | 20 | | 26 | | 37 | | 35 | | 25 | | 40 |
| kb743829.1_10880_mature | | kb743829.1_10880_mature | | aaatggtgctgatcctgag | 0 | | | 6 | | 5 | | 0 | | 3 | | 0 | | 2 | | 0 |
| kb742393.1_17361_mature | | kb742393.1_17361_mature | | ttatccagaatctgaccgacc | 2 | | | 2 | | 2 | | 0 | | 1 | | 4 | | 0 | | 0 |
| kb743833.1_10822_mature | | kb743833.1_10822_mature | | atgatcttagagctctt | 3 | | | 16 | | 1 | | 13 | | 1 | | 5 | | 6 | | 1 |
| kb742542.1_13728_mature | | kb742542.1_13728_mature | | ctgttggtagttatgact | 0 | | | 0 | | 1 | | 0 | | 0 | | 0 | | 3 | | 0 |
| kb744206.1_21345_mature | | kb744206.1_21345_mature | | tttgaggagaatagtactga | 1 | | | 5 | | 3 | | 0 | | 1 | | 0 | | 2 | | 0 |
| kb742833.1_18_star | | kb742833.1_18_star | | tagtaaagatgtccacgcatatga | 0 | | | 4 | | 2 | | 1 | | 1 | | 0 | | 0 | | 0 |
| kb743007.1_12740_star | | kb743007.1_12740_star | | agtatgtcactagtttgat | 0 | | | 3 | | 0 | | 0 | | 0 | | 0 | | 0 | | 0 |
| kb742887.1_1506_star | | kb742887.1_1506_star | | aaatttctcaggtgaggtcagtag | 0 | | | 1 | | 1 | | 0 | | 0 | | 0 | | 1 | | 1 |
| kb743609.1_4586_star | | kb743609.1_4586_star | | ttaattctaattctatt | 0 | | | 0 | | 0 | | 1 | | 0 | | 0 | | 0 | | 0 |
| kb744927.1_18238_mature | | kb744927.1_18238_mature | | cgtgcgctgtctgggct | 0 | | | 5 | | 0 | | 1 | | 1 | | 1 | | 0 | | 0 |
| kb744435.1_16060_mature | | kb744435.1_16060_mature | | gagggctcagtaacactg | 0 | | | 5 | | 1 | | 2 | | 1 | | 2 | | 2 | | 1 |
| kb743260.1_3345_star | | kb743260.1_3345_star | | atactttttttgcatctgta | 0 | | | 1 | | 1 | | 0 | | 1 | | 0 | | 0 | | 0 |
| kb743773.1_18974_star | | kb743773.1_18974_star | | ctgcacacagtactcaagga | 4 | | | 37 | | 3 | | 36 | | 12 | | 0 | | 5 | | 2 |
| kb743686.1_3399_mature | | kb743686.1_3399_mature | | ctcacaatagggaactcagtg | 1 | | | 11 | | 0 | | 0 | | 5 | | 1 | | 3 | | 6 |
| kb743177.1_6598_mature | | kb743177.1_6598_mature | | ttgactgtgctcaagaaa | 1 | | | 13 | | 6 | | 7 | | 3 | | 0 | | 10 | | 2 |
| kb744429.1_15133_star | | kb744429.1_15133_star | | ccttgttccagttgtt | 2 | | | 1 | | 0 | | 0 | | 0 | | 0 | | 0 | | 0 |
| kb743121.1_14795_mature | | kb743121.1_14795_mature | | taggaccaagcagcctgcagcttcc | 4 | | | 5 | | 5 | | 4 | | 2 | | 3 | | 5 | | 4 |
| kb742833.1_47_mature | | kb742833.1_47_mature | | aggctttgatttttttaa | 0 | | | 3 | | 0 | | 0 | | 0 | | 0 | | 0 | | 0 |
| kb743471.1_15461_mature | | kb743471.1_15461_mature | | gtggggacggggacacggggtgc | 1 | | | 2 | | 6 | | 5 | | 2 | | 0 | | 3 | | 4 |
| kb742878.1_11095_mature | | kb742878.1_11095_mature | | gcagagagggagagtgg | 0 | | | 0 | | 0 | | 0 | | 1 | | 0 | | 0 | | 0 |
| kb742633.1_16972_star | | kb742633.1_16972_star | | gaagccaaaaatcccacagcatc | 0 | | | 4 | | 3 | | 3 | | 0 | | 2 | | 0 | | 1 |
| kb743441.1_16401_mature | | kb743441.1_16401_mature | | ttgcacttggctttgaa | 1 | | | 9 | | 0 | | 4 | | 3 | | 0 | | 0 | | 0 |
| kb744185.1_18095_mature | | kb744185.1_18095_mature | | cttgctgtagctgagca | 506 | | | 4056 | | 34 | | 3238 | | 177 | | 87 | | 82 | | 23 |
| kb802439.1_25550_mature | | kb802439.1_25550_mature | | actggagctggtgctgcacgagc | 4 | | | 8 | | 16 | | 6 | | 6 | | 0 | | 5 | | 5 |
| kb743534.1_15861_mature | kb743534.1_15861_mature | | attttgactgtaaagca | | | 0 | | | 2 | | 2 | | 5 | | 2 | | 0 | | 6 | 1 |
| kb743226.1_4867_star | kb743226.1_4867_star | | cctcgagcagccgtgcaga | | | 0 | | | 0 | | 1 | | 1 | | 1 | | 0 | | 0 | 0 |
| kb744332.1_17329_star | kb744332.1_17329_star | | cagaccctgccagaaga | | | 1 | | | 1 | | 0 | | 3 | | 2 | | 0 | | 0 | 0 |
| kb742975.1_9901_star | kb742975.1_9901_star | | tgcttgctgcaggtgagcttgcca | | | 0 | | | 2 | | 3 | | 1 | | 2 | | 2 | | 0 | 0 |
| kb744248.1_19315_mature | kb744248.1_19315_mature | | tggtgtgttgtcgtgttgctt | | | 2 | | | 5 | | 3 | | 3 | | 0 | | 0 | | 1 | 0 |
| kb742870.1_17110_mature | kb742870.1_17110_mature | | ccaggccggccgtaggatg | | | 1 | | | 0 | | 4 | | 1 | | 0 | | 0 | | 1 | 1 |
| kb744292.1_12777_mature | kb744292.1_12777_mature | | aggtaatgtgtttaagctct | | | 0 | | | 0 | | 0 | | 0 | | 0 | | 1 | | 4 | 0 |
| kb742411.1_7603_mature | kb742411.1_7603_mature | | tattctcagactgtcttaga | | | 5 | | | 9 | | 3 | | 9 | | 0 | | 3 | | 3 | 1 |
| kb743097.1_10493_star | kb743097.1_10493_star | | ttgtctttttgtctgtcctca | | | 1 | | | 10 | | 7 | | 2 | | 1 | | 5 | | 3 | 1 |
| kb742477.1_14355_mature | kb742477.1_14355_mature | | aaagaagcgtcagtattgc | | | 0 | | | 0 | | 3 | | 0 | | 0 | | 0 | | 1 | 0 |
| kb742585.1_9431_mature | kb742585.1_9431_mature | | gtgctggcgtgacactgt | | | 0 | | | 4 | | 1 | | 0 | | 1 | | 0 | | 1 | 0 |
| kb744259.1_24637_star | kb744259.1_24637_star | | ccaaaggtcaagaagcttcagc | | | 0 | | | 0 | | 1 | | 0 | | 0 | | 0 | | 2 | 1 |
| kb743153.1_6808_star | kb743153.1_6808_star | | caaagtgctcggttacctgc | | | 0 | | | 0 | | 0 | | 0 | | 0 | | 0 | | 1 | 0 |
| kb742671.1_7172_star | kb742671.1_7172_star | | gtgtgtgttatgtttgag | | | 0 | | | 0 | | 0 | | 1 | | 0 | | 0 | | 0 | 1 |
| kb743359.1_15222_star | kb743359.1_15222_star | | ggcgtcgctcctcagagctgttttgca | | | 0 | | | 3 | | 2 | | 2 | | 2 | | 2 | | 4 | 1 |
| kb742750.1_2074_star | kb742750.1_2074_star | | acctgcactggacctg | | | 0 | | | 0 | | 0 | | 0 | | 0 | | 1 | | 0 | 0 |
| kb742522.1_6436_mature | kb742522.1_6436_mature | | gaaaccaagcgtcggtggagcct | | | 33 | | | 119 | | 40 | | 40 | | 42 | | 39 | | 51 | 34 |
| kb744579.1_22792_mature | kb744579.1_22792_mature | | aaaactgttgtagacttgga | | | 1 | | | 4 | | 7 | | 0 | | 2 | | 3 | | 2 | 0 |
| kb742934.1_13228_mature | kb742934.1_13228_mature | | atgacaatttgtagagagacc | | | 0 | | | 6 | | 3 | | 0 | | 0 | | 1 | | 1 | 1 |
| kb743110.1_6064_mature | kb743110.1_6064_mature | | ttctccaggctgaacagg | | | 24 | | | 259 | | 3 | | 252 | | 57 | | 2 | | 25 | 7 |
| kb748326.1_22635_star | kb748326.1_22635_star | | aagcacttttggtggaagcaactagt | | | 2 | | | 12 | | 2 | | 7 | | 2 | | 1 | | 2 | 4 |
| kb742602.1_14852_mature | kb742602.1_14852_mature | | tgatgtggctggaaagtaatcc | | | 5 | | | 31 | | 35 | | 23 | | 34 | | 5 | | 188 | 13 |
| kb743276.1_20028_star | kb743276.1_20028_star | | atgctgtagacttcaggggtt | | | 1 | | | 0 | | 1 | | 0 | | 1 | | 0 | | 0 | 0 |
| kb743113.1_6629_mature | kb743113.1_6629_mature | | agggtctcctggagcactttcc | | | 0 | | | 4 | | 0 | | 4 | | 3 | | 0 | | 0 | 4 |
| kb743343.1_14295_mature | kb743343.1_14295_mature | | ccggcatggaagaagactg | | | 2 | | | 9 | | 3 | | 13 | | 3 | | 1 | | 0 | 0 |
| kb746071.1_19965_star | kb746071.1_19965_star | | ccgcagaggagagggttgggg | | | 0 | | | 0 | | 2 | | 1 | | 0 | | 0 | | 2 | 1 |
| kb744900.1_18360_mature | kb744900.1_18360_mature | | tatctgttcaacactgtgaaa | | | 1 | | | 3 | | 2 | | 0 | | 1 | | 0 | | 2 | 1 |
| kb742757.1_11061_star | kb742757.1_11061_star | | agcactgcgtggggtttcgcagc | | | 0 | | | 2 | | 1 | | 2 | | 1 | | 2 | | 0 | 4 |
| kb743525.1_10176_mature | kb743525.1_10176_mature | | tatttgtgccaatctatt | | | 3 | | | 0 | | 0 | | 0 | | 0 | | 0 | | 1 | 1 |
| kb742844.1_17370_mature | kb742844.1_17370_mature | | cagggagggagggaggcg | | | 121 | | | 211 | | 275 | | 177 | | 56 | | 134 | | 63 | 204 |
| kb743446.1_11689_star | kb743446.1_11689_star | | tgacatgaggtgaattcccac | | | 0 | | | 2 | | 3 | | 0 | | 0 | | 2 | | 0 | 0 |
| kb744461.1_20389_mature | kb744461.1_20389_mature | | tagctggtgagttttgttgcacgtg | | | 0 | | | 2 | | 7 | | 0 | | 0 | | 1 | | 1 | 0 |
| kb742931.1_959_mature | kb742931.1_959_mature | | tgaccagggttaaaaataacctt | | | 5 | | | 1 | | 3 | | 1 | | 4 | | 1 | | 4 | 0 |
| kb742933.1_16267_star | kb742933.1_16267_star | | ttttagaagagtggtcaccgaagaag | | | 0 | | | 1 | | 0 | | 0 | | 0 | | 0 | | 0 | 0 |
| kb744000.1_19343_star | kb744000.1_19343_star | | ctagcagaaattatagc | | | 0 | | | 0 | | 1 | | 0 | | 0 | | 0 | | 0 | 0 |
| kb743525.1_10182_star | kb743525.1_10182_star | | catgtttgaggaacttcttttgc | | | 0 | | | 3 | | 1 | | 4 | | 2 | | 1 | | 4 | 0 |
| kb742772.1_18996_mature | kb742772.1_18996_mature | | ctgtcccgatcccccgctctgctgc | | | 6 | | | 3 | | 2 | | 7 | | 1 | | 0 | | 0 | 0 |
| kb744077.1_12500_mature | kb744077.1_12500_mature | | gctagctaaagaggaatt | | | 8 | | | 6 | | 3 | | 6 | | 10 | | 10 | | 75 | 4 |
| kb743100.1_5645_mature | kb743100.1_5645_mature | | ttagagcatactgtgact | | | 0 | | | 9 | | 1 | | 1 | | 0 | | 0 | | 7 | 1 |
| kb744638.1_20605_mature | kb744638.1_20605_mature | | aggaaattgtgaagaagct | | | 2 | | | 9 | | 4 | | 2 | | 0 | | 2 | | 10 | 0 |
| kb742840.1_6715_mature | kb742840.1_6715_mature | | atgagcctgaacgacgtg | | | 0 | | | 0 | | 1 | | 0 | | 1 | | 0 | | 0 | 0 |
| kb744955.1_13796_star | kb744955.1_13796_star | | acaaatagttactgcagctaagc | | | 0 | | | 3 | | 3 | | 1 | | 0 | | 1 | | 1 | 2 |
| kb742830.1_16492_mature | kb742830.1_16492_mature | | tgaggaagtccagaacttgacgt | | | 1 | | | 4 | | 5 | | 2 | | 0 | | 2 | | 0 | 2 |
| kb742677.1_7512_star | kb742677.1_7512_star | | actatccccagtgaat | | | 0 | | | 0 | | 0 | | 0 | | 0 | | 0 | | 0 | 2 |
| kb742907.1_4760_mature | kb742907.1_4760_mature | | gaattttttgaggctctg | | | 0 | | | 9 | | 2 | | 1 | | 2 | | 4 | | 5 | 3 |
| kb744429.1_15136_mature | kb744429.1_15136_mature | | aggatttggaaggattttcc | | | 0 | | | 5 | | 0 | | 2 | | 0 | | 0 | | 3 | 0 |
| kb743662.1_7528_star | kb743662.1_7528_star | | gtggagaacattgtgatgtgtgcta | | | 4 | | | 13 | | 8 | | 4 | | 5 | | 5 | | 7 | 5 |
| kb744088.1_3983_star | kb744088.1_3983_star | | ttctctgtcactaaaactc | | | 0 | | | 1 | | 0 | | 2 | | 0 | | 0 | | 0 | 0 |
| kb743226.1_4903_mature | kb743226.1_4903_mature | | tggagaagaacgtgaacc | | | 2 | | | 5 | | 106 | | 16 | | 7 | | 2 | | 37 | 12 |
| kb745209.1_21437_mature | kb745209.1_21437_mature | | aacgtgacgactgcactgc | | | 2 | | | 0 | | 1 | | 2 | | 1 | | 0 | | 1 | 1 |
| kb742922.1_21237_mature | kb742922.1_21237_mature | | acggaagccatgtggaagaccct | | | 3 | | | 1 | | 0 | | 1 | | 0 | | 0 | | 0 | 1 |
| kb742823.1_13495_star | kb742823.1_13495_star | | caggtaccgaaggtggaagct | | | 0 | | | 1 | | 1 | | 2 | | 0 | | 0 | | 3 | 0 |
| kb743858.1_24463_star | kb743858.1_24463_star | | aaccaagttctccttagcagcatgtcat | | | 1 | | | 1 | | 1 | | 3 | | 1 | | 0 | | 2 | 1 |
| kb742757.1_11077_mature | kb742757.1_11077_mature | | aaggacctggaggaggtg | | | 0 | | | 1 | | 0 | | 6 | | 4 | | 0 | | 2 | 9 |
| kb803155.1_25524_star | kb803155.1_25524_star | | gctgccctgtgggagtaa | | | 0 | | | 0 | | 1 | | 0 | | 1 | | 0 | | 0 | 0 |
| kb744353.1_15015_mature | kb744353.1_15015_mature | | ccctgcgggccttgagaca | | | 0 | | | 0 | | 1 | | 35 | | 1 | | 1 | | 0 | 0 |
| kb743809.1_17031_mature | kb743809.1_17031_mature | | cgtgcgtggcttacctggaagt | | | 4 | | | 1 | | 0 | | 2 | | 2 | | 2 | | 3 | 1 |
| kb742969.1_1017_mature | kb742969.1_1017_mature | | agccttgaactcttcagtt | | | 0 | | | 2 | | 0 | | 2 | | 0 | | 0 | | 2 | 4 |
| kb744039.1_22842_mature | kb744039.1_22842_mature | | tggttcaggctgcagcagtt | | | 0 | | | 4 | | 0 | | 0 | | 3 | | 1 | | 3 | 3 |
| kb743357.1_14743_star | kb743357.1_14743_star | | aggcagcgagaccagcttag | | | 1 | | | 1 | | 0 | | 0 | | 2 | | 1 | | 0 | 1 |
| kb742465.1_20588_star | kb742465.1_20588_star | | tccctgtctttctgaatgacggg | | | 1 | | | 3 | | 1 | | 7 | | 3 | | 1 | | 1 | 0 |
| kb745135.1_22103_star | kb745135.1_22103_star | | ttcccactgcgcatta | | | 1 | | | 0 | | 1 | | 0 | | 0 | | 0 | | 0 | 0 |
| kb744899.1_8646_mature | kb744899.1_8646_mature | | tgagctctgccttggtgaagtg | | | 0 | | | 2 | | 0 | | 3 | | 1 | | 0 | | 3 | 1 |
| kb742504.1_8579_star | kb742504.1_8579_star | | ttctgttgtagttcgagat | | | 0 | | | 0 | | 1 | | 3 | | 1 | | 1 | | 2 | 0 |
| kb742580.1_2720_star | kb742580.1_2720_star | | gggcacggatatgtctttgtt | | | 0 | | | 0 | | 0 | | 1 | | 0 | | 1 | | 0 | 0 |
| kb742464.1_2344_star | kb742464.1_2344_star | | gccaggcgcaccccgctg | | | 1 | | | 0 | | 0 | | 0 | | 0 | | 0 | | 0 | 0 |
| kb742957.1_4280_mature | kb742957.1_4280_mature | | tgttctgatgatgtgtgccttca | | | 1 | | | 3 | | 7 | | 2 | | 1 | | 3 | | 5 | 0 |
| kb742890.1_15883_mature | kb742890.1_15883_mature | | ttggattctgtttgaaac | | | 0 | | | 7 | | 2 | | 5 | | 4 | | 1 | | 3 | 0 |
| kb742499.1_8270_mature | kb742499.1_8270_mature | | aagtgaaggttgtatgact | | | 0 | | | 3 | | 3 | | 2 | | 1 | | 1 | | 0 | 0 |
| kb742807.1_16449_star | kb742807.1_16449_star | | agggcagcgtgcacagagact | | | 0 | | | 1 | | 0 | | 0 | | 0 | | 2 | | 0 | 1 |
| kb743301.1_2054_mature | kb743301.1_2054_mature | | ttagactcttaacgctgag | | | 0 | | | 25 | | 0 | | 18 | | 3 | | 0 | | 1 | 0 |
| kb742554.1_1757_star | kb742554.1_1757_star | | gatagtgggctatgacacacaa | | | 1 | | | 0 | | 0 | | 0 | | 0 | | 1 | | 0 | 0 |
| kb742853.1_2256_star | kb742853.1_2256_star | | ccattttgtttgatcatctt | | | 1 | | | 0 | | 0 | | 4 | | 0 | | 0 | | 0 | 0 |
| kb742819.1_21534_mature | kb742819.1_21534_mature | | tggatggtttagtgaggt | | | 12 | | | 16 | | 17 | | 8 | | 6 | | 8 | | 12 | 11 |
| kb742598.1_14469_mature | kb742598.1_14469_mature | | acagcccaggactgaaga | | | 0 | | | 0 | | 1 | | 4 | | 0 | | 0 | | 0 | 0 |
| kb742469.1_19787_mature | kb742469.1_19787_mature | | ttggactcgatgatccttatggg | | | 82 | | | 1469 | | 8 | | 1875 | | 281 | | 7 | | 123 | 37 |
| kb818085.1_24982_star | kb818085.1_24982_star | | tacttggctgggatttca | | | 0 | | | 1 | | 0 | | 0 | | 0 | | 1 | | 3 | 0 |
| kb806524.1_25436_mature | kb806524.1_25436_mature | | cctacaagcacggactgt | | | 0 | | | 0 | | 3 | | 1 | | 0 | | 0 | | 1 | 2 |
| kb742856.1_19615_mature | kb742856.1_19615_mature | | tggttctgtactgtgtac | | | 5 | | | 6 | | 3 | | 5 | | 5 | | 5 | | 33 | 4 |
| kb742957.1_4301_mature | kb742957.1_4301_mature | | ttgcttttactctgaaggaca | | | 5 | | | 4 | | 7 | | 3 | | 1 | | 3 | | 2 | 0 |
| kb742844.1_17370_star | kb742844.1_17370_star | | cctcctcagtctgcc | | | 1 | | | 1 | | 1 | | 0 | | 0 | | 0 | | 1 | 0 |
| kb810642.1_25334_mature | kb810642.1_25334_mature | | aattcgtcaccacttggtgga | | | 0 | | | 0 | | 3 | | 1 | | 0 | | 0 | | 0 | 3 |
| kb744204.1_11870_mature | kb744204.1_11870_mature | | ttggactcttgcttttgca | | | 2 | | | 2 | | 8 | | 1 | | 0 | | 1 | | 1 | 0 |
| kb744385.1_24162_mature | kb744385.1_24162_mature | | tgtcccttcgggctgtctcacg | | | 6 | | | 1 | | 9 | | 1 | | 0 | | 0 | | 4 | 7 |
| kb743569.1_19227_mature | kb743569.1_19227_mature | | gatcagctggagccagttaac | | | 0 | | | 0 | | 7 | | 1 | | 0 | | 0 | | 1 | 0 |
| kb742744.1_6102_mature | kb742744.1_6102_mature | | agactgtgtgacctgtgc | | | 0 | | | 10 | | 1 | | 2 | | 0 | | 4 | | 4 | 2 |
| kb743396.1_14410_mature | kb743396.1_14410_mature | | aagttttgtttactggtgtgagca | | | 3 | | | 0 | | 3 | | 2 | | 3 | | 2 | | 1 | 2 |
| kb743733.1_16906_mature | kb743733.1_16906_mature | | gacgttagggcctggacct | | | 1 | | | 1 | | 6 | | 0 | | 0 | | 0 | | 0 | 1 |
| kb744656.1_20746_star | kb744656.1_20746_star | | aaagatggcagtgcatgttt | | | 0 | | | 1 | | 0 | | 0 | | 0 | | 0 | | 1 | 0 |
| kb742655.1_5346_star | kb742655.1_5346_star | | tcatccgggtgagcacat | | | 0 | | | 0 | | 1 | | 0 | | 0 | | 0 | | 0 | 0 |
| kb743024.1_15400_star | kb743024.1_15400_star | | tttaattgtttctgctatgtgtt | | | 1 | | | 0 | | 0 | | 1 | | 0 | | 1 | | 1 | 0 |
| kb743670.1_4933_star | kb743670.1_4933_star | | gacaggaatttgcatcttctcaa | | | 1 | | | 1 | | 1 | | 0 | | 1 | | 1 | | 5 | 0 |
| kb742833.1_45_star | kb742833.1_45_star | | taggaggaaacaggcactt | | | 1 | | | 1 | | 2 | | 0 | | 0 | | 0 | | 1 | 0 |
| kb746572.1_23773_mature | kb746572.1_23773_mature | | agggtctgggtgtctctgtctgt | | | 5 | | | 3 | | 3 | | 7 | | 2 | | 3 | | 15 | 2 |
| kb742543.1_11193_mature | kb742543.1_11193_mature | | ttaacgctgtacatgactgaggtt | | | 1 | | | 2 | | 0 | | 0 | | 0 | | 0 | | 2 | 1 |
| kb742811.1_13103_star | kb742811.1_13103_star | | tccagagaaggactacaaagctggtg | | | 7 | | | 15 | | 2 | | 34 | | 23 | | 1 | | 3 | 2 |
| kb742418.1_2464_star | kb742418.1_2464_star | | acagtcgtttataaaactcttttc | | | 3 | | | 1 | | 3 | | 6 | | 1 | | 2 | | 2 | 0 |
| kb742904.1_12935_mature | kb742904.1_12935_mature | | aggtaggacagtggatctg | | | 0 | | | 0 | | 3 | | 0 | | 2 | | 0 | | 0 | 0 |
| kb744232.1_13272_star | kb744232.1_13272_star | | gggtgagaatatct | | | 1 | | | 0 | | 0 | | 0 | | 0 | | 1 | | 0 | 0 |
| kb742418.1_2435_mature | kb742418.1_2435_mature | | gactagcctcctcctgttgct | | | 1 | | | 0 | | 0 | | 2 | | 4 | | 1 | | 0 | 1 |
| kb743525.1_10189_star | kb743525.1_10189_star | | cagtaagattaaaaggaacat | | | 0 | | | 2 | | 2 | | 1 | | 1 | | 1 | | 0 | 0 |
| kb742543.1_11193_star | kb742543.1_11193_star | | ccacagcatgacagcattacca | | | 0 | | | 3 | | 1 | | 7 | | 4 | | 0 | | 2 | 2 |
| kb742887.1_1518_mature | kb742887.1_1518_mature | | tcaagactgctttctgctttgggt | | | 20 | | | 98 | | 6 | | 120 | | 18 | | 6 | | 22 | 13 |
| kb744214.1_20085_star | kb744214.1_20085_star | | gctctccgctaatcacac | | | 0 | | | 2 | | 0 | | 0 | | 0 | | 0 | | 0 | 0 |
| kb742756.1_19117_mature | kb742756.1_19117_mature | | agttgttgaactctgcgcgtg | | | 1 | | | 3 | | 3 | | 3 | | 1 | | 2 | | 0 | 0 |
| kb743366.1_17984_mature | kb743366.1_17984_mature | | agacactagtgtagactg | | | 0 | | | 0 | | 0 | | 0 | | 0 | | 0 | | 0 | 5 |
| kb743180.1_19957_star | kb743180.1_19957_star | | agagcacccgtctcgagct | | | 0 | | | 3 | | 0 | | 1 | | 1 | | 0 | | 1 | 0 |
| kb743037.1_11805_star | kb743037.1_11805_star | | gtgggatttggtaggtcaggct | | | 0 | | | 2 | | 1 | | 0 | | 1 | | 1 | | 1 | 1 |
| kb742477.1_14364_star | kb742477.1_14364_star | | gcatgcacacagcagccgaattc | | | 0 | | | 8 | | 1 | | 1 | | 2 | | 0 | | 0 | 0 |
| kb745404.1_16854_mature | kb745404.1_16854_mature | | gtctctggatggtgaatt | | | 0 | | | 3 | | 1 | | 0 | | 1 | | 1 | | 1 | 1 |
| kb742712.1_8598_star | kb742712.1_8598_star | | tgtacacaaagagttggca | | | 0 | | | 0 | | 1 | | 0 | | 0 | | 0 | | 0 | 0 |
| kb743611.1_22297_mature | kb743611.1_22297_mature | | cacaggcaatctctggagaag | | | 0 | | | 6 | | 6 | | 4 | | 2 | | 0 | | 3 | 1 |
| kb742807.1_16447_mature | kb742807.1_16447_mature | | tgagttgctgtcggcgtgtgct | | | 0 | | | 3 | | 0 | | 2 | | 1 | | 1 | | 0 | 1 |
| kb742801.1_3454_mature | kb742801.1_3454_mature | | gtgattgccaggtactgaat | | | 0 | | | 1 | | 0 | | 0 | | 0 | | 0 | | 0 | 1 |
| kb743446.1_11674_mature | kb743446.1_11674_mature | | ctgtctttctgtataagct | | | 2 | | | 0 | | 2 | | 6 | | 0 | | 1 | | 4 | 0 |
| kb742389.1_11430_mature | kb742389.1_11430_mature | | gaacaagaaaacaaggtcctct | | | 0 | | | 2 | | 5 | | 0 | | 1 | | 0 | | 3 | 1 |
| kb743110.1_6039_star | kb743110.1_6039_star | | atccaatagttttgaacttc | | | 0 | | | 4 | | 0 | | 0 | | 0 | | 0 | | 2 | 0 |
| kb742404.1_2876_star | kb742404.1_2876_star | | gctgaaaggtgcgctgttc | | | 0 | | | 2 | | 0 | | 2 | | 0 | | 0 | | 0 | 0 |
| kb743564.1_13643_star | kb743564.1_13643_star | | tggcattagcaaaagacaattc | | | 1 | | | 0 | | 0 | | 0 | | 1 | | 0 | | 1 | 0 |
| kb745312.1_22392_mature | kb745312.1_22392_mature | | caacgcggcatggagatgtac | | | 1 | | | 1 | | 1 | | 0 | | 0 | | 0 | | 0 | 3 |
| kb742637.1_18799_mature | kb742637.1_18799_mature | | ttcggtgctgcctcgcttgc | | | 1 | | | 3 | | 0 | | 0 | | 1 | | 0 | | 0 | 5 |
| kb742712.1_8613_star | kb742712.1_8613_star | | tgtgagatccctactcc | | | 0 | | | 0 | | 2 | | 0 | | 0 | | 0 | | 0 | 0 |
| kb742611.1_4468_mature | kb742611.1_4468_mature | | tattagaactgtgtgacta | | | 6 | | | 2 | | 3 | | 0 | | 1 | | 1 | | 2 | 0 |
| kb742627.1_5356_star | kb742627.1_5356_star | | cctgtgggcagtcagcaaaaagt | | | 0 | | | 1 | | 3 | | 1 | | 1 | | 0 | | 1 | 1 |
| kb743374.1_21334_star | kb743374.1_21334_star | | accacagtacaaaaaggaca | | | 1 | | | 3 | | 3 | | 4 | | 4 | | 0 | | 2 | 1 |
| kb743973.1_15353_mature | kb743973.1_15353_mature | | acacgtgtcttttgaactt | | | 2 | | | 5 | | 0 | | 1 | | 2 | | 1 | | 1 | 2 |
| kb744206.1_21346_star | kb744206.1_21346_star | | agcccaatactcataggaaca | | | 0 | | | 0 | | 0 | | 0 | | 1 | | 0 | | 1 | 0 |
| kb742741.1_17420_star | kb742741.1_17420_star | | cccttggagcccagccactcaa | | | 1 | | | 4 | | 5 | | 0 | | 1 | | 1 | | 0 | 4 |
| kb742915.1_23424_mature | kb742915.1_23424_mature | | tgggtctgtggctggtgac | | | 0 | | | 5 | | 2 | | 3 | | 4 | | 2 | | 1 | 0 |
| kb742595.1_8422_star | kb742595.1_8422_star | | ggggtggttcagctcacct | | | 2 | | | 1 | | 0 | | 0 | | 0 | | 0 | | 1 | 0 |
| kb744306.1_3705_mature | kb744306.1_3705_mature | | tggcggagttgatgctggtc | | | 0 | | | 0 | | 0 | | 0 | | 4 | | 0 | | 0 | 1 |
| kb744353.1_15020_star | kb744353.1_15020_star | | cgatttcactagtacctggcatct | | | 0 | | | 0 | | 0 | | 0 | | 0 | | 0 | | 2 | 0 |
| kb743569.1_19235_star | kb743569.1_19235_star | | gagcagggccgctccgtt | | | 1 | | | 0 | | 0 | | 0 | | 0 | | 0 | | 0 | 2 |
| kb742959.1_16120_mature | kb742959.1_16120_mature | | ataactgcatttgaaca | | | 1 | | | 0 | | 2 | | 1 | | 0 | | 0 | | 1 | 0 |
| kb742464.1_2344_mature | kb742464.1_2344_mature | | tcgaggagtgcctggacaacccc | | | 0 | | | 1 | | 11 | | 2 | | 2 | | 0 | | 3 | 3 |
| kb742382.1_14251_star | kb742382.1_14251_star | | atggagacgggaaaaagt | | | 0 | | | 1 | | 0 | | 0 | | 0 | | 0 | | 2 | 0 |
| kb742943.1_5715_star | kb742943.1_5715_star | | cttgtaacagtcatcaattgta | | | 2 | | | 2 | | 2 | | 3 | | 1 | | 6 | | 2 | 2 |
| kb742808.1_178_star | kb742808.1_178_star | | tgaaggacctcacccctccctagca | | | 0 | | | 3 | | 4 | | 0 | | 0 | | 0 | | 0 | 2 |
| kb742725.1_13868_star | kb742725.1_13868_star | | cctctgttttcacagt | | | 0 | | | 0 | | 0 | | 1 | | 0 | | 0 | | 0 | 0 |
| kb742627.1_5359_mature | kb742627.1_5359_mature | | ctgtgcaggattcatctgaata | | | 0 | | | 3 | | 6 | | 6 | | 2 | | 2 | | 2 | 0 |
| kb742605.1_4571_mature | kb742605.1_4571_mature | | tgggcttttctgttgaag | | | 0 | | | 3 | | 1 | | 1 | | 0 | | 2 | | 6 | 0 |
| kb743070.1_18310_star | kb743070.1_18310_star | | tctaactttgtgccatgttg | | | 0 | | | 1 | | 1 | | 0 | | 0 | | 0 | | 0 | 0 |
| kb743948.1_17925_mature | kb743948.1_17925_mature | | ttgatgtgtgaaaagtcc | | | 0 | | | 7 | | 5 | | 6 | | 1 | | 3 | | 7 | 1 |
| kb744039.1_22842_star | kb744039.1_22842_star | | ctgtcactaaaaccagg | | | 1 | | | 0 | | 0 | | 0 | | 0 | | 2 | | 0 | 0 |
| kb743108.1_15447_star | kb743108.1_15447_star | | cccgtataaaacagcttagca | | | 0 | | | 0 | | 1 | | 0 | | 0 | | 0 | | 0 | 0 |
| kb743040.1_2799_mature | kb743040.1_2799_mature | | tgggccacctctgtgatgtgacggt | | | 4 | | | 148 | | 2 | | 102 | | 1 | | 3 | | 9 | 2 |
| kb744265.1_23560_mature | kb744265.1_23560_mature | | agggacatgaaactgttggagagt | | | 29 | | | 282 | | 2 | | 385 | | 31 | | 7 | | 31 | 12 |
| kb744345.1_17163_mature | kb744345.1_17163_mature | | ctgaaggcatctctgtagctgttc | | | 1 | | | 3 | | 0 | | 3 | | 1 | | 1 | | 7 | 1 |
| kb743810.1_19135_star | kb743810.1_19135_star | | ccttgctccctgcccgggc | | | 5 | | | 6 | | 15 | | 1 | | 1 | | 2 | | 6 | 3 |
| kb742651.1_5184_mature | kb742651.1_5184_mature | | atgcatcggccgcccggg | | | 0 | | | 0 | | 0 | | 2 | | 1 | | 3 | | 0 | 1 |
| kb742648.1_9725_star | kb742648.1_9725_star | | cttagagcccacagtatgc | | | 0 | | | 1 | | 0 | | 0 | | 1 | | 0 | | 3 | 0 |
| kb742890.1_15883_star | kb742890.1_15883_star | | ttcagtgcaagtctgatg | | | 1 | | | 1 | | 2 | | 0 | | 0 | | 0 | | 0 | 0 |
| kb742730.1_8115_mature | kb742730.1_8115_mature | | cagagaaggacctgggag | | | 3 | | | 21 | | 2 | | 23 | | 4 | | 1 | | 2 | 0 |
| kb743686.1_3418_mature | kb743686.1_3418_mature | | caggactaacagtgtaata | | | 0 | | | 4 | | 3 | | 0 | | 3 | | 2 | | 1 | 0 |
| kb743357.1_14743_mature | kb743357.1_14743_mature | | aacttggtcagtgtgttctgc | | | 2 | | | 1 | | 5 | | 0 | | 1 | | 0 | | 2 | 1 |
| kb742735.1_8554_star | kb742735.1_8554_star | | taatttttaattcagattatttttg | | | 0 | | | 3 | | 1 | | 0 | | 0 | | 1 | | 0 | 1 |
| kb744033.1_9320_mature | kb744033.1_9320_mature | | aacaggagctggagatgtgc | | | 4 | | | 7 | | 4 | | 2 | | 4 | | 1 | | 3 | 1 |
| kb743183.1_7713_star | kb743183.1_7713_star | | ctcaatgaatttccctgacaccaag | | | 1 | | | 1 | | 2 | | 4 | | 1 | | 0 | | 0 | 0 |
| kb742966.1_4459_mature | kb742966.1_4459_mature | | tcttgcacaagaattggcctgt | | | 34 | | | 27 | | 39 | | 26 | | 33 | | 57 | | 62 | 22 |
| kb743645.1_18018_star | kb743645.1_18018_star | | gtgtgctagagtactcggaaa | | | 0 | | | 0 | | 0 | | 2 | | 0 | | 1 | | 0 | 1 |
| kb743197.1_1131_star | kb743197.1_1131_star | | gtcctgattgagtttacta | | | 0 | | | 0 | | 0 | | 0 | | 1 | | 0 | | 0 | 0 |
| kb743645.1_18024_mature | kb743645.1_18024_mature | | cagcacctgtgggaattag | | | 3 | | | 12 | | 1 | | 15 | | 3 | | 1 | | 3 | 0 |
| kb742618.1_11453_star | kb742618.1_11453_star | | gagtatctgtcttatttcat | | | 0 | | | 5 | | 1 | | 0 | | 3 | | 0 | | 0 | 1 |
| kb743541.1_21773_mature | kb743541.1_21773_mature | | tgtgttcttcctccctcccagg | | | 0 | | | 4 | | 5 | | 1 | | 0 | | 0 | | 4 | 1 |
| kb742605.1_4500_mature | kb742605.1_4500_mature | | tctggttgacatgctcagt | | | 2 | | | 4 | | 3 | | 3 | | 1 | | 0 | | 0 | 0 |
| kb743028.1_7250_mature | kb743028.1_7250_mature | | tgagctggctgtagaagca | | | 3 | | | 5 | | 5 | | 4 | | 3 | | 1 | | 7 | 1 |
| kb742479.1_270_mature | kb742479.1_270_mature | | atttgtgctgtcctatgaca | | | 2 | | | 3 | | 5 | | 1 | | 0 | | 0 | | 2 | 1 |
| kb742808.1_145_star | kb742808.1_145_star | | aagtgagctaaacttca | | | 0 | | | 1 | | 0 | | 0 | | 0 | | 0 | | 1 | 0 |
| kb747234.1_23193_star | kb747234.1_23193_star | | caccgtgcttgtgctcga | | | 1 | | | 1 | | 0 | | 1 | | 0 | | 1 | | 2 | 0 |
| kb744139.1_14168_mature | kb744139.1_14168_mature | | ataggaacaagcaaagcaagtt | | | 6 | | | 1 | | 26 | | 3 | | 2 | | 7 | | 0 | 2 |
| kb742554.1_1757_mature | kb742554.1_1757_mature | | gtgaagcgttccatatttt | | | 14 | | | 6 | | 2 | | 31 | | 8 | | 5 | | 29 | 7 |
| kb744345.1_17163_star | kb744345.1_17163_star | | gcagcaaagcagtagtcagcgttccagg | | | 0 | | | 2 | | 1 | | 0 | | 1 | | 1 | | 3 | 1 |
| kb743609.1_4587_mature | kb743609.1_4587_mature | | atggaaattttgcttcatctcc | | | 0 | | | 1 | | 6 | | 1 | | 0 | | 2 | | 1 | 1 |
| kb742490.1_1402_star | kb742490.1_1402_star | | cagagtggttcaggctgggatgag | | | 1 | | | 7 | | 2 | | 6 | | 0 | | 0 | | 0 | 2 |
| kb742824.1_11561_star | kb742824.1_11561_star | | aagatctcataacaaaactatcaaa | | | 0 | | | 4 | | 1 | | 2 | | 0 | | 0 | | 0 | 1 |
| kb742923.1_14524_star | kb742923.1_14524_star | | ttatacctacggatagacgc | | | 0 | | | 0 | | 0 | | 0 | | 0 | | 1 | | 0 | 0 |
| kb742697.1_16940_mature | kb742697.1_16940_mature | | aggactcgcagcaagctgtg | | | 0 | | | 3 | | 3 | | 0 | | 0 | | 0 | | 0 | 0 |
| kb742588.1_6984_star | kb742588.1_6984_star | | ccctgagaactcctggccctggccgc | | | 1 | | | 1 | | 3 | | 0 | | 0 | | 1 | | 3 | 2 |
| kb742489.1_7333_mature | kb742489.1_7333_mature | | ttttctgtgcctgtgctctgaga | | | 6 | | | 15 | | 16 | | 6 | | 0 | | 2 | | 13 | 4 |
| kb742668.1_5616_star | kb742668.1_5616_star | | cagctcagcctacgccacaaacgccaa | | | 2 | | | 5 | | 2 | | 5 | | 0 | | 1 | | 1 | 1 |
| kb743226.1_4916_star | kb743226.1_4916_star | | acacaacaaacgctgggacttga | | | 0 | | | 1 | | 3 | | 1 | | 2 | | 0 | | 0 | 0 |
| kb743458.1_14257_mature | kb743458.1_14257_mature | | tatctgattttgcctgatttc | | | 2 | | | 0 | | 1 | | 2 | | 0 | | 0 | | 4 | 0 |
| kb744725.1_16613_mature | kb744725.1_16613_mature | | tggaataataatgctgtt | | | 0 | | | 4 | | 0 | | 0 | | 1 | | 0 | | 0 | 0 |
| kb743167.1_13284_star | kb743167.1_13284_star | | cctgcagaattgcatagacagaagagt | | | 0 | | | 1 | | 2 | | 0 | | 1 | | 0 | | 1 | 1 |
| kb742678.1_15760_mature | kb742678.1_15760_mature | | tttgtggttgtagtctctgca | | | 4 | | | 10 | | 8 | | 3 | | 0 | | 5 | | 5 | 2 |
| kb743589.1_20880_star | kb743589.1_20880_star | | ggctgctctactttcc | | | 0 | | | 0 | | 0 | | 1 | | 0 | | 0 | | 0 | 0 |
| kb742794.1_18827_star | kb742794.1_18827_star | | tgccagctgtgagtgtttct | | | 0 | | | 1 | | 1 | | 0 | | 1 | | 1 | | 1 | 0 |
| kb744435.1_16058_star | kb744435.1_16058_star | | gatgtttcccgttcagatt | | | 0 | | | 1 | | 0 | | 0 | | 0 | | 0 | | 0 | 0 |
| kb743702.1_18437_mature | kb743702.1_18437_mature | | ttcttagagggacaagt | | | 3 | | | 16 | | 2 | | 1 | | 3 | | 2 | | 3 | 1 |
| kb742832.1_11519_star | kb742832.1_11519_star | | ctggaagaccagaa | | | 0 | | | 0 | | 0 | | 0 | | 0 | | 0 | | 1 | 0 |
| kb742735.1_8566_mature | kb742735.1_8566_mature | | acgacgagttcgaggacgacc | | | 4 | | | 14 | | 78 | | 11 | | 6 | | 5 | | 4 | 6 |
| kb743402.1_5917_mature | kb743402.1_5917_mature | | tgtactgttgctgatttcttct | | | 2 | | | 5 | | 6 | | 5 | | 3 | | 8 | | 4 | 5 |
| kb744139.1_14168_star | kb744139.1_14168_star | | attgctttgtttgttcctaccctctct | | | 2 | | | 1 | | 17 | | 6 | | 0 | | 4 | | 2 | 0 |
| kb746737.1_23829_star | kb746737.1_23829_star | | tactatgtttgtatctgtgtaca | | | 1 | | | 3 | | 2 | | 0 | | 2 | | 0 | | 2 | 1 |
| kb742809.1_13891_mature | kb742809.1_13891_mature | | cctcccctccgcccccc | | | 2 | | | 4 | | 5 | | 6 | | 1 | | 2 | | 2 | 11 |
| kb743662.1_7538_mature | kb743662.1_7538_mature | | cagccagctgttcaggagtgt | | | 0 | | | 3 | | 7 | | 4 | | 1 | | 0 | | 6 | 2 |
| kb742750.1_2085_star | kb742750.1_2085_star | | taagtaatgctaaagggagggattgaggt | | | 1 | | | 10 | | 1 | | 5 | | 9 | | 0 | | 0 | 2 |
| kb742929.1_337_mature | kb742929.1_337_mature | | gcgtgtatagtggagaacc | | | 2 | | | 2 | | 1 | | 0 | | 0 | | 1 | | 2 | 1 |
| kb743374.1_21337_mature | kb743374.1_21337_mature | | actatggctgtgtactgctat | | | 2 | | | 2 | | 8 | | 1 | | 0 | | 1 | | 3 | 1 |
| kb743158.1_2878_star | kb743158.1_2878_star | | attgttttgagcacccag | | | 0 | | | 0 | | 0 | | 0 | | 0 | | 0 | | 1 | 0 |
| kb742629.1_588_star | kb742629.1_588_star | | tcattaagacacactgag | | | 0 | | | 0 | | 0 | | 0 | | 0 | | 0 | | 2 | 0 |
| kb743364.1_9935_mature | kb743364.1_9935_mature | | caagtggttgggctgggagc | | | 1 | | | 4 | | 1 | | 1 | | 1 | | 3 | | 2 | 6 |
| kb743855.1_17849_mature | kb743855.1_17849_mature | | cggactggcaccaagcctcg | | | 0 | | | 0 | | 1 | | 1 | | 1 | | 1 | | 5 | 0 |
| kb742471.1_390_star | kb742471.1_390_star | | ggtaaaagagtaacacatccttg | | | 0 | | | 2 | | 2 | | 0 | | 0 | | 2 | | 1 | 0 |
| kb742481.1_5270_mature | kb742481.1_5270_mature | | ccgttgctgtccgtctggac | | | 0 | | | 1 | | 1 | | 0 | | 0 | | 3 | | 5 | 1 |
| kb743809.1_17031_star | kb743809.1_17031_star | | ctccaggcagcagaccttgcacggc | | | 1 | | | 4 | | 2 | | 1 | | 1 | | 5 | | 4 | 2 |
| kb744105.1_20091_star | kb744105.1_20091_star | | agcagatgccttcagtgccacgg | | | 2 | | | 0 | | 1 | | 0 | | 0 | | 2 | | 0 | 1 |
| kb742572.1_11741_mature | kb742572.1_11741_mature | | tcactttgtcgtagaagg | | | 35 | | | 145 | | 0 | | 176 | | 13 | | 0 | | 9 | 3 |
| kb743153.1_6806_mature | kb743153.1_6806_mature | | attttgatcttttctctggcag | | | 4 | | | 22 | | 4 | | 30 | | 3 | | 3 | | 20 | 2 |
| kb743412.1_5041_star | kb743412.1_5041_star | | acttgctaacgtgaattctac | | | 0 | | | 4 | | 0 | | 1 | | 0 | | 0 | | 0 | 0 |
| kb743109.1_14541_star | kb743109.1_14541_star | | ttcacagttcctcagacaca | | | 2 | | | 4 | | 0 | | 2 | | 1 | | 3 | | 0 | 0 |
| kb742845.1_5868_mature | kb742845.1_5868_mature | | actcatgtctgtggatgactct | | | 0 | | | 9 | | 3 | | 2 | | 3 | | 4 | | 2 | 4 |
| kb742418.1_2461_mature | kb742418.1_2461_mature | | aaatgttgggacctggtgtc | | | 0 | | | 1 | | 0 | | 0 | | 1 | | 0 | | 5 | 0 |
| kb743004.1_8916_mature | kb743004.1_8916_mature | | cgtgtgctaagcttgaca | | | 0 | | | 1 | | 0 | | 3 | | 0 | | 0 | | 0 | 0 |
| kb744049.1_21214_mature | kb744049.1_21214_mature | | tgtggcagggcaggtaaga | | | 0 | | | 11 | | 3 | | 7 | | 1 | | 3 | | 3 | 0 |
| kb743471.1_15457_star | kb743471.1_15457_star | | gccgtaaaaagctgccccggcggc | | | 0 | | | 1 | | 0 | | 1 | | 0 | | 0 | | 4 | 0 |
| kb742969.1_1017_star | kb742969.1_1017_star | | ctaagtttaagatgccatttttc | | | 0 | | | 0 | | 3 | | 0 | | 0 | | 0 | | 0 | 0 |
| kb742734.1_7067_star | kb742734.1_7067_star | | ggtggtttggtctcgggag | | | 0 | | | 1 | | 0 | | 0 | | 1 | | 0 | | 0 | 0 |
| kb744461.1_20389_star | kb744461.1_20389_star | | tgtgcacaaaggcccccgcttgctaca | | | 0 | | | 0 | | 0 | | 1 | | 0 | | 0 | | 0 | 0 |
| kb744634.1_20170_mature | kb744634.1_20170_mature | | aggtcttggagaattaca | | | 0 | | | 4 | | 0 | | 0 | | 0 | | 0 | | 2 | 0 |
| kb743829.1_10857_mature | kb743829.1_10857_mature | | acaccaaggcgggctgagcg | | | 0 | | | 0 | | 2 | | 3 | | 0 | | 0 | | 1 | 0 |
| kb742931.1_975_mature | kb742931.1_975_mature | | aagggaagagctctgcccaact | | | 4 | | | 9 | | 0 | | 11 | | 4 | | 3 | | 1 | 2 |
| kb742401.1_19759_star | kb742401.1_19759_star | | actctgttggtgtggcgtcaccttg | | | 2 | | | 2 | | 0 | | 1 | | 0 | | 1 | | 0 | 0 |
| kb743121.1_14795_star | kb743121.1_14795_star | | gagatacaggtgtttggcacaagt | | | 1 | | | 4 | | 2 | | 0 | | 0 | | 0 | | 3 | 0 |
| kb742432.1_487_mature | kb742432.1_487_mature | | tctggtccaacccctctg | | | 5 | | | 5 | | 2 | | 17 | | 10 | | 2 | | 3 | 3 |
| kb744927.1_18238_star | kb744927.1_18238_star | | ctttggtggcagtgtttgat | | | 1 | | | 1 | | 3 | | 0 | | 0 | | 0 | | 0 | 0 |
| kb742439.1_3147_mature | kb742439.1_3147_mature | | ttgtgcactgtttagaga | | | 0 | | | 1 | | 5 | | 0 | | 0 | | 0 | | 1 | 0 |
| kb742685.1_1574_mature | kb742685.1_1574_mature | | agatgtgtgtgggacacggact | | | 0 | | | 1 | | 1 | | 0 | | 2 | | 0 | | 4 | 0 |
| kb742544.1_17748_mature | kb742544.1_17748_mature | | tgctgtatgttcacactcatt | | | 0 | | | 2 | | 0 | | 4 | | 0 | | 0 | | 2 | 0 |
| kb743531.1_19939_mature | kb743531.1_19939_mature | | agacgtgcctgtagtacgacg | | | 0 | | | 1 | | 1 | | 0 | | 1 | | 1 | | 6 | 0 |
| kb742794.1_18829_mature | kb742794.1_18829_mature | | tggactgggagcctactgcttcc | | | 1 | | | 3 | | 0 | | 0 | | 4 | | 4 | | 0 | 1 |
| kb744212.1_8552_mature | kb744212.1_8552_mature | | tacattgcatgaggatgaggacg | | | 1 | | | 1 | | 3 | | 4 | | 0 | | 1 | | 2 | 0 |
| kb742899.1_7111_mature | kb742899.1_7111_mature | | aagactggggctgctgtcc | | | 1 | | | 3 | | 5 | | 1 | | 1 | | 0 | | 7 | 2 |
| kb743475.1_18384_star | kb743475.1_18384_star | | gtggagaaagaaagaagataatta | | | 0 | | | 4 | | 1 | | 1 | | 1 | | 1 | | 2 | 2 |
| kb743783.1_22087_star | kb743783.1_22087_star | | ttacagacctttggcacagagac | | | 2 | | | 1 | | 1 | | 1 | | 0 | | 1 | | 1 | 0 |
| kb742985.1_12952_mature | kb742985.1_12952_mature | | accggatagcgagcagggct | | | 0 | | | 5 | | 1 | | 4 | | 0 | | 1 | | 0 | 5 |
| kb742771.1_18565_mature | kb742771.1_18565_mature | | tcccagcctgtagctct | | | 28 | | | 91 | | 0 | | 143 | | 39 | | 2 | | 3 | 7 |
| kb742444.1_8011_star | kb742444.1_8011_star | | aaggtcatagctaaagacaaacaga | | | 0 | | | 0 | | 0 | | 5 | | 1 | | 0 | | 1 | 0 |
| kb742496.1_9558_mature | kb742496.1_9558_mature | | tggttggctctgagcttggagac | | | 2 | | | 8 | | 8 | | 14 | | 2 | | 2 | | 1 | 4 |
| kb742685.1_1599_star | kb742685.1_1599_star | | catgtcagggtattggg | | | 0 | | | 1 | | 0 | | 0 | | 0 | | 0 | | 1 | 0 |
| kb742539.1_3291_star | kb742539.1_3291_star | | ttctggtggacaaaaagc | | | 0 | | | 4 | | 0 | | 2 | | 2 | | 0 | | 2 | 0 |
| kb742685.1_1574_star | kb742685.1_1574_star | | tccgggttctccacaccttgct | | | 1 | | | 5 | | 2 | | 0 | | 0 | | 0 | | 0 | 1 |
| kb742967.1_10123_mature | kb742967.1_10123_mature | | aatgaactggctgctcttt | | | 0 | | | 4 | | 7 | | 0 | | 3 | | 1 | | 5 | 0 |
| kb742802.1_7867_star | kb742802.1_7867_star | | tgacaatcaaacagctttctttttact | | | 0 | | | 2 | | 1 | | 2 | | 1 | | 2 | | 3 | 1 |
| kb743110.1_6038_mature | kb743110.1_6038_mature | | cttgcggctgtctgaccag | | | 1 | | | 0 | | 2 | | 0 | | 2 | | 0 | | 2 | 2 |
| kb742808.1_102_mature | kb742808.1_102_mature | | tagatgactttgaaagccc | | | 0 | | | 0 | | 0 | | 4 | | 2 | | 0 | | 7 | 2 |
| kb745110.1_19011_star | kb745110.1_19011_star | | cagaatgatttttagttttgta | | | 0 | | | 0 | | 2 | | 1 | | 0 | | 0 | | 2 | 1 |
| kb742963.1_10700_mature | kb742963.1_10700_mature | | aagaaggacatcgaggtgcttgagc | | | 17 | | | 181 | | 2 | | 136 | | 17 | | 2 | | 11 | 4 |
| kb743024.1_15400_mature | kb743024.1_15400_mature | | catcagtaacaatgaata | | | 3 | | | 1 | | 0 | | 0 | | 0 | | 1 | | 1 | 0 |
| kb743733.1_16907_mature | kb743733.1_16907_mature | | atcccaccgctgccagca | | | 296 | | | 764 | | 998 | | 373 | | 324 | | 144 | | 419 | 165 |
| kb742701.1_6480_mature | kb742701.1_6480_mature | | tctcgatggacacagctcctgt | | | 1 | | | 3 | | 1 | | 3 | | 3 | | 0 | | 6 | 1 |
| kb742845.1_5775_mature | kb742845.1_5775_mature | | agtgtggtattttgatgc | | | 1 | | | 9 | | 20 | | 5 | | 5 | | 0 | | 15 | 3 |
| kb742652.1_6522_mature | kb742652.1_6522_mature | | tagactgctttctctgg | | | 1 | | | 79 | | 0 | | 22 | | 2 | | 0 | | 1 | 0 |
| kb743589.1_20878_mature | kb743589.1_20878_mature | | gtggctgtaggagatgg | | | 0 | | | 2 | | 1 | | 1 | | 1 | | 0 | | 2 | 1 |
| kb742800.1_13139_mature | kb742800.1_13139_mature | | agccaaccctgtaacttg | | | 7 | | | 0 | | 1 | | 2 | | 1 | | 1 | | 20 | 1 |
| kb744435.1_16060_star | kb744435.1_16060_star | | gtgttgcttcagcccctc | | | 1 | | | 4 | | 0 | | 9 | | 0 | | 1 | | 0 | 0 |
| kb743645.1_18018_mature | kb743645.1_18018_mature | | gccgagactagagtcacatct | | | 162 | | | 30 | | 98 | | 77 | | 37 | | 13 | | 186 | 25 |
| kb742815.1_7230_star | kb742815.1_7230_star | | tctgctgggctgtaggtgttca | | | 3 | | | 2 | | 5 | | 1 | | 1 | | 4 | | 3 | 2 |
| kb743192.1_11618_mature | kb743192.1_11618_mature | | ttgtgtgtgtgctttttct | | | 7 | | | 3 | | 4 | | 2 | | 3 | | 0 | | 6 | 0 |
| kb742497.1_4716_mature | kb742497.1_4716_mature | | aatgaaatagaaatgaat | | | 0 | | | 1 | | 2 | | 0 | | 0 | | 0 | | 5 | 1 |
| kb742627.1_5356_mature | kb742627.1_5356_mature | | agtgtgactgagagccccttgggtg | | | 0 | | | 8 | | 4 | | 3 | | 6 | | 1 | | 8 | 0 |
| kb742544.1_17751_mature | kb742544.1_17751_mature | | aagtcagtagatattcgca | | | 0 | | | 1 | | 0 | | 0 | | 0 | | 2 | | 3 | 0 |
| kb743109.1_14543_mature | kb743109.1_14543_mature | | aggaggtgtcagaaaag | | | 2 | | | 1 | | 0 | | 6 | | 0 | | 0 | | 1 | 2 |
| kb743217.1_9779_star | kb743217.1_9779_star | | ttggagggtatatgacactgggcag | | | 0 | | | 1 | | 0 | | 0 | | 0 | | 0 | | 3 | 0 |
| kb744074.1_11226_mature | kb744074.1_11226_mature | | agcaaggggctccggatc | | | 0 | | | 0 | | 0 | | 4 | | 0 | | 0 | | 0 | 0 |
| kb742736.1_11994_star | kb742736.1_11994_star | | tttttttctttgcattg | | | 1 | | | 0 | | 0 | | 3 | | 0 | | 0 | | 0 | 0 |
| kb742750.1_2175_star | kb742750.1_2175_star | | ctgcggtggcacatggcattg | | | 1 | | | 0 | | 0 | | 0 | | 0 | | 1 | | 1 | 0 |
| kb744198.1_12212_star | kb744198.1_12212_star | | tgtaaaagagtttagagggaacaaag | | | 2 | | | 5 | | 1 | | 5 | | 5 | | 1 | | 1 | 2 |
| kb744385.1_24162_star | kb744385.1_24162_star | | tgagagcatcgtgaggggggacc | | | 1 | | | 1 | | 0 | | 1 | | 10 | | 0 | | 3 | 29 |
| kb743589.1_20878_star | kb743589.1_20878_star | | ctttcctaccctctg | | | 0 | | | 0 | | 0 | | 0 | | 0 | | 1 | | 0 | 0 |
| kb742757.1_11056_star | kb742757.1_11056_star | | tcagctgccagctccacgtcc | | | 1 | | | 3 | | 2 | | 3 | | 1 | | 2 | | 2 | 3 |
| kb742887.1_1484_mature | kb742887.1_1484_mature | | ctctgtacttgtagctga | | | 3 | | | 38 | | 5 | | 18 | | 5 | | 3 | | 3 | 2 |
| kb744900.1_18360_star | kb744900.1_18360_star | | tcacagtcttgcacagtcaaca | | | 2 | | | 3 | | 7 | | 1 | | 4 | | 2 | | 7 | 0 |
| kb743275.1_2654_mature | kb743275.1_2654_mature | | atagctggcttgaagttactg | | | 3 | | | 0 | | 0 | | 0 | | 0 | | 0 | | 1 | 1 |
| kb748900.1_25258_star | kb748900.1_25258_star | | accatcggctgcctcgtcaccgac | | | 0 | | | 0 | | 2 | | 0 | | 0 | | 2 | | 0 | 4 |
| kb742770.1_17488_star | kb742770.1_17488_star | | tccagacacaattcttggttc | | | 1 | | | 1 | | 2 | | 0 | | 1 | | 2 | | 0 | 0 |
| kb743525.1_10189_mature | kb743525.1_10189_mature | | tttccttctgtcactgta | | | 2 | | | 13 | | 1 | | 18 | | 2 | | 0 | | 2 | 0 |
| kb743197.1_1098_mature | kb743197.1_1098_mature | | ccggcagaggagacgtgt | | | 0 | | | 3 | | 0 | | 1 | | 2 | | 3 | | 1 | 0 |
| kb743644.1_3285_star | kb743644.1_3285_star | | ttattttgagcttgaactgaatctgaa | | | 5 | | | 18 | | 5 | | 8 | | 5 | | 5 | | 11 | 0 |
| kb745353.1_23446_star | kb745353.1_23446_star | | tttggagtatgtgattgcaca | | | 1 | | | 5 | | 0 | | 2 | | 1 | | 2 | | 1 | 1 |
| kb742963.1_10701_star | kb742963.1_10701_star | | tcttggggtttacaaagttcacata | | | 1 | | | 2 | | 1 | | 14 | | 1 | | 0 | | 2 | 0 |
| kb742845.1_5868_star | kb742845.1_5868_star | | agttcactaacatgttgca | | | 0 | | | 1 | | 0 | | 0 | | 0 | | 0 | | 0 | 0 |
| kb742696.1_10419_star | kb742696.1_10419_star | | aacgagtcctggtctggttgtaga | | | 0 | | | 3 | | 1 | | 4 | | 4 | | 1 | | 2 | 1 |
| kb785238.1_25837_mature | kb785238.1_25837_mature | | tgtccccttcctgctgcccaga | | | 0 | | | 3 | | 5 | | 2 | | 1 | | 0 | | 1 | 2 |
| kb744722.1_10780_mature | kb744722.1_10780_mature | | aaactgtagggctacggagttgc | | | 3 | | | 4 | | 4 | | 4 | | 1 | | 0 | | 1 | 3 |
| kb743311.1_18864_star | kb743311.1_18864_star | | gctgggacggtgaagggaggc | | | 0 | | | 2 | | 3 | | 3 | | 1 | | 1 | | 3 | 0 |
| kb743145.1_853_mature | kb743145.1_853_mature | | tatttagttgcgtggcactga | | | 3 | | | 0 | | 2 | | 0 | | 0 | | 0 | | 0 | 0 |
| kb743240.1_19701_star | kb743240.1_19701_star | | taaagattaacttttgtcctgtg | | | 0 | | | 1 | | 0 | | 2 | | 0 | | 0 | | 3 | 0 |
| kb742773.1_1227_star | kb742773.1_1227_star | | tgcagaacttggcatt | | | 0 | | | 0 | | 0 | | 0 | | 0 | | 1 | | 1 | 0 |
| kb743381.1_20038_mature | kb743381.1_20038_mature | | cagggacgttgtcaggttaacact | | | 3 | | | 6 | | 10 | | 4 | | 1 | | 0 | | 3 | 2 |
| kb743532.1_9203_star | kb743532.1_9203_star | | gccgtggagaggtgctaggatgga | | | 0 | | | 1 | | 1 | | 0 | | 1 | | 2 | | 1 | 0 |
| kb743739.1_14645_mature | kb743739.1_14645_mature | | cactggatgaatttagagtaat | | | 3 | | | 0 | | 4 | | 3 | | 1 | | 0 | | 0 | 0 |
| kb742970.1_9483_mature | kb742970.1_9483_mature | | gtgttgatatggaggaccttt | | | 0 | | | 3 | | 3 | | 0 | | 0 | | 0 | | 3 | 0 |
| kb742777.1_8157_mature | kb742777.1_8157_mature | | ttcaaatgaagaaaagcgccc | | | 0 | | | 1 | | 0 | | 4 | | 0 | | 0 | | 4 | 0 |
| kb743235.1_6577_mature | kb743235.1_6577_mature | | ttctgaagagcttaaaaac | | | 18 | | | 160 | | 4 | | 162 | | 33 | | 0 | | 13 | 3 |
| kb742933.1_16263_star | kb742933.1_16263_star | | agcctgattcagcatttttgcac | | | 0 | | | 1 | | 2 | | 1 | | 0 | | 1 | | 6 | 0 |
| kb744204.1_11858_star | kb744204.1_11858_star | | tgagtgtagtaggaactgaat | | | 0 | | | 2 | | 2 | | 3 | | 1 | | 0 | | 0 | 0 |
| kb745306.1_22118_mature | kb745306.1_22118_mature | | taggatctgatagcgaac | | | 0 | | | 2 | | 0 | | 2 | | 1 | | 0 | | 3 | 0 |
| kb742856.1_19615_star | kb742856.1_19615_star | | atacattagaagaacaaag | | | 0 | | | 2 | | 0 | | 0 | | 0 | | 0 | | 0 | 0 |
| kb742833.1_90_star | kb742833.1_90_star | | aattttgtttcaggtcatagt | | | 1 | | | 1 | | 0 | | 1 | | 0 | | 0 | | 1 | 0 |
| kb744105.1_20145_mature | kb744105.1_20145_mature | | tgaggatgagggcttaaattccaca | | | 0 | | | 0 | | 3 | | 2 | | 0 | | 0 | | 3 | 0 |
| kb742668.1_5585_star | kb742668.1_5585_star | | tagtttttggaagaaacatgca | | | 0 | | | 4 | | 4 | | 1 | | 0 | | 1 | | 7 | 0 |
| kb743297.1_21458_star | kb743297.1_21458_star | | acccaatcaacagccaacaggca | | | 1 | | | 0 | | 5 | | 1 | | 1 | | 2 | | 2 | 1 |
| kb743948.1_17919_mature | kb743948.1_17919_mature | | aggcttgagagaaattcttca | | | 0 | | | 4 | | 2 | | 0 | | 0 | | 0 | | 3 | 0 |
| kb742531.1_10736_mature | kb742531.1_10736_mature | | ctggctggagcttggggc | | | 1 | | | 0 | | 1 | | 1 | | 0 | | 0 | | 1 | 4 |
| kb743177.1_6598_star | kb743177.1_6598_star | | tacttcttcatgaacaaat | | | 0 | | | 1 | | 0 | | 0 | | 0 | | 1 | | 1 | 2 |
| kb742486.1_15041_mature | kb742486.1_15041_mature | | atgcttgtctttttgctgga | | | 1 | | | 3 | | 0 | | 7 | | 4 | | 1 | | 3 | 0 |
| kb744389.1_22416_mature | kb744389.1_22416_mature | | gactgctgatagatgat | | | 0 | | | 0 | | 0 | | 4 | | 19 | | 0 | | 2 | 0 |
| kb746371.1_24769_star | kb746371.1_24769_star | | cttctttgcttcagtcttcact | | | 0 | | | 10 | | 8 | | 17 | | 7 | | 4 | | 5 | 1 |
| kb743217.1_9778_mature | kb743217.1_9778_mature | | gagtatgtgttagaactgtg | | | | 2 | | 1 | | 2 | | 0 | | 0 | | 0 | | 4 | 0 |
| kb742745.1_15186_mature | kb742745.1_15186_mature | | actgggcaccactgggaacagc | | | | 2 | | 5 | | 5 | | 4 | | 11 | | 4 | | 2 | 4 |
| kb742722.1_13456_star | kb742722.1_13456_star | | gatttcagtgaaatgaagcccg | | | | 0 | | 0 | | 1 | | 1 | | 0 | | 0 | | 0 | 0 |
| kb743356.1_12127_mature | kb743356.1_12127_mature | | tacgggaagaatgtactgatt | | | | 4 | | 1 | | 8 | | 0 | | 5 | | 5 | | 1 | 0 |
| kb743058.1_6258_mature | kb743058.1_6258_mature | | attgtgactcatctttctgtac | | | | 4 | | 1 | | 1 | | 5 | | 1 | | 0 | | 3 | 0 |
| kb743381.1_20038_star | kb743381.1_20038_star | | ggtaagccttgctatgcactgag | | | | 0 | | 0 | | 1 | | 0 | | 1 | | 0 | | 0 | 0 |
| kb742772.1_18989_star | kb742772.1_18989_star | | tcaatcttccggctgcaat | | | | 0 | | 0 | | 1 | | 1 | | 0 | | 1 | | 0 | 1 |
| kb742595.1_8379_mature | kb742595.1_8379_mature | | tttactctttctggttagac | | | | 0 | | 0 | | 0 | | 3 | | 0 | | 1 | | 0 | 0 |
| kb743048.1_8305_star | kb743048.1_8305_star | | aaatgagaaaagggatcagtg | | | | 1 | | 0 | | 0 | | 0 | | 0 | | 0 | | 1 | 0 |
| kb742685.1_1622_star | kb742685.1_1622_star | | gattatgtgtctgaaat | | | | 0 | | 0 | | 0 | | 0 | | 0 | | 0 | | 2 | 0 |
| kb742386.1_9876_mature | kb742386.1_9876_mature | | tatccaaattctcgtctctgggcac | | | | 0 | | 0 | | 6 | | 0 | | 1 | | 1 | | 2 | 0 |
| kb743381.1_20048_star | kb743381.1_20048_star | | ttatttcaggctcacttgc | | | | 0 | | 0 | | 0 | | 0 | | 1 | | 0 | | 1 | 0 |
| kb742798.1_12572_mature | kb742798.1_12572_mature | | cccctgtttctgtagagc | | | | 0 | | 14 | | 3 | | 4 | | 1 | | 5 | | 4 | 0 |
| kb742855.1_12687_star | kb742855.1_12687_star | | agtgctcctgttgtggactctca | | | | 0 | | 0 | | 1 | | 3 | | 1 | | 0 | | 2 | 0 |
| kb743897.1_19283_star | kb743897.1_19283_star | | gcgcaggagctgcatgtag | | | | 0 | | 0 | | 0 | | 1 | | 0 | | 0 | | 0 | 1 |
| kb743203.1_21095_mature | kb743203.1_21095_mature | | tcatggaaatgtgtgcttt | | | | 3 | | 10 | | 4 | | 3 | | 1 | | 6 | | 5 | 0 |
| kb743366.1_17984_star | kb743366.1_17984_star | | aaatattccagtagcttt | | | | 0 | | 1 | | 1 | | 1 | | 0 | | 0 | | 1 | 0 |
| kb742441.1_711_star | kb742441.1_711_star | | taaacagctgaggaatgtgtattgctgt | | | | 2 | | 2 | | 2 | | 0 | | 1 | | 2 | | 7 | 0 |
| kb742745.1_15181_star | kb742745.1_15181_star | | actcgtttctgcttcactaaaat | | | | 1 | | 1 | | 1 | | 0 | | 0 | | 1 | | 1 | 0 |
| kb743789.1_20582_star | kb743789.1_20582_star | | tcattaggaatcttaacaga | | | | 0 | | 0 | | 0 | | 1 | | 0 | | 0 | | 1 | 0 |
| kb744020.1_10268_star | kb744020.1_10268_star | | tgtgaagttggtgcttgaggaggtaacg | | | | 0 | | 3 | | 0 | | 2 | | 0 | | 2 | | 3 | 0 |
| kb742479.1_264_star | kb742479.1_264_star | | tttttaaatattgtaatt | | | | 1 | | 0 | | 1 | | 2 | | 0 | | 0 | | 2 | 0 |
| kb742629.1_525_mature | kb742629.1_525_mature | | cctgacaatgactgtaaaact | | | | 1 | | 9 | | 1 | | 3 | | 0 | | 0 | | 5 | 1 |
| kb742833.1_90_mature | kb742833.1_90_mature | | aacgatcaagagcaaaaactct | | | | 1 | | 3 | | 0 | | 4 | | 1 | | 0 | | 3 | 1 |
| kb743612.1_12155_star | kb743612.1_12155_star | | ggccttcagacgccgatctctaaa | | | | 0 | | 2 | | 0 | | 0 | | 0 | | 3 | | 2 | 0 |
| kb745974.1_23663_mature | kb745974.1_23663_mature | | gggggggccgggccgggc | | | | 5 | | 7 | | 10 | | 5 | | 3 | | 3 | | 4 | 12 |
| kb746876.1_23003_star | kb746876.1_23003_star | | ggccgtgagctcccctcatcccagcc | | | | 2 | | 1 | | 0 | | 3 | | 0 | | 0 | | 1 | 0 |
| kb745179.1_20929_mature | kb745179.1_20929_mature | | ttggatagtcttctggca | | | | 0 | | 4 | | 0 | | 0 | | 1 | | 1 | | 1 | 0 |
| kb742947.1_20580_mature | kb742947.1_20580_mature | | ccacgtgaggacgggtgctgt | | | | 2 | | 2 | | 2 | | 0 | | 0 | | 0 | | 7 | 1 |
| kb742504.1_8579_mature | kb742504.1_8579_mature | | ttcggacaaaaatggaacg | | | | 0 | | 0 | | 3 | | 0 | | 0 | | 0 | | 2 | 0 |
| kb743257.1_4665_star | kb743257.1_4665_star | | cactcagtgactgaaaatgc | | | | 2 | | 3 | | 2 | | 0 | | 0 | | 4 | | 1 | 1 |
| kb742382.1_14244_star | kb742382.1_14244_star | | acagagggagatataaaacca | | | | 0 | | 0 | | 2 | | 0 | | 4 | | 0 | | 0 | 1 |
| kb742459.1_3529_star | kb742459.1_3529_star | | tctcttcctggagcttgagcac | | | | 0 | | 12 | | 1 | | 0 | | 0 | | 0 | | 1 | 1 |
| kb743374.1_21334_mature | kb743374.1_21334_mature | | acctcgagtactgtgtgc | | | | 11 | | 130 | | 4 | | 86 | | 21 | | 2 | | 7 | 5 |
| kb744171.1_23782_mature | kb744171.1_23782_mature | | tagtgactggcctccaact | | | | 108 | | 690 | | 14 | | 794 | | 152 | | 9 | | 28 | 17 |
| kb742730.1_8115_star | kb742730.1_8115_star | | ctcatcctccgcc | | | | 0 | | 0 | | 1 | | 0 | | 0 | | 0 | | 1 | 0 |
| kb744454.1_19867_mature | kb744454.1_19867_mature | | tagatgagctcagatttaa | | | | 0 | | 4 | | 5 | | 3 | | 0 | | 0 | | 2 | 0 |
| kb743137.1_10071_star | kb743137.1_10071_star | | cttgcaaatttcagtt | | | | 0 | | 0 | | 0 | | 1 | | 0 | | 0 | | 2 | 0 |
| kb742430.1_11435_mature | kb742430.1_11435_mature | | agtgaagtctgaagaggga | | | | 0 | | 2 | | 5 | | 2 | | 1 | | 3 | | 2 | 0 |
| kb744429.1_15133_mature | kb744429.1_15133_mature | | tggctggaactgagctg | | | | 0 | | 3 | | 1 | | 1 | | 2 | | 0 | | 1 | 0 |
| kb742984.1_1913_star | kb742984.1_1913_star | | cctttcacttttccctgaaatg | | | | 1 | | 1 | | 0 | | 1 | | 0 | | 0 | | 4 | 1 |
| kb742444.1_7983_mature | kb742444.1_7983_mature | | acctcaagtactgtgtgt | | | | 1 | | 4 | | 0 | | 3 | | 1 | | 1 | | 0 | 0 |
| kb742497.1_4727_star | kb742497.1_4727_star | | agtttctgtttagatatttacac | | | | 0 | | 0 | | 0 | | 1 | | 0 | | 1 | | 1 | 0 |
| kb743564.1_13643_mature | kb743564.1_13643_mature | | attgtcctctgctgatgctggt | | | | 0 | | 6 | | 2 | | 3 | | 1 | | 1 | | 4 | 0 |
| kb742745.1_15171_star | kb742745.1_15171_star | | gatgtgaagttctgctccta | | | | 0 | | 3 | | 2 | | 0 | | 3 | | 1 | | 4 | 0 |
| kb744699.1_17461_mature | kb744699.1_17461_mature | | cttatgtggcggatggttcgc | | | | 0 | | 0 | | 0 | | 0 | | 1 | | 0 | | 0 | 3 |
| kb743973.1_15351_star | kb743973.1_15351_star | | tgctctgttttaaccatc | | | | 0 | | 0 | | 0 | | 0 | | 0 | | 1 | | 1 | 0 |
| kb742402.1_7669_mature | kb742402.1_7669_mature | | cttttggtattttgaacaaa | | | | 0 | | 2 | | 0 | | 5 | | 0 | | 2 | | 6 | 3 |
| kb742418.1_2465_star | kb742418.1_2465_star | | tcaaggtgcagaag | | | | 0 | | 0 | | 1 | | 0 | | 0 | | 0 | | 0 | 0 |
| kb742652.1_6556_star | kb742652.1_6556_star | | tgtgtgatggtggggcacatgcgt | | | | 0 | | 0 | | 0 | | 1 | | 2 | | 0 | | 1 | 0 |
| kb743809.1_17044_mature | kb743809.1_17044_mature | | ccctgagatgtagacatg | | | | 1 | | 1 | | 0 | | 13 | | 1 | | 1 | | 0 | 0 |
| kb744073.1_20230_mature | kb744073.1_20230_mature | | tcaagaagtgtttggacggtgctct | | | | 20 | | 1215 | | 12 | | 1106 | | 74 | | 7 | | 38 | 10 |
| kb743580.1_14137_mature | kb743580.1_14137_mature | | aggtgtgtgaattcagtac | | | | 0 | | 0 | | 2 | | 1 | | 0 | | 0 | | 4 | 1 |
| kb743608.1_9442_star | kb743608.1_9442_star | | cgtggaaggaaagactgaact | | | | 2 | | 2 | | 1 | | 2 | | 0 | | 1 | | 2 | 3 |
| kb742671.1_7172_mature | kb742671.1_7172_mature | | caagggctaggatgactccc | | | | 19 | | 13 | | 19 | | 8 | | 6 | | 3 | | 21 | 15 |
| kb744185.1_18095_star | kb744185.1_18095_star | | ccaagctaccagggcaacta | | | | 1 | | 3 | | 0 | | 0 | | 1 | | 0 | | 0 | 0 |
| kb742435.1_5941_star | kb742435.1_5941_star | | tcactggtatatactacagatt | | | | 0 | | 0 | | 0 | | 0 | | 0 | | 1 | | 0 | 0 |
| kb743649.1_15515_mature | kb743649.1_15515_mature | | gtgattgccaggtactgaca | | | | 0 | | 2 | | 0 | | 0 | | 0 | | 6 | | 9 | 1 |
| kb742970.1_9483_star | kb742970.1_9483_star | | gggtcccagaggcagcgctg | | | | 0 | | 2 | | 3 | | 0 | | 0 | | 0 | | 1 | 0 |
| kb785238.1_25837_star | kb785238.1_25837_star | | aggtgacagcggcggggtgacagg | | | | 1 | | 5 | | 2 | | 0 | | 1 | | 0 | | 0 | 2 |
| kb742711.1_5023_mature | kb742711.1_5023_mature | | atgtgagtgagctctgtcc | | | | 2 | | 2 | | 4 | | 1 | | 0 | | 1 | | 4 | 2 |
| kb744588.1_17428_mature | kb744588.1_17428_mature | | ttggtaagtgtacttggaatagc | | | | 1 | | 3 | | 4 | | 5 | | 1 | | 0 | | 3 | 0 |
| kb742815.1_7230_mature | kb742815.1_7230_mature | | aagtgcctgacagactgaga | | | | 1 | | 5 | | 1 | | 3 | | 0 | | 0 | | 3 | 1 |
| kb763423.1_26018_mature | kb763423.1_26018_mature | | aggggccggggctggggc | | | | 2 | | 2 | | 8 | | 5 | | 15 | | 2 | | 6 | 166 |
| kb743278.1_18082_star | kb743278.1_18082_star | | gcagagagaggccccccccagc | | | | 1 | | 0 | | 2 | | 0 | | 0 | | 0 | | 0 | 0 |
| kb745863.1_24310_star | kb745863.1_24310_star | | gatcctgccctggggatgggct | | | | 1 | | 0 | | 0 | | 1 | | 1 | | 2 | | 0 | 1 |
| kb747149.1_25441_star | kb747149.1_25441_star | | ataggtctaggccttgtc | | | | 0 | | 0 | | 1 | | 0 | | 0 | | 0 | | 0 | 0 |
| kb742808.1_102_star | kb742808.1_102_star | | gctggctaagtatcttcatttcct | | | | 0 | | 1 | | 0 | | 2 | | 0 | | 2 | | 0 | 0 |
| kb744353.1_15001_mature | kb744353.1_15001_mature | | ttttgtgagagtaaggactgca | | | | 1 | | 6 | | 8 | | 2 | | 0 | | 3 | | 2 | 4 |
| kb752032.1_25714_mature | kb752032.1_25714_mature | | gtgggtgggagcggctggg | | | | 1 | | 4 | | 2 | | 3 | | 0 | | 0 | | 1 | 3 |
| kb744477.1_11827_mature | kb744477.1_11827_mature | | atttatctgagcactgaca | | | | 2 | | 0 | | 0 | | 2 | | 0 | | 0 | | 2 | 3 |
| kb742931.1_976_star | kb742931.1_976_star | | gagggcagagcactcctctcct | | | | 1 | | 3 | | 2 | | 0 | | 3 | | 2 | | 1 | 4 |
| kb744353.1_15005_mature | kb744353.1_15005_mature | | actgagatcgctggggtgctc | | | | 1 | | 3 | | 1 | | 3 | | 0 | | 0 | | 0 | 1 |
| kb742496.1_9564_mature | kb742496.1_9564_mature | | tttcctgtacatgtaact | | | | 0 | | 0 | | 0 | | 0 | | 0 | | 4 | | 0 | 0 |
| kb744720.1_9047_mature | kb744720.1_9047_mature | | tggatgtctgaattcagg | | | | 1 | | 2 | | 4 | | 0 | | 2 | | 0 | | 5 | 0 |
| kb743162.1_14321_mature | kb743162.1_14321_mature | | tctttaattcatttctgtca | | | | 0 | | 2 | | 0 | | 0 | | 0 | | 0 | | 3 | 0 |
| kb742931.1_959_star | kb742931.1_959_star | | ggtatttttagcccagatcatt | | | | 0 | | 0 | | 2 | | 0 | | 3 | | 0 | | 4 | 0 |
| kb743162.1_14321_star | kb743162.1_14321_star | | aggaaggatagtaaacacagatt | | | | 1 | | 1 | | 1 | | 0 | | 0 | | 1 | | 0 | 0 |
| kb742770.1_17490_star | kb742770.1_17490_star | | tggggcaggagagactccctgc | | | | 0 | | 2 | | 1 | | 0 | | 1 | | 2 | | 0 | 0 |
| kb742811.1_13111_star | kb742811.1_13111_star | | atgcatcggctgtgccagccactt | | | | 1 | | 1 | | 2 | | 5 | | 2 | | 0 | | 7 | 17 |
| kb743167.1_13294_star | kb743167.1_13294_star | | ttgttttccgctctcc | | | | 0 | | 0 | | 2 | | 0 | | 0 | | 0 | | 0 | 0 |
| kb742446.1_12973_mature | kb742446.1_12973_mature | | attgctctgatggctttggga | | | | 4 | | 17 | | 6 | | 8 | | 2 | | 9 | | 2 | 1 |
| kb742574.1_16076_mature | kb742574.1_16076_mature | | caggactgtggctagcacaaggg | | | | 1 | | 2 | | 6 | | 0 | | 0 | | 0 | | 4 | 2 |
| kb742527.1_3882_star | kb742527.1_3882_star | | atacatgttctttatcac | | | | 2 | | 0 | | 0 | | 1 | | 0 | | 1 | | 0 | 0 |
| kb742904.1_12934_mature | kb742904.1_12934_mature | | tgatgtgagactgacagg | | | | 43 | | 187 | | 3 | | 441 | | 33 | | 1 | | 17 | 12 |
| kb742970.1_9501_mature | kb742970.1_9501_mature | | aacgcggagcctcgcagatg | | | | 1 | | 2 | | 3 | | 1 | | 0 | | 0 | | 0 | 0 |
| kb742444.1_7990_star | kb742444.1_7990_star | | gagcatgttgcaggatcacagcaagg | | | | 1 | | 9 | | 1 | | 6 | | 8 | | 1 | | 3 | 0 |
| kb744306.1_3751_mature | kb744306.1_3751_mature | | ttttgatggattttgataaaaagaa | | | | 1 | | 13 | | 0 | | 5 | | 4 | | 4 | | 21 | 0 |
| kb743214.1_8712_mature | kb743214.1_8712_mature | | aaaagcatggcaacccca | | | | 17 | | 5 | | 13 | | 23 | | 6 | | 0 | | 33 | 2 |
| kb742848.1_6910_star | kb742848.1_6910_star | | tcttcaaaggtcacatggt | | | | 0 | | 2 | | 0 | | 1 | | 2 | | 0 | | 4 | 0 |
| kb743532.1_9209_star | kb743532.1_9209_star | | atttgttcattgaaggcaattgctc | | | | 0 | | 1 | | 0 | | 0 | | 0 | | 1 | | 0 | 0 |
| kb746371.1_24769_mature | kb746371.1_24769_mature | | tgacggcagatacagagaaggt | | | | 2 | | 16 | | 3 | | 6 | | 4 | | 1 | | 5 | 5 |
| kb744079.1_21184_mature | kb744079.1_21184_mature | | agaactgtcagggcatctgtc | | | | 0 | | 5 | | 3 | | 3 | | 1 | | 0 | | 3 | 1 |
| kb743062.1_19334_star | kb743062.1_19334_star | | ctttgactgcctctccagac | | | | 1 | | 3 | | 0 | | 2 | | 2 | | 0 | | 1 | 0 |
| kb742757.1_11033_star | kb742757.1_11033_star | | ctcatccctctctccaggt | | | | 0 | | 0 | | 0 | | 1 | | 1 | | 0 | | 1 | 1 |
| kb742734.1_7059_mature | kb742734.1_7059_mature | | ttgctgtgatgatggatttctgagc | | | | 19 | | 68 | | 43 | | 43 | | 28 | | 21 | | 35 | 13 |
| kb743686.1_3384_star | kb743686.1_3384_star | | ccagcagccgcagatgagaca | | | | 1 | | 2 | | 3 | | 1 | | 0 | | 1 | | 0 | 0 |
| kb744209.1_16235_star | kb744209.1_16235_star | | ctcgaacatggagcggttg | | | | 0 | | 0 | | 0 | | 3 | | 0 | | 0 | | 0 | 0 |
| kb742596.1_13305_star | kb742596.1_13305_star | | ctcccaagtccttctctgccaggaagc | | | | 5 | | 24 | | 3 | | 27 | | 1 | | 5 | | 6 | 4 |
| kb743090.1_9114_star | kb743090.1_9114_star | | ttaatgagtactcaaatttg | | | | 0 | | 0 | | 2 | | 1 | | 0 | | 0 | | 0 | 0 |
| kb742694.1_7959_star | kb742694.1_7959_star | | cacaagctatgcactgctatc | | | | 1 | | 3 | | 1 | | 0 | | 0 | | 0 | | 0 | 0 |
| kb743505.1_24117_star | kb743505.1_24117_star | | tcgtggggagggggacactgcc | | | | 1 | | 2 | | 0 | | 1 | | 0 | | 2 | | 3 | 3 |
| kb743105.1_19469_star | kb743105.1_19469_star | | gaggaacacagcttgctgaaga | | | | 1 | | 1 | | 3 | | 0 | | 1 | | 1 | | 0 | 1 |
| kb742404.1_2825_mature | kb742404.1_2825_mature | | aaagactgtcctggtaacc | | | | 5 | | 128 | | 4 | | 123 | | 33 | | 5 | | 15 | 3 |
| kb742995.1_20813_star | kb742995.1_20813_star | | tcttcagtcttttaaat | | | | 4 | | 4 | | 4 | | 2 | | 2 | | 0 | | 10 | 1 |
| kb751569.1_24640_star | kb751569.1_24640_star | | agcacaggtccctcagcc | | | | 1 | | 4 | | 2 | | 1 | | 0 | | 1 | | 0 | 1 |
| kb743387.1_22887_mature | kb743387.1_22887_mature | | cagagaggactggggacagc | | | | 0 | | 1 | | 3 | | 1 | | 5 | | 0 | | 2 | 4 |
| kb742928.1_10842_mature | kb742928.1_10842_mature | | ctgagctggaggaccaggacggt | | | | 7 | | 9 | | 7 | | 37 | | 5 | | 0 | | 3 | 5 |
| kb743487.1_17951_mature | kb743487.1_17951_mature | | tggcaacagaagggaac | | | | 5 | | 9 | | 0 | | 4 | | 5 | | 0 | | 1 | 1 |
| kb743977.1_12630_mature | kb743977.1_12630_mature | | aggctgtcagatttgtactatgac | | | | 1 | | 3 | | 3 | | 2 | | 5 | | 0 | | 5 | 0 |
| kb743228.1_14904_mature | kb743228.1_14904_mature | | attgttctgtaaacccttta | | | | 0 | | 0 | | 4 | | 1 | | 1 | | 0 | | 1 | 1 |
| kb743307.1_13925_star | kb743307.1_13925_star | | aggatgccgacttcgaca | | | | 0 | | 1 | | 0 | | 0 | | 0 | | 0 | | 0 | 0 |
| kb742899.1_7120_mature | kb742899.1_7120_mature | | tttgggcttagtgcacctgacg | | | | 1 | | 5 | | 1 | | 1 | | 0 | | 2 | | 1 | 5 |
| kb742997.1_15682_mature | kb742997.1_15682_mature | | ttctaggctgctctccagt | | | | 16 | | 185 | | 0 | | 202 | | 52 | | 11 | | 11 | 11 |
| kb742969.1_1047_star | kb742969.1_1047_star | | gtttagaggcaaactttagagtacttg | | | | 0 | | 1 | | 1 | | 0 | | 2 | | 0 | | 1 | 1 |
| kb743260.1_3350_star | kb743260.1_3350_star | | ctcataaatccgtctgtgcattgct | | | | 0 | | 2 | | 0 | | 1 | | 0 | | 2 | | 1 | 0 |
| kb742847.1_16291_star | kb742847.1_16291_star | | tccacttacctaggccac | | | | 0 | | 2 | | 1 | | 0 | | 0 | | 0 | | 0 | 0 |
| kb743145.1_772_star | kb743145.1_772_star | | ctaaactttatcttctcttctgggat | | | | 4 | | 10 | | 0 | | 5 | | 4 | | 1 | | 0 | 2 |
| kb743662.1_7544_mature | kb743662.1_7544_mature | | tttcatttctctgtacgtgtga | | | | 0 | | 1 | | 0 | | 0 | | 1 | | 4 | | 5 | 0 |
| kb743545.1_16427_star | kb743545.1_16427_star | | gggttgttttctcgcaggtgg | | | | 3 | | 1 | | 0 | | 9 | | 2 | | 1 | | 0 | 1 |
| kb742599.1_14531_mature | kb742599.1_14531_mature | | gcctgacggctgtgcggac | | | | 1 | | 6 | | 3 | | 2 | | 1 | | 2 | | 0 | 2 |
| kb742418.1_2464_mature | kb742418.1_2464_mature | | ccagagtgatgtggactgactgggc | | | | 1 | | 8 | | 10 | | 1 | | 8 | | 3 | | 6 | 7 |
| kb742503.1_10977_mature | kb742503.1_10977_mature | | taggtctggcctttatggac | | | | 0 | | 2 | | 1 | | 0 | | 0 | | 0 | | 1 | 1 |
| kb742811.1_13093_mature | kb742811.1_13093_mature | | ctggtgtggaagaagactg | | | | 3 | | 7 | | 3 | | 32 | | 2 | | 1 | | 8 | 3 |
| kb743176.1_3945_mature | kb743176.1_3945_mature | | tgaatgctgggctgtggtag | | | | 0 | | 3 | | 2 | | 0 | | 1 | | 1 | | 2 | 4 |
| kb742811.1_13093_star | kb742811.1_13093_star | | gtctgttcccacgtgcc | | | | 0 | | 1 | | 0 | | 0 | | 0 | | 1 | | 1 | 0 |
| kb742452.1_10600_star | kb742452.1_10600_star | | cagtaagtgccatcaggtc | | | | 0 | | 0 | | 0 | | 0 | | 1 | | 0 | | 1 | 0 |
| kb743175.1_22042_star | kb743175.1_22042_star | | cgacttccaggccctgg | | | | 0 | | 1 | | 0 | | 2 | | 1 | | 0 | | 0 | 0 |
| kb742997.1_15682_star | kb742997.1_15682_star | | tgcccagtctgtatgtatagc | | | | 27 | | 69 | | 26 | | 22 | | 17 | | 18 | | 28 | 22 |
| kb742714.1_4146_mature | kb742714.1_4146_mature | | tctaggtgacttaggggcgtgt | | | | 0 | | 1 | | 3 | | 0 | | 1 | | 1 | | 0 | 0 |
| kb746876.1_23003_mature | kb746876.1_23003_mature | | agatgatgagagctctgggtgc | | | | 7 | | 3 | | 3 | | 2 | | 2 | | 1 | | 4 | 3 |
| kb742414.1_13361_star | kb742414.1_13361_star | | gggcacagttcctccc | | | | 2 | | 0 | | 0 | | 0 | | 1 | | 0 | | 0 | 0 |
| kb744105.1_20113_mature | kb744105.1_20113_mature | | aattggcgtggtgcagttc | | | | 0 | | 5 | | 0 | | 0 | | 0 | | 0 | | 0 | 1 |
| kb742693.1_8764_star | kb742693.1_8764_star | | catctcaaaactctggacaa | | | | 0 | | 4 | | 3 | | 0 | | 0 | | 2 | | 2 | 1 |
| kb742694.1_7959_mature | kb742694.1_7959_mature | | atgctgtccatgctgctgac | | | | 0 | | 5 | | 0 | | 1 | | 0 | | 0 | | 1 | 1 |
| kb742465.1_20588_mature | kb742465.1_20588_mature | | tgggatgggcgtgggggc | | | | 1 | | 4 | | 2 | | 7 | | 0 | | 2 | | 1 | 2 |
| kb745208.1_24259_mature | kb745208.1_24259_mature | | cgcgacatgccagcattatgaca | | | | 0 | | 0 | | 0 | | 3 | | 0 | | 0 | | 0 | 0 |
| kb742490.1_1386_mature | kb742490.1_1386_mature | | tgcagaattactaggagcagatgt | | | | 0 | | 1 | | 2 | | 1 | | 3 | | 1 | | 2 | 0 |
| kb742651.1_5179_mature | kb742651.1_5179_mature | | cccaagcacatttgtccacc | | | | 3 | | 2 | | 0 | | 0 | | 2 | | 0 | | 1 | 0 |
| kb743197.1_1098_star | kb743197.1_1098_star | | acagcgcccagcaggaa | | | | 0 | | 2 | | 0 | | 1 | | 0 | | 0 | | 0 | 1 |
| kb744087.1_18315_mature | kb744087.1_18315_mature | | tgatcctgtaggagctttgc | | | | 3 | | 57 | | 2 | | 29 | | 34 | | 3 | | 5 | 0 |
| kb744909.1_18946_star | kb744909.1_18946_star | | tctgaaggctttattgtta | | | | 0 | | 0 | | 1 | | 0 | | 0 | | 0 | | 2 | 0 |
| kb742758.1_20730_star | kb742758.1_20730_star | | ttgtttaaactgatgagattttgg | | | | 0 | | 0 | | 3 | | 1 | | 0 | | 2 | | 0 | 0 |
| kb742637.1_18799_star | kb742637.1_18799_star | | agcgagctgcagcccctttc | | | | 1 | | 1 | | 5 | | 4 | | 3 | | 1 | | 3 | 2 |
| kb743526.1_18254_mature | kb743526.1_18254_mature | | gatttaatgaggctgat | | | | 0 | | 0 | | 0 | | 1 | | 1 | | 0 | | 0 | 0 |
| kb743939.1_22763_mature | kb743939.1_22763_mature | | agtaactatgactctct | | | | 38 | | 31 | | 57 | | 25 | | 15 | | 18 | | 29 | 18 |
| kb744353.1_15008_star | kb744353.1_15008_star | | gccccatggagcctccttccaca | | | | 1 | | 5 | | 0 | | 4 | | 0 | | 0 | | 1 | 0 |
| kb742786.1_17316_star | kb742786.1_17316_star | | tcaggaaggctgtttcaatgtt | | | | 3 | | 2 | | 1 | | 1 | | 0 | | 0 | | 3 | 0 |
| kb744088.1_3989_mature | kb744088.1_3989_mature | | aagaactgtgaaaagag | | | | 1 | | 8 | | 5 | | 4 | | 4 | | 1 | | 3 | 2 |
| kb742970.1_9501_star | kb742970.1_9501_star | | tctgaggatgtgccttgc | | | | 1 | | 4 | | 0 | | 3 | | 2 | | 0 | | 5 | 0 |
| kb742750.1_2171_mature | kb742750.1_2171_mature | | ctcccactgctgcacttgactagtc | | | | 157 | | 169 | | 397 | | 318 | | 279 | | 170 | | 316 | 125 |
| kb816396.1_25096_star | kb816396.1_25096_star | | ttaatttttgtttttacttcca | | | | 0 | | 0 | | 0 | | 0 | | 0 | | 0 | | 2 | 0 |
| kb744462.1_23603_star | kb744462.1_23603_star | | tcgtgcaccaggatcagtgct | | | | 2 | | 0 | | 0 | | 4 | | 0 | | 1 | | 1 | 1 |
| kb743818.1_19948_star | kb743818.1_19948_star | | tttttaccagctgtcctcgct | | | | 1 | | 1 | | 0 | | 1 | | 1 | | 0 | | 0 | 0 |
| kb742693.1_8763_mature | kb742693.1_8763_mature | | gaattccgggcttatgtgaaagtt | | | | 0 | | 0 | | 1 | | 0 | | 3 | | 0 | | 0 | 0 |
| kb742668.1_5611_mature | kb742668.1_5611_mature | | ttgaagaatgtgtgaagattgctaa | | | | 1 | | 3 | | 1 | | 4 | | 2 | | 2 | | 4 | 1 |
| kb744022.1_20234_star | kb744022.1_20234_star | | gtgtttgctgacaatgcagcagtg | | | | 0 | | 4 | | 2 | | 0 | | 1 | | 0 | | 1 | 1 |
| kb742650.1_8472_star | kb742650.1_8472_star | | agacttcaccattgctgcctccatgg | | | | 0 | | 1 | | 0 | | 1 | | 1 | | 0 | | 0 | 0 |
| kb743807.1_23035_mature | kb743807.1_23035_mature | | tagcgactggcctccaactggat | | | | 3 | | 29 | | 3 | | 68 | | 2 | | 1 | | 2 | 0 |
| kb742904.1_12931_mature | kb742904.1_12931_mature | | agatttgtaagtttgagat | | | | 0 | | 1 | | 0 | | 0 | | 0 | | 0 | | 2 | 0 |
| kb742588.1_6991_mature | kb742588.1_6991_mature | | atgaggctgatcttgtgt | | | | 3 | | 1 | | 1 | | 0 | | 0 | | 0 | | 1 | 1 |
| kb743110.1_6093_star | kb743110.1_6093_star | | taggttccattccagtgctaca | | | | 2 | | 0 | | 4 | | 2 | | 0 | | 0 | | 1 | 0 |
| kb742554.1_1746_star | kb742554.1_1746_star | | agcagtactgcatttgcacata | | | | 1 | | 1 | | 2 | | 4 | | 2 | | 0 | | 2 | 3 |
| kb742605.1_4544_mature | kb742605.1_4544_mature | | cagtgacagaccctgcagtgact | | | | 16 | | 25 | | 2 | | 51 | | 7 | | 3 | | 3 | 3 |
| kb746576.1_24553_star | kb746576.1_24553_star | | gtagggactggttttttagc | | | | 1 | | 2 | | 1 | | 0 | | 0 | | 0 | | 0 | 0 |
| kb744344.1_22131_star | kb744344.1_22131_star | | gccacgtgaagcaagccaggcgtccg | | | | 1 | | 2 | | 2 | | 0 | | 0 | | 0 | | 0 | 0 |
| kb742404.1_2825_star | kb742404.1_2825_star | | ttatggggaatcatacgtccttgg | | | | 0 | | 5 | | 2 | | 1 | | 0 | | 1 | | 0 | 0 |
| kb742547.1_16706_mature | kb742547.1_16706_mature | | aaaagaactaggacgaagggc | | | | 0 | | 2 | | 3 | | 1 | | 1 | | 0 | | 2 | 3 |
| kb744014.1_19188_star | kb744014.1_19188_star | | tcaagttccacttgcct | | | | 0 | | 0 | | 0 | | 3 | | 3 | | 0 | | 0 | 0 |
| kb742388.1_5741_mature | kb742388.1_5741_mature | | aatctcagcagggctctg | | | | 0 | | 0 | | 0 | | 1 | | 2 | | 0 | | 0 | 5 |
| kb743446.1_11681_mature | kb743446.1_11681_mature | | ggtacagggagtagaaatgtg | | | | 0 | | 5 | | 0 | | 0 | | 1 | | 0 | | 0 | 0 |
| kb742515.1_4126_mature | kb742515.1_4126_mature | | ctcagatggtgattactgtacc | | | | 1 | | 5 | | 1 | | 0 | | 0 | | 0 | | 7 | 0 |
| kb742478.1_20553_star | kb742478.1_20553_star | | atatgtctgagtttggaatg | | | | 1 | | 0 | | 0 | | 1 | | 0 | | 0 | | 0 | 0 |
| kb743220.1_18068_star | kb743220.1_18068_star | | ctactctatcataaacaaacc | | | | 0 | | 0 | | 1 | | 0 | | 0 | | 1 | | 0 | 0 |
| kb742977.1_9640_star | kb742977.1_9640_star | | gtgaggacggagccagc | | | | 0 | | 2 | | 0 | | 0 | | 0 | | 2 | | 0 | 0 |
| kb743260.1_3328_mature | kb743260.1_3328_mature | | tgctttgcagtcttctgac | | | | 3 | | 3 | | 9 | | 5 | | 1 | | 7 | | 6 | 0 |
| kb742554.1_1806_mature | kb742554.1_1806_mature | | ctgtcatgatgtgctacgggaat | | | | 0 | | 0 | | 4 | | 1 | | 0 | | 0 | | 3 | 0 |
| kb742513.1_2628_star | kb742513.1_2628_star | | tgcttacgggaagtgga | | | | 0 | | 1 | | 0 | | 0 | | 0 | | 0 | | 0 | 0 |
| kb742535.1_7731_star | kb742535.1_7731_star | | gctataaatacttttgcatcagcact | | | | 3 | | 2 | | 2 | | 2 | | 0 | | 0 | | 3 | 1 |
| kb745353.1_23445_mature | kb745353.1_23445_mature | | attgatggaatctgaatg | | | | 0 | | 1 | | 1 | | 6 | | 0 | | 1 | | 0 | 0 |
| kb742543.1_11181_mature | kb742543.1_11181_mature | | aagaacatgtagcatttc | | | | 1 | | 3 | | 0 | | 0 | | 1 | | 0 | | 0 | 0 |
| kb742810.1_4972_mature | kb742810.1_4972_mature | | tggttggattctgtcct | | | | 9 | | 22 | | 15 | | 12 | | 45 | | 19 | | 35 | 3 |
| kb744256.1_9631_mature | kb744256.1_9631_mature | | tatattgtaaatggctct | | | | 0 | | 3 | | 1 | | 1 | | 0 | | 0 | | 0 | 0 |
| kb743464.1_14553_mature | kb743464.1_14553_mature | | gggattatgactgaacgca | | | | 16 | | 33 | | 17 | | 9 | | 6 | | 8 | | 8 | 9 |
| kb742464.1_2384_mature | kb742464.1_2384_mature | | ttgtgtttgtgtttctcttcagt | | | | 7 | | 3 | | 3 | | 12 | | 3 | | 3 | | 5 | 3 |
| kb742553.1_13408_star | kb742553.1_13408_star | | ggcgagggggaaggtgtctggtgag | | | | 0 | | 4 | | 1 | | 0 | | 0 | | 0 | | 2 | 0 |
| kb742520.1_11487_star | kb742520.1_11487_star | | ttcaaatatcacactcatg | | | | 0 | | 0 | | 0 | | 0 | | 0 | | 1 | | 0 | 0 |
| kb742697.1_16950_mature | kb742697.1_16950_mature | | gattactgcgctttgttccttgcc | | | | 0 | | 0 | | 1 | | 0 | | 0 | | 0 | | 3 | 0 |
| kb743040.1_2799_star | kb743040.1_2799_star | | tgtcagggcagagttcgtggctcaca | | | | 8 | | 4 | | 10 | | 4 | | 5 | | 2 | | 1 | 16 |
| kb743246.1_10153_mature | kb743246.1_10153_mature | | tcgggagggagggacgaggc | | | | 6 | | 3 | | 9 | | 4 | | 6 | | 3 | | 6 | 2 |
| kb743396.1_14429_star | kb743396.1_14429_star | | tcctgcatttagatcatcgccg | | | | 0 | | 2 | | 2 | | 0 | | 0 | | 1 | | 0 | 0 |
| kb742668.1_5624_mature | kb742668.1_5624_mature | | aagtggtgactttgaaacc | | | | 1 | | 4 | | 3 | | 2 | | 0 | | 3 | | 2 | 1 |
| kb743005.1_3054_mature | kb743005.1_3054_mature | | actagcttctcagtttgtctta | | | | 0 | | 3 | | 2 | | 0 | | 3 | | 0 | | 5 | 3 |
| kb742382.1_14251_mature | kb742382.1_14251_mature | | gattttttgtgactagaa | | | | 0 | | 4 | | 6 | | 1 | | 1 | | 1 | | 0 | 0 |
| kb742803.1_17885_mature | kb742803.1_17885_mature | | cagtagcttccaaatgtcccagca | | | | 0 | | 2 | | 1 | | 1 | | 0 | | 0 | | 4 | 0 |
| kb742677.1_7524_star | kb742677.1_7524_star | | ctccgggctggggctggcttt | | | | 0 | | 9 | | 1 | | 27 | | 4 | | 1 | | 4 | 1 |
| kb745269.1_19546_mature | kb745269.1_19546_mature | | cggaactgcctgctgaacaggtgat | | | | 0 | | 0 | | 1 | | 2 | | 1 | | 1 | | 3 | 0 |
| kb743335.1_9717_mature | kb743335.1_9717_mature | | aggattttaaatagagaatg | | | | 0 | | 1 | | 1 | | 0 | | 0 | | 3 | | 2 | 2 |
| kb743601.1_14592_mature | kb743601.1_14592_mature | | tgtgcttttaaccgaacatac | | | | 2 | | 1 | | 0 | | 0 | | 0 | | 0 | | 2 | 0 |
| kb743366.1_17996_mature | kb743366.1_17996_mature | | ggaggtgagtggtggtacgctca | | | | 0 | | 2 | | 3 | | 0 | | 0 | | 0 | | 0 | 0 |
| kb818085.1_24982_mature | kb818085.1_24982_mature | | agatttagtcagtggtgtaca | | | | 1 | | 2 | | 5 | | 0 | | 0 | | 1 | | 0 | 0 |
| kb743948.1_17919_star | kb743948.1_17919_star | | gagttctataatgtcttt | | | | 0 | | 1 | | 0 | | 0 | | 0 | | 0 | | 1 | 0 |
| kb742384.1_20257_star | kb742384.1_20257_star | | ctcatcaaagttactcactc | | | | 0 | | 0 | | 0 | | 0 | | 0 | | 0 | | 0 | 1 |
| kb742635.1_16101_star | kb742635.1_16101_star | | acatgcagctttctgact | | | | 0 | | 0 | | 0 | | 0 | | 0 | | 2 | | 3 | 0 |
| kb742406.1_7383_star | kb742406.1_7383_star | | gaacattgcaatagtggatg | | | | 0 | | 0 | | 1 | | 0 | | 0 | | 0 | | 0 | 0 |
| kb744588.1_17421_mature | kb744588.1_17421_mature | | tatggacccaacagtgat | | | | 7 | | 21 | | 0 | | 12 | | 4 | | 1 | | 2 | 0 |
| kb744029.1_22147_mature | kb744029.1_22147_mature | | tgtgtctgtcagtaaatgca | | | | 0 | | 0 | | 3 | | 0 | | 0 | | 3 | | 1 | 2 |
| kb742563.1_12900_mature | kb742563.1_12900_mature | | cggggaggggggcgcggg | | | | 4 | | 22 | | 17 | | 11 | | 13 | | 7 | | 9 | 7 |
| kb743650.1_19108_star | kb743650.1_19108_star | | ttcctgattttccagtagtca | | | | 0 | | 1 | | 0 | | 1 | | 0 | | 0 | | 1 | 0 |
| kb744462.1_23599_mature | kb744462.1_23599_mature | | tcctcgtgcaccgggactgggg | | | | 2 | | 5 | | 2 | | 5 | | 4 | | 4 | | 2 | 5 |
| kb743040.1_2759_mature | kb743040.1_2759_mature | | tccgactggtacaaacggctcg | | | | 10 | | 10 | | 0 | | 19 | | 4 | | 6 | | 7 | 2 |
| kb742712.1_8601_star | kb742712.1_8601_star | | caccgcagggcagacgatga | | | | 0 | | 2 | | 0 | | 2 | | 0 | | 0 | | 0 | 0 |
| kb742449.1_19456_mature | kb742449.1_19456_mature | | tggacgtgatgcgatgaaaagagaa | | | | 1 | | 1 | | 1 | | 1 | | 0 | | 0 | | 2 | 0 |
| kb742799.1_11292_mature | kb742799.1_11292_mature | | tttctcagctgttgttctg | | | | 0 | | 2 | | 2 | | 0 | | 1 | | 1 | | 3 | 0 |
| kb742540.1_13389_mature | kb742540.1_13389_mature | | attggagaatttctgggcaatcg | | | | 0 | | 1 | | 0 | | 6 | | 4 | | 3 | | 4 | 1 |
| kb744259.1_24637_mature | kb744259.1_24637_mature | | tggactcttgatcttcatca | | | | 0 | | 1 | | 2 | | 1 | | 0 | | 0 | | 6 | 2 |
| kb817286.1_25038_mature | kb817286.1_25038_mature | | ttccttggatgtctgagcg | | | | 10 | | 37 | | 95 | | 45 | | 70 | | 18 | | 26 | 17 |
| kb743697.1_21516_mature | kb743697.1_21516_mature | | ttgtctgcaagttctcctagc | | | | 1 | | 7 | | 1 | | 0 | | 1 | | 1 | | 1 | 2 |
| kb742411.1_7603_star | kb742411.1_7603_star | | tactgatatacatgcagaatcaacc | | | | 0 | | 0 | | 1 | | 0 | | 0 | | 0 | | 2 | 0 |
| kb743609.1_4594_star | kb743609.1_4594_star | | agatgaagcaaaatttccatcc | | | | 1 | | 1 | | 2 | | 0 | | 0 | | 0 | | 1 | 0 |
| kb742873.1_1648_star | kb742873.1_1648_star | | tggtgtggagaggagtgcttgga | | | | 0 | | 1 | | 1 | | 1 | | 1 | | 0 | | 4 | 4 |
| kb742772.1_18989_mature | kb742772.1_18989_mature | | ccaagtctgtgattgaac | | | | 2 | | 2 | | 3 | | 5 | | 0 | | 1 | | 4 | 1 |
| kb742937.1_7573_mature | kb742937.1_7573_mature | | tgttgaattttctgtttt | | | | 1 | | 0 | | 2 | | 0 | | 3 | | 0 | | 1 | 0 |
| kb743108.1_15446_mature | kb743108.1_15446_mature | | gtaaggttccgacttgact | | | | 0 | | 1 | | 0 | | 0 | | 0 | | 0 | | 0 | 2 |
| kb744519.1_22650_mature | kb744519.1_22650_mature | | tcagacgtgctggacatacc | | | | 2 | | 24 | | 17 | | 8 | | 7 | | 5 | | 32 | 6 |
| kb743098.1_20648_star | kb743098.1_20648_star | | atgaaaagcatgttgggcagg | | | | 0 | | 0 | | 0 | | 0 | | 0 | | 1 | | 0 | 1 |
| kb744105.1_20092_mature | kb744105.1_20092_mature | | ctggctctgcagcatctgtttc | | | | 2 | | 6 | | 6 | | 2 | | 2 | | 2 | | 4 | 1 |
| kb742922.1_21237_star | kb742922.1_21237_star | | tctctttgcctcaagggccactgcattt | | | | 1 | | 1 | | 3 | | 1 | | 1 | | 0 | | 2 | 7 |
| kb743070.1_18310_mature | kb743070.1_18310_mature | | atgtgccatagactcatagact | | | | 0 | | 3 | | 1 | | 0 | | 0 | | 0 | | 1 | 0 |
| kb742864.1_10343_mature | kb742864.1_10343_mature | | aggatgtgttggttttct | | | | 0 | | 14 | | 1 | | 4 | | 2 | | 2 | | 6 | 1 |
| kb742503.1_10986_mature | kb742503.1_10986_mature | | gcaagtggactgctctctttga | | | | 10 | | 22 | | 38 | | 10 | | 3 | | 1 | | 17 | 5 |
| kb742449.1_19456_star | kb742449.1_19456_star | | ctctgagggtcacggatctgagggtccaca | | | | 1 | | 3 | | 3 | | 3 | | 2 | | 1 | | 1 | 1 |
| kb742757.1_11059_mature | kb742757.1_11059_mature | | cctcttggagaacctgggcgagc | | | | 1 | | 2 | | 4 | | 2 | | 2 | | 1 | | 3 | 1 |
| kb742887.1_1499_star | kb742887.1_1499_star | | ccagggttcaatcgga | | | | 0 | | 1 | | 0 | | 0 | | 0 | | 0 | | 1 | 0 |
| kb743590.1_5530_mature | kb743590.1_5530_mature | | agcatctccggagggcaattc | | | | 0 | | 2 | | 0 | | 0 | | 0 | | 0 | | 0 | 0 |
| kb742873.1_1665_star | kb742873.1_1665_star | | actaactcattttctatcagat | | | | 0 | | 2 | | 0 | | 0 | | 0 | | 0 | | 1 | 0 |
| kb742848.1_6910_mature | kb742848.1_6910_mature | | attctgatttttggagctc | | | | 1 | | 3 | | 0 | | 4 | | 0 | | 1 | | 2 | 1 |
| kb743073.1_9063_mature | kb743073.1_9063_mature | | cactgctggggacagagc | | | | 0 | | 1 | | 2 | | 0 | | 2 | | 5 | | 1 | 0 |
| kb744353.1_15008_mature | kb744353.1_15008_mature | | tctgatcgaggcgtcctgtggctg | | | | 0 | | 0 | | 1 | | 3 | | 2 | | 1 | | 1 | 2 |
| kb743171.1_10216_mature | kb743171.1_10216_mature | | gccgagcgtcggcgacgttgtgca | | | | 3 | | 5 | | 0 | | 2 | | 2 | | 2 | | 1 | 0 |
| kb743391.1_16599_mature | kb743391.1_16599_mature | | ttggaagaccaggctctggtt | | | | 0 | | 2 | | 1 | | 2 | | 0 | | 0 | | 0 | 1 |
| kb742810.1_4993_star | kb742810.1_4993_star | | tgtcacaatagaaaagattgctt | | | | 0 | | 0 | | 1 | | 0 | | 0 | | 0 | | 0 | 0 |
| kb742808.1_108_mature | kb742808.1_108_mature | | tggactttgggctatttggcttctc | | | | 0 | | 3 | | 0 | | 0 | | 5 | | 1 | | 2 | 0 |
| kb742904.1_12931_star | kb742904.1_12931_star | | cttttcttgccattctta | | | | 0 | | 0 | | 2 | | 0 | | 1 | | 0 | | 0 | 0 |
| kb745238.1_19987_star | kb745238.1_19987_star | | ccttcagcacgcagactgcccacgtga | | | | 1 | | 4 | | 1 | | 0 | | 0 | | 1 | | 1 | 1 |
| kb742845.1_5842_star | kb742845.1_5842_star | | ctggggagctgcagtaggtgtgc | | | | 2 | | 3 | | 3 | | 0 | | 1 | | 1 | | 1 | 2 |
| kb742811.1_13111_mature | kb742811.1_13111_mature | | atggctggactgtcaccgcatgc | | | | 4 | | 6 | | 2 | | 1 | | 4 | | 4 | | 10 | 2 |
| kb742618.1_11461_mature | kb742618.1_11461_mature | | tgctggctgggcctgata | | | | 1 | | 4 | | 0 | | 36 | | 12 | | 1 | | 1 | 3 |
| kb743058.1_6261_mature | kb743058.1_6261_mature | | tctgtgtgtgtgtctgtacatg | | | | 13 | | 12 | | 4 | | 8 | | 5 | | 14 | | 31 | 1 |
| kb743226.1_4903_star | kb743226.1_4903_star | | ttcggcgatcagctcctgc | | | | 5 | | 0 | | 4 | | 1 | | 0 | | 2 | | 2 | 2 |
| kb743696.1_14044_mature | kb743696.1_14044_mature | | gaaggacactccactcta | | | | 0 | | 0 | | 3 | | 0 | | 0 | | 0 | | 3 | 2 |
| kb743833.1_10822_star | kb743833.1_10822_star | | gtgctgggttaaag | | | | 0 | | 0 | | 0 | | 1 | | 0 | | 0 | | 0 | 0 |
| kb743240.1_19701_mature | kb743240.1_19701_mature | | gtggatgtcagagttacc | | | | 0 | | 4 | | 7 | | 0 | | 1 | | 4 | | 2 | 0 |
| kb742966.1_4461_mature | kb742966.1_4461_mature | | tctgtccagtgttcatatgatgtct | | | | 0 | | 6 | | 0 | | 5 | | 0 | | 1 | | 0 | 0 |
| kb758252.1_26056_mature | kb758252.1_26056_mature | | cggagccgtggggggggaca | | | | 0 | | 2 | | 6 | | 2 | | 0 | | 0 | | 0 | 5 |
| kb742588.1_7035_star | kb742588.1_7035_star | | atagctcttgatatagctccgaaaa | | | | 0 | | 0 | | 0 | | 0 | | 0 | | 0 | | 4 | 0 |
| kb742850.1_14445_mature | kb742850.1_14445_mature | | tcaggatatgaagaacatg | | | | 0 | | 6 | | 3 | | 0 | | 1 | | 0 | | 0 | 0 |
| kb743403.1_19668_mature | kb743403.1_19668_mature | | gagggtgggagggtggca | | | | 4 | | 6 | | 6 | | 3 | | 2 | | 0 | | 1 | 0 |
| kb746388.1_21634_star | kb746388.1_21634_star | | tgaggttttggagtgttcttgg | | | | 6 | | 31 | | 15 | | 11 | | 5 | | 7 | | 14 | 2 |
| kb743402.1_5888_star | kb743402.1_5888_star | | gttcaaatctcagcagaac | | | | 2 | | 1 | | 1 | | 3 | | 0 | | 0 | | 0 | 0 |
| kb742675.1_10544_mature | kb742675.1_10544_mature | | tgtttgctgttggaagaac | | | | 0 | | 1 | | 3 | | 1 | | 1 | | 0 | | 11 | 4 |
| kb743364.1_9938_mature | kb743364.1_9938_mature | | caagattctgaatatttttttg | | | | 2 | | 1 | | 5 | | 1 | | 1 | | 0 | | 3 | 0 |
| kb744214.1_20085_mature | kb744214.1_20085_mature | | tagaaagacagatgagctt | | | | 1 | | 4 | | 1 | | 0 | | 1 | | 5 | | 2 | 0 |
| kb742430.1_11435_star | kb742430.1_11435_star | | tcatcttatcttcctta | | | | 0 | | 0 | | 0 | | 0 | | 0 | | 0 | | 1 | 0 |
| kb744430.1_14517_star | kb744430.1_14517_star | | tgtagtccccaacaggaa | | | | 0 | | 0 | | 0 | | 0 | | 0 | | 0 | | 1 | 0 |
| kb742887.1_1523_mature | kb742887.1_1523_mature | | aggaaagtacggcagaagtcttt | | | | 0 | | 0 | | 5 | | 1 | | 0 | | 1 | | 5 | 0 |
| kb746576.1_24553_mature | kb746576.1_24553_mature | | tgccaagccggtcctgttag | | | | 0 | | 2 | | 0 | | 0 | | 1 | | 0 | | 0 | 0 |
| kb743025.1_20344_mature | kb743025.1_20344_mature | | tctatggctgtgtttgaac | | | | 4 | | 2 | | 6 | | 0 | | 3 | | 1 | | 6 | 2 |
| kb742390.1_22076_mature | kb742390.1_22076_mature | | ctgcactgtgaaagaaagc | | | | 3 | | 16 | | 3 | | 16 | | 5 | | 1 | | 0 | 0 |
| kb744212.1_8552_star | kb744212.1_8552_star | | gccacatcatcttgcaatgactagt | | | | 2 | | 8 | | 0 | | 10 | | 6 | | 0 | | 1 | 4 |
| kb744486.1_15068_star | kb744486.1_15068_star | | gagggcaaggcttgcactgaacaga | | | | 0 | | 1 | | 5 | | 1 | | 1 | | 0 | | 0 | 1 |
| kb743226.1_4913_star | kb743226.1_4913_star | | ttagcttttcatgtagtgtttttt | | | | 0 | | 1 | | 0 | | 1 | | 0 | | 1 | | 5 | 0 |
| kb742897.1_21528_star | kb742897.1_21528_star | | tttttcctctgagtcttgttc | | | | 2 | | 1 | | 0 | | 0 | | 0 | | 3 | | 1 | 0 |
| kb743446.1_11706_star | kb743446.1_11706_star | | nnnggaagggggcagcggggggtg | | | | 2 | | 1 | | 7 | | 0 | | 3 | | 1 | | 3 | 3 |
| kb744722.1_10794_star | kb744722.1_10794_star | | gttctacaccttttttttt | | | | 0 | | 0 | | 1 | | 1 | | 0 | | 1 | | 2 | 2 |
| kb742855.1_12687_mature | kb742855.1_12687_mature | | agaggacaggagcttgagcacaca | | | | 4 | | 2 | | 4 | | 1 | | 3 | | 2 | | 2 | 0 |
| kb742693.1_8767_star | kb742693.1_8767_star | | cagtgttgaaaacatccagccg | | | | 1 | | 1 | | 2 | | 3 | | 0 | | 0 | | 1 | 0 |
| kb742712.1_8641_star | kb742712.1_8641_star | | tgccacgtgctgaggtagacac | | | | 0 | | 5 | | 2 | | 1 | | 7 | | 0 | | 2 | 0 |
| kb742772.1_18996_star | kb742772.1_18996_star | | cccggagccgatcgcctcaacg | | | | 0 | | 4 | | 2 | | 0 | | 4 | | 0 | | 1 | 1 |
| kb743553.1_21115_star | kb743553.1_21115_star | | gtggagctgggtgcaaagaa | | | | 0 | | 0 | | 1 | | 1 | | 3 | | 1 | | 0 | 1 |
| kb742809.1_13889_mature | kb742809.1_13889_mature | | tcctgtcttctctctgcacagg | | | | 2 | | 13 | | 2 | | 3 | | 4 | | 2 | | 5 | 1 |
| kb742870.1_17110_star | kb742870.1_17110_star | | tcaccgatgggaccactcctgggg | | | | 0 | | 1 | | 1 | | 1 | | 0 | | 0 | | 4 | 1 |
| kb743399.1_14114_star | kb743399.1_14114_star | | tttgctaaggagatcttctg | | | | 1 | | 2 | | 1 | | 2 | | 0 | | 0 | | 1 | 0 |
| kb743818.1_19948_mature | kb743818.1_19948_mature | | cggatggagaatggtagagg | | | | 0 | | 3 | | 0 | | 0 | | 0 | | 0 | | 1 | 0 |
| kb742943.1_5715_mature | kb742943.1_5715_mature | | cgatgatggactgagggtt | | | | 0 | | 3 | | 5 | | 0 | | 1 | | 0 | | 1 | 0 |
| kb742680.1_6654_mature | kb742680.1_6654_mature | | aaggatgtggagttgct | | | | 1 | | 3 | | 6 | | 4 | | 1 | | 1 | | 2 | 6 |
| kb742969.1_1049_mature | kb742969.1_1049_mature | | cggggcggggcggggcggg | | | | 22 | | 22 | | 73 | | 33 | | 7 | | 18 | | 26 | 42 |
| kb743016.1_20435_star | kb743016.1_20435_star | | ctcgtccctcccgttg | | | | 0 | | 1 | | 0 | | 0 | | 0 | | 0 | | 0 | 0 |
| kb743120.1_18166_star | kb743120.1_18166_star | | gagccaatggcgaaaacagcgga | | | | 0 | | 0 | | 1 | | 0 | | 0 | | 0 | | 0 | 0 |
| kb742907.1_4739_star | kb742907.1_4739_star | | catacagacatcctttggg | | | | 1 | | 0 | | 2 | | 0 | | 0 | | 0 | | 0 | 1 |
| kb742847.1_16291_mature | kb742847.1_16291_mature | | tgctgaggtcaaggtagacc | | | | 25 | | 321 | | 6 | | 194 | | 44 | | 0 | | 9 | 5 |
| kb742464.1_2369_mature | kb742464.1_2369_mature | | ccactggtggtagagactgc | | | | 1 | | 12 | | 5 | | 5 | | 2 | | 0 | | 1 | 0 |
| kb742586.1_16543_mature | kb742586.1_16543_mature | | cttcctcttcaagagctcgggct | | | | 0 | | 4 | | 4 | | 5 | | 2 | | 4 | | 1 | 8 |
| kb742637.1_18796_star | kb742637.1_18796_star | | cactgacctcaacacagtcaaggagca | | | | 1 | | 4 | | 2 | | 6 | | 3 | | 0 | | 2 | 0 |
| kb743868.1_15927_mature | kb743868.1_15927_mature | | cagatatgaagaagctga | | | | 3 | | 18 | | 42 | | 4 | | 5 | | 0 | | 22 | 4 |
| kb742647.1_12247_mature | kb742647.1_12247_mature | | aggaacattgtaggtatgaacca | | | | 1 | | 2 | | 3 | | 1 | | 2 | | 4 | | 1 | 1 |
| kb743529.1_12860_mature | kb743529.1_12860_mature | | ctcagctcggtcgatctg | | | | 0 | | 0 | | 0 | | 1 | | 3 | | 0 | | 0 | 0 |
| kb742830.1_16492_star | kb742830.1_16492_star | | tgacagttctagggcttcttccat | | | | 2 | | 0 | | 4 | | 0 | | 1 | | 5 | | 0 | 0 |
| kb742921.1_16137_mature | kb742921.1_16137_mature | | attgaagtgacgtgaggcc | | | | 0 | | 3 | | 1 | | 4 | | 0 | | 0 | | 0 | 0 |
| kb743561.1_15977_star | kb743561.1_15977_star | | cacctgcgaggtttgagctcggtt | | | | 1 | | 13 | | 2 | | 0 | | 0 | | 2 | | 2 | 2 |
| kb744208.1_14955_star | kb744208.1_14955_star | | ttaatgaatttgtgttcctgac | | | | 0 | | 3 | | 0 | | 2 | | 1 | | 0 | | 0 | 0 |
| kb742833.1_43_mature | kb742833.1_43_mature | | gtgactgtgttaccctgca | | | | 0 | | 11 | | 10 | | 2 | | 1 | | 6 | | 13 | 1 |
| kb743561.1_15977_mature | kb743561.1_15977_mature | | tcgggctgaatccttgcgtgct | | | | 2 | | 17 | | 22 | | 5 | | 5 | | 0 | | 0 | 0 |
| kb743670.1_4943_mature | kb743670.1_4943_mature | | agactattggtagaccctct | | | | 0 | | 0 | | 0 | | 0 | | 1 | | 1 | | 3 | 1 |
| kb742489.1_7317_mature | kb742489.1_7317_mature | | aggacagataaggtgaagaagtgt | | | | 3 | | 2 | | 0 | | 1 | | 0 | | 0 | | 0 | 1 |
| kb742777.1_8154_mature | kb742777.1_8154_mature | | gtgatctgtctaaacttgc | | | | 2 | | 5 | | 2 | | 0 | | 0 | | 0 | | 2 | 1 |
| kb743364.1_9944_mature | kb743364.1_9944_mature | | gggtatgttacgttagaattct | | | | 0 | | 0 | | 4 | | 1 | | 0 | | 0 | | 1 | 0 |
| kb742478.1_20560_star | kb742478.1_20560_star | | atatgtctgagtttggaatt | | | | 0 | | 0 | | 0 | | 1 | | 0 | | 1 | | 3 | 1 |
| kb742446.1_12973_star | kb742446.1_12973_star | | ctgaggctttgggcaaggg | | | | 0 | | 2 | | 0 | | 0 | | 0 | | 0 | | 0 | 1 |
| kb744204.1_11870_star | kb744204.1_11870_star | | cagcaggattccttct | | | | 0 | | 0 | | 0 | | 0 | | 2 | | 0 | | 1 | 0 |
| kb742777.1_8175_star | kb742777.1_8175_star | | tcccagttccattcctgttatg | | | | 2 | | 4 | | 1 | | 6 | | 0 | | 0 | | 0 | 1 |
| kb742840.1_6695_star | kb742840.1_6695_star | | ccgtggggtgccgctgagcgag | | | | 0 | | 0 | | 3 | | 0 | | 0 | | 0 | | 0 | 0 |
| kb742995.1_20813_mature | kb742995.1_20813_mature | | ttttagggccaggaacagc | | | | 0 | | 3 | | 1 | | 1 | | 0 | | 0 | | 3 | 0 |
| kb742675.1_10544_star | kb742675.1_10544_star | | ctgtttaaagtaagcttt | | | | 2 | | 0 | | 0 | | 0 | | 0 | | 0 | | 0 | 0 |
| kb743590.1_5530_star | kb743590.1_5530_star | | attgctctcaggagttgtctc | | | | 0 | | 1 | | 0 | | 0 | | 0 | | 1 | | 0 | 0 |
| kb742777.1_8210_star | kb742777.1_8210_star | | ccacgttctgaaagaaa | | | | 1 | | 2 | | 1 | | 2 | | 4 | | 0 | | 3 | 0 |
| kb742739.1_8235_star | kb742739.1_8235_star | | ataatcttgtgtgaaacact | | | | 3 | | 5 | | 1 | | 9 | | 0 | | 0 | | 15 | 1 |
| kb742969.1_1034_star | kb742969.1_1034_star | | aagatcctagccccaccaccta | | | | 0 | | 1 | | 1 | | 0 | | 0 | | 0 | | 2 | 0 |
| kb742900.1_11376_mature | kb742900.1_11376_mature | | tccttgaattactctgagg | | | | 0 | | 4 | | 3 | | 2 | | 4 | | 0 | | 0 | 1 |
| kb742811.1_13121_star | kb742811.1_13121_star | | tcaccattatccatgttc | | | | 0 | | 2 | | 0 | | 1 | | 0 | | 0 | | 0 | 0 |
| kb742467.1_8783_star | kb742467.1_8783_star | | ggacatgctgctggctcacatcc | | | | 1 | | 3 | | 3 | | 2 | | 3 | | 2 | | 0 | 4 |
| kb742520.1_11487_mature | kb742520.1_11487_mature | | attttgtggtgctgaatg | | | | 0 | | 8 | | 5 | | 1 | | 3 | | 4 | | 5 | 0 |
| kb744739.1_20150_mature | kb744739.1_20150_mature | | ttctgctgaattttttatc | | | | 0 | | 0 | | 1 | | 1 | | 0 | | 0 | | 3 | 0 |
| kb744353.1_15020_mature | kb744353.1_15020_mature | | ctgttagattctggaaactgac | | | | 4 | | 2 | | 1 | | 3 | | 3 | | 1 | | 0 | 0 |
| kb742616.1_12593_star | kb742616.1_12593_star | | tgcatttagctgtgtgcggtga | | | | 0 | | 1 | | 0 | | 2 | | 2 | | 2 | | 0 | 1 |
| kb742459.1_3581_star | kb742459.1_3581_star | | tacagaatatcaaacatttgaag | | | | 0 | | 2 | | 0 | | 4 | | 0 | | 0 | | 2 | 0 |
| kb802439.1_25550_star | kb802439.1_25550_star | | ttgttctgcagcttcagttcctgagc | | | | 3 | | 15 | | 13 | | 4 | | 5 | | 10 | | 6 | 2 |
| kb742439.1_3137_mature | kb742439.1_3137_mature | | cagatgaggtgcctgaagg | | | | 0 | | 15 | | 2 | | 6 | | 2 | | 0 | | 0 | 0 |
| kb788489.1_25797_mature | kb788489.1_25797_mature | | acagactgaattctgagaagtgct | | | | 2 | | 15 | | 3 | | 3 | | 1 | | 3 | | 2 | 1 |
| kb743013.1_13175_mature | kb743013.1_13175_mature | | tctggtttggtagagagcga | | | | 3 | | 5 | | 1 | | 0 | | 1 | | 3 | | 5 | 2 |
| kb742479.1_264_mature | kb742479.1_264_mature | | tttgaagctttgaagaaa | | | | 1 | | 0 | | 0 | | 3 | | 0 | | 3 | | 3 | 0 |
| kb754269.1_25092_mature | kb754269.1_25092_mature | | ctcctgcctggctcgcca | | | | 9 | | 15 | | 13 | | 10 | | 9 | | 13 | | 12 | 10 |
