# Supplementary figures and images for: High-throughput sequencing analysis identified microRNAs associated with egg production in ducks ovaries
Source: PeerJ. 2020 Feb 4;8:e8440. doi: 10.7717/peerj.8440 (PMC7006514; doi:10.7717/peerj.8440)

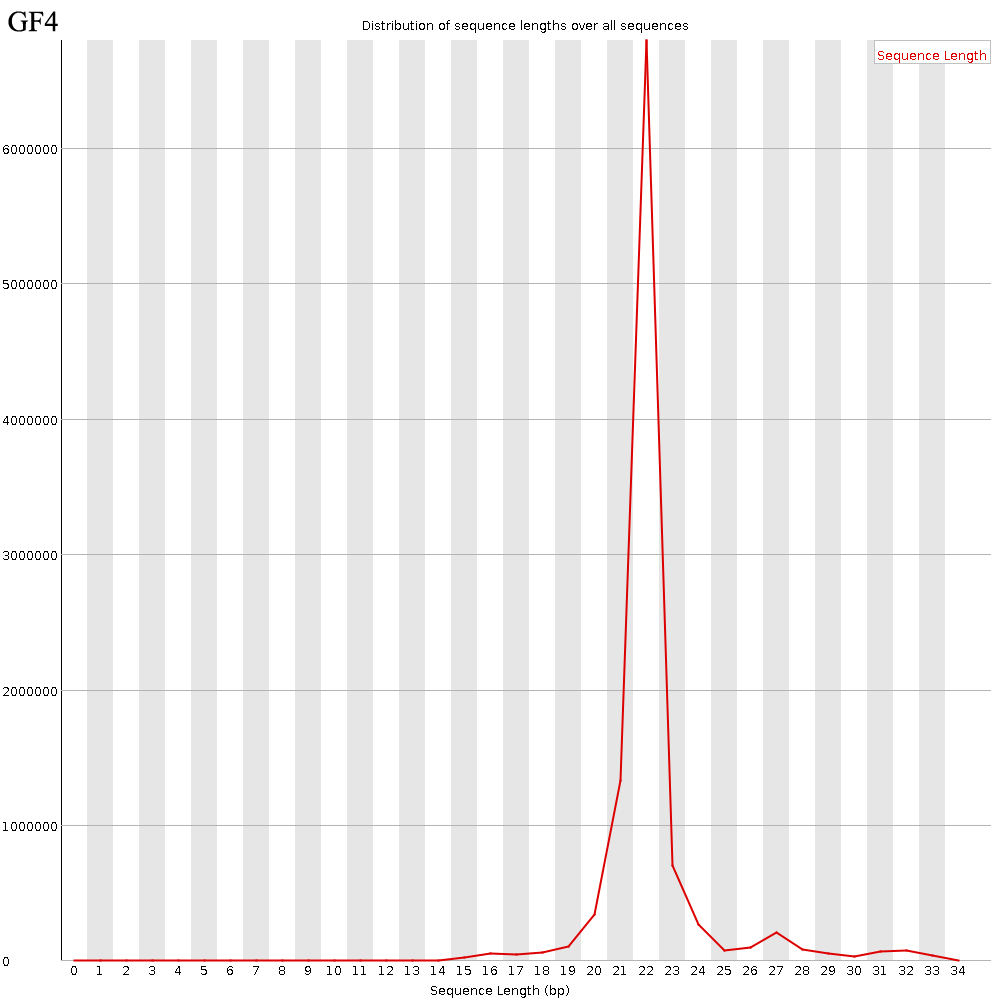

Supplement: Figure S1 — Note: GF4 represent the ovary libraries from fourth group of Jian Chang ducks. [file peerj-08-8440-s002.jpg]

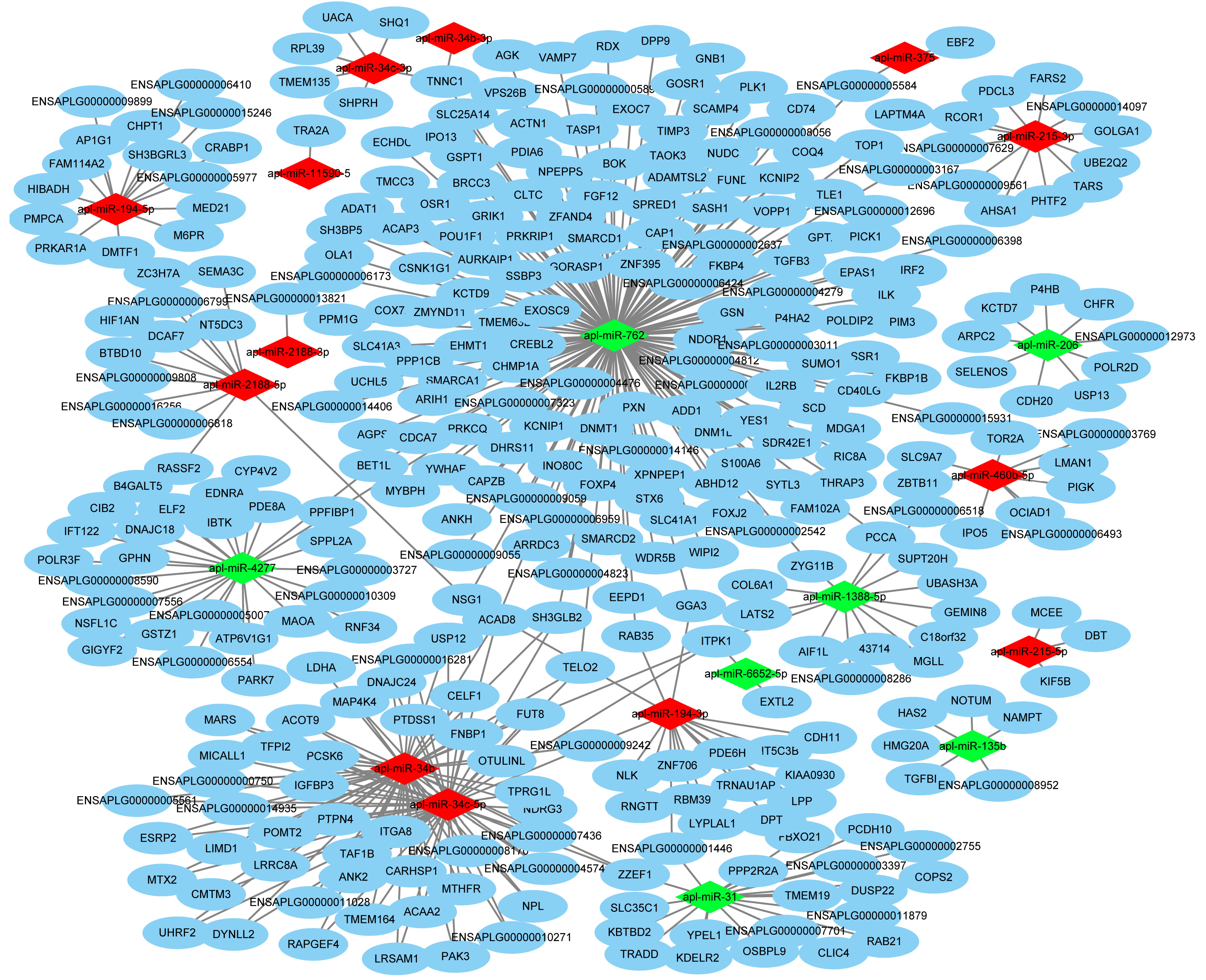

Supplement: Figure S2 — Orange indicate DEmiRNA, blue indicate target genes. Blue oval represents the target gene, red diamond represents the up-regulated DEmiRNAs in JD, and green represents the down-regulated DEmiRNAs in JD. [file peerj-08-8440-s003.jpg]
